# Supplementary material for: Effects of Copy Number Variations in the Plectin (PLEC) Gene on the Growth Traits and Meat Quality of Leizhou Black Goats
Source: Animals (Basel). 2023 Nov 25;13(23):3651. doi: 10.3390/ani13233651 (PMC10705363; doi:10.3390/ani13233651)
Supplement: Supplementary file 1 [file animals-13-03651-s001.zip › Supplementary Material Figures and Tables.pdf]

Supplementary Materials

Table S1. Primer information.

Gene name		5'-3'	Length	Note
PLEC	F	GCTCAGAACCCTGGAGACAG	222	Exonic-CNV
	R	TCTCAGCAGGTTGGGGTTTC		
	F	TGTTCGGACGGCACTTCTTT	206	Intronic-CNV
	R	CCTGTGAGAGACGCCTTGAG		
	F	GGGGTAGGGGACAGCTTAGA	114	Intronic-CNV
	R	TTAATAGGGCATGGTGCGGC		
MC1R	F	GGGCAGTCCCTTGACAAAGA	129	CNV-qPCR
	R	ATCTCCCCAGCCTCCTCATT		
PLEC	F	CAAAGTTGCACCGCACAGAG	232	mRNA-qPCR
	R	TGCAAGGCCTGAGTCTCCT		
GADPH	F	TGAAGGTCGGTGTGAACGGATTTGG	277	mRNA-qPCR
	R	ACGACATACTCAGCACCAGCATCAC		

Table S2. The association analysis between the traits and CNV-2 in the goat *PLEC* gene.

Growth Traits	CNV Types (Mean ± SE)			*p* values
	**Loss （1 Copy） (*n* = 31)**	**Normal (2 Copies) (*n* =245)**	**Gain (⩾3 Copies) (*n* = 140)**	
body height (BH,cm)	51.74±0.24	52.07±0.09	51.65±0.10	0.241
body oblique length (BOL, cm)	54.37±0.25	54.76±0.10	55.12±0.08	0.417
chest circumference(CC, cm)	56.94±0.35	57.88±0.13	58.09±0.12	0.072
body weight (BW, kg)	18.99±0.24	19.27±0.09	20.31±0.09	0.061
cannon circumference(CAC, cm)	7.17±0.05	7.19±0.01	7.33±0.04	0.104
**Carcass Traits & Meat Quality**	**Loss （1 Copy） (*n* = 9)**	**Normal (2 Copies) (*n* =42)**	**Gain (⩾3 Copies) (*n* =29)**	
Carcass weight (CW, kg)	8.91^{b}±0.24	9.52^{b}±0.16	10.01^{a}±0.22	0.044
cross-section area of *longissimus dorsi lumbois* muscle(CALM, cm^2)	7.44±0.24	7.67±0.06	8.14±0.20	0.058
water loss rate (WLR, %)	4.83±0.09	4.81±0.18	4.87±0.14	0.366
water holding capacity (WHC, %)	4.80±0.14	4.79±0.05	4.94±0.06	0.217
shear stress (SS, N)	49.10^{a}±0.66	47.98^{b}±0.17	46.49^{b}±0.32	0.037

Table S3. The association analysis between the traits and CNV-3 in the goat *PLEC* gene.

Growth Traits	CNV Types (Mean ± SE)			*p* values
	**Loss**<br>**（1 Copy）**<br>**(*n* = 52)**	**Normal**<br>**(2 Copies)**<br>**(*n* =265)**	**Gain**<br>**(⩾3 Copies)**<br>**(*n* = 98)**	
body height (BH,cm)	52.03±0.22	52.19±0.09	51.77±0.09	0.437
body oblique length (BOL, cm)	54.01±0.23	54.33±0.08	55.48±0.12	0.210
chest circumference(CC, cm)	57.32±0.23	56.77±0.12	58.17±0.18	0.357
body weight (BW, kg)	18.64±0.24	19.72±0.10	19.59±0.14	0.175
cannon circumference(CAC, cm)	7.14±0.03	7.21±0.01	7.23±0.03	0.218
**Carcass Traits & Meat Quality**	**Loss**<br>**（1 copy）**<br>**(*n* = 21)**	**Normal**<br>**(2 copies)**<br>**(*n* =41)**	**Gain**<br>**(⩾3 copies)**<br>**(*n* = 18)**	
Carcass weight (CW, kg)	9.12^{b}±0.13	9.71^{a}±0.23	9.64^{a}±0.43	0.179
cross-section area of *longissimus dorsi lumbois* muscle(CALM, cm^2)	7.50±0.11	7.61±0.42	8.04±0.44	0.097
water loss rate (WLR, %)	4.81±0.08	4.85±0.07	4.75±0.22	0.772
water holding capacity (WHC, %)	4.93±0.14	4.81±0.03	4.86±0.10	0.391
shear stress (SS, N)	48.81±0.33	48.09±0.22	47.79±0.21	0.064
