# Supplementary material for: Effects of Copy Number Variations in the Plectin (PLEC) Gene on the Growth Traits and Meat Quality of Leizhou Black Goats
Source: Animals (Basel). 2023 Nov 25;13(23):3651. doi: 10.3390/ani13233651 (PMC10705363; doi:10.3390/ani13233651)
Supplement: Supplementary file 1 [file animals-13-03651-s001.zip › Supplementary Material S1 swissmodel-PLEC-CNV Report.pdf]

SWISS-MODEL Homology Modelling Report

Model Building Report

This document lists the results for the homology modelling project "PLEC-CNV" submitted to SWISS-MODEL workspace on Nov. 16, 2023, 11:15 a.m..The submitted primary amino acid sequence is given in Table T1.

If you use any results in your research, please cite the relevant publications:

- Waterhouse, A., Bertoni, M., Bienert, S., Studer, G., Tauriello, G., Gumienny, R., Heer, F.T., de Beer, T.A.P., Rempfer, C., Bordoli, L., Lepore, R., Schwede, T. SWISS-MODEL: homology modelling of protein structures and complexes. Nucleic Acids Res. 46(W1), W296-W303 (2018). doi>
- Bienert, S., Waterhouse, A., de Beer, T.A.P., Tauriello, G., Studer, G., Bordoli, L., Schwede, T. The SWISS-MODEL Repository - new features and functionality. Nucleic Acids Res. 45, D313-D319 (2017). doi>
- Studer, G., Tauriello, G., Bienert, S., Biasini, M., Johner, N., Schwede, T. ProMod3 - A versatile homology modelling toolbox. PLOS Comp. Biol. 17(1), e1008667 (2021). doi>
- Studer, G., Rempfer, C., Waterhouse, A.M., Gumienny, G., Haas, J., Schwede, T. QMEANDisCo - distance constraints applied on model quality estimation. Bioinformatics 36, 1765-1771 (2020). doi>
- Bertoni, M., Kiefer, F., Biasini, M., Bordoli, L., Schwede, T. Modeling protein quaternary structure of homo- and hetero-oligomers beyond binary interactions by homology. Scientific Reports 7 (2017). doi>

Results

The SWISS-MODEL template library (SMTL version 2023-11-08, PDB release 2023-11-03) was searched with for evolutionary related structures matching the target sequence in Table T1. For details on the template search, see Materials and Methods. Overall 1144 templates were found (Table T2).

Models

The following models were built (see Materials and Methods "Model Building"):

Model #01	File	Built with	Oligo-State	Ligands	GMQE
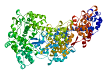	PDB	ProMod3 3.3.0	monomer	None	0.33

Template	Seq Identity	Oligo-state	QSQE	Found by	Method	Resolution	Seq Similarity	Range	Coverage	Description
H9FGT6.1.A	90.19	monomer	-	AFDB search	AlphaFold v2	-	0.57	1058 - 2239	0.43	Plectin isoform 1a

The template contained no ligands.

```
Target     RALAEEAKRQRQLAEEDAARQRAEAERVLSEKLAAISEATRLKTEAEIALKEKEAENERLRRLAEDEAFQRRRLEEQAAQ
H9FGT6.1.A--------------------------------------------------------------------------------

Target     HKADIEERLAQLRKASESELERQKGLVEDTLRQRRQVEEEILALKASFEKAAAGKAELELELGRIRGNAEDTLRSKEQAE
H9FGT6.1.A--------------------------------------------------------------------------------

Target     QEAARQRQLAAEEERRRREAEERVQKSLAAEEEAARQRKAALEEVERLKAKVEEARRLRERAEHESVRQLQLAQEAAQKR
H9FGT6.1.A--------------------------------------------------------------------------------

Target     LQAEEKAHAFAVQQKEQELQQTLQQEQSVLEKLRSEAEAARRAAEEAEEARERAEREAAQSRQRVEEAERLKQAAEEQAQ
H9FGT6.1.A--------------------------------------------------------------------------------

Target     AQAQAQAAAEKLRKEAEQEAARRAQAEQAALRQKQAADAEMEKHKKFAEQTLRQKAQVEQELTALRLKLEETDHQKSILD
H9FGT6.1.A--------------------------------------------------------------------------------

Target     QELQRLKAEVTEAARQRSQVEEELFSVRVQMEELGKLKARIEAENRALILRDKDNTQRLLQEEAEKMKQVAEEAARLSVA
H9FGT6.1.A--------------------------------------------------------------------------------

Target     AQEAARLRQLAEEDLAQQRALAEKMLKEKMQAVQEATRLKAEAELLQQQKELAQEQARRLQEDKEQMAQQLAQETQGFQR
H9FGT6.1.A--------------------------------------------------------------------------------

Target     TLETERQRQLEMSAEAERLRLRVAEMSRAQARAEEDAQRFRKQAEEIGAKLHRTELATQEKVTLVQTLETQRQQSDRDAD
H9FGT6.1.A--------------------------------------------------------------------------------
```

Target RLREAIAELEREKDKLKKEAELLQLKSEEMQTVQQEQLLQETQALQQSFLSEKDSLLQRERFIEEEKAKLERLFQDEVAK
H9FGT6.1.A--

Target AQKLREEQQRQQQQMQQEKQQLLASMEEARRRQHEAEEGVRRKQEELQLLEQQRQQQEQLLAEENRRLRERLEHLEEEHR
H9FGT6.1.A--

Target AALAHSEEITAAQAAATRALPNGQDAADGPAAEPEHAFEGLRQKVPAQQLQEAGILSTEELQRLAQGHTTVAELTQREDV
H9FGT6.1.A--

Target RRYLQGHSSIAGLLLKPANEKLTIYAALRRQLLSPGTALILLEAQAASGFLLDPVRNRRLTVTEAVKEGVVGPELHHKLL
H9FGT6.1.A--

Target SAERAVTGYKDPYTGEKISLFQAMKKDLIVREHGIRLLEAQIATGGVIDPVHSHRVPVDVAYHRGYFDEEMNRVLQDPSD
H9FGT6.1.A--

Target DTKGFFDPNTHENLTYLQLLERCVEDPETGLRLLPLTDQAAKGGELVYTDSEARDVFEKATVSAPFGKFQGKTVTIWELI
H9FGT6.1.A-----------------QLLERCVEDPETGLRLLPLTDKAAKGRELVYTDSEARDVFEKATVSAPFGKFQGKTVTIWEII

Target NSEYFTAEQRRDLLRQFRTGKVTVEKIIKIVITVIEEHEQKGQLCFQGLRALVPAAELLESGIIDWDLFRQLQLGERSVQ
H9FGT6.1.ANSEYFTAEQRRDLLRQFRTGRITVEKIIKIIITVVEEQEQKGQLCFEGLRSLVPAAELLESRVIDRELYHQLQQGERSVR

Target EVAEVEGVRRALRGSGVIAGVWLEEARQKLSIYEALKKELLQPEAAVALLEAQAGTGHVIDPATSARLTVDEAVRAGLVG
H9FGT6.1.AEVAEVDAVRRALRGANVIAGVWLEEAGQKLSVYDALKKDLLPSDVAVALLEAQAGTGHIIDPSTSARLTVDEAVRAGLVG

Target PELHEKLLSAEKAVTGYKDPYSGQSVSLFQALKKGLIPREQGLRLLDAQLSTGGTVDPSKSHRLPLDVACARGYLDEETS
H9FGT6.1.APEFHEKLLSAEKAVTGYRDPYTGQSVSLFQALKKGLIPREQGLRLLDAQLSTGGVVDPSKSHRVPLDVACARGFLDEETS

Target TALSAPRDDAKTYYDPRTWELATYSQLQQQCRPDPLTGLSLLPLSEEAARARQQELYSEVQAREAFQKATVEVPVGSFQG
H9FGT6.1.ARALSAPRADAKAYCDPSTGEPVTYSELQQRCRPDQLTGLSLLPLSEKAARARQEELYSELQARETFEKTPVDVPVGGFKG

Target RAVTIWELINSEYFTAEQRQELLRQFRTGKVTVEKIIKIVITIVEEVETTRRERLSFSGLRAPVPASELLAAGILSSSQF
H9FGT6.1.ARTVTVWELISSEYFTTEQRQELLRQFRTGKVTVEKVIKILITIVEEVETLRQERLSFSGLRAPVPASELLASRVLSRAQF

Target EQLKDGKTSVKDLSELSSLQTLLQGSGCLAGIYLEESKEKVTIYEAMRRGLLRPSTAILLLEAQAATGFLVDPVRNQRLY
H9FGT6.1.AEQLKDGKTTVKDLSELDSVRTLLQGSGCLAGIYLEDTKEKVSIYEAMRRGLLRPSTAALLLEAQAATGFLVDPVRNQRLY

Target VHEAVKAGVVGPELHEKLLSAEKAVTGYKDPYSGSTISLFQAMKKGLVVREHGIRLLEAQIATGGIIDPVHSHRVPVDVA
H9FGT6.1.AVHEAVKAGVVGPELHEQLLSAEKAVTGYRDPYSGSTISLFQAMQKGLVLRQHGIRLLEAQIATGGVIDPVHSHRVPVDVA

Target CQRGYFDEEMNRVLEDPSDDTKGFFDPNTHENLTYRQLLERCVEDPETGLRLLPLKGPEKAEVVETTRVYTEEETRRAFE
H9FGT6.1.AYRRGYFDEEMNRVLADPSDDTKGFFDPNTHENLTYLQLLERCVEDPETGLRLLPLKGAEKAEVVETTQVYTEEETRRAFE

Target ETQIDIPGGGSHGGSTMSLWEVMQSDLIPEEQRAQLMADFQAGRVTKERMIIIIIEIIEKTEIVRQQNLASYDYVRRRLT
H9FGT6.1.AETQIDIPGGGSHGGSTMSLWEVMQSDLIPEEQRAQLMADFQAGRVTKERMIIIIIEIIEKTEIIRQQGLASYDYVRRRLT

Target AEDLYEARVISRESYSLLREGTRSLREVLEAESAWRYLYGTGCVAGVYLPGSRQTLPIYQALKKGLLSAEVARLLLEAQA
H9FGT6.1.AAEDLYEARIISLETYNLLREGTRSLREALEAESAWRYLYGTGCVAGVYLPGSRQTLSIYQALKKGLLSAEVARLLLEAQA

Target ATGFLLDPVKGERLTVDEAVRKGLVGPELHDRLLSAERAVTGYRDPYTEQTISLFQAMKKDLIPAEEALRLLDAQLATGG
H9FGT6.1.AATGFLLDPVKGQRLTVDEAVRKGLVGPELHDRLLSAERAVTGYRDPYTEQTISLFQAMKKELIPTEEALRLLDAQLATGG

Target IVDPRLGFHLPLEVAYQRGYLNKDTHDQLSEPSEVRSYVDPSTDERLSYTQLLRRCRRDETSGLFLLPLSEARKLTFRGL
H9FGT6.1.AIVDPRLGFHLPLEVAYQRGYLNKDTHDQLSEPSEVRSYVDPSTDERLSYTQLLRRCRRDDGTGQLLLPLSDARKLTFRGL

Target RKQITVEELVRSHVMDEATAQRLQEGLTSIEEVSKNLQKFLEGTSSIAGVLVDATKERLSVYQAMKKGIIRPGTAFELLE
H9FGT6.1.ARKQITVEELVRSQVMDEATALQLREGLTSIEEVTKNLQKFLEGTSCIAGVFVDATKERLSVYQAMKKGIIRPGTAFELLE

Target AQAATGYVIDPIKGLKLTVEEAVRMGIVGPEFKDKLLSAERAVTGYKDPYSGKLISLFQAMKKGLILKDHGIRLLEAQIA
H9FGT6.1.AAQAATGYVIDPIKGLKLTVEEAVRMGIVGPEFKDKLLSAERAVTGYKDPYSGKLISLFQAMKKGLILKDHGIRLLEAQI-

Target TGGIIDPEESHRLPVDVAYQRGLFDEEMNEILTDPSDDTKGFFDPNTEENLTYLQLMERCVTDPQTGLRLLPLKEKKRER
H9FGT6.1.A--

Target KTSSKSSVRKRRVVIVDPETGKEMSVYEAYRKGLIDHQTYLELSEQECEWEEITISSSDGVVKSMIIDRRSGRQYDIDEA
H9FGT6.1.A--

Target IAKSLIDRSALDQYRAGTLSITEFADMLSGNAGGFRSRSSSVGSSSSYPISPAVSRTQLASWSDPTEETGPVAGILDTET
H9FGT6.1.A--

Target LEKVSITEAMHRNLVDNITGQRLLEAQACTGGIIDPNTGERFPVTDAVNKGLVDKIMVDRINLAQKAFCGFEDPRTKTKM
H9FGT6.1.A--

Target SAAQALKKGWLYYEAGQRFLEVQYLTGGLIEPDTPGRVPLDEALQRGTVDARTAQKLRDVSAYSKYLTCPKTKLKISYKD

```
H9FGT6.1.A--------------------------------------------------------------------------------

Target     ALDRSMVEEGTGLRLLEAAAQSSKGYYSPYSVSGSGSTTGSRSGSRTGSRAGSRRGSFDATGSGFSMTFSSSSYSSSGYG
H9FGT6.1.A--------------------------------------------------------------------------------

Target     RRYASGPTSSLGGPESAAA
H9FGT6.1.A-------------------
```

Model #02	File	Built with	Oligo-State	Ligands	GMQE	QMEANDisCo Global
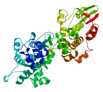	PDB	ProMod3 3.3.0	monomer	None	0.15	0.76 ± 0.05

Template	Seq Identity	Oligo-state	QSQE	Found by	Method	Resolution	Seq Similarity	Range	Coverage	Description
5dzz.1.A	51.24	monomer	0.00	HHblits	X-ray	2.60Å	0.45	1829 - 2314	0.18	Desmoplakin

The template contained no ligands.

```
Target     RALAEEAKRQRQLAEEDAARQRAEAERVLSEKLAAISEATRLKTEAEIALKEKEAENERLRRLAEDEAFQRRRLEEQAAQ
5dzz.1.A   --------------------------------------------------------------------------------

Target     HKADIEERLAQLRKASESELERQKGLVEDTLRQRRQVEEEILALKASFEKAAAGKAELELELGRIRGNAEDTLRSKEQAE
5dzz.1.A   --------------------------------------------------------------------------------

Target     QEAARQRQLAAEEERRRREAEERVQKSLAAEEEAARQRKAALEEVERLKAKVEEARRLRERAEHESVRQLQLAQEAAQKR
5dzz.1.A   --------------------------------------------------------------------------------

Target     LQAEEKAHAFAVQQKEQELQQTLQQEQSVLEKLRSEAEAARRAAEEAEEARERAEREAAQSRQRVEEAERLKQAAEEQAQ
5dzz.1.A   --------------------------------------------------------------------------------

Target     AQAQAQAAAEKLRKEAEQEAARRAQAEQAALRQKQAADAEMEKHKKFAEQTLRQKAQVEQELTALRLKLEETDHQKSILD
5dzz.1.A   --------------------------------------------------------------------------------

Target     QELQRLKAEVTEAARQRSQVEEELFSVRVQMEELGKLKARIEAENRALILRDKDNTQRLLQEEAEKMKQVAEEAARLSVA
5dzz.1.A   --------------------------------------------------------------------------------

Target     AQEAARLRQLAEEDLAQQRALAEKMLKEKMQAVQEATRLKAEAELLQQQKELAQEQARRLQEDKEQMAQQLAQETQGFQR
5dzz.1.A   --------------------------------------------------------------------------------

Target     TLETERQRQLEMSAEAERLRLRVAEMSRAQARAEEDAQRFRKQAEEIGAKLHRTELATQEKVTLVQTLETQRQQSDRDAD
5dzz.1.A   --------------------------------------------------------------------------------

Target     RLREAIAELEREKDKLKKEAELLQLKSEEMQTVQQEQLLQETQALQQSFLSEKDSLLQRERFIEEEKAKLERLFQDEVAK
5dzz.1.A   --------------------------------------------------------------------------------

Target     AQKLREEQQRQQQQMQQEKQQLLASMEEARRRQHEAEEGVRRKQEELQLLEQQRQQQEQLLAEENRRLRERLEHLEEEHR
5dzz.1.A   --------------------------------------------------------------------------------

Target     AALAHSEEITAAQAAATRALPNGQDAADGPAAEPEHAFEGLRQKVPAQQLQEAGILSTEELQRLAQGHTTVAELTQREDV
5dzz.1.A   --------------------------------------------------------------------------------

Target     RRYLQGHSSIAGLLLKPANEKLTIYAALRRQLLSPGTALILLEAQAASGFLLDPVRNRRLTVTEAVKEGVVGPELHHKLL
5dzz.1.A   --------------------------------------------------------------------------------

Target     SAERAVTGYKDPYTGEKISLFQAMKKDLIVREHGIRLLEAQIATGGVIDPVHSHRVPVDVAYHRGYFDEEMNRVLQDPSD
5dzz.1.A   --------------------------------------------------------------------------------

Target     DTKGFFDPNTHENLTYLQLLERCVEDPETGLRLLPLTDQAAKGGELVYTDSEARDVFEKATVSAPFGKFQGKTVTIWELI
5dzz.1.A   --------------------------------------------------------------------------------

Target     NSEYFTAEQRRDLLRQFRTGKVTVEKIIKIVITVIEEHEQKGQLCFQGLRALVPAAELLESGIIDWDLFRQLQLGERSVQ
5dzz.1.A   --------------------------------------------------------------------------------

Target     EVAEVEGVRRALRGSGVIAGVWLEEARQKLSIYEALKKELLQPEAAVALLEAQAGTGHVIDPATSARLTVDEAVRAGLVG
```

```
5dzz.1.A   --------------------------------------------------------------------------------

Target     PELHEKLLSAEKAVTGYKDPYSGGQSVSLFQALKKGLIPREQGLRLLDAQLSTGGTVDPSKSHRLPLDVACARGYLDEETS
5dzz.1.A   --------------------------------------------------------------------------------

Target     TALSAPRDDAKTYYDPRTWELATYSQLQQQCRPDPLTGLSLLPLSEEAARARQQELYSEVQAREAFQKATVEVPVGSFQG
5dzz.1.A   --------------------------------------------------------------------------------

Target     RAVTIWELINSEYFTAEQRQELLRQFRTGKVTVEKIIKIVITIVEEVETTRRERLSFSGLRAPVPASELLAAGILSSSQF
5dzz.1.A   --------------------------------------------------------------------------------

Target     EQLKDGKTSVKDLSELSSLQTLLQGSGCLAGIYLEESKEKVTIYEAMRRGLLRPSTAILLLEAQAATGFLVDPVRNQRLY
5dzz.1.A   --------------------------------------------------------------------------------

Target     VHEAVKAGVVGPELHEKLLSAEKAVTGYKDPYSGSTISLFQAMKKGLVVREHGIRLLEAQIATGGIIDPVHSHRVPVDVA
5dzz.1.A   --------------------------------------------------------------------------------

Target     CQRGYFDEEMNRVLEDPSDDTKGFFDPNTHENLTYRQLLERCVEDPETGLRLLPLKGPEKAEVVETTRVYTEEETRRAFE
5dzz.1.A   --------------------------------------------------------------------------------

Target     ETQIDIPGGGSHGGSTMSLWEVMQSDLIPEEQRAQLMADFQAGRVTKERMIIIIIEIIEKTEIVRQQNLASYDYVRRRLT
5dzz.1.A   -------------------------------------------------------------------KLVFDGLRKKVT

Target     AEDLYEARVISRESYSLLREGTRSLREVLEAESAWRYLYGTGCVAGVYLPGSRQTLPIYQALKKGLLSAEVARLLLEAQA
5dzz.1.A   AMQLYECQLIDKTTLDKLLKGKKSVEEVAS--EIQPFLRGAGSIAGAS-ASPKEKYSLVEAKRKKLISPESTVMLLEAQA

Target     ATGFLLDPVKGERLTVDEAVRKGLVGPELHDRLLSAERAVTGYRDPYTEQTISLFQAMKKDLIPAEEALRLLDAQLATGG
5dzz.1.A   ATGGIIDPHRNEKLTVDSAIARDLIDFDDRQQIYAAEKAITGFDDPFSGKTVSVSEAIKKNLIDRETGMRLLEAQIASGG

Target     IVDPRLGFHLPLEVAYQRGYLNKDTHDQLSEPSE-VRSYVDPSTDERLSYTQLLRRCRRDETSGLFLLPLSEARKLTFRG
5dzz.1.A   VVDPVNSVFLPKDVALARGLIDRDLYRSLNDPRDSQKNFVDPVTKKKVSYVQLKERCRIEPHTGLLLLSVQKR-SMSFQG

Target     LRKQITVEELVRSHVMDEATAQRLQEGLTSIEEVSKNLQKFLEGTSSIAGVLVDATKERLSVYQAMKKGIIRPGTAFELL
5dzz.1.A   IRQPVTVTELVDSGILRPSTVNELESGQISYDEVGERIKDFLQGSSCIAGIYNETTKQKLGIYEAMKIGLVRPGTALELL

Target     EAQAATGYVIDPIKGLKLTVEEAVRMGIVGPEFKDKLLSAERAVTGYKDPYSGKLISLFQAMKKGLILKDHGIRLLEAQI
5dzz.1.A   EAQAATGFIVDPVSNLRLPVEEAYKRGLVGIEFKEKLLSAERAVTGYNDPETGNIISLFQAMNKELIEKGHGIRLLEAQI

Target     ATGGIIDPEESHRLPVDVAYQRGLFDEEMNEILTDPSDDTKGFFDPNTEENLTYLQLMERCVTDPQTGLRLLPLKEKKRE
5dzz.1.A   ATGGIIDPKESHRLPVDIAYKRGYFNEELSEILSDPSDDTKGFFDPNTEENLTYLQLKERCIKDEETGLCLLPLK-----

Target     RKTSSKSSVRKRRVVIVDPETGKEMSVYEAYRKGLIDHQTYLELSEQECEWEEITISSSDGVVKSMIIDRRSGRQYDIDE
5dzz.1.A   --------------------------------------------------------------------------------

Target     AIAKSLIDRSALDQYRAGTLSITEFADMLSGNAGGFRSRSSSVGSSSSYPISPAVSRTQLASWSDPTEETGPVAGILDTE
5dzz.1.A   --------------------------------------------------------------------------------

Target     TLEKVSITEAMHRNLVDNITGQRLLEAQACTGGIIDPNTGERFPVTDAVNKGLVDKIMVDRINLAQKAFCGFEDPRTKTK
5dzz.1.A   --------------------------------------------------------------------------------

Target     MSAAQALKKGWLYYEAGQRFLEVQYLTGGLIEPDTPGRVPLDEALQRGTVDARTAQKLRDVSAYSKYLTCPKTKLKISYK
5dzz.1.A   --------------------------------------------------------------------------------

Target     DALDRSMVEEGTGLRLLEAAAQSSKGYYSPYSVSGSGSTTGSRSGSRTGSRAGSRRGSFDATGSGFSMTFSSSSYSSSGY
5dzz.1.A   --------------------------------------------------------------------------------

Target     GRRYASGPTSSLGGPESAAA
5dzz.1.A   --------------------
```

Model #03	File	Built with	Oligo-State	Ligands	GMQE	QMEANDisCo Global
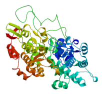	PDB	ProMod3 3.3.0	monomer	None	0.12	0.68 ± 0.05

Template	Seq Identity	Oligo-state	QSQE	Found by	Method	Resolution	Seq Similarity	Range	Coverage	Description
5dzz.1.A	54.32	monomer	0.00	HHblits	X-ray	2.60Å	0.45	1160 - 1736	0.18	Desmoplakin

The template contained no ligands.

```
Target    RALAEEAKRQRQLAEEDAARQRAEAERVLSEKLAAISEATRLKTEAEIALKEKEAENERLRRLAEDEAFQRRRLEEQAAQ
5dzz.1.A  --------------------------------------------------------------------------------

Target    HKADIEERLAQLRKASESELERQKGLVEDTLRQRRQVEEEILALKASFEKAAAGKAELELELGRIRGNAEDTLRSKEQAE
5dzz.1.A  --------------------------------------------------------------------------------

Target    QEAARQRQLAAEEERRRREAEERVQKSLAAEEEAARQRKAALEEVERLKAKVEEARRLRERAEHESVRQLQLAQEAAQKR
5dzz.1.A  --------------------------------------------------------------------------------

Target    LQAEEKAHAFAVQQKEQELQQTLQQEQSVLEKLRSEAEAARRAAEEAEEARERAEREAAQSRQRVEEAERLKQAAEEQAQ
5dzz.1.A  --------------------------------------------------------------------------------

Target    AQAQAQAAAEKLRKEAEQEAARRAQAEQAALRQKQAADAEMEKHKKFAEQTLRQKAQVEQELTALRLKLEETDHQKSILD
5dzz.1.A  --------------------------------------------------------------------------------

Target    QELQRLKAEVTEAARQRSQVEEELFSVRVQMEELGKLKARIEAENRALILRDKDNTQRLLQEEAEKMKQVAEEAARLSVA
5dzz.1.A  --------------------------------------------------------------------------------

Target    AQEAARLRQLAEEDLAQQRALAEKMLKEKMQAVQEATRLKAEAELLQQQKELAQEQARRLQEDKEQMAQQLAQETQGFQR
5dzz.1.A  --------------------------------------------------------------------------------

Target    TLETERQRQLEMSAEAERLRLRVAEMSRAQARAEEDAQRFRKQAEEIGAKLHRTELATQEKVTLVQTLETQRQQSDRDAD
5dzz.1.A  --------------------------------------------------------------------------------

Target    RLREAIAELEREKDKLKKEAELLQLKSEEMQTVQQEQLLQETQALQQSFLSEKDSLLQRERFIEEEKAKLERLFQDEVAK
5dzz.1.A  --------------------------------------------------------------------------------

Target    AQKLREEQQRQQQMQQEKQQLLASMEEARRRQHEAEEGVRRKQEELQLLEQQRQQQEQLLAEENRRLRERLEHLEEEHR
5dzz.1.A  --------------------------------------------------------------------------------

Target    AALAHSEEITAAQAAATRALPNGQDAADGPAAEPEHAFEGLRQKVPAQQLQEAGILSTEELQRLAQGHTTVAELTQREDV
5dzz.1.A  --------------------------------------------------------------------------------

Target    RRYLQGHSSIAGLLLKPANEKLTIYAALRRQLLSPGTALILLEAQAASGFLLDPVRNRRLTVTEAVKEGVVGPELHHKLL
5dzz.1.A  --------------------------------------------------------------------------------

Target    SAERAVTGYKDPYTGEKISLFQAMKKDLIVREHGIRLLEAQIATGGVIDPVHSHRVPVDVAYHRGYFDEEMNRVLQDPSD
5dzz.1.A  --------------------------------------------------------------------------------

Target    DTKGFFDPNTHENLTYLQLLERCVEDPETGLRLLPLTDQAAKGGELVYTDSEARDVFEKATVSAPFGKFQGKTVTIWELI
5dzz.1.A  --------------------------------------------------------------------------------

Target    NSEYFTAEQRRDLLRQFRTGKVTVEKIIKIVITVIEEHEQKGQLCFQGLRALVPAAELLESGIIDWDLFRQLQLGERSVQ
5dzz.1.A  ---------------------------------------DTSKLVFDGLRKKVTAMQLYECQLIDKTTLDKLLKGKKSVE

Target    EVAEVEGVRRALRGSGVIAGVWLEEARQKLSIYEALKKELLQPEAAVALLEAQAGTGHVIDPATSARLTVDEAVRAGLVG
5dzz.1.A  EVA--SEIQPFLRGAGSIAGAS-ASPKEKYSLVEAKRKKLISPESTVMLLEAQAATGGIIDPHRNEKLTVDSAIARDLID

Target    PELHEKLLSAEKAVTGYKDPYSGQSVSLFQALKKGLIPREQGLRLLDAQLSTGGTVDPSKSHRLPLDVACARGYLDEETS
5dzz.1.A  FDDRQQIYAAEKAITGFDDPFSGKTVSVSEAIKKNLIDRETGMRLLEAQIASGGVVDPVNSVFLPKDVALARGLIDRDLY

Target    TALSAPRDDAKTYYDPRTWELATYSQLQQQCRPDPLTGLSLLPLSEEAARARQQELYSEVQAREAFQKATVEVPVGSFQG
5dzz.1.A  RSLNDPRDSQKNFVDPVTKKKVSYVQLKERCRIEPHTGLLLLSVQKRS--------------------------------

Target    RAVTIWELINSEYFTAEQRQELLRQFRTGKVTVEKIIKIVITIVEEVETTRRERLSFSGLRAPVPASELLAAGILSSSQF
5dzz.1.A  ------------------------------------------------------MSFQGIRQPVTVTELVDSGILRPSTV

Target    EQLKDGKTSVKDLSELSSLQTLLQGSGCLAGIYLEESKEKVTIYEAMRRGLLRPSTAILLLEAQAATGFLVDPVRNQRLY
5dzz.1.A  NELESGQISYDEVGE--RIKDFLQGSSCIAGIYNETTKQKLGIYEAMKIGLVRPGTALELLEAQAATGFIVDPVSNLRLP

Target    VHEAVKAGVVGPELHEKLLSAEKAVTGYKDPYSGSTISLFQAMKKGLVVREHGIRLLEAQIATGGIIDPVHSHRVPVDVA
5dzz.1.A  VEEAYKRGLVGIEFKEKLLSAERAVTGYNDPETGNIISLFQAMNKELIEKGHGIRLLEAQIATGGIIDPKESHRLPVDIA

Target    CQRGYFDEEMNRVLEDPSDDTKGFFDPNTHENLTYRQLLERCVEDPETGLRLLPLKGPEKAEVVETTRVYTEEETRRAFE
5dzz.1.A  YKRGYFNEELSEILSDPSDDTKGFFDPNTEENLTYLQLKERCIKDEETGLCLLPLK------------------------
```

```
Target    ETQIDIPGGGSHGGSTMSLWEVMQSDLIPEEQRAQLMADFQAGRVTKERMIIIIIEIIEKTEIVRQQNLASYDYVRRRLT
5dzz.1.A  --------------------------------------------------------------------------------

Target    AEDLYEARVISRESYSLLREGTRSLREVLEAESAWRYLYGTGCVAGVYLPGSRQTLPIYQALKKGLLSAEVARLLLEAQA
5dzz.1.A  --------------------------------------------------------------------------------

Target    ATGFLLDPVKGERLTVDEAVRKGLVGPELHDRLLSAERAVTGYRDPYTEQTISLFQAMKKDLIPAEEALRLLDAQLATGG
5dzz.1.A  --------------------------------------------------------------------------------

Target    IVDPRLGFHLPLEVAYQRGYLNKDTHDQLSEPSEVRSYVDPSTDERLSYTQLLRRCRRDETSGLFLLPLSEARKLTFRGL
5dzz.1.A  --------------------------------------------------------------------------------

Target    RKQITVEELVRSHVMDEATAQRLQEGLTSIEEVSKNLQKFLEGTSSIAGVLVDATKERLSVYQAMKKGIIRPGTAFELLE
5dzz.1.A  --------------------------------------------------------------------------------

Target    AQAATGYVIDPIKGLKLTVEEAVRMGIVGPEFKDKLLSAERAVTGYKDPYSGKLISLFQAMKKGLILKDHGIRLLEAQIA
5dzz.1.A  --------------------------------------------------------------------------------

Target    TGGIIDPEESHRLPVDVAYQRGLFDEEMNEILTDPSDDTKGFFDPNTEENLTYLQLMERCVTDPQTGLRLLPLKEKKRER
5dzz.1.A  --------------------------------------------------------------------------------

Target    KTSSKSSVRKRRVVIVDPETGKEMSVYEAYRKGLIDHQTYLELSEQECEWEEITISSSDGVVKSMIIDRRSGRQYDIDEA
5dzz.1.A  --------------------------------------------------------------------------------

Target    IAKSLIDRSALDQYRAGTLSITEFADMLSGNAGGFRSRSSSVGSSSSYPISPAVSRTQLASWSDPTEETGPVAGILDTET
5dzz.1.A  --------------------------------------------------------------------------------

Target    LEKVSITEAMHRNLVDNITGQRLLEAQACTGGIIDPNTGERFPVTDAVNKGLVDKIMVDRINLAQKAFCGFEDPRTKTKM
5dzz.1.A  --------------------------------------------------------------------------------

Target    SAAQALKKGWLYYEAGQRFLEVQYLTGGLIEPDTPGRVPLDEALQRGTVDARTAQKLRDVSAYSKYLTCPKTKLKISYKD
5dzz.1.A  --------------------------------------------------------------------------------

Target    ALDRSMVEEGTGLRLLEAAAQSSKGYYSPYSVSGSGSTTGSRSGSRTGSRAGSRRGSFDATGSGFSMTFSSSSYSSSGYG
5dzz.1.A  --------------------------------------------------------------------------------

Target    RRYASGPTSSLGGPESAAA
5dzz.1.A  -------------------
```

Model #04	File	Built with	Oligo-State	Ligands	GMQE	QMEANDisCo Global
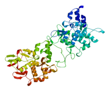	PDB	ProMod3 3.3.0	monomer	None	0.09	0.66 ± 0.05

Template	Seq Identity	Oligo-state	QSQE	Found by	Method	Resolution	Seq Similarity	Range	Coverage	Description
5dzz.1.A	47.22	monomer	0.00	HHblits	X-ray	2.60Å	0.42	833 - 1405	0.18	Desmoplakin

The template contained no ligands.

```
Target    RALAEEAKRQRQLAEEDAARQRAEAERVLSEKLAAISEATRLKTEAEIALKEKEAENERLRRLAEDEAFQRRRLEEQAAQ
5dzz.1.A  --------------------------------------------------------------------------------

Target    HKADIEERLAQLRKASESELERQKGLVEDTLRQRRQVEEEILALKASFEKAAAGKAELELELGRIRGNAEDTLRSKEQAE
5dzz.1.A  --------------------------------------------------------------------------------

Target    QEAARQRQLAAEEERRRREAEERVQKSLAAEEEAARQRKAALEEVERLKAKVEEARRLRERAEHESVRQLQLAQEAAQKR
5dzz.1.A  --------------------------------------------------------------------------------

Target    LQAEEKAHAFAVQQKEQELQQTLQQEQSVLEKLRSEAEAARRAAEEAEEARERAEREAAQSRQRVEEAERLKQAAEEQAQ
5dzz.1.A  --------------------------------------------------------------------------------

Target    AQAQAQAAAEKLRKEAEQEAARRAQAEQAALRQKQAADAEMEKHKKFAEQTLRQKAQVEQELTALRLKLEETDHQKSILD
5dzz.1.A  --------------------------------------------------------------------------------
```

```
Target     QELQRLKAEVTEAARQRSQVEEELFSVRVQMEELGKLKARIEAENRALILRDKDNTQRLLQEEAEKMKQVAEEAARLSVA
5dzz.1.A   --------------------------------------------------------------------------------

Target     AQEAARLRQLAEEDLAQQRALAEKMLKEKMQAVQEATRLKAEAELLQQQKELAQEQARRLQEDKEQMAQQLAQETQGFQR
5dzz.1.A   --------------------------------------------------------------------------------

Target     TLETERQRQLEMSAEAERLRLRVAEMSRAQARAEEDAQRFRKQAEEIGAKLHRTELATQEKVTLVQTLETQRQQSDRDAD
5dzz.1.A   --------------------------------------------------------------------------------

Target     RLREAIAELEREKDKLKKEAELLQLKSEEMQTVQQEQLLQETQALQQSFLSEKDSLLQRERFIEEEKAKLERLFQDEVAK
5dzz.1.A   --------------------------------------------------------------------------------

Target     AQKLREEQQRQQQQMQQEKQQLLASMEEARRRQHEAEEGVRRKQEELQLLEQQRQQQEQLLAEENRRLRERLEHLEEEHR
5dzz.1.A   --------------------------------------------------------------------------------

Target     AALAHSEEITAAQAAATRALPNGQDAADGPAAEPEHAFEGLRQKVPAQQLQEAGILSTEELQRLAQGHTTVAELTQREDV
5dzz.1.A   --------------------------------TSKLVFDGLRKKVTAMQLYECQLIDKTTLDKLLKGKKSVEEVA--SEI

Target     RRYLQGHSSIAGLLLKPANEKLTIYAALRRQLLSPGTALILLEAQAASGFLLDPVRNRRLTVTEAVKEGVVGPELHHKLL
5dzz.1.A   QPFLRGAGSIAGAS-ASPKEKYSLVEAKRKKLISPESTVMLLEAQAATGGIIDPHRNEKLTVDSAIARDLIDFDDRQQIY

Target     SAERAVTGYKDPYTGEKISLFQAMKKDLIVREHGIRLLEAQIATGGVIDPVHSHRVPVDVAYHRGYFDEEMNRVLQDPSD
5dzz.1.A   AAEKAITGFDDPFSGKTVSVSEAIKKNLIDRETGMRLLEAQIASGGVVDPVNSVFLPKDVALARGLIDRDLYRSLNDPRD

Target     DTKGFFDPNTHENLTYLQLLERCVEDPETGLRLLPLTDQAAKGGELVYTDSEARDVFEKATVSAPFGKFQGKTVTIWELI
5dzz.1.A   SQKNFVDPVTKKKVSYVQLKERCRIEPHTGLLLLSVQKRS----------------------------------------

Target     NSEYFTAEQRRDLLRQFRTGKVTVEKIIKIVITVIEEHEQKGQLCFQGLRALVPAAELLESGIIDWDLFRQLQLGERSVQ
5dzz.1.A   -------------------------------------------MSFQGIRQPVTVTELVDSGILRPSTVNELESGQISYD

Target     EVAEVEGVRRALRGSGVIAGVWLEEARQKLSIYEALKKELLQPEAAVALLEAQAGTGHVIDPATSARLTVDEAVRAGLVG
5dzz.1.A   EVGE--RIKDFLQGSSCIAGIYNETTKQKLGIYEAMKIGLVRPGTALELLEAQAATGFIVDPVSNLRLPVEEAYKRGLVG

Target     PELHEKLLSAEKAVTGYKDPYSGQSVSLFQALKKGLIPREQGLRLLDAQLSTGGTVDPSKSHRLPLDVACARGYLDEETS
5dzz.1.A   IEFKEKLLSAERAVTGYNDPETGNIISLFQAMNKELIEKGHGIRLLEAQIATGGIIDPKESHRLPVDIAYKRGYFNEELS

Target     TALSAPRDDAKTYYDPRTWELATYSQLQQQCRPDPLTGLSLLPLSEEAARARQQELYSEVQAREAFQKATVEVPVGSFQG
5dzz.1.A   EILSDPSDDTKGFFDPNTEENLTYLQLKERCIKDEETGLCLLPLK-----------------------------------

Target     RAVTIWELINSEYFTAEQRQELLRQFRTGKVTVEKIIKIVITIVEEVETTRRERLSFSGLRAPVPASELLAAGILSSSQF
5dzz.1.A   --------------------------------------------------------------------------------

Target     EQLKDGKTSVKDLSELSSLQTLLQGSGCLAGIYLEESKEKVTIYEAMRRGLLRPSTAILLLEAQAATGFLVDPVRNQRLY
5dzz.1.A   --------------------------------------------------------------------------------

Target     VHEAVKAGVVGPELHEKLLSAEKAVTGYKDPYSGSTISLFQAMKKGLVVREHGIRLLEAQIATGGIIDPVHSHRVPVDVA
5dzz.1.A   --------------------------------------------------------------------------------

Target     CQRGYFDEEMNRVLEDPSDDTKGFFDPNTHENLTYRQLLERCVEDPETGLRLLPLKGPEKAEVVETTRVYTEEETRRAFE
5dzz.1.A   --------------------------------------------------------------------------------

Target     ETQIDIPGGGSHGGSTMSLWEVMQSDLIPEEQRAQLMADFQAGRVTKERMIIIIIEIIEKTEIVRQQNLASYDYVRRRLT
5dzz.1.A   --------------------------------------------------------------------------------

Target     AEDLYEARVISRESYSLLREGTRSLREVLEAESAWRYLYGTGCVAGVYLPGSRQTLPIYQALKKGLLSAEVARLLLEAQA
5dzz.1.A   --------------------------------------------------------------------------------

Target     ATGFLLDPVKGERLTVDEAVRKGLVGPELHDRLLSAERAVTGYRDPYTEQTISLFQAMKKDLIPAEEALRLLDAQLATGG
5dzz.1.A   --------------------------------------------------------------------------------

Target     IVDPRLGFHLPLEVAYQRGYLNKDTHDQLSEPSEVRSYVDPSTDERLSYTQLLRRCRRDETSGLFLLPLSEARKLTFRGL
5dzz.1.A   --------------------------------------------------------------------------------

Target     RKQITVEELVRSHVMDEATAQRLQEGLTSIEEVSKNLQKFLEGTSSIAGVLVDATKERLSVYQAMKKGIIRPGTAFELLE
5dzz.1.A   --------------------------------------------------------------------------------

Target     AQAATGYVIDPIKGLKLTVEEAVRMGIVGPEFKDKLLSAERAVTGYKDPYSGKLISLFQAMKKGLILKDHGIRLLEAQIA
5dzz.1.A   --------------------------------------------------------------------------------

Target     TGGIIDPEESHRLPVDVAYQRGLFDEEMNEILTDPSDDTKGFFDPNTEENLTYLQLMERCVTDPQTGLRLLPLKEKKRER
5dzz.1.A   --------------------------------------------------------------------------------

Target     KTSSKSSVRKRRVVIVDPETGKEMSVYEAYRKGLIDHQTYLELSEQECEWEEITISSSDGVVKSMIIDRRSGRQYDIDEA
5dzz.1.A   --------------------------------------------------------------------------------
```

```
Target    IAKSLIDRSALDQYRAGTLSITEFADMLSGNAGGFRSRSSSVGSSSSYPISPAVSRTQLASWSDPTEETGPVAGILDTET
5dzz.1.A  --------------------------------------------------------------------------------

Target    LEKVSITEAMHRNLVDNITGQRLLEAQACTGGIIDPNTGERFPVTDAVNKGLVDKIMVDRINLAQKAFCGFEDPRTKTKM
5dzz.1.A  --------------------------------------------------------------------------------

Target    SAAQALKKGWLYYEAGQRFLEVQYLTGGLIEPDTPGRVPLDEALQRGTVDARTAQKLRDVSAYSKYLTCPKTKLKISYKD
5dzz.1.A  --------------------------------------------------------------------------------

Target    ALDRSMVEEGTGLRLLEAAAQSSKGYYSPYSVSGSGSTTGSRSGSRTGSRAGSRRGSFDATGSGFSMTFSSSSYSSSGYG
5dzz.1.A  --------------------------------------------------------------------------------

Target    RRYASGPTSSLGGPESAAA
5dzz.1.A  -------------------
```

Model #06	File	Built with	Oligo-State	Ligands	GMQE	QMEANDisCo Global
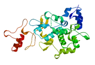	PDB	ProMod3 3.3.0	monomer	None	0.04	0.69 ± 0.05

Template	Seq Identity	Oligo-state	QSQE	Found by	Method	Resolution	Seq Similarity	Range	Coverage	Description
5dzz.1.A	45.93	monomer	0.00	BLAST	X-ray	2.60Å	0.41	1163 - 1462	0.10	Desmoplakin

The template contained no ligands.

```
Target    RALAEEAKRQRQLAEEDAARQRAEAERVLSEKLAAISEATRLKTEAEIALKEKEAENERLRRLAEDEAFQRRRLEEQAAQ
5dzz.1.A  --------------------------------------------------------------------------------

Target    HKADIEERLAQLRKASESELERQKGLVEDTLRQRRQVEEEILALKASFEKAAAGKAELELELGRIRGNAEDTLRSKEQAE
5dzz.1.A  --------------------------------------------------------------------------------

Target    QEAARQRQLAAEEERRRREAEERVQKSLAAEEEAARQRKAALEEVERLKAKVEEARRLRERAEHESVRQLQLAQEAAQKR
5dzz.1.A  --------------------------------------------------------------------------------

Target    LQAEEKAHAFAVQQKEQELQQTLQQEQSVLEKLRSEAEAARRAAEEAEEARERAEREAAQSRQRVEEAERLKQAAEEQAQ
5dzz.1.A  --------------------------------------------------------------------------------

Target    AQAQAQAAAEKLRKEAEQEAARRAQAEQAALRQKQAADAEMEKHKKFAEQTLRQKAQVEQELTALRLKLEETDHQKSILD
5dzz.1.A  --------------------------------------------------------------------------------

Target    QELQRLKAEVTEAARQRSQVEEELFSVRVQMEELGKLKARIEAENRALILRDKDNTQRLLQEEAEKMKQVAEEAARLSVA
5dzz.1.A  --------------------------------------------------------------------------------

Target    AQEAARLRQLAEEDLAQQRALAEKMLKEKMQAVQEATRLKAEAELLQQQKELAQEQARRLQEDKEQMAQQLAQETQGFQR
5dzz.1.A  --------------------------------------------------------------------------------

Target    TLETERQRQLEMSAEAERLRLRVAEMSRAQARAEEDAQRFRKQAEEIGAKLHRTELATQEKVTLVQTLETQRQQSDRDAD
5dzz.1.A  --------------------------------------------------------------------------------

Target    RLREAIAELEREKDKLKKEAELLQLKSEEMQTVQQEQLLQETQALQQSFLSEKDSLLQRERFIEEEKAKLERLFQDEVAK
5dzz.1.A  --------------------------------------------------------------------------------

Target    AQKLREEQQRQQQQMQQEKQQLLASMEEARRRQHEAEEGVRRKQEELQLLEQQRQQQEQLLAEENRRLRERLEHLEEEHR
5dzz.1.A  --------------------------------------------------------------------------------

Target    AALAHSEEITAAQAAATRALPNGQDAADGPAAEPEHAFEGLRQKVPAQQLQEAGILSTEELQRLAQGHTTVAELTQREDV
5dzz.1.A  --------------------------------------------------------------------------------

Target    RRYLQGHSSIAGLLLKPANEKLTIYAALRRQLLSPGTALILLEAQAASGFLLDPVRNRRLTVTEAVKEGVVGPELHHKLL
5dzz.1.A  --------------------------------------------------------------------------------

Target    SAERAVTGYKDPYTGEKISLFQAMKKDLIVREHGIRLLEAQIATGGVIDPVHSHRVPVDVAYHRGYFDEEMNRVLQDPSD
5dzz.1.A  --------------------------------------------------------------------------------
```

Target   DTKGFFDPNTHENLTYLQLLERCVEDPETGLRLLPLTDQAAKGGELVYTDSEARDVFEKATVSAPFGKFQGKTVTIWELI
5dzz.1.A   --

Target   NSEYFTAEQRRDLLRQFRTGKVTVEKIIKIVITVIEEHEQKGQLCFQGLRALVPAAELLESGIIDWDLFRQLQLGERSVQ
5dzz.1.A   --KLVFDGLRKKVTAMQLYECQLIDKTTLDKLLKGKKSVE

Target   EVAEVEGVRRALRGSGVIAGVWLEEARQKLSIYEALKKELLQPEAAVALLEAQAGTGHVIDPATSARLTVDEAVRAGLVG
5dzz.1.A   EVAS--EIQPFLRGAGSIAGAS-ASPKEKYSLVEAKRKKLISPESTVMLLEAQAATGGIIDPHRNEKLTVDSAIARDLID

Target   PELHEKLLSAEKAVTGYKDPYSGQSVSLFQALKKGLIPREQGLRLLDAQLSTGGTVDPSKSHRLPLDVACARGYLDEETS
5dzz.1.A   FDDRQQIYAAEKAITGFDDPFSGKTVSVSEAIKKNLIDRETGMRLLEAQIASGGVVDPVNSVFLPKDVALARGLIDRDLY

Target   TALSAPRDDAKTYYDPRTWELATYSQLQQQCRPDPLTGLSLLPLSEEAARARQQELYSEVQAREAFQKATVEVPVGSFQG
5dzz.1.A   RSLNDPRDSQKNFVDPVTKKKVSYVQLKERCRIEPHTGLLLL----------------SVQKRSM-----------SFQG

Target   --RAVTIWELINSEYFTAEQRQELLRQFRTGKVTVEKIIKIVITIVEEVETTRRERLSFSGLRAPVPASELLAAGILSSS
5dzz.1.A   IRQPVTVTELVDSGILRPSTVNEL--

Target   QFEQLKDGKTSVKDLSELSSLQTLLQGSGCLAGIYLEESKEKVTIYEAMRRGLLRPSTAILLLEAQAATGFLVDPVRNQR
5dzz.1.A   --

Target   LYVHEAVKAGVVGPELHEKLLSAEKAVTGYKDPYSGSTISLFQAMKKGLVVREHGIRLLEAQIATGGIIDPVHSHRVPVD
5dzz.1.A   --

Target   VACQRGYFDEEMNRVLEDPSDDTKGFFDPNTHENLTYRQLLERCVEDPETGLRLLPLKGPEKAEVVETTRVYTEEETRRA
5dzz.1.A   --

Target   FEETQIDIPGGGSHGGSTMSLWEVMQSDLIPEEQRAQLMADFQAGRVTKERMIIIIIEIIEKTEIVRQQNLASYDYVRRR
5dzz.1.A   --

Target   LTAEDLYEARVISRESYSLLREGTRSLREVLEAESAWRYLYGTGCVAGVYLPGSRQTLPIYQALKKGLLSAEVARLLLEA
5dzz.1.A   --

Target   QAATGFLLDPVKGERLTVDEAVRKGLVGPELHDRLLSAERAVTGYRDPYTEQTISLFQAMKKDLIPAEEALRLLDAQLAT
5dzz.1.A   --

Target   GGIVDPRLGFHLPLEVAYQRGYLNKDTHDQLSEPSEVRSYVDPSTDERLSYTQLLRRCRRDETSGLFLLPLSEARKLTFR
5dzz.1.A   --

Target   GLRKQITVEELVRSHVMDEATAQRLQEGLTSIEEVSKNLQKFLEGTSSIAGVLVDATKERLSVYQAMKKGIIRPGTAFEL
5dzz.1.A   --

Target   LEAQAATGYVIDPIKGLKLTVEEAVRMGIVGPEFKDKLLSAERAVTGYKDPYSGKLISLFQAMKKGLILKDHGIRLLEAQ
5dzz.1.A   --

Target   IATGGIIDPEESHRLPVDVAYQRGLFDEEMNEILTDPSDDTKGFFDPNTEENLTYLQLMERCVTDPQTGLRLLPLKEKKR
5dzz.1.A   --

Target   ERKTSSKSSVRKRRVVIVDPETGKEMSVYEAYRKGLIDHQTYLELSEQECEWEEITISSSDGVVKSMIIDRRSGRQYDID
5dzz.1.A   --

Target   EAIAKSLIDRSALDQYRAGTLSITEFADMLSGNAGGFRSRSSSVGSSSSYPISPAVSRTQLASWSDPTEETGPVAGILDT
5dzz.1.A   --

Target   ETLEKVSITEAMHRNLVDNITGQRLLEAQACTGGIIDPNTGERFPVTDAVNKGLVDKIMVDRINLAQKAFCGFEDPRTKT
5dzz.1.A   --

Target   KMSAAQALKKGWLYYEAGQRFLEVQYLTGGLIEPDTPGRVPLDEALQRGTVDARTAQKLRDVSAYSKYLTCPKTKLKISY
5dzz.1.A   --

Target   KDALDRSMVEEGTGLRLLEAAAQSSKGYYSPYSVSGSGSTTGSRSGSRTGSRAGSRRGSFDATGSGFSMTFSSSSYSSSG
5dzz.1.A   --

Target   YGRRYASGPTSSLGGPESAAA
5dzz.1.A   ---------------------

Model #05	File	Built with	Oligo-State	Ligands	GMQE	QMEANDisCo Global
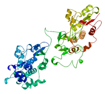	PDB	ProMod3 3.3.0	monomer	None	0.03	0.59 ± 0.05

Template	Seq Identity	Oligo-state	QSQE	Found by	Method	Resolution	Seq Similarity	Range	Coverage	Description
5dzz.1.A	38.27	monomer	0.00	HHblits	X-ray	2.60Å	0.38	2071 - 2659	0.18	Desmoplakin

The template contained no ligands.

```
Target     RALAEEAKRQRQLAEEDAARQRAEAERVLSEKLAAISEATRLKTEAEIALKEKEAENERLRRLAEDEAFQRRRLEEQAAQ
5dzz.1.A   --------------------------------------------------------------------------------

Target     HKADIEERLAQLRKASESELERQKGLVEDTLRQRRQVEEEILALKASFEKAAAGKAELELELGRIRGNAEDTLRSKEQAE
5dzz.1.A   --------------------------------------------------------------------------------

Target     QEAARQRQLAAEEERRRREAEERVQKSLAAEEEAARQRKAALEEVERLKAKVEEARRLRERAEHESVRQLQLAQEAAQKR
5dzz.1.A   --------------------------------------------------------------------------------

Target     LQAEEKAHAFAVQQKEQELQQTLQQEQSVLEKLRSEAEAARRAAEEAEEARERAEREAAQSRQRVEEAERLKQAAEEQAQ
5dzz.1.A   --------------------------------------------------------------------------------

Target     AQAQAQAAAEKLRKEAEQEAARRAQAEQAALRQKQAADAEMEKHKKFAEQTLRQKAQVEQELTALRLKLEETDHQKSILD
5dzz.1.A   --------------------------------------------------------------------------------

Target     QELQRLKAEVTEAARQRSQVEEELFSVRVQMEELGKLKARIEAENRALILRDKDNTQRLLQEEAEKMKQVAEEAARLSVA
5dzz.1.A   --------------------------------------------------------------------------------

Target     AQEAARLRQLAEEDLAQQRALAEKMLKEKMQAVQEATRLKAEAELLQQQKELAQEQARRLQEDKEQMAQQLAQETQGFQR
5dzz.1.A   --------------------------------------------------------------------------------

Target     TLETERQRQLEMSAEAERLRLRVAEMSRAQARAEEDAQRFRKQAEEIGAKLHRTELATQEKVTLVQTLETQRQQSDRDAD
5dzz.1.A   --------------------------------------------------------------------------------

Target     RLREAIAELEREKDKLKKEAELLQLKSEEMQTVQQEQLLQETQALQQSFLSEKDSLLQRERFIEEEKAKLERLFQDEVAK
5dzz.1.A   --------------------------------------------------------------------------------

Target     AQKLREEQQRQQQMQQEKQQLLASMEEARRRQHEAEEGVRRKQEELQLLEQQRQQQEQLLAEENRRLRERLEHLEEEHR
5dzz.1.A   --------------------------------------------------------------------------------

Target     AALAHSEEITAAQAAATRALPNGQDAADGPAAEPEHAFEGLRQKVPAQQLQEAGILSTEELQRLAQGHTTVAELTQREDV
5dzz.1.A   --------------------------------------------------------------------------------

Target     RRYLQGHSSIAGLLLKPANEKLTIYAALRRQLLSPGTALILLEAQAASGFLLDPVRNRRLTVTEAVKEGVVGPELHHKLL
5dzz.1.A   --------------------------------------------------------------------------------

Target     SAERAVTGYKDPYTGEKISLFQAMKKDLIVREHGIRLLEAQIATGGVIDPVHSHRVPVDVAYHRGYFDEEMNRVLQDPSD
5dzz.1.A   --------------------------------------------------------------------------------

Target     DTKGFFDPNTHENLTYLQLLERCVEDPETGLRLLPLTDQAAKGGELVYTDSEARDVFEKATVSAPFGKFQGKTVTIWELI
5dzz.1.A   --------------------------------------------------------------------------------

Target     NSEYFTAEQRRDLLRQFRTGKVTVEKIIKIVITVIEEHEQKGQLCFQGLRALVPAAELLESGIIDWDLFRQLQLGERSVQ
5dzz.1.A   --------------------------------------------------------------------------------

Target     EVAEVEGVRRALRGSGVIAGVWLEEARQKLSIYEALKKELLQPEAAVALLEAQAGTGHVIDPATSARLTVDEAVRAGLVG
5dzz.1.A   --------------------------------------------------------------------------------

Target     PELHEKLLSAEKAVTGYKDPYSGQSVSLFQALKKGLIPREQGLRLLDAQLSTGGTVDPSKSHRLPLDVACARGYLDEETS
5dzz.1.A   --------------------------------------------------------------------------------

Target     TALSAPRDDAKTYYDPRTWELATYSQLQQQCRPDPLTGLSLLPLSEEAARARQQELYSEVQAREAFQKATVEVPVGSFQG
5dzz.1.A   --------------------------------------------------------------------------------

Target     RAVTIWELINSEYFTAEQRQELLRQFRTGKVTVEKIIKIVITIVEEVETTRRERLSFSGLRAPVPASELLAAGILSSSQF
5dzz.1.A   --------------------------------------------------------------------------------
```

```
Target     EQLKDGKTSVKDLSELSSLQTLLQGSGCLAGIYLEESKEKVTIYEAMRRGLLRPSTAILLLEAQAATGFLVDPVRNQRLY
5dzz.1.A   --------------------------------------------------------------------------------

Target     VHEAVKAGVVGPELHEKLLSAEKAVTGYKDPYSGSTISLFQAMKKGLVVREHGIRLLEAQIATGGIIDPVHSHRVPVDVA
5dzz.1.A   --------------------------------------------------------------------------------

Target     CQRGYFDEEMNRVLEDPSDDTKGFFDPNTHENLTYRQLLERCVEDPETGLRLLPLKGPEKAEVVETTRVYTEEETRRAFE
5dzz.1.A   --------------------------------------------------------------------------------

Target     ETQIDIPGGGSHGGSTMSLWEVMQSDLIPEEQRAQLMADFQAGRVTKERMIIIIIEIIEKTEIVRQQNLASYDYVRRRLT
5dzz.1.A   --------------------------------------------------------------------------------

Target     AEDLYEARVISRESYSLLREGTRSLREVLEAESAWRYLYGTGCVAGVYLPGSRQTLPIYQALKKGLLSAEVARLLLEAQA
5dzz.1.A   --------------------------------------------------------------------------------

Target     ATGFLLDPVKGERLTVDEAVRKGLVGPELHDRLLSAERAVTGYRDPYTEQTISLFQAMKKDLIPAEEALRLLDAQLATGG
5dzz.1.A   --------------------------------------------------------------------------------

Target     IVDPRLGFHLPLEVAYQRGYLNKDTHDQLSEPSEVRSYVDPSTDERLSYTQLLRRCRRDETSGLFLLPLSEARKLTFRGL
5dzz.1.A   ----------------------------------------------------------------------DTSKLVFDGL

Target     RKQITVEELVRSHVMDEATAQRLQEGLTSIEEVSKNLQKFLEGTSSIAGVLVDATKERLSVYQAMKKGIIRPGTAFELLE
5dzz.1.A   RKKVTAMQLYECQLIDKTTLDKLLKGKKSVEEVASEIQPFLRGAGSIAGAS-ASPKEKYSLVEAKRKKLISPESTVMLLE

Target     AQAATGYVIDPIKGLKLTVEEAVRMGIVGPEFKDKLLSAERAVTGYKDPYSGKLISLFQAMKKGLILKDHGIRLLEAQIA
5dzz.1.A   AQAATGGIIDPHRNEKLTVDSAIARDLIDFDDRQQIYAAEKAITGFDDPFSGKTVSVSEAIKKNLIDRETGMRLLEAQIA

Target     TGGIIDPEESHRLPVDVAYQRGLFDEEMNEILTDPSDDTKGFFDPNTEENLTYLQLMERCVTDPQTGLRLLPLKEKKRER
5dzz.1.A   SGGVVDPVNSVFLPKDVALARGLIDRDLYRSLNDPRDSQKNFVDPVTKKKVSYVQLKERCRIEPHTGLLLLSVQKRSM--

Target     KTSSKSSVRKRRVVIVDPETGKEMSVYEAYRKGLIDHQTYLELSEQECEWEEITISSSDGVVKSMIIDRRSGRQYDIDEA
5dzz.1.A   ---------------SFQGIRQPVTVTELVDSGILRPS------------------------------------------

Target     IAKSLIDRSALDQYRAGTLSITEFADMLSGNAGGFRSRSSSVGSSSSYPISPAVSRTQLASWSDPTEETGPVAGILDTET
5dzz.1.A   ---------TVNELESGQISYDEVGE----------------------------------RIKDFLQGSSCIAGIYNETT

Target     LEKVSITEAMHRNLVDNITGQRLLEAQACTGGIIDPNTGERFPVTDAVNKGLVDKIMVDRINLAQKAFCGFEDPRTKTKM
5dzz.1.A   KQKLGIYEAMKIGLVRPGTALELLEAQAATGFIVDPVSNLRLPVEEAYKRGLVGIEFKEKLLSAERAVTGYNDPETGNII

Target     SAAQALKKGWLYYEAGQRFLEVQYLTGGLIEPDTPGRVPLDEALQRGTVDARTAQKLRDVSAYSKYLTCPKTKLKISYKD
5dzz.1.A   SLFQAMNKELIEKGHGIRLLEAQIATGGIIDPKESHRLPVDIAYKRGYFNEELSEILSDPSDDTKGFFDPNTEENLTYLQ

Target     ALDRSMVEEGTGLRLLEAAAQSSKGYYSPYSVSGSGSTTGSRSGSRTGSRAGSRRGSFDATGSGFSMTFSSSSYSSSGYG
5dzz.1.A   LKERCIKDEETGLCLLPLK-------------------------------------------------------------

Target     RRYASGPTSSLGGPESAAA
5dzz.1.A   -------------------
```

Model #08	File	Built with	Oligo-State	Ligands	GMQE	QMEANDisCo Global
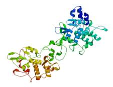	PDB	ProMod3 3.3.0	monomer	None	0.01	0.56 ± 0.05

Template	Seq Identity	Oligo-state	QSQE	Found by	Method	Resolution	Seq Similarity	Range	Coverage	Description
5dzz.1.A	37.12	monomer	0.00	BLAST	X-ray	2.60Å	0.38	837 - 1326	0.17	Desmoplakin

The template contained no ligands.

```
Target     RALAEEAKRQRQLAEEDAARQRAEAERVLSEKLAAISEATRLKTEAEIALKEKEAENERLRRLAEDEAFQRRRLEEQAAQ
5dzz.1.A   --------------------------------------------------------------------------------

Target     HKADIEERLAQLRKASESELERQKGLVEDTLRQRRQVEEEILALKASFEKAAAGKAELELELGRIRGNAEDTLRSKEQAE
5dzz.1.A   --------------------------------------------------------------------------------
```

Target   QEAARQRQLAAEEERRRREAEERVQKSLAAEEEAARQRKAALEEVERLKAKVEEARRLRERAEHESVRQLQLAQEAAQKR
5dzz.1.A   --

Target   LQAEEKAHAFAVQQKEQELQQTLQQEQSVLEKLRSEAEAARRAAEEAEEARERAEREAAQSRQRVEEAERLKQAAEEQAQ
5dzz.1.A   --

Target   AQAQAQAAAEKLRKEAEQEAARRAQAEQAALRQKQAADAEMEKHKKFAEQTLRQKAQVEQELTALRLKLEETDHQKSILD
5dzz.1.A   --

Target   QELQRLKAEVTEAARQRSQVEEELFSVRVQMEELGKLKARIEAENRALILRDKDNTQRLLQEEAEKMKQVAEEAARLSVA
5dzz.1.A   --

Target   AQEAARLRQLAEEDLAQQRALAEKMLKEKMQAVQEATRLKAEAELLQQQKELAQEQARRLQEDKEQMAQQLAQETQGFQR
5dzz.1.A   --

Target   TLETERQRQLEMSAEAERLRLRVAEMSRAQARAEEDAQRFRKQAEEIGAKLHRTELATQEKVTLVQTLETQRQQSDRDAD
5dzz.1.A   --

Target   RLREAIAELEREKDKLKKEAELLQLKSEEMQTVQQEQLLQETQALQQSFLSEKDSLLQRERFIEEEKAKLERLFQDEVAK
5dzz.1.A   --

Target   AQKLREEQQRQQQQMQQEKQQLLASMEEARRRQHEAEEGVRRKQEELQLLEQQRQQQEQLLAEENRRLRERLEHLEEEHR
5dzz.1.A   --

Target   AALAHSEEITAAQAAATRALPNGQDAADGPAAEPEHAFEGLRQKVPAQQLQEAGILSTEELQRLAQGHTTVAELTQREDV
5dzz.1.A   ------------------------------------VFDGLRKKVTAMQLYECQLIDKTTLDKLLKGKKSVEEVAS--EI

Target   RRYLQGHSSIAGLLLKPANEKLTIYAALRRQLLSPGTALILLEAQAASGFLLDPVRNRRLTVTEAVKEGVVGPELHHKLL
5dzz.1.A   QPFLRGAGSIAGASASP-KEKYSLVEAKRKKLISPESTVMLLEAQAATGGIIDPHRNEKLTVDSAIARDLIDFDDRQQIY

Target   SAERAVTGYKDPYTGEKISLFQAMKKDLIVREHGIRLLEAQIATGGVIDPVHSHRVPVDVAYHRGYFDEEMNRVLQDPSD
5dzz.1.A   AAEKAITGFDDPFSGKTVSVSEAIKKNLIDRETGMRLLEAQIASGGVVDPVNSVFLPKDVALARGLIDRDLYRSLNDPRD

Target   DTKGFFDPNTHENLTYLQLLERCVEDPETGLRLLPLTDQAAKGGELVYTDSEARDVFEKATVSAPFGKFQG--KTVTIWE
5dzz.1.A   SQKNFVDPVTKKKVSYVQLKERCRIEPHTGLLLLSV---------------------QKRSMS-----FQGIRQPVTVTE

Target   LINSEYFTAEQRRDL-------------LRQFRTGKVTVEKIIKIVITVIEEHEQKGQLCFQGLRALVPAAELLESG---
5dzz.1.A   LVDSGILRPSTVNELESGQISYDEVGERIKDFLQGSSCIAGIYNETTKQKLGIYEAMKI---GLVRPGTALELLEAQAAT

Target   --IIDWDLFRQLQLGERSVQEVAEVEGVRRALRGSGVIAGVWLEEARQKLSIYEALKKELLQPEAAVALLEAQAGTGHVI
5dzz.1.A   GFIVDPVSNLRLPVEEAYKRGLVGIEFKEKLLSAERAVTGYNDPETGNIISLFQAMNKELIEKGHGIRLLEAQIATGGII

Target   DPATSARLTVDEAVRAGLVGPELHEKLLSAEKAVTGYKDPYSGQSVSLFQALKKGLIPREQGLRLLDAQLSTGGTVDPSK
5dzz.1.A   DPKESHRLPVDIAYKRGYFNEELSEILSDPSDDTKGFFDPNTEENLTYLQLKERCIKDEETGLCLL--------------

Target   SHRLPLDVACARGYLDEETSTALSAPRDDAKTYYDPRTWELATYSQLQQQCRPDPLTGLSLLPLSEEAARARQQELYSEV
5dzz.1.A   --

Target   QAREAFQKATVEVPVGSFQGRAVTIWELINSEYFTAEQRQELLRQFRTGKVTVEKIIKIVITIVEEVETTRRERLSFSGL
5dzz.1.A   --

Target   RAPVPASELLAAGILSSSQFEQLKDGKTSVKDLSELSSLQTLLQGSGCLAGIYLEESKEKVTIYEAMRRGLLRPSTAILL
5dzz.1.A   --

Target   LEAQAATGFLVDPVRNQRLYVHEAVKAGVVGPELHEKLLSAEKAVTGYKDPYSGSTISLFQAMKKGLVVREHGIRLLEAQ
5dzz.1.A   --

Target   IATGGIIDPVHSHRVPVDVACQRGYFDEEMNRVLEDPSDDTKGFFDPNTHENLTYRQLLERCVEDPETGLRLLPLKGPEK
5dzz.1.A   --

Target   AEVVETTRVYTEEETRRAFEETQIDIPGGGSHGGSTMSLWEVMQSDLIPEEQRAQLMADFQAGRVTKERMIIIIIEIIEK
5dzz.1.A   --

Target   TEIVRQQNLASYDYVRRRLTAEDLYEARVISRESYSLLREGTRSLREVLEAESAWRYLYGTGCVAGVYLPGSRQTLPIYQ
5dzz.1.A   --

Target   ALKKGLLSAEVARLLLEAQAATGFLLDPVKGERLTVDEAVRKGLVGPELHDRLLSAERAVTGYRDPYTEQTISLFQAMKK
5dzz.1.A   --

Target   DLIPAEEALRLLDAQLATGGIVDPRLGFHLPLEVAYQRGYLNKDTHDQLSEPSEVRSYVDPSTDERLSYTQLLRRCRRDE
5dzz.1.A   --

Target   TSGLFLLPLSEARKLTFRGLRKQITVEELVRSHVMDEATAQRLQEGLTSIEEVSKNLQKFLEGTSSIAGVLVDATKERLS
5dzz.1.A   --

```
Target    VYQAMKKGIIRPGTAFELLEAQAATGYVIDPIKGLKLTVEEAVRMGIVGPEFKDKLLSAERAVTGYKDPYSGKLISLFQA
5dzz.1.A  --------------------------------------------------------------------------------

Target    MKKGLILKDHGIRLLEAQIATGGIIDPEESHRLPVDVAYQRGLFDEEMNEILTDPSDDTKGFFDPNTEENLTYLQLMERC
5dzz.1.A  --------------------------------------------------------------------------------

Target    VTDPQTGLRLLPLKEKKRERKTSSKSSVRKRRVVIVDPETGKEMSVYEAYRKGLIDHQTYLELSEQECEWEEITISSSDG
5dzz.1.A  --------------------------------------------------------------------------------

Target    VVKSMIIDRRSGRQYDIDEAIAKSLIDRSALDQYRAGTLSITEFADMLSGNAGGFRSRSSSVGSSSSYPISPAVSRTQLA
5dzz.1.A  --------------------------------------------------------------------------------

Target    SWSDPTEETGPVAGILDTETLEKVSITEAMHRNLVDNITGQRLLEAQACTGGIIDPNTGERFPVTDAVNKGLVDKIMVDR
5dzz.1.A  --------------------------------------------------------------------------------

Target    INLAQKAFCGFEDPRTKTKMSAAQALKKGWLYYEAGQRFLEVQYLTGGLIEPDTPGRVPLDEALQRGTVDARTAQKLRDV
5dzz.1.A  --------------------------------------------------------------------------------

Target    SAYSKYLTCPKTKLKISYKDALDRSMVEEGTGLRLLEAAAQSSKGYYSPYSVSGSGSTTGSRSGSRTGSRAGSRRGSFDA
5dzz.1.A  --------------------------------------------------------------------------------

Target    TGSGFSMTFSSSSYSSSGYGRRYASGPTSSLGGPESAAA
5dzz.1.A  ---------------------------------------
```

Model #07	File	Built with	Oligo-State	Ligands	GMQE	QMEANDisCo Global
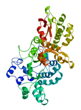	PDB	ProMod3 3.3.0	monomer	None	0.01	0.55 ± 0.05

Template	Seq Identity	Oligo-state	QSQE	Found by	Method	Resolution	Seq Similarity	Range	Coverage	Description
5dzz.1.A	45.24	monomer	0.00	BLAST	X-ray	2.60Å	0.40	1232 - 1736	0.17	Desmoplakin

The template contained no ligands.

```
Target    RALAEEAKRQRQLAEEDAARQRAEAERVLSEKLAAISEATRLKTEAEIALKEKEAENERLRRLAEDEAFQRRRLEEQAAQ
5dzz.1.A  --------------------------------------------------------------------------------

Target    HKADIEERLAQLRKASESELERQKGLVEDTLRQRRQVEEEILALKASFEKAAAGKAELELELGRIRGNAEDTLRSKEQAE
5dzz.1.A  --------------------------------------------------------------------------------

Target    QEAARQRQLAAEEERRRREAEERVQKSLAAEEEAARQRKAALEEVERLKAKVEEARRLRERAEHESVRQLQLAQEAAQKR
5dzz.1.A  --------------------------------------------------------------------------------

Target    LQAEEKAHAFAVQQKEQELQQTLQQEQSVLEKLRSEAEAARRAAEEAEEARERAEREAAQSRQRVEEAERLKQAAEEQAQ
5dzz.1.A  --------------------------------------------------------------------------------

Target    AQAQAQAAAEKLRKEAEQEAARRAQAEQAALRQKQAADAEMEKHKKFAEQTLRQKAQVEQELTALRLKLEETDHQKSILD
5dzz.1.A  --------------------------------------------------------------------------------

Target    QELQRLKAEVTEAARQRSQVEEELFSVRVQMEELGKLKARIEAENRALILRDKDNTQRLLQEEAEKMKQVAEEAARLSVA
5dzz.1.A  --------------------------------------------------------------------------------

Target    AQEAARLRQLAEEDLAQQRALAEKMLKEKMQAVQEATRLKAEAELLQQQKELAQEQARRLQEDKEQMAQQLAQETQGFQR
5dzz.1.A  --------------------------------------------------------------------------------

Target    TLETERQRQLEMSAEAERLRLRVAEMSRAQARAEEDAQRFRKQAEEIGAKLHRTELATQEKVTLVQTLETQRQQSDRDAD
5dzz.1.A  --------------------------------------------------------------------------------

Target    RLREAIAELEREKDKLKKEAELLQLKSEEMQTVQQEQLLQETQALQQSFLSEKDSLLQRERFIEEEKAKLERLFQDEVAK
5dzz.1.A  --------------------------------------------------------------------------------

Target    AQKLREEQQRQQQQMQQEKQQLLASMEEARRRQHEAEEGVRRKQEELQLLEQQRQQQEQLLAEENRRLRERLEHLEEEHR
5dzz.1.A  --------------------------------------------------------------------------------
```

```
Target     AALAHSEEITAAQAAATRALPNGGQDAADGPAAEPEHAFEGLRQKVPAQQLQEAGILSTEELQRLAQGHTTVAELTQREDV
5dzz.1.A   --------------------------------------------------------------------------------

Target     RRYLQGHSSIAGLLLKPANEKLTIYAALRRQLLSPGTALILLEAQAASGFLLDPVRNRRLTVTEAVKEGVVGPELHHKLL
5dzz.1.A   --------------------------------------------------------------------------------

Target     SAERAVTGYKDPYTGEKISLFQAMKKDLIVREHGIRLLEAQIATGGVIDPVHSHRVPVDVAYHRGYFDEEMNRVLQDPSD
5dzz.1.A   --------------------------------------------------------------------------------

Target     DTKGFFDPNTHENLTYLQLLERCVEDPETGLRLLPLTDQAAKGGELVYTDSEARDVFEKATVSAPFGKFQGKTVTIWELI
5dzz.1.A   --------------------------------------------------------------------------------

Target     NSEYFTAEQRRDLLRQFRTGKVTVEKIIKIVITVIEEHEQKGQLCFQGLRALVPAAELLESGIIDWDLFRQLQLGERSVQ
5dzz.1.A   --------------------------------------------------------------------------------

Target     EVAEVEGVRRALRGSGVIAGVWLEEARQKLSIYEALKKELLQPEAAVALLEAQAGTGHVIDPATSARLTVDEAVRAGLVG
5dzz.1.A   -------------------------------VFDGLRKKV----TAMQLYECQLIDKTTLDKLLKGKKSVEE------VA

Target     PELHEKLLSAEKAVTGYKDPYSGQSVSLFQALKKGLIPREQGLRLLDAQLSTGGTVDPSKSHRLPLDVACARGYLDEETS
5dzz.1.A   SEIQPFLRGAGSIAGASASPK--EKYSLVEAKRKKLISPESTVMLLEAQAATGGIIDPHRNEKLTVDSAIARDLIDFDDR

Target     TALSAPRDDAKTYYDPRTWELATYSQLQQQCRPDPLTGLSLLP----------------LSEEAARAR---QQELYSEVQ
5dzz.1.A   QQIYAAEKAITGFDDPFSGKTVSVSEAIKKNLIDRETGMRLLEAQIASGGVVDPVNSVFLPKDVALARGLIDRDLYRSLN

Target     AREAFQKATVEVPVGSFQGRAVTIWELINSEYFTAEQRQELLRQFRTGKVTVEKIIKIVITIVEEVETTRRERLSFSGLR
5dzz.1.A   DPRDSQKNFVD-PVTKKKVSYVQLKERCRIEPHTG----------------------------LLLLSVQKRSMSFQGIR

Target     APVPASELLAAGILSSSQFEQLKDGKTSVKDLSELSSLQTLLQGSGCLAGIYLEESKEKVTIYEAMRRGLLRPSTAILLL
5dzz.1.A   QPVTVTELVDSGILRPSTVNELESGQISYDEVGERIKDFLQGSSCI--AGIYNETTKQKLGIYEAMKIGLVRPGTALELL

Target     EAQAATGFLVDPVRNQRLYVHEAVKAGVVGPELHEKLLSAEKAVTGYKDPYSGSTISLFQAMKKGLVVREHGIRLLEAQI
5dzz.1.A   EAQAATGFIVDPVSNLRLPVEEAYKRGLVGIEFKEKLLSAERAVTGYNDPETGNIISLFQAMNKELIEKGHGIRLLEAQI

Target     ATGGIIDPVHSHRVPVDVACQRGYFDEEMNRVLEDPSDDTKGFFDPNTHENLTYRQLLERCVEDPETGLRLLPLKGPEKA
5dzz.1.A   ATGGIIDPKESHRLPVDIAYKRGYFNEELSEILSDPSDDTKGFFDPNTEENLTYLQLKERCIKDEETGLCLLPLK-----

Target     EVVETTRVYTEEETRRAFEETQIDIPGGGSHGGSTMSLWEVMQSDLIPEEQRAQLMADFQAGRVTKERMIIIIIEIIEKT
5dzz.1.A   --------------------------------------------------------------------------------

Target     EIVRQQNLASYDYVRRRLTAEDLYEARVISRESYSLLREGTRSLREVLEAESAWRYLYGTGCVAGVYLPGSRQTLPIYQA
5dzz.1.A   --------------------------------------------------------------------------------

Target     LKKGLLSAEVARLLLEAQAATGFLLDPVKGERLTVDEAVRKGLVGPELHDRLLSAERAVTGYRDPYTEQTISLFQAMKKD
5dzz.1.A   --------------------------------------------------------------------------------

Target     LIPAEEALRLLDAQLATGGIVDPRLGFHLPLEVAYQRGYLNKDTHDQLSEPSEVRSYVDPSTDERLSYTQLLRRCRRDET
5dzz.1.A   --------------------------------------------------------------------------------

Target     SGLFLLPLSEARKLTFRGLRKQITVEELVRSHVMDEATAQRLQEGLTSIEEVSKNLQKFLEGTSSIAGVLVDATKERLSV
5dzz.1.A   --------------------------------------------------------------------------------

Target     YQAMKKGIIRPGTAFELLEAQAATGYVIDPIKGLKLTVEEAVRMGIVGPEFKDKLLSAERAVTGYKDPYSGKLISLFQAM
5dzz.1.A   --------------------------------------------------------------------------------

Target     KKGLILKDHGIRLLEAQIATGGIIDPEESHRLPVDVAYQRGLFDEEMNEILTDPSDDTKGFFDPNTEENLTYLQLMERCV
5dzz.1.A   --------------------------------------------------------------------------------

Target     TDPQTGLRLLPLKEKKRERKTSSKSSVRKRRVVIVDPETGKEMSVYEAYRKGLIDHQTYLELSEQECEWEEITISSSDGV
5dzz.1.A   --------------------------------------------------------------------------------

Target     VKSMIIDRRSGRQYDIDEAIAKSLIDRSALDQYRAGTLSITEFADMLSGNAGGFRSRSSSVGSSSSYPISPAVSRTQLAS
5dzz.1.A   --------------------------------------------------------------------------------

Target     WSDPTEETGPVAGILDTETLEKVSITEAMHRNLVDNITGQRLLEAQACTGGIIDPNTGERFPVTDAVNKGLVDKIMVDRI
5dzz.1.A   --------------------------------------------------------------------------------

Target     NLAQKAFCGFEDPRTKTKMSAAQALKKGWLYYEAGQRFLEVQYLTGGLIEPDTPGRVPLDEALQRGTVDARTAQKLRDVS
5dzz.1.A   --------------------------------------------------------------------------------

Target     AYSKYLTCPKTKLKISYKDALDRSMVEEGTGLRLLEAAAQSSKGYYSPYSVSGSGSTTGSRSGSRTGSRAGSRRGSFDAT
5dzz.1.A   --------------------------------------------------------------------------------

Target     GSGFSMTFSSSSYSSSGYGRRYASGPTSSLGGPESAAA
```

```
5dzz.1.A    --------------------------------------
```

Model #09	File	Built with	Oligo-State	Ligands	GMQE	QMEANDisCo Global
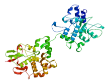	PDB	ProMod3 3.3.0	monomer	None	0.01	0.55 ± 0.05

Template	Seq Identity	Oligo-state	QSQE	Found by	Method	Resolution	Seq Similarity	Range	Coverage	Description
5dzz.1.A	41.31	monomer	0.00	BLAST	X-ray	2.60Å	0.39	904 - 1406	0.17	Desmoplakin

The template contained no ligands.

```
Target      RALAEEAKRQRQLAEEDAARQRAEAERVLSEKLAAISEATRLKTEAEIALKEKEAENERLRRLAEDEAFQRRRLEEQAAQ
5dzz.1.A    --------------------------------------------------------------------------------

Target      HKADIEERLAQLRKASESELERQKGLVEDTLRQRRQVEEEILALKASFEKAAAGKAELELELGRIRGNAEDTLRSKEQAE
5dzz.1.A    --------------------------------------------------------------------------------

Target      QEAARQRQLAAEEERRRREAEERVQKSLAAEEEAARQRKAALEEVERLKAKVEEARRLRERAEHESVRQLQLAQEAAQKR
5dzz.1.A    --------------------------------------------------------------------------------

Target      LQAEEKAHAFAVQQKEQELQQTLQQEQSVLEKLRSEAEAARRAAEEAEEARERAEREAAQSRQRVEEAERLKQAAEEQAQ
5dzz.1.A    --------------------------------------------------------------------------------

Target      AQAQAQAAAEKLRKEAEQEAARRAQAEQAALRQKQAADAEMEKHKKFAEQTLRQKAQVEQELTALRLKLEETDHQKSILD
5dzz.1.A    --------------------------------------------------------------------------------

Target      QELQRLKAEVTEAARQRSQVEEELFSVRVQMEELGKLKARIEAENRALILRDKDNTQRLLQEEAEKMKQVAEEAARLSVA
5dzz.1.A    --------------------------------------------------------------------------------

Target      AQEAARLRQLAEEDLAQQRALAEKMLKEKMQAVQEATRLKAEAELLQQQKELAQEQARRLQEDKEQMAQQLAQETQGFQR
5dzz.1.A    --------------------------------------------------------------------------------

Target      TLETERQRQLEMSAEAERLRLRVAEMSRAQARAEEDAQRFRKQAEEIGAKLHRTELATQEKVTLVQTLETQRQQSDRDAD
5dzz.1.A    --------------------------------------------------------------------------------

Target      RLREAIAELEREKDKLKKEAELLQLKSEEMQTVQQEQLLQETQALQQSFLSEKDSLLQRERFIEEEKAKLERLFQDEVAK
5dzz.1.A    --------------------------------------------------------------------------------

Target      AQKLREEQQRQQQQMQQEKQQLLASMEEARRRQHEAEEGVRRKQEELQLLEQQRQQQEQLLAEENRRLRERLEHLEEEHR
5dzz.1.A    --------------------------------------------------------------------------------

Target      AALAHSEEITAAQAAATRALPNGQDAADGPAAEPEHAFEGLRQKVPAQQLQEAGILSTEELQRLAQGHTTVAELTQREDV
5dzz.1.A    --------------------------------------------------------------------------------

Target      RRYLQGHSSIAGLLLKPANEKLTIYAALRRQLLSPGTALILLEAQAASGFLLDPVRNRRLTVTEAVKEGVVGPELHHKLL
5dzz.1.A    -----------------------VFDGLRKKV----TAMQLYECQLIDKTTLDKLLKGKKSVEE------VASEIQPFLR

Target      SAERAVTGYKDPYTGEKISLFQAMKKDLIVREHGIRLLEAQIATGGVIDPVHSHRVPVDVAYHRGYFDEEMNRVLQDPSD
5dzz.1.A    GAGSIAGASASPK--EKYSLVEAKRKKLISPESTVMLLEAQAATGGIIDPHRNEKLTVDSAIARDLIDFDDRQQIYAAEK

Target      DTKGFFDPNTHENLTYLQLLERCVEDPETGLRLLPLTDQAAKGGELVYTDSEARDVFEKATVSAPFGKFQGKTVTIWELI
5dzz.1.A    AITGFDDPFSGKTVSVSEAIKKNLIDRETGMRLLEA--QIASGGVVDPVNS----VFLPKDVALARG-----------LI

Target      NSE-YFTAEQRRDLLRQFRTGKVTVEKIIKIVITVIEEHE---------QKGQLCFQGLRALVPAAELLESGIIDWDLFR
5dzz.1.A    DRDLYRSLNDPRDSQKNFVDPVTKKKVSYVQLKERCRIEPHTGLLLLSVQKRSMSFQGIRQPVTVTELVDSGILRPSTVN

Target      QLQLGERSVQEVAEVEGVRRALRGSGVIAGVWLEEARQKLSIYEALKKELLQPEAAVALLEAQAGTGHVIDPATSARLTV
5dzz.1.A    ELESGQISYDEVGE--RIKDFLQGSSCIAGIYNETTKQKLGIYEAMKIGLVRPGTALELLEAQAATGFIVDPVSNLRLPV

Target      DEAVRAGLVGPELHEKLLSAEKAVTGYKDPYSGQSVSLFQALKKGLIPREQGLRLLDAQLSTGGTVDPSKSHRLPLDVAC
5dzz.1.A    EEAYKRGLVGIEFKEKLLSAERAVTGYNDPETGNIISLFQAMNKELIEKGHGIRLLEAQIATGGIIDPKESHRLPVDIAY

Target      ARGYLDEETSTALSAPRDDAKTYYDPRTWELATYSQLQQQCRPDPLTGLSLLPLSEEAARARQQELYSEVQAREAFQKAT
```

```
5dzz.1.A   KRGYFNEELSEILSDPSDDTKGFFDPNTEENLTYLQLKERCIKDEETGLCLLPLKE------------------------

Target     VEVPVGSFQGRAVTIWELINSEYFTAEQRQELLRQFRTGKVTVEKIIKIVITIVEEVETTRRERLSFSGLRAPVPASELL
5dzz.1.A   --------------------------------------------------------------------------------

Target     AAGILSSSQFEQLKDGKTSVKDLSELSSLQTLLQGSGCLAGIYLEESKEKVTIYEAMRRGLLRPSTAILLLEAQAATGFL
5dzz.1.A   --------------------------------------------------------------------------------

Target     VDPVRNQRLYVHEAVKAGVVGPELHEKLLSAEKAVTGYKDPYSGSTISLFQAMKKGLVVREHGIRLLEAQIATGGIIDPV
5dzz.1.A   --------------------------------------------------------------------------------

Target     HSHRVPVDVACQRGYFDEEMNRVLEDPSDDTKGFFDPNTHENLTYRQLLERCVEDPETGLRLLPLKGPEKAEVVETTRVY
5dzz.1.A   --------------------------------------------------------------------------------

Target     TEEETRRAFEETQIDIPGGGSHGGSTMSLWEVMQSDLIPEEQRAQLMADFQAGRVTKERMIIIIIEIIEKTEIVRQQNLA
5dzz.1.A   --------------------------------------------------------------------------------

Target     SYDYVRRRLTAEDLYEARVISRESYSLLREGTRSLREVLEAESAWRYLYGTGCVAGVYLPGSRQTLPIYQALKKGLLSAE
5dzz.1.A   --------------------------------------------------------------------------------

Target     VARLLLEAQAATGFLLDPVKGERLTVDEAVRKGLVGPELHDRLLSAERAVTGYRDPYTEQTISLFQAMKKDLIPAEEALR
5dzz.1.A   --------------------------------------------------------------------------------

Target     LLDAQLATGGIVDPRLGFHLPLEVAYQRGYLNKDTHDQLSEPSEVRSYVDPSTDERLSYTQLLRRCRRDETSGLFLLPLS
5dzz.1.A   --------------------------------------------------------------------------------

Target     EARKLTFRGLRKQITVEELVRSHVMDEATAQRLQEGLTSIEEVSKNLQKFLEGTSSIAGVLVDATKERLSVYQAMKKGII
5dzz.1.A   --------------------------------------------------------------------------------

Target     RPGTAFELLEAQAATGYVIDPIKGLKLTVEEAVRMGIVGPEFKDKLLSAERAVTGYKDPYSGKLISLFQAMKKGLILKDH
5dzz.1.A   --------------------------------------------------------------------------------

Target     GIRLLEAQIATGGIIDPEESHRLPVDVAYQRGLFDEEMNEILTDPSDDTKGFFDPNTEENLTYLQLMERCVTDPQTGLRL
5dzz.1.A   --------------------------------------------------------------------------------

Target     LPLKEKKRERKTSSKSSVRKRRVVIVDPETGKEMSVYEAYRKGLIDHQTYLELSEQECEWEEITISSSDGVVKSMIIDRR
5dzz.1.A   --------------------------------------------------------------------------------

Target     SGRQYDIDEAIAKSLIDRSALDQYRAGTLSITEFADMLSGNAGGFRSRSSSVGSSSSYPISPAVSRTQLASWSDPTEETG
5dzz.1.A   --------------------------------------------------------------------------------

Target     PVAGILDTETLEKVSITEAMHRNLVDNITGQRLLEAQACTGGIIDPNTGERFPVTDAVNKGLVDKIMVDRINLAQKAFCG
5dzz.1.A   --------------------------------------------------------------------------------

Target     FEDPRTKTKMSAAQALKKGWLYYEAGQRFLEVQYLTGGLIEPDTPGRVPLDEALQRGTVDARTAQKLRDVSAYSKYLTCP
5dzz.1.A   --------------------------------------------------------------------------------

Target     KTKLKISYKDALDRSMVEEGTGLRLLEAAAQSSKGYYSPYSVSGSGSTTGSRSGSRTGSRAGSRRGSFDATGSGFSMTFS
5dzz.1.A   --------------------------------------------------------------------------------

Target     SSSYSSSGYGRRYASGPTSSLGGPESAAA
5dzz.1.A   -----------------------------
```

Model #10	File	Built with	Oligo-State	Ligands	GMQE	QMEANDisCo Global
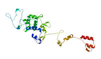	PDB	ProMod3 3.3.0	monomer	None	0.00	0.37 ± 0.05

Template	Seq Identity	Oligo-state	QSQE	Found by	Method	Resolution	Seq Similarity	Range	Coverage	Description
5dzz.1.A	35.94	monomer	0.00	BLAST	X-ray	2.60Å	0.37	2386 - 2660	0.08	Desmoplakin

The template contained no ligands.

```
Target     RALAEEAKRQRQLAEEDAARQRAEAERVLSEKLAAISEATRLKTEAEIALKEKEAENERLRRLAEDEAFQRRRLEEQAAQ
5dzz.1.A   --------------------------------------------------------------------------------

Target     HKADIEERLAQLRKASESELERQKGLVEDTLRQRRQVEEEILALKASFEKAAAGKAELELELGRIRGNAEDTLRSKEQAE
5dzz.1.A   --------------------------------------------------------------------------------

Target     QEAARQRQLAAEEERRRREAEERVQKSLAAEEEAARQRKAALEEVERLKAKVEEARRLRERAEHESVRQLQLAQEAAQKR
5dzz.1.A   --------------------------------------------------------------------------------

Target     LQAEEKAHAFAVQQKEQELQQTLQQEQSVLEKLRSEAEAARRAAEEAEEARERAEREAAQSRQRVEEAERLKQAAEEQAQ
5dzz.1.A   --------------------------------------------------------------------------------

Target     AQAQAQAAAEKLRKEAEQEAARRAQAEQAALRQKQAADAEMEKHKKFAEQTLRQKAQVEQELTALRLKLEETDHQKSILD
5dzz.1.A   --------------------------------------------------------------------------------

Target     QELQRLKAEVTEAARQRSQVEEELFSVRVQMEELGKLKARIEAENRALILRDKDNTQRLLQEEAEKMKQVAEEAARLSVA
5dzz.1.A   --------------------------------------------------------------------------------

Target     AQEAARLRQLAEEDLAQQRALAEKMLKEKMQAVQEATRLKAEAELLQQQKELAQEQARRLQEDKEQMAQQLAQETQGFQR
5dzz.1.A   --------------------------------------------------------------------------------

Target     TLETERQRQLEMSAEAERLRLRVAEMSRAQARAEEDAQRFRKQAEEIGAKLHRTELATQEKVTLVQTLETQRQQSDRDAD
5dzz.1.A   --------------------------------------------------------------------------------

Target     RLREAIAELEREKDKLKKEAELLQLKSEEMQTVQQEQLLQETQALQQSFLSEKDSLLQRERFIEEEKAKLERLFQDEVAK
5dzz.1.A   --------------------------------------------------------------------------------

Target     AQKLREEQQRQQQMQQEKQQLLASMEEARRRQHEAEEGVRRKQEELQLLEQQRQQQEQLLAEENRRLRERLEHLEEEHR
5dzz.1.A   --------------------------------------------------------------------------------

Target     AALAHSEEITAAQAAATRALPNGQDAADGPAAEPEHAFEGLRQKVPAQQLQEAGILSTEELQRLAQGHTTVAELTQREDV
5dzz.1.A   --------------------------------------------------------------------------------

Target     RRYLQGHSSIAGLLLKPANEKLTIYAALRRQLLSPGTALILLEAQAASGFLLDPVRNRRLTVTEAVKEGVVGPELHHKLL
5dzz.1.A   --------------------------------------------------------------------------------

Target     SAERAVTGYKDPYTGEKISLFQAMKKDLIVREHGIRLLEAQIATGGVIDPVHSHRVPVDVAYHRGYFDEEMNRVLQDPSD
5dzz.1.A   --------------------------------------------------------------------------------

Target     DTKGFFDPNTHENLTYLQLLERCVEDPETGLRLLPLTDQAAKGGELVYTDSEARDVFEKATVSAPFGKFQGKTVTIWELI
5dzz.1.A   --------------------------------------------------------------------------------

Target     NSEYFTAEQRRDLLRQFRTGKVTVEKIIKIVITVIEEHEQKGQLCFQGLRALVPAAELLESGIIDWDLFRQLQLGERSVQ
5dzz.1.A   --------------------------------------------------------------------------------

Target     EVAEVEGVRRALRGSGVIAGVWLEEARQKLSIYEALKKELLQPEAAVALLEAQAGTGHVIDPATSARLTVDEAVRAGLVG
5dzz.1.A   --------------------------------------------------------------------------------

Target     PELHEKLLSAEKAVTGYKDPYSGQSVSLFQALKKGLIPREQGLRLLDAQLSTGGTVDPSKSHRLPLDVACARGYLDEETS
5dzz.1.A   --------------------------------------------------------------------------------

Target     TALSAPRDDAKTYYDPRTWELATYSQLQQQCRPDPLTGLSLLPLSEEAARARQQELYSEVQAREAFQKATVEVPVGSFQG
5dzz.1.A   --------------------------------------------------------------------------------

Target     RAVTIWELINSEYFTAEQRQELLRQFRTGKVTVEKIIKIVITIVEEVETTRRERLSFSGLRAPVPASELLAAGILSSSQF
5dzz.1.A   --------------------------------------------------------------------------------

Target     EQLKDGKTSVKDLSELSSLQTLLQGSGCLAGIYLEESKEKVTIYEAMRRGLLRPSTAILLLEAQAATGFLVDPVRNQRLY
5dzz.1.A   --------------------------------------------------------------------------------

Target     VHEAVKAGVVGPELHEKLLSAEKAVTGYKDPYSGSTISLFQAMKKGLVVREHGIRLLEAQIATGGIIDPVHSHRVPVDVA
5dzz.1.A   --------------------------------------------------------------------------------

Target     CQRGYFDEEMNRVLEDPSDDTKGFFDPNTHENLTYRQLLERCVEDPETGLRLLPLKGPEKAEVVETTRVYTEEETRRAFE
5dzz.1.A   --------------------------------------------------------------------------------

Target     ETQIDIPGGGSHGGSTMSLWEVMQSDLIPEEQRAQLMADFQAGRVTKERMIIIIIEIIEKTEIVRQQNLASYDYVRRRLT
5dzz.1.A   --------------------------------------------------------------------------------

Target     AEDLYEARVISRESYSLLREGTRSLREVLEAESAWRYLYGTGCVAGVYLPGSRQTLPIYQALKKGLLSAEVARLLLEAQA
5dzz.1.A   --------------------------------------------------------------------------------

Target     ATGFLLDPVKGERLTVDEAVRKGLVGPELHDRLLSAERAVTGYRDPYTEQTISLFQAMKKDLIPAEEALRLLDAQLATGG
5dzz.1.A   --------------------------------------------------------------------------------
```

```
Target     IVDPRLGFHLPLEVAYQRGYLNKDTHDQLSEPSEVRSYVDPSTDERLSYTQLLRRCRRDETSGLFLLPLSEARKLTFRGL
5dzz.1.A   --------------------------------------------------------------------------------

Target     RKQITVEELVRSHVMDEATAQRLQEGLTSIEEVSKNLQKFLEGTSSIAGVLVDATKERLSVYQAMKKGIIRPGTAFELLE
5dzz.1.A   --------------------------------------------------------------------------------

Target     AQAATGYVIDPIKGLKLTVEEAVRMGIVGPEFKDKLLSAERAVTGYKDPYSGKLISLFQAMKKGLILKDHGIRLLEAQIA
5dzz.1.A   --------------------------------------------------------------------------------

Target     TGGIIDPEESHRLPVDVAYQRGLFDEEMNEILTDPSDDTKGFFDPNTEENLTYLQLMERCVTDPQTGLRLLPLKEKKRER
5dzz.1.A   --------------------------------------------------------------------------------

Target     KTSSKSSVRKRRVVIVDPETGKEMSVYEAYRKGLIDHQTYLELSEQECEWEEITISSSDGVVKSMIIDRRSGRQYDIDEA
5dzz.1.A   -----------------------------------------------------------------IIDPHRNEKLTVDSA

Target     IAKSLIDRSALDQYRAGTLSITEFADMLSGNAGGFRSRSSSVGSSSSYPISPAVSRTQLASWSDPTEETGPVAGILDTET
5dzz.1.A   IARDLIDFDDRQQIYAAEKAITGFDDPFSGKT------------------------------------------------

Target     LEKVSITEAMHRNLVDNITGQRLLEAQACTGGIIDPNTGERFPVTDAVNKGLVDKIMVDRINLAQKAFCGFEDPRTKTKM
5dzz.1.A   ---VSVSEAIKKNLIDRETGMRLLEAQIASGGVVDPVNSVFLPKDVALARGLIDRDLYRSLNDPRDSQKNFVDPVTKKKV

Target     SAAQALKKGWLYYEAGQRFLEVQ-----------------YLTGGLIEPDT-----PGRVPLDEALQRGTVDARTAQKLR
5dzz.1.A   SYVQLKERCRIEPHTGLLLLSVQKRSMSFQGIRQPVTVTELVDSGILRPSTVNELESGQISYDE------VGERIKDFLQ

Target     DVSAYSKYLTCPKTKLKISYKDALDRSMVEEGTGLRLLEAAAQSSKGYYSPYSVSGSGSTTGSRSGSRTGSRAGSRRGSF
5dzz.1.A   GSSCIAGIYN-ETTKQKLGIYEAMKIGLVRPGTALELLEAQA--------------------------------------

Target     DATGSGFSMTFSSSSYSSSGYGRRYASGPTSSLGGPESAAA
5dzz.1.A   -----------------------------------------
```

Materials and Methods

Template Search

Template search with has been performed against the SWISS-MODEL template library (SMTL, last update: 2023-11-08, last included PDB release: 2023-11-03).

Template Selection

For each identified template, the template's quality has been predicted from features of the target-template alignment. The templates with the highest quality have then been selected for model building.

Model Building

Models are built based on the target-template alignment using ProMod3 (Studer et al.). Coordinates which are conserved between the target and the template are copied from the template to the model. Insertions and deletions are remodelled using a fragment library. Side chains are then rebuilt. Finally, the geometry of the resulting model is regularized by using a force field.

Model Quality Estimation

The global and per-residue model quality has been assessed using the QMEAN scoring function (Studer et al.).

Ligand Modelling

Ligands present in the template structure are transferred by homology to the model when the following criteria are met: (a) The ligands are annotated as biologically relevant in the template library, (b) the ligand is in contact with the model, (c) the ligand is not clashing with the protein, (d) the residues in contact with the ligand are conserved between the target and the template. If any of these four criteria is not satisfied, a certain ligand will not be included in the model. The model summary includes information on why and which ligand has not been included.

Oligomeric State Conservation

The quaternary structure annotation of the template is used to model the target sequence in its oligomeric form. The method (Bertoni et al.) is based on a supervised machine learning algorithm, Support Vector Machines (SVM), which combines interface conservation, structural clustering, and other template features to provide a quaternary structure quality estimate (QSQE). The QSQE score is a number between 0 and 1, reflecting the expected accuracy of the interchain contacts for a

model built based a given alignment and template. Higher numbers indicate higher reliability. This complements the GMQE score which estimates the accuracy of the tertiary structure of the resulting model.

References

- **BLAST**
  Camacho, C., Coulouris, G., Avagyan, V., Ma, N., Papadopoulos, J., Bealer, K., Madden, T.L. BLAST+: architecture and applications. BMC Bioinformatics 10, 421-430 (2009).
- **HHblits**
  Steinegger, M., Meier, M., Mirdita, M., Vöhringer, H., Haunsberger, S. J., Söding, J. HH-suite3 for fast remote homology detection and deep protein annotation. BMC Bioinformatics 20, 473 (2019).

Table T1:

Primary amino acid sequence for which templates were searched and models were built.

```
RALAEEEAKRQRQLAEEDAARQRAEAERVLSEKLAAISEATRLKTEAEIALKEKEAENERLRRLAEDEAFQRRRLEEQAAQHKADIEERLAQLRKASESEL
ERQKGLVEDTLRQRRQVEEEILALKASFEKAAAGKAELELELGRIRGNAEDTLRSKEQAEQEAARQRQLAAEEERRRREAEERVQKSLAAEEEAARQRKA
ALEEVERLKAKVEEARRLRERAEHESVRQLQLAQEAAQKRLQAEEKAHAFAVQQKEQELQQTLQQEQSVLEKLRSEAEAARRAAEEAEEARERAEREAAQ
SRQRVEEAERLKQAAEEQAQAQAQAQAAAEKLRKEAEQEAARRAQAEQAALRQKQAADAEMEKHKKFAEQTLRQKAQVEQELTALRLKLEETDHQKSILD
QELQRLKAEVTEAARQRSQVEEELFSVRVQMEELGKLKARIEAENRALILRDKDNTQRLLQEEAEKMKQVAEEAARLSVAAQEAARLRQLAEEDLAQQRA
LAEKMLKEKMQAVQEATRLKAEAELLQQQKELAQEQARRLQEDKEQMAQQLAQETQGFQRTLETERQRQLEMSAEAERLRLRVAEMSRAQARAEEDAQRF
RKQAEEIGAKLHRTELATQEKVTLVQTLETQRQQSDRDADRLREAIAELEREKDKLKKEAELLQLKSEEMQTVQQEQLLQETQALQQSFLSEKDSLLQRE
RFIEEEKAKLERLFQDEVAKAQKLREEQQRQQQQMQQEKQQLLASMEEARRRQHEAEEGVRRKQEELQLLEQQRQQQEQLLAEENRRLRERLEHLEEEHR
AALAHSEEITAAQAAATRALPNGQDAADGPAAEPEHAFEGLRQKVPAQQLQEAGILSTEELQRLAQGHTTVAELTQREDVRRYLQGHSSIAGLLLKPANE
KLTIYAALRRQLLSPGTALILLEAQAASGFLLDPVRNRRLTVTEAVKEGVVGPELHHKLLSAERAVTGYKDPYTGEKISLFQAMKKDLIVREHGIRLLEA
QIATGGVIDPVHSHRVPVDVAYHRGYFDEEMNRVLQDPSDDTKGFFDPNTHENLTYLQLLERCVEDPETGLRLLPLTDQAAKGGELVYTDSEARDVFEKA
TVSAPFGKFQGKTVTIWELINSEYFTAEQRRDLLRQFRTGKVTVEKIIKIVITVIEEHEQKGQLCFQGLRALVPAAELLESGIIDWDLFRQLQLGERSVQ
EVAEVEGVRRALRGSGVIAGVWLEEARQKLSIYEALKKELLQPEAAVALLEAQAGTGHVIDPATSARLTVDEAVRAGLVGPELHEKLLSAEKAVTGYKDP
YSGQSVSLFQALKKGLIPREQGLRLLDAQLSTGGTVDPSKSHRLPLDVACARGYLDEETSTALSAPRDDAKTYYDPRTWELATYSQLQQQCRPDPLTGLS
LLPLSEEAARARQQELYSEVQAREAFQKATVEVPVGSFQGRAVTIWELINSEYFTAEQRQELLRQFRTGKVTVEKIIKIVITIVEEVETTRRERLSFSGL
RAPVPASELLAAGILSSSQFEQLKDGKTSVKDLSELSSLQTLLQGSGCLAGIYLEESKEKVTIYEAMRRGLLRPSTAILLLEAQAATGFLVDPVRNQRLY
VHEAVKAGVVGPELHEKLLSAEKAVTGYKDPYSGSTISLFQAMKKGLVVREHGIRLLEAQIATGGIIDPVHSHRVPVDVACQRGYFDEEMNRVLEDPSDD
TKGFFDPNTHENLTYRQLLERCVEDPETGLRLLPLKGPEKAEVVETTRVYTEEETRRAFEETQIDIPGGGSHGGSTMSLWEVMQSDLIPEEQRAQLMADF
QAGRVTKERMIIIIIEIIEKTEIVRQQNLASYDYVRRRLTAEDLYEARVISRESYSLLREGTRSLREVLEAESAWRYLYGTGCVAGVYLPGSRQTLPIYQ
ALKKGLLSAEVARLLLEAQAATGFLLDPVKGERLTVDEAVRKGLVGPELHDRLLSAERAVTGYRDPYTEQTISLFQAMKKDLIPAEEALRLLDAQLATGG
IVDPRLGFHLPLEVAYQRGYLNKDTHDQLSEPSEVRSYVDPSTDERLSYTQLLRRCRRDETSGLFLLPLSEARKLTFRGLRKQITVEELVRSHVMDEATA
QRLQEGLTSIEEVSKNLQKFLEGTSSIAGVLVDATKERLSVYQAMKKGIIRPGTAFELLEAQAATGYVIDPIKGLKLTVEEAVRMGIVGPEFKDKLLSAE
RAVTGYKDPYSGKLISLFQAMKKGLILKDHGIRLLEAQIATGGIIDPEESHRLPVDVAYQRGLFDEEMNEILTDPSDDTKGFFDPNTEENLTYLQLMERC
VTDPQTGLRLLPLKEKKRERKTSSKSSVRKRRVVIVDPETGKEMSVYEAYRKGLIDHQTYLELSEQECEWEEITISSSDGVVKSMIIDRRSGRQYDIDEA
IAKSLIDRSALDQYRAGTLSITEFADMLSGNAGGFRSRSSSVGSSSSYPISPAVSRTQLASWSDPTEETGPVAGILDTETLEKVSITEAMHRNLVDNITG
QRLLEAQACTGGIIDPNTGERFPVTDAVNKGLVDKIMVDRINLAQKAFCGFEDPRTKTKMSAAQALKKGWLYYEAGQRFLEVQYLTGGLIEPDTPGRVPL
DEALQRGTVDARTAQKLRDVSAYSKYLTCPKTKLKISYKDALDRSMVEEGTGLRLLEAAAQSSKGYYSPYSVSGSGSTTGSRSGSRTGSRAGSRRGSFDA
TGSGFSMTFSSSSYSSSGYGRRYASGPTSSLGGPESAAA
```

Table T2:

Template	Seq Identity	Oligo-state	QSQE	Found by	Method	Resolution	Seq Similarity	Coverage	Description
H9FGT6.1.A	90.19	monomer	-	AFDB search	AlphaFold v2	NA	0.57	0.43	Plectin isoform 1a
5dzz.1.A	51.24	monomer	-	HHblits	X-ray	2.60Å	0.45	0.18	Desmoplakin
5dzz.1.A	54.32	monomer	-	HHblits	X-ray	2.60Å	0.45	0.18	Desmoplakin
5dzz.1.A	51.48	monomer	-	BLAST	X-ray	2.60Å	0.45	0.17	Desmoplakin
5dzz.1.A	47.22	monomer	-	HHblits	X-ray	2.60Å	0.42	0.18	Desmoplakin
5dzz.1.A	38.27	monomer	-	HHblits	X-ray	2.60Å	0.38	0.18	Desmoplakin
1lm7.1.A	63.75	homo-dimer	0.08	HHblits	X-ray	3.00Å	0.48	0.09	subdomain of Desmoplakin Carboxy-Terminal domain (DPCT)
1lm7.1.A	65.56	homo-dimer	0.09	HHblits	X-ray	3.00Å	0.50	0.09	subdomain of Desmoplakin Carboxy-Terminal domain (DPCT)
2n03.1.A	99.02	monomer	-	BLAST	NMR	NA	0.61	0.07	Plectin
2n03.1.A	99.01	monomer	-	HHblits	NMR	NA	0.61	0.07	Plectin
1lm7.1.A	64.66	homo-dimer	0.09	BLAST	X-ray	3.00Å	0.50	0.08	subdomain of Desmoplakin Carboxy-Terminal domain (DPCT)
1lm7.1.A	62.34	homo-dimer	0.07	BLAST	X-ray	3.00Å	0.47	0.09	subdomain of Desmoplakin Carboxy-Terminal domain (DPCT)
5dzz.1.A	61.34	monomer	-	BLAST	X-ray	2.60Å	0.47	0.09	Desmoplakin
5dzz.1.A	47.54	monomer	-	BLAST	X-ray	2.60Å	0.43	0.09	Desmoplakin

Template	Seq Identity	Oligo-state	QSQE	Found by	Method	Resolution	Seq Similarity	Coverage	Description
1lm7.1.A	60.58	homo-dimer	0.07	BLAST	X-ray	3.00Å	0.47	0.09	subdomain of Desmoplakin Carboxy-Terminal domain (DPCT)
5dzz.1.A	44.92	monomer	-	BLAST	X-ray	2.60Å	0.42	0.09	Desmoplakin
1lm5.1.B	62.74	homo-dimer	0.08	HHblits	X-ray	1.80Å	0.49	0.08	subdomain of Desmoplakin Carboxy-Terminal domain (DPCT)
1lm7.1.A	47.93	homo-dimer	-	BLAST	X-ray	3.00Å	0.43	0.09	subdomain of Desmoplakin Carboxy-Terminal domain (DPCT)
1lm7.1.A	51.87	homo-dimer	-	HHblits	X-ray	3.00Å	0.44	0.09	subdomain of Desmoplakin Carboxy-Terminal domain (DPCT)
5dzz.1.A	45.93	monomer	-	BLAST	X-ray	2.60Å	0.41	0.10	Desmoplakin
1lm5.1.A	62.74	homo-dimer	0.08	HHblits	X-ray	1.80Å	0.49	0.08	subdomain of Desmoplakin Carboxy-Terminal domain (DPCT)
1lm5.1.B	62.81	homo-dimer	0.09	BLAST	X-ray	1.80Å	0.49	0.07	subdomain of Desmoplakin Carboxy-Terminal domain (DPCT)
1lm7.1.A	52.08	homo-dimer	-	BLAST	X-ray	3.00Å	0.44	0.09	subdomain of Desmoplakin Carboxy-Terminal domain (DPCT)
1lm5.1.A	62.81	homo-dimer	0.09	BLAST	X-ray	1.80Å	0.49	0.07	subdomain of Desmoplakin Carboxy-Terminal domain (DPCT)
5dzz.1.A	45.24	monomer	-	BLAST	X-ray	2.60Å	0.40	0.17	Desmoplakin
4qmd.1.A	38.62	monomer	-	HHblits	X-ray	1.60Å	0.38	0.07	Envoplakin
5dzz.1.A	42.68	monomer	-	BLAST	X-ray	2.60Å	0.40	0.09	Desmoplakin
5dzz.1.A	37.12	monomer	-	BLAST	X-ray	2.60Å	0.38	0.17	Desmoplakin
4qmd.1.A	39.46	monomer	-	BLAST	X-ray	1.60Å	0.39	0.07	Envoplakin
4qmd.2.A	38.62	monomer	-	HHblits	X-ray	1.60Å	0.38	0.07	Envoplakin
4qmd.1.A	47.09	monomer	-	HHblits	X-ray	1.60Å	0.42	0.07	Envoplakin
4qmd.1.A	37.30	monomer	-	BLAST	X-ray	1.60Å	0.38	0.07	Envoplakin
4qmd.1.A	37.37	monomer	-	HHblits	X-ray	1.60Å	0.38	0.07	Envoplakin
4qmd.2.A	39.46	monomer	-	BLAST	X-ray	1.60Å	0.39	0.07	Envoplakin
4qmd.1.A	46.63	monomer	-	BLAST	X-ray	1.60Å	0.42	0.07	Envoplakin
4qmd.1.A	37.63	monomer	-	BLAST	X-ray	1.60Å	0.38	0.07	Envoplakin
5dzz.1.A	41.31	monomer	-	BLAST	X-ray	2.60Å	0.39	0.17	Desmoplakin
4qmd.2.A	47.09	monomer	-	HHblits	X-ray	1.60Å	0.42	0.07	Envoplakin
4qmd.2.A	46.63	monomer	-	BLAST	X-ray	1.60Å	0.42	0.07	Envoplakin
4qmd.1.A	37.10	monomer	-	BLAST	X-ray	1.60Å	0.38	0.07	Envoplakin
4qmd.2.A	37.37	monomer	-	HHblits	X-ray	1.60Å	0.38	0.07	Envoplakin
4qmd.2.A	37.63	monomer	-	BLAST	X-ray	1.60Å	0.38	0.07	Envoplakin
4qmd.2.A	37.10	monomer	-	BLAST	X-ray	1.60Å	0.38	0.07	Envoplakin
1lm5.1.A	29.95	homo-dimer	-	HHblits	X-ray	1.80Å	0.35	0.07	subdomain of Desmoplakin Carboxy-Terminal domain (DPCT)
1lm5.1.B	29.95	homo-dimer	-	HHblits	X-ray	1.80Å	0.35	0.07	subdomain of Desmoplakin Carboxy-Terminal domain (DPCT)
1lm5.1.B	27.84	homo-dimer	-	BLAST	X-ray	1.80Å	0.36	0.07	subdomain of Desmoplakin Carboxy-Terminal domain (DPCT)
2n03.1.A	35.53	monomer	-	HHblits	NMR	NA	0.36	0.07	Plectin
1lm5.1.A	27.84	homo-dimer	-	BLAST	X-ray	1.80Å	0.36	0.07	subdomain of Desmoplakin Carboxy-Terminal domain (DPCT)
1lm7.1.A	32.07	homo-dimer	-	HHblits	X-ray	3.00Å	0.35	0.09	subdomain of Desmoplakin Carboxy-Terminal domain (DPCT)

Template	Seq Identity	Oligo-state	QSQE	Found by	Method	Resolution	Seq Similarity	Coverage	Description
5dzz.1.A	35.94	monomer	-	BLAST	X-ray	2.60Å	0.37	0.08	Desmoplakin

The table above shows the top 50 filtered templates. A further 544 templates were found which were considered to be less suitable for modelling than the filtered list.
1d7m.1.A, 1d7m.1.B, 1lm5.1.A, 1lm5.1.B, 1lm7.1.A, 2b9c.1.A, 2b9c.1.B, 2d3e.1.A, 2d3e.1.B, 2e7s.1.A, 2e7s.1.B, 2e7s.10.A, 2e7s.10.B, 2e7s.2.A, 2e7s.2.B, 2e7s.3.A, 2e7s.3.B, 2e7s.4.A, 2e7s.4.B, 2e7s.5.A, 2e7s.5.B, 2e7s.6.A, 2e7s.6.B, 2e7s.7.A, 2e7s.7.B, 2e7s.8.A, 2e7s.8.B, 2e7s.9.A, 2e7s.9.B, 2efr.1.A, 2efr.1.B, 2efs.1.A, 2efs.1.B, 2fxm.1.A, 2fxm.1.B, 2fxo.1.A, 2fxo.1.B, 2fxo.2.A, 2fxo.2.B, 2i1k.1.A, 2n03.1.A, 2ocy.1.A, 2ocy.1.B, 2oto.1.A, 2oto.1.B, 2oto.2.A, 2oto.2.B, 2v66.1.A, 2v66.1.B, 2v66.1.C, 2v66.1.D, 2w4u.1.K, 2w4u.1.L, 3cl3.1.C, 3cl3.1.D, 3dtp.1.A, 3dtp.1.B, 3jax.1.A, 3jax.1.B, 3o0z.1.A, 3o0z.1.B, 3o0z.2.A, 3o0z.2.B, 3oja.1.B, 3vkg.1.A, 3vkg.1.B, 3vkh.1.A, 3vkh.2.A, 4a7f.1.B, 4a7f.1.H, 4gkw.1.A, 4gkw.1.B, 4ll8.1.B, 4ll8.1.C, 4mh6.1.A, 4q28.1.A, 4qmd.1.A, 4qmd.2.A, 4rfx.1.A, 4rfx.1.B, 4rfx.2.A, 4rfx.2.B, 4rfx.3.A, 4rh7.1.A, 4rm8.1.A, 4rm8.2.A, 4whe.1.A, 4xa1.1.A, 4xa1.1.B, 4xa1.2.A, 4xa1.2.B, 4xa3.1.A, 4xa3.1.B, 4xa6.1.A, 4xa6.1.B, 4xa6.2.A, 4xa6.2.B, 5cj0.1.A, 5cj0.1.B, 5cj1.1.A, 5cj1.1.B, 5cj1.2.A, 5cj1.2.B, 5cj1.3.A, 5cj1.3.B, 5cj1.4.A, 5cj1.4.B, 5dzz.1.A, 5ijn.1.F, 5ijn.1.G, 5ijn.1.H, 5ijn.1.L, 5ijn.1.R, 5nen.1.A, 5nen.1.B, 5nug.1.A, 5to5.1.A, 5to5.1.B, 5to6.1.A, 5to6.1.B, 5to6.1.C, 5to6.1.D, 5to7.1.A, 5to7.1.B, 5to7.1.C, 5to7.1.D, 5tvb.1.A, 5tvb.1.B, 5wjb.1.A, 5wjb.1.B, 5wlq.1.A, 5wlz.1.A, 5wlz.1.B, 5wlz.1.C, 5wlz.1.D, 5y05.1.A, 6f1t.1.U, 6f1t.1.h, 6h2x.1.A, 6ian.1.A, 6ian.1.B, 6ian.2.A, 6ian.2.B, 6ixv.1.A, 6ixv.2.A, 6ixv.3.A, 6ixv.4.A, 6jlb.1.A, 6jlb.1.B, 6jlb.1.C, 6jlb.1.D, 6l5j.1.A, 6l5j.1.B, 6l5j.2.A, 6l5j.2.B, 6mi3.1.A, 6mi3.1.B, 6mi4.1.A, 6mi4.1.B, 6pf2.1.A, 6pf2.1.B, 6pfp.1.A, 6pfp.1.B, 6pfp.2.A, 6pfp.2.B, 6snz.1.A, 6snz.1.B, 6snz.2.A, 6snz.2.B, 6u42.439.A, 6u42.440.A, 6vzf.1.A, 6yrl.1.A, 6yrl.1.B, 6yrl.1.C, 6yrl.1.D, 6yrn.1.A, 6yrn.1.B, 6yrn.1.C, 6yrn.1.D, 6yrn.2.A, 6yrn.2.B, 6yrn.2.C, 6yrn.2.D, 6ys4.1.A, 6ys4.1.B, 6ys4.2.A, 6ys4.2.B, 6ys4.3.A, 6ys4.3.B, 6z26.1.A, 6z26.1.B, 6z26.2.A, 6z26.2.B, 6z6f.1.C, 6z6f.1.D, 6z6h.1.C, 6z6h.1.D, 6z6o.1.C, 6z6o.1.D, 6z6p.1.C, 6z6p.1.D, 6z9l.1.A, 6zbc.1.A, 6zbe.1.A, 6znm.1.H, 6znm.1.J, 6zpm.1.A, 6zpm.1.B, 6zyw.1.B, 6zyw.1.C, 7abk.1.A, 7d9n.1.A, 7d9n.1.B, 7dl2.1.A, 7dl2.1.D, 7ju4.1.A, 7ju4.1.E, 7ju4.1.J, 7ju4.1.K, 7k58.1.A, 7k58.1.B, 7k58.1.D, 7k5b.1.A, 7k5b.1.B, 7k5b.1.C, 7kek.1.A, 7kek.1.D, 7ko4.1.P, 7ko4.1.Q, 7ko5.1.P, 7ko5.1.Q, 7kog.1.A, 7kog.1.B, 7kzm.1.6, 7kzm.1.O, 7kzm.1.Q, 7moq.1.C, 7moq.1.X, 7moq.1.Z, 7n85.1.E, 7n85.1.H, 7n85.1.K, 7n85.1.N, 7n9f.1.4, 7n9f.1.5, 7n9f.1.8, 7n9f.1.9, 7n9f.1.E, 7n9f.1.H, 7n9f.1.K, 7n9f.1.N, 7nyw.1.B, 7nyx.1.A, 7nyx.1.B, 7nyy.1.A, 7nyy.1.B, 7nyz.1.B, 7nz0.1.A, 7nz0.1.B, 7nz3.1.A, 7o3w.1.A, 7o3w.1.B, 7o3w.1.C, 7o3w.1.D, 7o3w.1.E, 7o3w.1.F, 7o3x.1.A, 7o3x.1.B, 7o3x.1.C, 7o3x.1.D, 7o3x.1.E, 7o3x.1.F, 7o3y.1.A, 7o3y.1.B, 7o3y.1.C, 7o3y.1.E, 7o3y.1.F, 7o3z.1.A, 7o3z.1.B, 7o3z.1.C, 7o3z.1.D, 7o3z.1.E, 7o3z.1.F, 7o40.1.A, 7o40.1.B, 7o40.1.C, 7o40.1.D, 7o40.1.E, 7o40.1.F, 7ogt.1.B, 7per.1.G, 7per.1.J, 7qcd.1.A, 7qcd.1.B, 7r5k.43.A, 7r5k.49.A, 7r5k.53.A, 7r5k.54.A, 7r5k.94.A, 7r5k.95.A, 7rro.121.A, 7rro.122.A, 7rro.123.A, 7rro.153.A, 7rro.154.A, 7rro.156.A, 7rro.157.A, 7rro.158.A, 7rro.56.A, 7rro.57.A, 7rro.58.A, 7rro.61.A, 7rro.62.A, 7rro.63.A, 7tbi.50.A, 7tbi.51.A, 7tdz.1.3, 7tdz.1.5, 7tve.1.D, 7ung.57.A, 7ung.58.A, 7ung.59.A, 7uti.1.Q, 7uti.1.R, 7utl.1.S, 7utl.1.T, 7uw5.1.A, 7ux1.1.A, 7vf2.1.C, 7vf2.1.D, 7vf5.1.B, 7vf5.1.C, 7vop.1.0, 7vop.1.2, 7vop.1.4, 7vop.1.5, 7wkk.1.G, 7wkk.1.I, 7wkk.1.J, 7wkk.1.L, 7wkk.1.U, 7wkk.1.X, 7woo.1.H, 7wot.1.W, 7yg4.1.B, 7yg4.1.C, 7z8f.1.0, 7z8f.1.7, 7z8f.1.8, 7z8f.1.Z, 7zr1.1.C, 7zr1.1.D, 8aud.1.A, 8bd7.1.G, 8bwy.1.B, 8bwy.1.C, 8bx8.1.A, 8bx8.1.B, 8dd0.1.G, 8dd0.1.H, 8dzz.1.A, 8g4l.100.A, 8g4l.101.A, 8g4l.102.A, 8g4l.104.A, 8g4l.118.A, 8g4l.17.A, 8g4l.18.A, 8g4l.19.A, 8g4l.20.A, 8g4l.21.A, 8g4l.22.A, 8g4l.35.A, 8g4l.36.A, 8g4l.58.A, 8g4l.59.A, 8g4l.60.A, 8g4l.61.A, 8g4l.62.A, 8g4l.63.A, 8g4l.76.A, 8g4l.77.A, 8g4l.99.A, 8glv.195.A, 8glv.215.A, 8glv.216.A, 8glv.217.A, 8glv.218.A, 8glv.346.A, 8glv.347.A, 8glv.357.A, 8glv.358.A, 8glv.359.A, 8glv.360.A, 8glv.420.A, 8glv.421.A, 8glv.455.A, 8glv.456.A, 8glv.492.A, 8glv.498.A, 8glv.530.A, 8glv.531.A, 8glv.542.A, 8glv.554.A, 8glv.602.A, 8glv.603.A, 8glv.756.A, 8glv.844.A, 8glv.854.A, 8glv.891.A, 8glv.902.A, 8glv.913.A, 8glv.953.A, 8glv.959.A, 8glv.960.A, 8glv.962.A, 8glv.966.A, 8i7o.47.A, 8i7o.48.A, 8i7o.49.A, 8i7o.56.A, 8i7o.57.A, 8i7o.59.A, 8i7r.107.A, 8i7r.109.A, 8i7r.110.A, 8i7r.111.A, 8i7r.112.A, 8i7r.113.A, 8i7r.114.A, 8i7r.115.A, 8i7r.128.A, 8i7r.129.A, 8i7r.130.A, 8i7r.132.A, 8i7r.133.A, 8i7r.134.A, 8i7r.135.A, 8i7r.136.A, 8i7r.137.A, 8i7r.138.A, 8i7r.139.A, 8i7r.140.A, 8iah.1.5, 8iah.1.6, 8iah.1.7, 8iai.1.2, 8iyj.424.A, 8iyj.425.A, 8iyj.426.A, 8iyj.427.A, 8iyj.428.A, 8iyj.429.A, 8iyj.430.A, 8iyj.431.A, 8iyj.432.A, 8iyj.433.A, 8iyj.450.A, 8iyj.451.A, 8iyj.452.A, 8iyj.453.A, 8iyj.454.A, 8iyj.455.A, 8iyj.456.A, 8iyj.457.A, 8iyj.458.A, 8iyj.459.A, 8iyj.476.A, 8iyj.477.A, 8iyj.478.A, 8iyj.479.A, 8iyj.480.A, 8iyj.481.A, 8j07.1.A, 8j07.163.A, 8j07.2.A, 8j07.78.A, 8j07.844.A, 8j07.848.A, 8j07.849.A, 8j07.851.A, 8j07.852.A, 8j07.853.A, 8j07.854.A, 8j07.859.A, 8j07.860.A, 8j07.861.A, 8j07.863.A, 8j07.864.A, 8j07.868.A, 8j07.876.A, 8j07.877.A, 8j07.894.A, 8j07.896.A, 8j07.899.A, 8j07.908.A, 8j07.916.A, 8j07.935.A, 8j07.937.A, 8j07.954.A, 8j07.956.A, 8j07.963.A, 8j07.965.A, 8p4y.1.A, 8q4g.1.G, 8q4g.1.H, 8q6t.1.A, 8q6t.1.B, 8q6t.1.H, 8q6t.1.N, 8q6t.1.O, 8q6t.1.Q, 8srq.1.H, 8tek.1.D, 8tek.1.F, 8tek.1.H, 8th8.1.A, 8th8.1.D, 8th8.1.E, 8th8.1.K, 8th8.1.P, 8th8.1.R, 8tid.1.2, 8tid.1.3, 8tid.1.A, 8tid.1.D, 8tid.1.E, 8tid.1.K, 8tid.1.N, 8tid.1.P, 8tid.1.S, 8tj5.1.U, 8tj5.1.b, 8to0.397.A, 8to0.398.A, 8to0.400.A, 8to0.409.A, 8to0.410.A, 8to0.412.A, 8to0.415.A, 8tvl.1.A, 8u95.1.A, 8u95.1.B
