# Supplementary material for: Effects of Copy Number Variations in the Plectin (PLEC) Gene on the Growth Traits and Meat Quality of Leizhou Black Goats
Source: Animals (Basel). 2023 Nov 25;13(23):3651. doi: 10.3390/ani13233651 (PMC10705363; doi:10.3390/ani13233651)
Supplement: Supplementary file 1 [file animals-13-03651-s001.zip › Supplementary Material S2 swissmodel-PLEC-CDS Report.pdf]

SWISS-MODEL Homology Modelling Report

Model Building Report

This document lists the results for the homology modelling project "PLEC-cds" submitted to SWISS-MODEL workspace on Nov. 16, 2023, 10:47 a.m..The submitted primary amino acid sequence is given in Table T1.

If you use any results in your research, please cite the relevant publications:

- Waterhouse, A., Bertoni, M., Bienert, S., Studer, G., Tauriello, G., Gumienny, R., Heer, F.T., de Beer, T.A.P., Rempfer, C., Bordoli, L., Lepore, R., Schwede, T. SWISS-MODEL: homology modelling of protein structures and complexes. Nucleic Acids Res. 46(W1), W296-W303 (2018).
- Bienert, S., Waterhouse, A., de Beer, T.A.P., Tauriello, G., Studer, G., Bordoli, L., Schwede, T. The SWISS-MODEL Repository - new features and functionality. Nucleic Acids Res. 45, D313-D319 (2017).
- Studer, G., Tauriello, G., Bienert, S., Biasini, M., Johner, N., Schwede, T. ProMod3 - A versatile homology modelling toolbox. PLOS Comp. Biol. 17(1), e1008667 (2021).
- Studer, G., Rempfer, C., Waterhouse, A.M., Gumienny, G., Haas, J., Schwede, T. QMEANDisCo - distance constraints applied on model quality estimation. Bioinformatics 36, 1765-1771 (2020).
- Bertoni, M., Kiefer, F., Biasini, M., Bordoli, L., Schwede, T. Modeling protein quaternary structure of homo- and hetero-oligomers beyond binary interactions by homology. Scientific Reports 7 (2017).

Results

The SWISS-MODEL template library (SMTL version 2023-11-08, PDB release 2023-11-03) was searched with for evolutionary related structures matching the target sequence in Table T1. For details on the template search, see Materials and Methods. Overall 3124 templates were found (Table T2).

Models

The following models were built (see Materials and Methods "Model Building"):

Model #01	File	Built with	Oligo-State	Ligands	GMQE
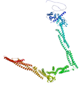	PDB	ProMod3 3.3.0	monomer	None	0.22

Template	Seq Identity	Oligo-state	QSQE	Found by	Method	Resolution	Seq Similarity	Range	Coverage	Description
H9F0F3.1.A	94.23	monomer	-	AFDB search	AlphaFold v2	-	0.59	1 - 1179	0.26	Plectin isoform 1c

The template contained no ligands.

```
Target    MSGEDQEVRAVVEDGSNGGSGSPSPGDTLPWNLEKTQRSRRGGGGPGGNGSVLDPAERAVIRIADERDRVQKKTFTKWVN
H9F0F3.1.AMSGEDAEVRAVPEDVSNGSSGSPSPGDTLPWNLGKTQRSRRSGGGAGSNGSVLDPAERAVIRIADERDRVQKKTFTKWVN

Target    KHLIKAQRHISDLYEDLRDGHNLISLLEVLSGDSLPREKGRMRFHKLQNVQIALDYLRHRQVKLVNIRNDDIADGNPKLT
H9F0F3.1.AKHLIKAQRHISDLYEDLRDGHNLISLLEVLSGDSLPREKGRMRFHKLQNVQIALDYLRHRQVKLVNIRNDDIADGNPKLT

Target    LGLIWTIILHFQISDIQVSGQSEDMTAKEKLLLWSQRMVEGYQGLRCDNFTSSWRDGRLFNAIIHRHKPMLIDMNKVYRQ
H9F0F3.1.ALGLIWTIILHFQISDIQVSGQSEDMTAKEKLLLWSQRMVEGYQGLRCDNFTSSWRDGRLFNAIIHRHKPMLIDMNKVYRQ

Target    TNLENLDQAFSVAERDLGVTRLLDPEDVDVPQPDEKSIITYVSSLYDAMPRVPDVQDGVKANELQLRWQEYRELVLLLLQ
H9F0F3.1.ATNLENLDQAFSVAERDLGVTRLLDPEDVDVPQPDEKSIITYVSSLYDAMPRVPDVQDGVRANELQLRWQEYRELVLLLLQ

Target    WIRAHTAAFEERRFPSSFEEIEILWCQFLKFKETELPAKEADKNRSKGIYQSLEGAVQAGQLKVPPGYHPLDVEKEWGKL
H9F0F3.1.AWIRHHTAAFEERRFPSSFEEIEILWSQFLKFKEMELPAKEADKNRSKGIYQSLEGAVQAGQLKVPPGYHPLDVEKEWGKL

Target    HVAILEREKQLRSEFERLERLQRIVSKLQMEAGLCEEQLNQADALLQSDVRLLAAGKAPQRAGEVERDLDKADGMIRLLF
H9F0F3.1.AHVAILEREKQLRSEFERLECLQRIVTKLQMEAGLCEEQLNQADTLLQSDVRLLAAGKVPQRAGEVERDLDKADSMIRLLF

Target    NDVQALKDGRHPQGEQMYRRVYRLHERLVAIRTEYNLRLR-G-----------T----PRHPELEDSTLRYLQDLLAWVE
H9F0F3.1.ANDVQTLKDGRHPQGEQMYRRVYRLHERLVAIRTEYNLRLKAGVAAPATQVTQVTLQSVQRRPELEDSTLRYLQDLLAWVE

Target    ENQRRVDSAEWGVDLPSVEAQLGSHRGLHQSVEEFRAKIERARTDEGQLSPATRGAYRDCLGRLDLQYAKLLNSSKARLR
H9F0F3.1.AENQHRVDGAEWGVDLPSVEAQLGSHRGLHQSIEEFRAKIERARSDEGQLSPATRGAYRDCLGRLDLQYAKLLNSSKARLR
```

```
Target    SLESLHGFVAAATKELMWLSEKEEEEVGFDWSERNSNMAAKKEAYSALMRELELKEKKIKEIQSTGDRLLREDHPARPTV
H9F0F3.1.ASLESLHSFVAAATKELMWLNEKEEEEVGFDWSDRNTNMTAKKESYSALMRELELKEKKIKELQSAGDRLLREDHPARPTV

Target    ESFQAALQTQWSWMLQLCCCIEAHLKENTAYFQFFSDVREAEEQLRKLQETLHRKYTCDRSITVTRLEDLLQDAQDEKDQ
H9F0F3.1.AESFQAALQTQWSWMLQLCCCIEAHLKENAAYFQFFSDVREAEGQLQKLQEALRRKYSCDRSATVTRLEDLLQDAQDEKEQ

Target    LNEYRGHLSGLAKRAKAIVQLTPRNPTQPTRGRVPLLAVCDYKQVEATVHKGDECQMLGPAQPFHWKVLSSSGSEAAVPS
H9F0F3.1.ALNEYKGHLSGLAKRAKAIVQLKPRHPAHPVRSRLPLLAVCDYKQVEVTVHKGDECQLVGPAQPSHWKVLSSSGSEAAVPS

Target    VCFLVPPPNQEALEAVARLEAQHQALVTLWHQLHTDMKSLLAWQSLSRDVQLIRSWSLVTFRTLKPEEQRQALRSLELHY
H9F0F3.1.AVCFLVPPPNQEAQEAVTRLEAQHQALVTLWHQLHVDMKSLLAWQSLRRDVQLIRSWSLATFRTLKPEEQRQALHSLELHY

Target    QAFLRDSQDAGGFGPEDRLQAEREYGSCSRHYQQLLQSLEQGEQEESRCQRCISELKDIRLQLEACETRTVHRLRLPLDK
H9F0F3.1.AQAFLRDSQDAGGFGPEDRLMAEREYGSCSHHYQQLLQSLEQGAQEESRCQRCISELKDIRLQLEACETRTVHRLRLPLDK

Target    EPARECAQRIAEQQKAQAEVEGLGKGVARLSAEAEKVLALPEPSPAAPTLRSELELTLGKLEQVRSLSAIYLEKLKTISL
H9F0F3.1.AEPARECAQRIAEQQKAQAEVEGLGKGVARLSAEAEKVLALPEPSPAAPTLRSELELTLGKLEQVRSLSAIYLEKLKTISL

Target    VIRSTQGAEEALRAHEEQLKEAQAVPAALPELEATKAAMKKLRAQAEAQQPVFDALRDELRGAQEVGERLQQRHGERDVE
H9F0F3.1.AVIHSTQGAEEVLRAHEEQLKEAQAVPATLPELEATKASLKKLRSQAEAQQPVFDALRDELRGAQEVGERLQQRHG-----

Target    VERWRERVTQLLERWQAVLAQTDVRQRELEQLGRQLRYYRESADPLGAWLQDARRRQEQIQAVPLADSQAVREQLRQEKA
H9F0F3.1.A--------------------------------------------------------------------------------

Target    LLEEIERHAEKVEECQRFAKQYINAIKDYELQLVTYKAQLEPVASPAKKPKVQSGSESVIQEYVDLRTRYSELSTLTSQY
H9F0F3.1.A--------------------------------------------------------------------------------

Target    IRFISETLRRMEEEERLAEQQRAEERERLAEVEAALEKQRQLAEAHAQAKAQAEREAQELQRRMQEEVARREEVAVDAQQ
H9F0F3.1.A--------------------------------------------------------------------------------

Target    QKRSIQEELQQLRQSSEAEIQAKARQVEAAERSRLRIEEEIRVVRLQLETTERQRGGAEGELQALRARAEEAEAQKRQAQ
H9F0F3.1.A--------------------------------------------------------------------------------

Target    EEAERLRRQVQEETQRKRQAEAELGLRVKAEAEAAREKQRALQALEELRLQAEEAERRLRQAEAERARQVQVALETAQRS
H9F0F3.1.A--------------------------------------------------------------------------------

Target    AQAELQSKHASFAEKTAQLERTLEEEHVTVVQLREEATRREQQQAEAERAREEAERELERWQLKANEALRLRLQAEEVAQ
H9F0F3.1.A--------------------------------------------------------------------------------

Target    QKSLAQAEAEKQKEAAEREARRRGKAEEQAVRQRELAEQELERQRQLAEGTAQQRLAAEQELIRLRAETEQGEQQRQLLE
H9F0F3.1.A--------------------------------------------------------------------------------

Target    EELARLQSEAAAATQKRQELEAELAKVRAEMEVLLASKARAEEESRSSSEKSKQRLEAEAGRFRELAEEAARLRALAEEA
H9F0F3.1.A--------------------------------------------------------------------------------

Target    KRQRQLAEEDAARQRAEAERVLSEKLAAISEATRLKTEAEIALKEKEAENERLRRLAEDEAFQRRRLEEQAAQHKADIEE
H9F0F3.1.A--------------------------------------------------------------------------------

Target    RLAQLRKASESELERQKGLVEDTLRQRRQVEEEILALKASFEKAAAGKAELELELGRIRGNAEDTLRSKEQAEQEAARQR
H9F0F3.1.A--------------------------------------------------------------------------------

Target    QLAAEEERRRREAEERVQKSLAAEEEAARQRKAALEEVERLKAKVEEARRLRERAEHESVRQLQLAQEAAQKRLQAEEKA
H9F0F3.1.A--------------------------------------------------------------------------------

Target    HAFAVQQKEQELQQTLQQEQSVLEKLRSEAEAARRAAEEAEEARERAEREAAQSRQRVEEAERLKQAAEEQAQAQAQAQA
H9F0F3.1.A--------------------------------------------------------------------------------

Target    AAEKLRKEAEQEAARRAQAEQAALRQKQAADAEMEKHKKFAEQTLRQKAQVEQELTALRLKLEETDHQKSILDQELQRLK
H9F0F3.1.A--------------------------------------------------------------------------------

Target    AEVTEAARQRSQVEEELFSVRVQMEELGKLKARIEAENRALILRDKDNTQRLLQEEAEKMKQVAEEAARLSVAAQEAARL
H9F0F3.1.A--------------------------------------------------------------------------------

Target    RQLAEEDLAQQRALAEKMLKEKMQAVQEATRLKAEAELLQQQKELAQEQARRLQEDKEQMAQQLAQETQGFQRTLETERQ
H9F0F3.1.A--------------------------------------------------------------------------------

Target    RQLEMSAEAERLRLRVAEMSRAQARAEEDAQRFRKQAEEIGAKLHRTELATQEKVTLVQTLETQRQQSDRDADRLREAIA
H9F0F3.1.A--------------------------------------------------------------------------------

Target    ELEREKDKLKKEAELLQLKSEEMQTVQQEQLLQETQALQQSFLSEKDSLLQRERFIEEEKAKLERLFQDEVAKAQKLREE
H9F0F3.1.A--------------------------------------------------------------------------------

Target    QQRQQQQMQQEKQQLLASMEEARRRQHEAEEGVRRKQEELQLLEQQRQQQEQLLAEENRRLRERLEHLEEEHRAALAHSE
```

```
H9F0F3.1.A--------------------------------------------------------------------------------

Target     EITAAQAAATRALPNGQDAADGPAAEPEHAFEGLRQKVPAQQLQEAGILSTEELQRLAQGHTTVAELTQREDVRRYLQGH
H9F0F3.1.A--------------------------------------------------------------------------------

Target     SSIAGLLLKPANEKLTIYAALRRQLLSPGTALILLEAQAASGFLLDPVRNRRLTVTEAVKEGVVGPELHHKLLSAERAVT
H9F0F3.1.A--------------------------------------------------------------------------------

Target     GYKDPYTGEKISLFQAMKKDLIVREHGIRLLEAQIATGGVIDPVHSHRVPVDVAYHRGYFDEEMNRVLQDPSDDTKGFFD
H9F0F3.1.A--------------------------------------------------------------------------------

Target     PNTHENLTYLQLLERCVEDPETGLRLLPLTDQAAKGGELVYTDSEARDVFEKATVSAPFGKFQGKTVTIWELINSEYFTA
H9F0F3.1.A--------------------------------------------------------------------------------

Target     EQRRDLLRQFRTGKVTVEKIIKIVITVIEEHEQKGQLCFQGLRALVPAAELLESGIIDWDLFRQLQLGERSVQEVAEVEG
H9F0F3.1.A--------------------------------------------------------------------------------

Target     VRRALRGSGVIAGVWLEEARQKLSIYEALKKELLQPEAAVALLEAQAGTGHVIDPATSARLTVDEAVRAGLVGPELHEKL
H9F0F3.1.A--------------------------------------------------------------------------------

Target     LSAEKAVTGYKDPYSGQSVSLFQALKKGLIPREQGLRLLDAQLSTGGTVDPSKSHRLPLDVACARGYLDEETSTALSAPR
H9F0F3.1.A--------------------------------------------------------------------------------

Target     DDAKTYYDPRTWELATYSQLQQQCRPDPLTGLSLLPLSEEAARARQQELYSEVQAREAFQKATVEVPVGSFQGRAVTIWE
H9F0F3.1.A--------------------------------------------------------------------------------

Target     LINSEYFTAEQRQELLRQFRTGKVTVEKIIKIVITIVEEVETTRRERLSFSGLRAPVPASELLAAGILSSSQFEQLKDGK
H9F0F3.1.A--------------------------------------------------------------------------------

Target     TSVKDLSELSSLQTLLQGSGCLAGIYLEESKEKVTIYEAMRRGLLRPSTAILLLEAQAATGFLVDPVRNQRLYVHEAVKA
H9F0F3.1.A--------------------------------------------------------------------------------

Target     GVVGPELHEKLLSAEKAVTGYKDPYSGSTISLFQAMKKGLVVREHGIRLLEAQIATGGIIDPVHSHRVPVDVACQRGYFD
H9F0F3.1.A--------------------------------------------------------------------------------

Target     EEMNRVLEDPSDDTKGFFDPNTHENLTYRQLLERCVEDPETGLRLLPLKGPEKAEVVETTRVYTEEETRRAFEETQIDIP
H9F0F3.1.A--------------------------------------------------------------------------------

Target     GGGSHGGSTMSLWEVMQSDLIPEEQRAQLMADFQAGRVTKERMIIIIIEIIEKTEIVRQQNLASYDYVRRRLTAEDLYEA
H9F0F3.1.A--------------------------------------------------------------------------------

Target     RVISRESYSLLREGTRSLREVLEAESAWRYLYGTGCVAGVYLPGSRQTLPIYQALKKGLLSAEVARLLLEAQAATGFLLD
H9F0F3.1.A--------------------------------------------------------------------------------

Target     PVKGERLTVDEAVRKGLVGPELHDRLLSAERAVTGYRDPYTEQTISLFQAMKKDLIPAEEALRLLDAQLATGGIVDPRLG
H9F0F3.1.A--------------------------------------------------------------------------------

Target     FHLPLEVAYQRGYLNKDTHDQLSEPSEVRSYVDPSTDERLSYTQLLRRCRRDETSGLFLLPLSEARKLTFRGLRKQITVE
H9F0F3.1.A--------------------------------------------------------------------------------

Target     ELVRSHVMDEATAQRLQEGLTSIEEVSKNLQKFLEGTSSIAGVLVDATKERLSVYQAMKKGIIRPGTAFELLEAQAATGY
H9F0F3.1.A--------------------------------------------------------------------------------

Target     VIDPIKGLKLTVEEAVRMGIVGPEFKDKLLSAERAVTGYKDPYSGKLISLFQAMKKGLILKDHGIRLLEAQIATGGIIDP
H9F0F3.1.A--------------------------------------------------------------------------------

Target     EESHRLPVDVAYQRGLFDEEMNEILTDPSDDTKGFFDPNTEENLTYLQLMERCVTDPQTGLRLLPLKEKKRERKTSSKSS
H9F0F3.1.A--------------------------------------------------------------------------------

Target     VRKRRVVIVDPETGKEMSVYEAYRKGLIDHQTYLELSEQECEWEEITISSSDGVVKSMIIDRRSGRQYDIDEAIAKSLID
H9F0F3.1.A--------------------------------------------------------------------------------

Target     RSALDQYRAGTLSITEFADMLSGNAGGFRSRSSSVGSSSSYPISPAVSRTQLASWSDPTEETGPVAGILDTETLEKVSIT
H9F0F3.1.A--------------------------------------------------------------------------------

Target     EAMHRNLVDNITGQRLLEAQACTGGIIDPNTGERFPVTDAVNKGLVDKIMVDRINLAQKAFCGFEDPRTKTKMSAAQALK
H9F0F3.1.A--------------------------------------------------------------------------------

Target     KGWLYYEAGQRFLEVQYLTGGLIEPDTPGRVPLDEALQRGTVDARTAQKLRDVSAYSKYLTCPKTKLKISYKDALDRSMV
H9F0F3.1.A--------------------------------------------------------------------------------

Target     EEGTGLRLLEAAAQSSKGYYSPYSVSGSGSTTGSRSGSRTGSRAGSRRGSFDATGSGFSMTFSSSSYSSSGYGRRYASGP
H9F0F3.1.A--------------------------------------------------------------------------------
```

```
Target     TSSLGGPESAAA
H9F0F3.1.A------------
```

Model #06	File	Built with	Oligo-State	Ligands	GMQE	QMEANDisCo Global
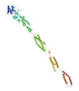	PDB	ProMod3 3.3.0	monomer (matching prediction)	None	0.00	0.48 ± 0.05

Template	Seq Identity	Oligo-state	QSQE	Found by	Method	Resolution	Seq Similarity	Range	Coverage	Description
1sjj.1.A	12.54	homo-dimer	0.00	HHblits	2DX	-	0.27	187 - 806	0.13	actinin

The template contained no ligands.

```
Target     MSGEDQEVRAVVEDGSNGGSGSPSPGDTLPWNLEKTQRSRRGGGGPGGNGSVLDPAERAVIRIADERDRVQKKTFTKWVN
1sjj.1.A   --------------------------------------------------------------------------------

Target     KHLIKAQRHISDLYEDLRDGHNLISLLEVLSGDSLPREKGRMRFHKLQNVQIALDYLRHRQVKLVNIRNDDIADGNPKLT
1sjj.1.A   --------------------------------------------------------------------------------

Target     LGLIWTIILHFQISDIQVSGQSEDMTAKEKLLLWSQRMVEGYQGLRCDNFTSSWRDGRLFNAIIHRHKPMLIDMN--KVY
1sjj.1.A   --------------------------QRKTFTAWCNSHLRKA-GTQIENIEEDFRDGLKLMLLLEVISGERLAKPERGKM

Target     RQTNLENLDQAFSVAERDLGVT-RLLDPEDVDVPQPDEKSIITYVSSLYDAMPRVPDVQDGVKANELQLRWQEYRELVLL
1sjj.1.A   RVHKISNVNKALDFIA-SKGVKLVSIGAEEIVDGNV--KMTLGMIWTIILRFAIQDISVE-------------ETSAKEG

Target     LLQWIRAHTAAFE---ERRFPSSFEEIEILWCQFLKFKETELP----AKEADKNRSKGIYQSLEGAVQAGQLKVPPGY--
1sjj.1.A   LLLWYQRKTAPYKNVNIQNFHISWKDGLGFCALIHRHRPELIDYGKLRKDDPLTNLNTAFDVAEKYLDIPKMLDAEDIVG

Target     -----------HPLDVEKEWGKLHVAILEREKQLRSEFERLERLQRIVSKLQMEAGLCEEQLNQADALLQSDVRL---LA
1sjj.1.A   TARPDEKAIMTYVSSFYHAFSGAQKAETAA-NRICKVLAVNQENEQLMEDYEKLASDLLEWIRRTIPWLENRAPENTMQA

Target     AGKAPQRAGEVERDLDKADG----MIRLLFNDV-QALKD-GRHPQGEQMYRRVYRLHERLVAIRT---EYNLRLRGTPRH
1sjj.1.A   MQQKLEDFRDYRRLHKPPKVQEKCQLEINFNTLQTKLRLSNRPAFMPSEGKMVSDINNAWGGLEQAEKGYEEWLLNEIRR

Target     P----ELEDSTLRYLQDLLAWVEENQRRVDSAEWGV-DLPSVEAQLGSHRGLHQSVEEFRAKIERARTDEGQLS---PAT
1sjj.1.A   LERLDHLAEKFRQKASIHESWTDGKEAMLQQKDYETATLSEIKALLKKHEAFESDLAAHQDRVEQIAAIAQELNELDYYD

Target     RGAYRDCLGRLDLQYAKLLNSSKARLRSLES-----------LHGFVAAATKELMWLSEKEEEEVGFDWSERNSNMAAKK
1sjj.1.A   SPSVNARCQKICDQWDNLGALTQKRREALERTEKLLETIDQLYLEYAKRAAPFNNWMEGAMEDLQDTFIVHTIEEIQGLT

Target     EAYSALMRELELKEKKIKEI---QSTGDRLLREDH----PARPTVESFQAALQTQWSWMLQLCCCIEAHLKENTAY----
1sjj.1.A   TAHEQFKATLPDADKERQAILGIHNEVSKIVQTYHVNMAGTNPYTTITPQEINGKWEHVRQLVPRRDQALMEEHARQQQN

Target     ----FQFFSDVREAEEQLRKLQETLHRKYTCDRSITVTRLEDLLQDAQDEKDQLNEYRGHLSGLAKRAKAIVQLTPRNPT
1sjj.1.A   ERLRKQFGAQANVIGPWIQTKMEEIGRISI----EMHGTLEDQLNHLRQYEKSIVNYKPKIDQLEGDHQQIQEAL-----

Target     QPTRGRVPLLAVCDYKQVEATVHKGDECQMLGPAQPFHWKVLSSSGSEAAVPSVCFLVPPPNQEALEAVARLEAQHQALV
1sjj.1.A   --------------------------------------------------------------------------------

Target     TLWHQLHTDMKSLLAWQSLSRDVQLIRSWSLVTFRTLKPEEQRQALRSLELHYQAFLRDSQDAGGFGPEDRLQAEREYGS
1sjj.1.A   --------------------------------------------------------------------------------

Target     CSRHYQQLLQSLEQGEQEESRCQRCISELKDIRLQLEACETRTVHRLRLPLDKEPARECAQRIAEQQKAQAEVEGLGKGV
1sjj.1.A   --------------------------------------------------------------------------------

Target     ARLSAEAEKVLALPEPSPAAPTLRSELELTLGKLEQVRSLSAIYLEKLKTISLVIRSTQGAEEALRAHEEQLKEAQAVPA
1sjj.1.A   --------------------------------------------------------------------------------

Target     ALPELEATKAAMKKLRAQAEAQQPVFDALRDELRGAQEVGERLQQRHGERDVEVERWRERVTQLLERWQAVLAQTDVRQR
1sjj.1.A   --------------------------------------------------------------------------------

Target     ELEQLGRQLRYYRESADPLGAWLQDARRRQEQIQAVPLADSQAVREQLRQEKALLEEIERHAEKVEECQRFAKQYINAIK
1sjj.1.A   --------------------------------------------------------------------------------
```

```
Target     DYELQLVTYKAQLEPVASPAKKPKVQSGSESVIQEYVDLRTRYSELSTLTSQYIRFISETLRRMEEEERLAEQQRAEERE
1sjj.1.A   --------------------------------------------------------------------------------

Target     RLAEVEAALEKQRQLAEAHAQAKAQAEREAQELQRRMQEEVARREEVAVDAQQQKRSIQEELQQLRQSSEAEIQAKARQV
1sjj.1.A   --------------------------------------------------------------------------------

Target     EAAERSRLRIEEEIRVVRLQLETTERQRGGAEGELQALRARAEEAEAQKRQAQEEAERLRRQVQEETQRKRQAEAELGLR
1sjj.1.A   --------------------------------------------------------------------------------

Target     VKAEAEAAREKQRALQALEELRLQAEEAERRLRQAEAERARQVQVALETAQRSAQAELQSKHASFAEKTAQLERTLEEEH
1sjj.1.A   --------------------------------------------------------------------------------

Target     VTVVQLREEATRREQQQAEAERAREEAERELERWQLKANEALRLRLQAEEVAQQKSLAQAEAEKQKEAAEREARRRGKAE
1sjj.1.A   --------------------------------------------------------------------------------

Target     EQAVRQRELAEQELERQRQLAEGTAQQRLAAEQELIRLRAETEQGEQQRQLLEEELARLQSEAAAATQKRQELEAELAKV
1sjj.1.A   --------------------------------------------------------------------------------

Target     RAEMEVLLASKARAEEESRSSSEKSKQRLEAEAGRFRELAEEAARLRALAEEAKRQRQLAEEDAARQRAEAERVLSEKLA
1sjj.1.A   --------------------------------------------------------------------------------

Target     AISEATRLKTEAEIALKEKEAENERLRRLAEDEAFQRRRLEEQAAQHKADIEERLAQLRKASESELERQKGLVEDTLRQR
1sjj.1.A   --------------------------------------------------------------------------------

Target     RQVEEEILALKASFEKAAAGKAELELELGRIRGNAEDTLRSKEQAEQEAARQRQLAAEEERRRREAEERVQKSLAAEEEA
1sjj.1.A   --------------------------------------------------------------------------------

Target     ARQRKAALEEVERLKAKVEEARRLRERAEHESVRQLQLAQEAAQKRLQAEEKAHAFAVQQKEQELQQTLQQEQSVLEKLR
1sjj.1.A   --------------------------------------------------------------------------------

Target     SEAEAARRAAEEAEEARERAEREAAQSRQRVEEAERLKQAAEEQAQAQAQAQAAAEKLRKEAEQEAARRAQAEQAALRQK
1sjj.1.A   --------------------------------------------------------------------------------

Target     QAADAEMEKHKKFAEQTLRQKAQVEQELTALRLKLEETDHQKSILDQELQRLKAEVTEAARQRSQVEEELFSVRVQMEEL
1sjj.1.A   --------------------------------------------------------------------------------

Target     GKLKARIEAENRALILRDKDNTQRLLQEEAEKMKQVAEEAARLSVAAQEAARLRQLAEEDLAQQRALAEKMLKEKMQAVQ
1sjj.1.A   --------------------------------------------------------------------------------

Target     EATRLKAEAELLQQQKELAQEQARRLQEDKEQMAQQLAQETQGFQRTLETERQRQLEMSAEAERLRLRVAEMSRAQARAE
1sjj.1.A   --------------------------------------------------------------------------------

Target     EDAQRFRKQAEEIGAKLHRTELATQEKVTLVQTLETQRQQSDRDADRLREAIAELEREKDKLKKEAELLQLKSEEMQTVQ
1sjj.1.A   --------------------------------------------------------------------------------

Target     QEQLLQETQALQQSFLSEKDSLLQRERFIEEEKAKLERLFQDEVAKAQKLREEQQRQQQMQQEKQQLLASMEEARRRQH
1sjj.1.A   --------------------------------------------------------------------------------

Target     EAEEGVRRKQEELQLLEQQRQQQEQLLAEENRRLRERLEHLEEEHRAALAHSEEITAAQAAATRALPNGQDAADGPAAEP
1sjj.1.A   --------------------------------------------------------------------------------

Target     EHAFEGLRQKVPAQQLQEAGILSTEELQRLAQGHTTVAELTQREDVRRYLQGHSSIAGLLLKPANEKLTIYAALRRQLLS
1sjj.1.A   --------------------------------------------------------------------------------

Target     PGTALILLEAQAASGFLLDPVRNRRLTVTEAVKEGVVGPELHHKLLSAERAVTGYKDPYTGEKISLFQAMKKDLIVREHG
1sjj.1.A   --------------------------------------------------------------------------------

Target     IRLLEAQIATGGVIDPVHSHRVPVDVAYHRGYFDEEMNRVLQDPSDDTKGFFDPNTHENLTYLQLLERCVEDPETGLRLL
1sjj.1.A   --------------------------------------------------------------------------------

Target     PLTDQAAKGGELVYTDSEARDVFEKATVSAPFGKFQGKTVTIWELINSEYFTAEQRRDLLRQFRTGKVTVEKIIKIVITV
1sjj.1.A   --------------------------------------------------------------------------------

Target     IEEHEQKGQLCFQGLRALVPAAELLESGIIDWDLFRQLQLGERSVQEVAEVEGVRRALRGSGVIAGVWLEEARQKLSIYE
1sjj.1.A   --------------------------------------------------------------------------------

Target     ALKKELLQPEAAVALLEAQAGTGHVIDPATSARLTVDEAVRAGLVGPELHEKLLSAEKAVTGYKDPYSGQSVSLFQALKK
1sjj.1.A   --------------------------------------------------------------------------------

Target     GLIPREQGLRLLDAQLSTGGTVDPSKSHRLPLDVACARGYLDEETSTALSAPRDDAKTYYDPRTWELATYSQLQQQCRPD
1sjj.1.A   --------------------------------------------------------------------------------

Target     PLTGLSLLPLSEEAARARQQELYSEVQAREAFQKATVEVPVGSFQGRAVTIWELINSEYFTAEQRQELLRQFRTGKVTVE
1sjj.1.A   --------------------------------------------------------------------------------
```

```
Target    KIIKIVITIVEEVETTRRERLSFSGLRAPVPASELLAAGILSSSQFEQLKDGKTSVKDLSELSSLQTLLQGSGCLAGIYL
1sjj.1.A  --------------------------------------------------------------------------------

Target    EESKEKVTIYEAMRRGLLRPSTAILLLEAQAATGFLVDPVRNQRLYVHEAVKAGVVGPELHEKLLSAEKAVTGYKDPYSG
1sjj.1.A  --------------------------------------------------------------------------------

Target    STISLFQAMKKGLVVREHGIRLLEAQIATGGIIDPVHSHRVPVDVACQRGYFDEEMNRVLEDPSDDTKGFFDPNTHENLT
1sjj.1.A  --------------------------------------------------------------------------------

Target    YRQLLERCVEDPETGLRLLPLKGPEKAEVVETTRVYTEEETRRAFEETQIDIPGGGSHGGSTMSLWEVMQSDLIPEEQRA
1sjj.1.A  --------------------------------------------------------------------------------

Target    QLMADFQAGRVTKERMIIIIIEIIEKTEIVRQQNLASYDYVRRRLTAEDLYEARVISRESYSLLREGTRSLREVLEAESA
1sjj.1.A  --------------------------------------------------------------------------------

Target    WRYLYGTGCVAGVYLPGSRQTLPIYQALKKGLLSAEVARLLLEAQAATGFLLDPVKGERLTVDEAVRKGLVGPELHDRLL
1sjj.1.A  --------------------------------------------------------------------------------

Target    SAERAVTGYRDPYTEQTISLFQAMKKDLIPAEEALRLLDAQLATGGIVDPRLGFHLPLEVAYQRGYLNKDTHDQLSEPSE
1sjj.1.A  --------------------------------------------------------------------------------

Target    VRSYVDPSTDERLSYTQLLRRCRRDETSGLFLLPLSEARKLTFRGLRKQITVEELVRSHVMDEATAQRLQEGLTSIEEVS
1sjj.1.A  --------------------------------------------------------------------------------

Target    KNLQKFLEGTSSIAGVLVDATKERLSVYQAMKKGIIRPGTAFELLEAQAATGYVIDPIKGLKLTVEEAVRMGIVGPEFKD
1sjj.1.A  --------------------------------------------------------------------------------

Target    KLLSAERAVTGYKDPYSGKLISLFQAMKKGLILKDHGIRLLEAQIATGGIIDPEESHRLPVDVAYQRGLFDEEMNEILTD
1sjj.1.A  --------------------------------------------------------------------------------

Target    PSDDTKGFFDPNTEENLTYLQLMERCVTDPQTGLRLLPLKEKKRERKTSSKSSVRKRRVVIVDPETGKEMSVYEAYRKGL
1sjj.1.A  --------------------------------------------------------------------------------

Target    IDHQTYLELSEQECEWEEITISSSDGVVKSMIIDRRSGRQYDIDEAIAKSLIDRSALDQYRAGTLSITEFADMLSGNAGG
1sjj.1.A  --------------------------------------------------------------------------------

Target    FRSRSSSVGSSSSYPISPAVSRTQLASWSDPTEETGPVAGILDTETLEKVSITEAMHRNLVDNITGQRLLEAQACTGGII
1sjj.1.A  --------------------------------------------------------------------------------

Target    DPNTGERFPVTDAVNKGLVDKIMVDRINLAQKAFCGFEDPRTKTKMSAAQALKKGWLYYEAGQRFLEVQYLTGGLIEPDT
1sjj.1.A  --------------------------------------------------------------------------------

Target    PGRVPLDEALQRGTVDARTAQKLRDVSAYSKYLTCPKTKLKISYKDALDRSMVEEGTGLRLLEAAAQSSKGYYSPYSVSG
1sjj.1.A  --------------------------------------------------------------------------------

Target    SGSTTGSRSGSRTGSRAGSRRGSFDATGSGFSMTFSSSSYSSSGYGRRYASGPTSSLGGPESAAA
1sjj.1.A  -----------------------------------------------------------------
```

Model #15	File	Built with	Oligo-State	Ligands	GMQE	QMEANDisCo Global
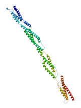	PDB	ProMod3 3.3.0	monomer (matching prediction)	None	0.00	0.55 ± 0.05

Template	Seq Identity	Oligo-state	QSQE	Found by	Method	Resolution	Seq Similarity	Range	Coverage	Description
1hci.1.A	14.35	homo-dimer	0.00	HHblits	X-ray	2.80Å	0.28	302 - 734	0.09	ALPHA-ACTININ 2

The template contained no ligands.

```
Target    MSGEDQEVRAVVEDGSNGGSGSPSPGDTLPWNLEKTQRSRRGGGGPGGNGSVLDPAERAVIRIADERDRVQKKTFTKWVN
1hci.1.A  --------------------------------------------------------------------------------

Target    KHLIKAQRHISDLYEDLRDGHNLISLLEVLSGDSLPREKGRMRFHKLQNVQIALDYLRHRQVKLVNIRNDDIADGNPKLT
1hci.1.A  --------------------------------------------------------------------------------
```

```
Target     LGLIWTIILHFQISDIQVSGQSEDMTAKEKLLLWSQRMVEGYQGLRCDNFTSSWRDGRLFNAIIHRHKPMLIDMNKVYRQ
1hci.1.A   --------------------------------------------------------------------------------

Target     TNLENLDQAFSVAERDLGVTRLLDPEDVDVPQPDEKSIITYVSSLYDAMPRVPDVQDGVKANELQLRWQEYRELVLLLLQ
1hci.1.A   -------------------------------------------------------------QENERLMEEYERLASELLE

Target     WIRAHTAAFEERRFPSSFEEIEILWCQFLKFKETELPAKEADKNRSKGIYQSLEGAVQAG---QLKVPPGYHPLDVEKEW
1hci.1.A   WIRRTIPWLENRTPEKTMQAMQKKLEDFRDYRRKHKPPKVQEKCQLEINFNTLQTKLRISNRPAFMPSEGKMVSDIAGAW

Target     GKLHVAILEREKQLRSEFERLERLQRIVSKLQMEAGLCEEQLNQADALLQSDV----RLLAAGKAPQRAGEVERDLDKAD
1hci.1.A   QRLEQAEKGYEEWLLNEIRRLERLEHLAEKFRQKASTHETWAYGKEQILLQKDYESASLTEVRALLRKHEAFESDLAAHQ

Target     GMIRLLFNDVQALKDGRHPQGEQMYRRVYRLHERLVAIRTEYNL---RLRGTPRHP-E---LEDSTLRYLQDLLAWVEEN
1hci.1.A   DRVEQIAAIAQELNELDYHDAVNVNDRCQKICDQWDRLGTLTQKRREALERMEKLLETIDQLHLEFAKRAAPFNNWMEGA

Target     QRRVDSAEWGVDLPSVEAQLGSHRGLHQSVEEFRAKI---ERARTDEGQ----LSP---ATRGAYRDCLGRLDLQYAKLL
1hci.1.A   MEDLQDMFIVHSIEEIQSLITAHEQFKATLPEADGERQSIMAIQNEVEKVIQSYNIRISSSNPYSTVTMDELRTKWDKVK

Target     NSSKARLRSLESL-----------HGFVAAATKELMWLSEKEEEEVGFDWSERNSNMAAKKEAYSALMRELELKEKKIKE
1hci.1.A   QLVPIRDQSLQEELARQHANERLRRQFAAQANAIGPWIQNKMEEIARSSIQI-TGALEDQMNQLKQYEHNIINYKNNIDK

Target     IQSTGDRLLREDHPARPTVESFQAALQTQWSWMLQLCCCIEAHLKENTAYFQFFSDVREAEEQLRKLQETLHRKYTCDRS
1hci.1.A   LEGDHQLIQEALVFDNKHTNYTMEHIRVGWELLLTTIARTINEVETQIL-------------------------------

Target     ITVTRLEDLLQDAQDEKDQLNEYRGHLSGLAKRAKAIVQLTPRNPTQPTRGRVPLLAVCDYKQVEATVHKGDECQMLGPA
1hci.1.A   --------------------------------------------------------------------------------

Target     QPFHWKVLSSSGSEAAVPSVCFLVPPPNQEALEAVARLEAQHQALVTLWHQLHTDMKSLLAWQSLSRDVQLIRSWSLVTF
1hci.1.A   --------------------------------------------------------------------------------

Target     RTLKPEEQRQALRSLELHYQAFLRDSQDAGGFGPEDRLQAEREYGSCSRHYQQLLQSLEQGEQEESRCQRCISELKDIRL
1hci.1.A   --------------------------------------------------------------------------------

Target     QLEACETRTVHRLRLPLDKEPARECAQRIAEQQKAQAEVEGLGKGVARLSAEAEKVLALPEPSPAAPTLRSELELTLGKL
1hci.1.A   --------------------------------------------------------------------------------

Target     EQVRSLSAIYLEKLKTISLVIRSTQGAEEALRAHEEQLKEAQAVPAALPELEATKAAMKKLRAQAEAQQPVFDALRDELR
1hci.1.A   --------------------------------------------------------------------------------

Target     GAQEVGERLQQRHGERDVEVERWRERVTQLLERWQAVLAQTDVRQRELEQLGRQLRYYRESADPLGAWLQDARRRQEQIQ
1hci.1.A   --------------------------------------------------------------------------------

Target     AVPLADSQAVREQLRQEKALLEEIERHAEKVEECQRFAKQYINAIKDYELQLVTYKAQLEPVASPAKKPKVQSGSESVIQ
1hci.1.A   --------------------------------------------------------------------------------

Target     EYVDLRTRYSELSTLTSQYIRFISETLRRMEEEERLAEQQRAEERERLAEVEAALEKQRQLAEAHAQAKAQAEREAQELQ
1hci.1.A   --------------------------------------------------------------------------------

Target     RRMQEEVARREEVAVDAQQQKRSIQEELQQLRQSSEAEIQAKARQVEAAERSRLRIEEEIRVVRLQLETTERQRGGAEGE
1hci.1.A   --------------------------------------------------------------------------------

Target     LQALRARAEEAEAQKRQAQEEAERLRRQVQEETQRKRQAEAELGLRVKAEAEAAREKQRALQALEELRLQAEEAERRLRQ
1hci.1.A   --------------------------------------------------------------------------------

Target     AEAERARQVQVALETAQRSAQAELQSKHASFAEKTAQLERTLEEEHVTVVQLREEATRREQQQAEAERAREEAERELERW
1hci.1.A   --------------------------------------------------------------------------------

Target     QLKANEALRLRLQAEEVAQQKSLAQAEAEKQKEAAEREARRRGKAEEQAVRQRELAEQELERQRQLAEGTAQQRLAAEQE
1hci.1.A   --------------------------------------------------------------------------------

Target     LIRLRAETEQGEQQRQLLEEELARLQSEAAAATQKRQELEAELAKVRAEMEVLLASKARAEEESRSSSEKSKQRLEAEAG
1hci.1.A   --------------------------------------------------------------------------------

Target     RFRELAEEAARLRALAEEAKRQRQLAEEDAARQRAEAERVLSEKLAAISEATRLKTEAEIALKEKEAENERLRRLAEDEA
1hci.1.A   --------------------------------------------------------------------------------

Target     FQRRRLEEQAAQHKADIEERLAQLRKASESELERQKGLVEDTLRQRRQVEEEILALKASFEKAAAGKAELELELGRIRGN
1hci.1.A   --------------------------------------------------------------------------------

Target     AEDTLRSKEQAEQEAARQRQLAAEEERRRREAEERVQKSLAAEEEAARQRKAALEEVERLKAKVEEARRLRERAEHESVR
1hci.1.A   --------------------------------------------------------------------------------

Target     QLQLAQEAAQKRLQAEEKAHAFAVQQKEQELQQTLQQEQSVLEKLRSEAEAARRAAEEAEEARERAEREAAQSRQRVEEA
```

```
1hci.1.A    --------------------------------------------------------------------------------

Target      ERLKQAAEEQAQAQAQAQAAAEKLRKEAEQEAARRAQAEQAALRQKQAADAEMEKHKKFAEQTLRQKAQVEQELTALRLK
1hci.1.A    --------------------------------------------------------------------------------

Target      LEETDHQKSILDQELQRLKAEVTEAARQRSQVEEELFSVRVQMEELGKLKARIEAENRALILRDKDNTQRLLQEEAEKMK
1hci.1.A    --------------------------------------------------------------------------------

Target      QVAEEAARLSVAAQEAARLRQLAEEDLAQQRALAEKMLKEKMQAVQEATRLKAEAELLQQQKELAQEQARRLQEDKEQMA
1hci.1.A    --------------------------------------------------------------------------------

Target      QQLAQETQGFQRTLETERQRQLEMSAEAERLRLRVAEMSRAQARAEEDAQRFRKQAEEIGAKLHRTELATQEKVTLVQTL
1hci.1.A    --------------------------------------------------------------------------------

Target      ETQRQQSDRDADRLREAIAELEREKDKLKKEAELLQLKSEEMQTVQQEQLLQETQALQQSFLSEKDSLLQRERFIEEEKA
1hci.1.A    --------------------------------------------------------------------------------

Target      KLERLFQDEVAKAQKLREEQQRQQQQMQQEKQQLLASMEEARRRQHEAEEGVRRKQEELQLLEQQRQQQEQLLAEENRRL
1hci.1.A    --------------------------------------------------------------------------------

Target      RERLEHLEEEHRAALAHSEEITAAQAAATRALPNGQDAADGPAAEPEHAFEGLRQKVPAQQLQEAGILSTEELQRLAQGH
1hci.1.A    --------------------------------------------------------------------------------

Target      TTVAELTQREDVRRYLQGHSSIAGLLLKPANEKLTIYAALRRQLLSPGTALILLEAQAASGFLLDPVRNRRLTVTEAVKE
1hci.1.A    --------------------------------------------------------------------------------

Target      GVVGPELHHKLLSAERAVTGYKDPYTGEKISLFQAMKKDLIVREHGIRLLEAQIATGGVIDPVHSHRVPVDVAYHRGYFD
1hci.1.A    --------------------------------------------------------------------------------

Target      EEMNRVLQDPSDDTKGFFDPNTHENLTYLQLLERCVEDPETGLRLLPLTDQAAKGGELVYTDSEARDVFEKATVSAPFGK
1hci.1.A    --------------------------------------------------------------------------------

Target      FQGKTVTIWELINSEYFTAEQRRDLLRQFRTGKVTVEKIIKIVITVIEEHEQKGQLCFQGLRALVPAAELLESGIIDWDL
1hci.1.A    --------------------------------------------------------------------------------

Target      FRQLQLGERSVQEVAEVEGVRRALRGSGVIAGVWLEEARQKLSIYEALKKELLQPEAAVALLEAQAGTGHVIDPATSARL
1hci.1.A    --------------------------------------------------------------------------------

Target      TVDEAVRAGLVGPELHEKLLSAEKAVTGYKDPYSGQSVSLFQALKKGLIPREQGLRLLDAQLSTGGTVDPSKSHRLPLDV
1hci.1.A    --------------------------------------------------------------------------------

Target      ACARGYLDEETSTALSAPRDDAKTYYDPRTWELATYSQLQQQCRPDPLTGLSLLPLSEEAARARQQELYSEVQAREAFQK
1hci.1.A    --------------------------------------------------------------------------------

Target      ATVEVPVGSFQGRAVTIWELINSEYFTAEQRQELLRQFRTGKVTVEKIIKIVITIVEEVETTRRERLSFSGLRAPVPASE
1hci.1.A    --------------------------------------------------------------------------------

Target      LLAAGILSSSQFEQLKDGKTSVKDLSELSSLQTLLQGSGCLAGIYLEESKEKVTIYEAMRRGLLRPSTAILLLEAQAATG
1hci.1.A    --------------------------------------------------------------------------------

Target      FLVDPVRNQRLYVHEAVKAGVVGPELHEKLLSAEKAVTGYKDPYSGSTISLFQAMKKGLVVREHGIRLLEAQIATGGIID
1hci.1.A    --------------------------------------------------------------------------------

Target      PVHSHRVPVDVACQRGYFDEEMNRVLEDPSDDTKGFFDPNTHENLTYRQLLERCVEDPETGLRLLPLKGPEKAEVVETTR
1hci.1.A    --------------------------------------------------------------------------------

Target      VYTEEETRRAFEETQIDIPGGGSHGGSTMSLWEVMQSDLIPEEQRAQLMADFQAGRVTKERMIIIIIEIIEKTEIVRQQN
1hci.1.A    --------------------------------------------------------------------------------

Target      LASYDYVRRRLTAEDLYEARVISRESYSLLREGTRSLREVLEAESAWRYLYGTGCVAGVYLPGSRQTLPIYQALKKGLLS
1hci.1.A    --------------------------------------------------------------------------------

Target      AEVARLLLEAQAATGFLLDPVKGERLTVDEAVRKGLVGPELHDRLLSAERAVTGYRDPYTEQTISLFQAMKKDLIPAEEA
1hci.1.A    --------------------------------------------------------------------------------

Target      LRLLDAQLATGGIVDPRLGFHLPLEVAYQRGYLNKDTHDQLSEPSEVRSYVDPSTDERLSYTQLLRRCRRDETSGLFLLP
1hci.1.A    --------------------------------------------------------------------------------

Target      LSEARKLTFRGLRKQITVEELVRSHVMDEATAQRLQEGLTSIEEVSKNLQKFLEGTSSIAGVLVDATKERLSVYQAMKKG
1hci.1.A    --------------------------------------------------------------------------------

Target      IIRPGTAFELLEAQAATGYVIDPIKGLKLTVEEAVRMGIVGPEFKDKLLSAERAVTGYKDPYSGKLISLFQAMKKGLILK
1hci.1.A    --------------------------------------------------------------------------------
```

```
Target    DHGIRLLEAQIATGGIIDPEESHRLPVDVAYQRGLFDEEMNEILTDPSDDTKGFFDPNTEENLTYLQLMERCVTDPQTGL
1hci.1.A  --------------------------------------------------------------------------------

Target    RLLPLKEKKRERKTSSKSSVRKRRVVIVDPETGKEMSVYEAYRKGLIDHQTYLELSEQECEWEEITISSSDGVVKSMIID
1hci.1.A  --------------------------------------------------------------------------------

Target    RRSGRQYDIDEAIAKSLIDRSALDQYRAGTLSITEFADMLSGNAGGFRSRSSSVGSSSSYPISPAVSRTQLASWSDPTEE
1hci.1.A  --------------------------------------------------------------------------------

Target    TGPVAGILDTETLEKVSITEAMHRNLVDNITGQRLLEAQACTGGIIDPNTGERFPVTDAVNKGLVDKIMVDRINLAQKAF
1hci.1.A  --------------------------------------------------------------------------------

Target    CGFEDPRTKTKMSAAQALKKGWLYYEAGQRFLEVQYLTGGLIEPDTPGRVPLDEALQRGTVDARTAQKLRDVSAYSKYLT
1hci.1.A  --------------------------------------------------------------------------------

Target    CPKTKLKISYKDALDRSMVEEGTGLRLLEAAAQSSKGYYSPYSVSGSGSTTGSRSGSRTGSRAGSRRGSFDATGSGFSMT
1hci.1.A  --------------------------------------------------------------------------------

Target    FSSSSYSSSGYGRRYASGPTSSLGGPESAAA
1hci.1.A  -------------------------------
```

Model #14	File	Built with	Oligo-State	Ligands	GMQE	QMEANDisCo Global
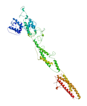	PDB	ProMod3 3.3.0	monomer	None	0.00	0.43 ± 0.05

Template	Seq Identity	Oligo-state	QSQE	Found by	Method	Resolution	Seq Similarity	Range	Coverage	Description
7ank.1.A	12.69	monomer	0.00	HHblits	X-ray	3.20Å	0.27	187 - 904	0.10	Alpha-actinin-2

The template contained no ligands.

```
Target    MSGEDQEVRAVVEDGSNGGSGSPSPGDTLPWNLEKTQRSRRGGGGPGGNGSVLDPAERAVIRIADERDRVQKKTFTKWVN
7ank.1.A  --------------------------------------------------------------------------------

Target    KHLIKAQRHISDLYEDLRDGHNLISLLEVLSGDSLPREKGRMRFHKLQNVQIALDYLRHRQVKLVNIRNDDIADGNPKLT
7ank.1.A  --------------------------------------------------------------------------------

Target    LGLIWTIILHFQISDIQVSGQSEDMTAKEKLLLWSQRMVEGYQGLRCDNFTSSWRDGRLFNAIIHRHKPMLIDMNK--VY
7ank.1.A  --------------------------QRKTFTAWCNSHLRKA-GTQIENIEEDFRNGLKLMLLLEVISGERLPKPDRGKM

Target    RQTNLENLDQAFSVAERDLGVT-RLLDPEDVDVPQPDEKSIITYVSSLYDAMPRVPDVQDGVKANELQLRWQEYRELVLL
7ank.1.A  RFHKIANVNKALDYIA-SKGVKLVSIGAEEIVDGNV--KMTLGMIWTIILRFAIQDISVE-------------ETSAKEG

Target    LLQWIRAHTAAFE---ERRFPSSFEEIEILWCQFLKFKETELPAKEAD----KNRSKGIYQSLEGAVQAGQLKVPPGYHP
7ank.1.A  LLLWCQRKTAPYRNVNIQNFHTSWKDGLGLCALIHRHRPDLIDYSKLNKDDPIGNINLAMEIAEK-----HLDIPKMLDA

Target    LDVEKEWGKLHVAILEREKQLRSEFERLERLQRIVSKLQMEAGLCEEQLNQADALLQSDVRLLAAGKAPQRAGEVERDLD
7ank.1.A  EDIVNT-P----------------------------KPDER--AIMTYVSCFYHAFAGAEQ-------------AET---

Target    KADGMIRLLFNDVQALKDGRHPQGEQMYRRVYRLHERLVAIRTEYNLRLRGTPRHPELEDSTLRYLQDLLAWVEENQRRV
7ank.1.A  -A-------------------------ANRICKVLAVNQENER--------------LMEEYERLASELLEWIRRTIPWL

Target    DSAEWGVDLPSVEAQLGSHRGLHQSVEEFRAKIERARTDEGQLSPATRGAYRDCLGRLDLQYAKLLNSSKARLRSLESLH
7ank.1.A  ENRTPEKTMQAMQKKLEDFRDYRRKHKPP---KV------------------QEKCQLEINFNTLQTKLR----------

Target    GFVAAATKELMWLSEKEEEEVGFDWSERNSNMAAKKEAYSALMRELELKEKKIKEIQSTGDRLLREDHPARPTVESFQAA
7ank.1.A  ------------------------------------------------------I---------SNRPAFMPSEGKMVSD

Target    LQTQWSWMLQLCCCIEAHLKE--------NTAYFQFFSDVREAEEQLRKLQETLHRKYTCDRSI-TVTRLEDLLQDAQDE
7ank.1.A  IAGAWQRLEQAEKGYEEWLLNEIRRLERLEHLAEKFRQKASTHETWAYGKEQILL---QKDYESASLTEVRALLRKHEAF

Target    KDQLNEYRGHLSGLAKRAKAIVQLTPRNPTQPTRGRVPLLAVCDYKQVEATVHKGDECQMLGPAQPFHWKVLSSSGSEAA
7ank.1.A  ESDLAAHQDRVEQIAAIAQELNELDY--H---------------------------------------------------
```

```
Target     VPSVCFLVPPPNQEALEAVARLEAQHQALVTLWHQLHTDMKSLLAWQSLSRDVQLIRSWSLVTFRTLKPEEQRQALRSLE
7ank.1.A   ----------DAVNVNDRCQKICDQWDRLGTLTQKRREALERM-------------------------------------

Target     LHYQAFLRDSQDAGGFGPEDRLQAEREYGSCSRHYQQLLQSLEQGEQEESRCQRCISELKDIRLQLEACETRTVHRLRLP
7ank.1.A   --------------------------------------------------------------------------------

Target     LDKEPARECAQRIAEQQKAQAEVEGLGKGVARLSAEAEKVLALPEPSPAAPTLRSELELTLGKLEQVRSLSAIYLEKLKT
7ank.1.A   --------------------------------------------------------------------------------

Target     ISLVIRSTQGAEEALRAHEEQLKEAQAVPAALPELEATKAAMKKLRAQAEAQQPVFDALRDELRGAQEVGERLQQRHGER
7ank.1.A   --------------------------------------------------------------------------------

Target     DVEVERWRERVTQLLERWQAVLAQTDVRQRELEQLGRQLRYYRESADPLGAWLQDARRRQEQIQAVPLADSQAVREQLRQ
7ank.1.A   --------------------------------------------------------------------------------

Target     EKALLEEIERHAEKVEECQRFAKQYINAIKDYELQLVTYKAQLEPVASPAKKPKVQSGSESVIQEYVDLRTRYSELSTLT
7ank.1.A   --------------------------------------------------------------------------------

Target     SQYIRFISETLRRMEEEERLAEQQRAEERERLAEVEAALEKQRQLAEAHAQAKAQAEREAQELQRRMQEEVARREEVAVD
7ank.1.A   --------------------------------------------------------------------------------

Target     AQQQKRSIQEELQQLRQSSEAEIQAKARQVEAAERSRLRIEEEIRVVRLQLETTERQRGGAEGELQALRARAEEAEAQKR
7ank.1.A   --------------------------------------------------------------------------------

Target     QAQEEAERLRRQVQEETQRKRQAEAELGLRVKAEAEAAREKQRALQALEELRLQAEEAERRLRQAEAERARQVQVALETA
7ank.1.A   --------------------------------------------------------------------------------

Target     QRSAQAELQSKHASFAEKTAQLERTLEEEHVTVVQLREEATRREQQQAEAERAREEAERELERWQLKANEALRLRLQAEE
7ank.1.A   --------------------------------------------------------------------------------

Target     VAQQKSLAQAEAEKQKEAAEREARRRGKAEEQAVRQRELAEQELERQRQLAEGTAQQRLAAEQELIRLRAETEQGEQQRQ
7ank.1.A   --------------------------------------------------------------------------------

Target     LLEEELARLQSEAAAATQKRQELEAELAKVRAEMEVLLASKARAEEESRSSSEKSKQRLEAEAGRFRELAEEAARLRALA
7ank.1.A   --------------------------------------------------------------------------------

Target     EEAKRQRQLAEEDAARQRAEAERVLSEKLAAISEATRLKTEAEIALKEKEAENERLRRLAEDEAFQRRRLEEQAAQHKAD
7ank.1.A   --------------------------------------------------------------------------------

Target     IEERLAQLRKASESELERQKGLVEDTLRQRRQVEEEILALKASFEKAAAGKAELELELGRIRGNAEDTLRSKEQAEQEAA
7ank.1.A   --------------------------------------------------------------------------------

Target     RQRQLAAEEERRRREAEERVQKSLAAEEEAARQRKAALEEVERLKAKVEEARRLRERAEHESVRQLQLAQEAAQKRLQAE
7ank.1.A   --------------------------------------------------------------------------------

Target     EKAHAFAVQQKEQELQQTLQQEQSVLEKLRSEAEAARRAAEEAEEARERAEREAAQSRQRVEEAERLKQAAEEQAQAQAQ
7ank.1.A   --------------------------------------------------------------------------------

Target     AQAAAEKLRKEAEQEAARRAQAEQAALRQKQAADAEMEKHKKFAEQTLRQKAQVEQELTALRLKLEETDHQKSILDQELQ
7ank.1.A   --------------------------------------------------------------------------------

Target     RLKAEVTEAARQRSQVEEELFSVRVQMEELGKLKARIEAENRALILRDKDNTQRLLQEEAEKMKQVAEEAARLSVAAQEA
7ank.1.A   --------------------------------------------------------------------------------

Target     ARLRQLAEEDLAQQRALAEKMLKEKMQAVQEATRLKAEAELLQQQKELAQEQARRLQEDKEQMAQQLAQETQGFQRTLET
7ank.1.A   --------------------------------------------------------------------------------

Target     ERQRQLEMSAEAERLRLRVAEMSRAQARAEEDAQRFRKQAEEIGAKLHRTELATQEKVTLVQTLETQRQQSDRDADRLRE
7ank.1.A   --------------------------------------------------------------------------------

Target     AIAELEREKDKLKKEAELLQLKSEEMQTVQQEQLLQETQALQQSFLSEKDSLLQRERFIEEEKAKLERLFQDEVAKAQKL
7ank.1.A   --------------------------------------------------------------------------------

Target     REEQQRQQQQMQQEKQQLLASMEEARRRQHEAEEGVRRKQEELQLLEQQRQQQEQLLAEENRRLRERLEHLEEEHRAALA
7ank.1.A   --------------------------------------------------------------------------------

Target     HSEEITAAQAAATRALPNGQDAADGPAAEPEHAFEGLRQKVPAQQLQEAGILSTEELQRLAQGHTTVAELTQREDVRRYL
7ank.1.A   --------------------------------------------------------------------------------

Target     QGHSSIAGLLLKPANEKLTIYAALRRQLLSPGTALILLEAQAASGFLLDPVRNRRLTVTEAVKEGVVGPELHHKLLSAER
7ank.1.A   --------------------------------------------------------------------------------

Target     AVTGYKDPYTGEKISLFQAMKKDLIVREHGIRLLEAQIATGGVIDPVHSHRVPVDVAYHRGYFDEEMNRVLQDPSDDTKG
7ank.1.A   --------------------------------------------------------------------------------
```

```
Target     FFDPNTHENLTYLQLLERCVEDPETGLRLLPLTDQAAKGGELVYTDSEARDVFEKATVSAPFGKFQGKTVTIWELINSEY
7ank.1.A   --------------------------------------------------------------------------------

Target     FTAEQRRDLLRQFRTGKVTVEKIIKIVITVIEEHEQKGQLCFQGLRALVPAAELLESGIIDWDLFRQLQLGERSVQEVAE
7ank.1.A   --------------------------------------------------------------------------------

Target     VEGVRRALRGSGVIAGVWLEEARQKLSIYEALKKELLQPEAAVALLEAQAGTGHVIDPATSARLTVDEAVRAGLVGPELH
7ank.1.A   --------------------------------------------------------------------------------

Target     EKLLSAEKAVTGYKDPYSGQSVSLFQALKKGLIPREQGLRLLDAQLSTGGTVDPSKSHRLPLDVACARGYLDEETSTALS
7ank.1.A   --------------------------------------------------------------------------------

Target     APRDDAKTYYDPRTWELATYSQLQQQCRPDPLTGLSLLPLSEEAARARQQELYSEVQAREAFQKATVEVPVGSFQGRAVT
7ank.1.A   --------------------------------------------------------------------------------

Target     IWELINSEYFTAEQRQELLRQFRTGKVTVEKIIKIVITIVEEVETTRRERLSFSGLRAPVPASELLAAGILSSSQFEQLK
7ank.1.A   --------------------------------------------------------------------------------

Target     DGKTSVKDLSELSSLQTLLQGSGCLAGIYLEESKEKVTIYEAMRRGLLRPSTAILLLEAQAATGFLVDPVRNQRLYVHEA
7ank.1.A   --------------------------------------------------------------------------------

Target     VKAGVVGPELHEKLLSAEKAVTGYKDPYSGSTISLFQAMKKGLVVREHGIRLLEAQIATGGIIDPVHSHRVPVDVACQRG
7ank.1.A   --------------------------------------------------------------------------------

Target     YFDEEMNRVLEDPSDDTKGFFDPNTHENLTYRQLLERCVEDPETGLRLLPLKGPEKAEVVETTRVYTEEETRRAFEETQI
7ank.1.A   --------------------------------------------------------------------------------

Target     DIPGGGSHGGSTMSLWEVMQSDLIPEEQRAQLMADFQAGRVTKERMIIIIIEIIEKTEIVRQQNLASYDYVRRRLTAEDL
7ank.1.A   --------------------------------------------------------------------------------

Target     YEARVISRESYSLLREGTRSLREVLEAESAWRYLYGTGCVAGVYLPGSRQTLPIYQALKKGLLSAEVARLLLEAQAATGF
7ank.1.A   --------------------------------------------------------------------------------

Target     LLDPVKGERLTVDEAVRKGLVGPELHDRLLSAERAVTGYRDPYTEQTISLFQAMKKDLIPAEEALRLLDAQLATGGIVDP
7ank.1.A   --------------------------------------------------------------------------------

Target     RLGFHLPLEVAYQRGYLNKDTHDQLSEPSEVRSYVDPSTDERLSYTQLLRRCRRDETSGLFLLPLSEARKLTFRGLRKQI
7ank.1.A   --------------------------------------------------------------------------------

Target     TVEELVRSHVMDEATAQRLQEGLTSIEEVSKNLQKFLEGTSSIAGVLVDATKERLSVYQAMKKGIIRPGTAFELLEAQAA
7ank.1.A   --------------------------------------------------------------------------------

Target     TGYVIDPIKGLKLTVEEAVRMGIVGPEFKDKLLSAERAVTGYKDPYSGKLISLFQAMKKGLILKDHGIRLLEAQIATGGI
7ank.1.A   --------------------------------------------------------------------------------

Target     IDPEESHRLPVDVAYQRGLFDEEMNEILTDPSDDTKGFFDPNTEENLTYLQLMERCVTDPQTGLRLLPLKEKKRERKTSS
7ank.1.A   --------------------------------------------------------------------------------

Target     KSSVRKRRVVIVDPETGKEMSVYEAYRKGLIDHQTYLELSEQECEWEEITISSSDGVVKSMIIDRRSGRQYDIDEAIAKS
7ank.1.A   --------------------------------------------------------------------------------

Target     LIDRSALDQYRAGTLSITEFADMLSGNAGGFRSRSSSVGSSSSYPISPAVSRTQLASWSDPTEETGPVAGILDTETLEKV
7ank.1.A   --------------------------------------------------------------------------------

Target     SITEAMHRNLVDNITGQRLLEAQACTGGIIDPNTGERFPVTDAVNKGLVDKIMVDRINLAQKAFCGFEDPRTKTKMSAAQ
7ank.1.A   --------------------------------------------------------------------------------

Target     ALKKGWLYYEAGQRFLEVQYLTGGLIEPDTPGRVPLDEALQRGTVDARTAQKLRDVSAYSKYLTCPKTKLKISYKDALDR
7ank.1.A   --------------------------------------------------------------------------------

Target     SMVEEGTGLRLLEAAAQSSKGYYSPYSVSGSGSTTGSRSGSRTGSRAGSRRGSFDATGSGFSMTFSSSSYSSSGYGRRYA
7ank.1.A   --------------------------------------------------------------------------------

Target     SGPTSSLGGPESAAA
7ank.1.A   ---------------
```

Model #02	File	Built with	Oligo-State	Ligands	GMQE	QMEANDisCo Global
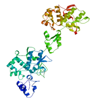	PDB	ProMod3 3.3.0	monomer	None	0.00	0.53 ± 0.05

Template	Seq Identity	Oligo-state	QSQE	Found by	Method	Resolution	Seq Similarity	Range	Coverage	Description
5dzz.1.A	45.12	monomer	0.00	BLAST	X-ray	2.60Å	0.40	3049 - 3553	0.10	Desmoplakin

The template contained no ligands.

```
Target    MSGEDQEVRAVVEDGSNGGSGSPSPGDTLPWNLEKTQRSRRGGGGPGGNGSVLDPAERAVIRIADERDRVQKKTFTKWVN
5dzz.1.A  --------------------------------------------------------------------------------

Target    KHLIKAQRHISDLYEDLRDGHNLISLLEVLSGDSLPREKGRMRFHKLQNVQIALDYLRHRQVKLVNIRNDDIADGNPKLT
5dzz.1.A  --------------------------------------------------------------------------------

Target    LGLIWTIILHFQISDIQVSGQSEDMTAKEKLLLWSQRMVEGYQGLRCDNFTSSWRDGRLFNAIIHRHKPMLIDMNKVYRQ
5dzz.1.A  --------------------------------------------------------------------------------

Target    TNLENLDQAFSVAERDLGVTRLLDPEDVDVPQPDEKSIITYVSSLYDAMPRVPDVQDGVKANELQLRWQEYRELVLLLLQ
5dzz.1.A  --------------------------------------------------------------------------------

Target    WIRAHTAAFEERRFPSSFEEIEILWCQFLKFKETELPAKEADKNRSKGIYQSLEGAVQAGQLKVPPGYHPLDVEKEWGKL
5dzz.1.A  --------------------------------------------------------------------------------

Target    HVAILEREKQLRSEFERLERLQRIVSKLQMEAGLCEEQLNQADALLQSDVRLLAAGKAPQRAGEVERDLDKADGMIRLLF
5dzz.1.A  --------------------------------------------------------------------------------

Target    NDVQALKDGRHPQGEQMYRRVYRLHERLVAIRTEYNLRLRGTPRHPELEDSTLRYLQDLLAWVEENQRRVDSAEWGVDLP
5dzz.1.A  --------------------------------------------------------------------------------

Target    SVEAQLGSHRGLHQSVEEFRAKIERARTDEGQLSPATRGAYRDCLGRLDLQYAKLLNSSKARLRSLESLHGFVAAATKEL
5dzz.1.A  --------------------------------------------------------------------------------

Target    MWLSEKEEEEVGFDWSERNSNMAAKKEAYSALMRELELKEKKIKEIQSTGDRLLREDHPARPTVESFQAALQTQWSWMLQ
5dzz.1.A  --------------------------------------------------------------------------------

Target    LCCCIEAHLKENTAYFQFFSDVREAEEQLRKLQETLHRKYTCDRSITVTRLEDLLQDAQDEKDQLNEYRGHLSGLAKRAK
5dzz.1.A  --------------------------------------------------------------------------------

Target    AIVQLTPRNPTQPTRGRVPLLAVCDYKQVEATVHKGDECQMLGPAQPFHWKVLSSSGSEAAVPSVCFLVPPPNQEALEAV
5dzz.1.A  --------------------------------------------------------------------------------

Target    ARLEAQHQALVTLWHQLHTDMKSLLAWQSLSRDVQLIRSWSLVTFRTLKPEEQRQALRSLELHYQAFLRDSQDAGGFGPE
5dzz.1.A  --------------------------------------------------------------------------------

Target    DRLQAEREYGSCSRHYQQLLQSLEQGEQEESRCQRCISELKDIRLQLEACETRTVHRLRLPLDKEPARECAQRIAEQQKA
5dzz.1.A  --------------------------------------------------------------------------------

Target    QAEVEGLGKGVARLSAEAEKVLALPEPSPAAPTLRSELELTLGKLEQVRSLSAIYLEKLKTISLVIRSTQGAEEALRAHE
5dzz.1.A  --------------------------------------------------------------------------------

Target    EQLKEAQAVPAALPELEATKAAMKKLRAQAEAQQPVFDALRDELRGAQEVGERLQQRHGERDVEVERWRERVTQLLERWQ
5dzz.1.A  --------------------------------------------------------------------------------

Target    AVLAQTDVRQRELEQLGRQLRYYRESADPLGAWLQDARRRQEQIQAVPLADSQAVREQLRQEKALLEEIERHAEKVEECQ
5dzz.1.A  --------------------------------------------------------------------------------

Target    RFAKQYINAIKDYELQLVTYKAQLEPVASPAKKPKVQSGSESVIQEYVDLRTRYSELSTLTSQYIRFISETLRRMEEEER
5dzz.1.A  --------------------------------------------------------------------------------

Target    LAEQQRAEERERLAEVEAALEKQRQLAEAHAQKAQAEREAQELQRRMQEEVARREEVAVDAQQQKRSIQEELQQLRQSS
5dzz.1.A  --------------------------------------------------------------------------------

Target    EAEIQAKARQVEAAERSRLRIEEEIRVVRLQLETTERQRGGAEGELQALRARAEEAEAQKRQAQEEAERLRRQVQEETQR
5dzz.1.A  --------------------------------------------------------------------------------
```

```
Target    KRQAEAELGLRVKAEAEAAREKQRALQALEELRLQAEEAERRLRQAEAERARQVQVALETAQRSAQAELQSKHASFAEKT
5dzz.1.A  --------------------------------------------------------------------------------

Target    AQLERTLEEEHVTVVQLREEATRREQQQAEAERAREEAERELERWQLKANEALRLRLQAEEVAQQKSLAQAEAEKQKEAA
5dzz.1.A  --------------------------------------------------------------------------------

Target    EREARRRGKAEEQAVRQRELAEQELERQRQLAEGTAQQRLAAEQELIRLRAETEQGEQQRQLLEEELARLQSEAAAATQK
5dzz.1.A  --------------------------------------------------------------------------------

Target    RQELEAELAKVRAEMEVLLASKARAEEESRSSSEKSKQRLEAEAGRFRELAEEAARLRALAEEAKRQRQLAEEDAARQRA
5dzz.1.A  --------------------------------------------------------------------------------

Target    EAERVLSEKLAAISEATRLKTEAEIALKEKEAENERLRRLAEDEAFQRRRLEEQAAQHKADIEERLAQLRKASESELERQ
5dzz.1.A  --------------------------------------------------------------------------------

Target    KGLVEDTLRQRRQVEEEILALKASFEKAAAGKAELELELGRIRGNAEDTLRSKEQAEQEAARQRQLAAEEERRRREAEER
5dzz.1.A  --------------------------------------------------------------------------------

Target    VQKSLAAEEEAARQRKAALEEVERLKAKVEEARRLRERAEHESVRQLQLAQEAAQKRLQAEEKAHAFAVQQKEQELQQTL
5dzz.1.A  --------------------------------------------------------------------------------

Target    QQEQSVLEKLRSEAEAARRAAEEAEEARERAEREAAQSRQRVEEAERLKQAAEEQAQAQAQAQAAAEKLRKEAEQEAARR
5dzz.1.A  --------------------------------------------------------------------------------

Target    AQAEQAALRQKQAADAEMEKHKKFAEQTLRQKAQVEQELTALRLKLEETDHQKSILDQELQRLKAEVTEAARQRSQVEEE
5dzz.1.A  --------------------------------------------------------------------------------

Target    LFSVRVQMEELGKLKARIEAENRALILRDKDNTQRLLQEEAEKMKQVAEEAARLSVAAQEAARLRQLAEEDLAQQRALAE
5dzz.1.A  --------------------------------------------------------------------------------

Target    KMLKEKMQAVQEATRLKAEAELLQQQKELAQEQARRLQEDKEQMAQQLAQETQGFQRTLETERQRQLEMSAEAERLRLRV
5dzz.1.A  --------------------------------------------------------------------------------

Target    AEMSRAQARAEEDAQRFRKQAEEIGAKLHRTELATQEKVTLVQTLETQRQQSDRDADRLREAIAELEREKDKLKKEAELL
5dzz.1.A  --------------------------------------------------------------------------------

Target    QLKSEEMQTVQQEQLLQETQALQQSFLSEKDSLLQRERFIEEEKAKLERLFQDEVAKAQKLREEQQRQQQMQQEKQQLL
5dzz.1.A  --------------------------------------------------------------------------------

Target    ASMEEARRRQHEAEEGVRRKQEELQLLEQQRQQQEQLLAEENRRLRERLEHLEEEHRAALAHSEEITAAQAAATRALPNG
5dzz.1.A  --------------------------------------------------------------------------------

Target    QDAADGPAAEPEHAFEGLRQKVPAQQLQEAGILSTEELQRLAQGHTTVAELTQREDVRRYLQGHSSIAGLLLKPANEKLT
5dzz.1.A  --------------------------------------------------------------------------------

Target    IYAALRRQLLSPGTALILLEAQAASGFLLDPVRNRRLTVTEAVKEGVVGPELHHKLLSAERAVTGYKDPYTGEKISLFQA
5dzz.1.A  --------------------------------------------------------------------------------

Target    MKKDLIVREHGIRLLEAQIATGGVIDPVHSHRVPVDVAYHRGYFDEEMNRVLQDPSDDTKGFFDPNTHENLTYLQLLERC
5dzz.1.A  --------------------------------------------------------------------------------

Target    VEDPETGLRLLPLTDQAAKGGELVYTDSEARDVFEKATVSAPFGKFQGKTVTIWELINSEYFTAEQRRDLLRQFRTGKVT
5dzz.1.A  --------------------------------------------------------------------------------

Target    VEKIIKIVITVIEEHEQKGQLCFQGLRALVPAAELLESGIIDWDLFRQLQLGERSVQEVAEVEGVRRALRGSGVIAGVWL
5dzz.1.A  --------------------------------------------------------------------------------

Target    EEARQKLSIYEALKKELLQPEAAVALLEAQAGTGHVIDPATSARLTVDEAVRAGLVGPELHEKLLSAEKAVTGYKDPYSG
5dzz.1.A  --------VFDGLRKKV----TAMQLYECQLIDKTTLDKLLKGKKSVEE------VASEIQPFLRGAGSIAGASASPK--

Target    QSVSLFQALKKGLIPREQGLRLLDAQLSTGGTVDPSKSHRLPLDVACAR-------------------GYLDEETSTALS
5dzz.1.A  EKYSLVEAKRKKLISPESTVMLLEAQAATGGIIDPHRNEKLTVDSAIARDLIDFDDRQQIYAAEKAITGFDDPFSGKTVS

Target    APRDDAKTYYDPRTWELATYSQLQQQCRPDPLTGLSLLPLSEEAARAR-QQELYSEVQAREAFQKATVEVPVGSFQGRAV
5dzz.1.A  VSEAIKKNLIDRETGMRLLEAQIASGGVVDPVNSV-FLPKDVALARGLIDRDLYRSLNDPRDSQKNFVD-PVTKKKVSYV

Target    TIWELINSEYFTAEQRQELLRQFRTGKVTVEKIIKIVITIVEEVETTRRERLSFSGLRAPVPASELLAAGILSSSQFEQL
5dzz.1.A  QLKERCRIEPHTG----------------------------LLLLSVQKRSMSFQGIRQPVTVTELVDSGILRPSTVNEL

Target    KDGKTSVKDLSELSSLQTLLQGSGCLAGIYLEESKEKVTIYEAMRRGLLRPSTAILLLEAQAATGFLVDPVRNQRLYVHE
5dzz.1.A  ESGQISYDEVGERIKDFLQGSSCI--AGIYNETTKQKLGIYEAMKIGLVRPGTALELLEAQAATGFIVDPVSNLRLPVEE

Target    AVKAGVVGPELHEKLLSAEKAVTGYKDPYSGSTISLFQAMKKGLVVREHGIRLLEAQIATGGIIDPVHSHRVPVDVACQR
5dzz.1.A  AYKRGLVGIEFKEKLLSAERAVTGYNDPETGNIISLFQAMNKELIEKGHGIRLLEAQIATGGIIDPKESHRLPVDIAYKR
```

```
Target     GYFDEEMNRVLEDPSDDTKGFFDPNTHENLTYRQLLERCVEDPETGLRLLPLKGPEKAEVVETTRVYTEEETRRAFEETQ
5dzz.1.A   GYFNEELSEILSDPSDDTKGFFDPNTEENLTYLQLKERCIKDEETGLCLLPLK---------------------------

Target     IDIPGGGSHGGSTMSLWEVMQSDLIPEEQRAQLMADFQAGRVTKERMIIIIIEIIEKTEIVRQQNLASYDYVRRRLTAED
5dzz.1.A   --------------------------------------------------------------------------------

Target     LYEARVISRESYSLLREGTRSLREVLEAESAWRYLYGTGCVAGVYLPGSRQTLPIYQALKKGLLSAEVARLLLEAQAATG
5dzz.1.A   --------------------------------------------------------------------------------

Target     FLLDPVKGERLTVDEAVRKGLVGPELHDRLLSAERAVTGYRDPYTEQTISLFQAMKKDLIPAEEALRLLDAQLATGGIVD
5dzz.1.A   --------------------------------------------------------------------------------

Target     PRLGFHLPLEVAYQRGYLNKDTHDQLSEPSEVRSYVDPSTDERLSYTQLLRRCRRDETSGLFLLPLSEARKLTFRGLRKQ
5dzz.1.A   --------------------------------------------------------------------------------

Target     ITVEELVRSHVMDEATAQRLQEGLTSIEEVSKNLQKFLEGTSSIAGVLVDATKERLSVYQAMKKGIIRPGTAFELLEAQA
5dzz.1.A   --------------------------------------------------------------------------------

Target     ATGYVIDPIKGLKLTVEEAVRMGIVGPEFKDKLLSAERAVTGYKDPYSGKLISLFQAMKKGLILKDHGIRLLEAQIATGG
5dzz.1.A   --------------------------------------------------------------------------------

Target     IIDPEESHRLPVDVAYQRGLFDEEMNEILTDPSDDTKGFFDPNTEENLTYLQLMERCVTDPQTGLRLLPLKEKKRERKTS
5dzz.1.A   --------------------------------------------------------------------------------

Target     SKSSVRKRRVVIVDPETGKEMSVYEAYRKGLIDHQTYLELSEQECEWEEITISSSDGVVKSMIIDRRSGRQYDIDEAIAK
5dzz.1.A   --------------------------------------------------------------------------------

Target     SLIDRSALDQYRAGTLSITEFADMLSGNAGGFRSRSSSVGSSSSYPISPAVSRTQLASWSDPTEETGPVAGILDTETLEK
5dzz.1.A   --------------------------------------------------------------------------------

Target     VSITEAMHRNLVDNITGQRLLEAQACTGGIIDPNTGERFPVTDAVNKGLVDKIMVDRINLAQKAFCGFEDPRTKTKMSAA
5dzz.1.A   --------------------------------------------------------------------------------

Target     QALKKGWLYYEAGQRFLEVQYLTGGLIEPDTPGRVPLDEALQRGTVDARTAQKLRDVSAYSKYLTCPKTKLKISYKDALD
5dzz.1.A   --------------------------------------------------------------------------------

Target     RSMVEEGTGLRLLEAAAQSSKGYYSPYSVSGSGSTTGSRSGSRTGSRAGSRRGSFDATGSGFSMTFSSSSYSSSGYGRRY
5dzz.1.A   --------------------------------------------------------------------------------

Target     ASGPTSSLGGPESAAA
5dzz.1.A   ----------------
```

Model #18	File	Built with	Oligo-State	Ligands	GMQE	QMEANDisCo Global
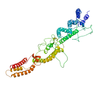	PDB	ProMod3 3.3.0	monomer (matching prediction)	None	0.00	0.44 ± 0.05

Template	Seq Identity	Oligo-state	QSQE	Found by	Method	Resolution	Seq Similarity	Range	Coverage	Description
4d1e.1.A	12.65	homo-dimer	0.00	HHblits	X-ray	3.50Å	0.26	627 - 1221	0.09	ALPHA-ACTININ-2

The template contained no ligands.

```
Target     MSGEDQEVRAVVEDGSNGGSGSPSPGDTLPWNLEKTQRSRRGGGGPGGNGSVLDPAERAVIRIADERDRVQKKTFTKWVN
4d1e.1.A   --------------------------------------------------------------------------------

Target     KHLIKAQRHISDLYEDLRDGHNLISLLEVLSGDSLPREKGRMRFHKLQNVQIALDYLRHRQVKLVNIRNDDIADGNPKLT
4d1e.1.A   --------------------------------------------------------------------------------

Target     LGLIWTIILHFQISDIQVSGQSEDMTAKEKLLLWSQRMVEGYQGLRCDNFTSSWRDGRLFNAIIHRHKPMLIDMNKVYRQ
4d1e.1.A   --------------------------------------------------------------------------------

Target     TNLENLDQAFSVAERDLGVTRLLDPEDVDVPQPDEKSIITYVSSLYDAMPRVPDVQDGVKANELQLRWQEYRELVLLLLQ
4d1e.1.A   --------------------------------------------------------------------------------
```

```
Target     WIRAHTAAFEERRFPSSFEEIEILWCQFLKFKETELPAKEADKNRSKGIYQSLEGAVQAGQLKVPPGYHPLDVEKEWGKL
4dle.1.A   --------------------------------------------------------------------------------

Target     HVAILEREKQLRSEFERLERLQRIVSKLQMEAGLCEEQLNQADALLQSDVRLLAAGKAPQRAGEVERDLDKADGMIRLLF
4dle.1.A   --------------------------------------------------------------------------------

Target     NDVQALKDGRHPQGEQMYRRVYRLHERLVAIRTEYNLRLRGTPRHPELEDSTLRYLQDLLAWVEENQRRVDSAEWGVDLP
4dle.1.A   --------------------------------------------------------------------------------

Target     SVEAQLGSHRGLHQSVEEFRAKIERARTDEGQLSPATRGAYRDCLGRLDLQYAKLLNSSKARLRSLESLHGFVAAATKEL
4dle.1.A   ------------------------------------------------------------------RLMEEYERLASELL

Target     MWLSEKEEEEVGFDWSERNSNMAAKKEAYSALMRELELKEKKIK-EIQST----GDRLL-REDHPARPTVESFQAALQTQ
4dle.1.A   EWIRRTIPWLENRTPAATMQAMQKKLEDFRDYRRKHKPPKVQEKCQLEINFNTLQTKLRISNRPAFMPSEGKMVSDIAGA

Target     WSWMLQLCCCIEAHLKE--------NTAYFQFFSDVREAEEQLRKLQETLHRKYTCDR-SITVTRLEDLLQDAQDEKDQL
4dle.1.A   WQRLEQAEKGYEEWLLNEIRRLERLEHLAEKFRQKASTHETWAYGKEQILLQ---KDYESASLTEVRALLRKHEAFESDL

Target     NEYRGHLSGLAKRAKAIVQLTPRNPTQPTRGRVPLLAVCDYKQVEATVHKGDECQMLGPAQPFHWKVLSSSGSEAAVPSV
4dle.1.A   AAHQDRVEQIAAIAQELNELDY--H-D-----------------------------------------------------

Target     CFLVPPPNQEALEAVARLEAQHQALVTLWHQLHTDMKSLLAW--------QSLSRDVQLIRSWSLVTFRTLKPEEQRQAL
4dle.1.A   -------AVNVNDRCQKICDQWDRLGTLTQKRREALERMEKLLETIDQLHLEFAKRAAPFNNWME---------------

Target     RSLELHYQAFLRDSQDAGGFGPEDRLQAEREYGSCSRHYQQLLQSLEQGEQEESRCQRCISELKDIRLQLEACETRTVHR
4dle.1.A   -----------------------------------------------------------------------GAMEDLQDM

Target     LRLPLDKEPARECAQRIAEQQKAQAEVEGLGKGVARLSAEAEKVLALPEP-----SPAAPTLRSELELTLGKLEQVRSLS
4dle.1.A   F---IV-HSIEEIQSLITAHEQFKATLPEADGERQSIMAIQNEVEKVIQSYNIRISSSNPYSTVTMDELRTKWDKVKQLV

Target     AIYLEKLKTI-----------SLVIRSTQGAEEALRAHEEQLKEAQAVPAALPELEATKAAMKKLRAQAEAQQPVFDALR
4dle.1.A   PIRDQSLQEELARQHANERLRRQFAAQANAIGPWIQNKMEEIAR--SSIQITGALEDQMNQLKQYEHNIINYKNNIDKLE

Target     DELRGAQEVGERLQQRHGERDVEVERWRERVTQLLERWQAVLAQTDVRQRELEQLGRQLRYYRESADPLGAWLQDARRRQ
4dle.1.A   -------GDHQLIQEALV---FDNKHTNYTMEHIRVGWELLLTTIARTINEVETQILTRD--------------------

Target     EQIQAVPLADSQAVREQLRQEKALLEEIERHAEKVEECQRFAKQYINAIKDYELQLVTYKAQLEPVASPAKKPKVQSGSE
4dle.1.A   --------------------------------------------------------------------------------

Target     SVIQEYVDLRTRYSELSTLTSQYIRFISETLRRMEEEERLAEQQRAEERERLAEVEAALEKQRQLAEAHAQAKAQAEREA
4dle.1.A   --------------------------------------------------------------------------------

Target     QELQRRMQEEVARREEVAVDAQQQKRSIQEELQQLRQSSEAEIQAKARQVEAAERSRLRIEEEIRVVRLQLETTERQRGG
4dle.1.A   --------------------------------------------------------------------------------

Target     AEGELQALRARAEEAEAQKRQAQEEAERLRRQVQEETQRKRQAEAELGLRVKAEAEAAREKQRALQALEELRLQAEEAER
4dle.1.A   --------------------------------------------------------------------------------

Target     RLRQAEAERARQVQVALETAQRSAQAELQSKHASFAEKTAQLERTLEEEHVTVVQLREEATRREQQQAEAERAREEAERE
4dle.1.A   --------------------------------------------------------------------------------

Target     LERWQLKANEALRLRLQAEEVAQQKSLAQAEAEKQKEAAEREARRRGKAEEQAVRQRELAEQELERQRQLAEGTAQQRLA
4dle.1.A   --------------------------------------------------------------------------------

Target     AEQELIRLRAETEQGEQQRQLLEEELARLQSEAAAATQKRQELEAELAKVRAEMEVLLASKARAEEESRSSSEKSKQRLE
4dle.1.A   --------------------------------------------------------------------------------

Target     AEAGRFRELAEEAARLRALAEEAKRQRQLAEEDAARQRAEAERVLSEKLAAISEATRLKTEAEIALKEKEAENERLRRLA
4dle.1.A   --------------------------------------------------------------------------------

Target     EDEAFQRRRLEEQAAQHKADIEERLAQLRKASESELERQKGLVEDTLRQRRQVEEEILALKASFEKAAAGKAELELELGR
4dle.1.A   --------------------------------------------------------------------------------

Target     IRGNAEDTLRSKEQAEQEAARQRQLAAEEERRRREAEERVQKSLAAEEEAARQRKAALEEVERLKAKVEEARRLRERAEH
4dle.1.A   --------------------------------------------------------------------------------

Target     ESVRQLQLAQEAAQKRLQAEEKAHAFAVQQKEQELQQTLQQEQSVLEKLRSEAEAARRAAEEAEEARERAEREAAQSRQR
4dle.1.A   --------------------------------------------------------------------------------

Target     VEEAERLKQAAEEQAQAQAQAQAAAEKLRKEAEQEAARRAQAEQAALRQKQAADAEMEKHKKFAEQTLRQKAQVEQELTA
4dle.1.A   --------------------------------------------------------------------------------

Target     LRLKLEETDHQKSILDQELQRLKAEVTEAARQRSQVEEELFSVRVQMEELGKLKARIEAENRALILRDKDNTQRLLQEEA
```

```
4d1e.1.A    --------------------------------------------------------------------------------

Target      EKMKQVAEEAARLSVAAQEAARLRQLAEEDLAQQRALAEKMLKEKMQAVQEATRLKAEAELLQQQKELAQEQARRLQEDK
4d1e.1.A    --------------------------------------------------------------------------------

Target      EQMAQQLAQETQGFQRTLETERQRQLEMSAEAERLRLRVAEMSRAQARAEEDAQRFRKQAEEIGAKLHRTELATQEKVTL
4d1e.1.A    --------------------------------------------------------------------------------

Target      VQTLETQRQQSDRDADRLREAIAELEREKDKLKKEAELLQLKSEEMQTVQQEQLLQETQALQQSFLSEKDSLLQRERFIE
4d1e.1.A    --------------------------------------------------------------------------------

Target      EEKAKLERLFQDEVAKAQKLREEQQRQQQMQQEKQQLLASMEEARRRQHEAEEGVRRKQEELQLLEQQRQQQEQLLAEE
4d1e.1.A    --------------------------------------------------------------------------------

Target      NRRLRERLEHLEEEHRAALAHSEEITAAQAAATRALPNGQDAADGPAAEPEHAFEGLRQKVPAQQLQEAGILSTEELQRL
4d1e.1.A    --------------------------------------------------------------------------------

Target      AQGHTTVAELTQREDVRRYLQGHSSIAGLLLKPANEKLTIYAALRRQLLSPGTALILLEAQAASGFLLDPVRNRRLTVTE
4d1e.1.A    --------------------------------------------------------------------------------

Target      AVKEGVVGPELHHKLLSAERAVTGYKDPYTGEKISLFQAMKKDLIVREHGIRLLEAQIATGGVIDPVHSHRVPVDVAYHR
4d1e.1.A    --------------------------------------------------------------------------------

Target      GYFDEEMNRVLQDPSDDTKGFFDPNTHENLTYLQLLERCVEDPETGLRLLPLTDQAAKGGELVYTDSEARDVFEKATVSA
4d1e.1.A    --------------------------------------------------------------------------------

Target      PFGKFQGKTVTIWELINSEYFTAEQRRDLLRQFRTGKVTVEKIIKIVITVIEEHEQKGQLCFQGLRALVPAAELLESGII
4d1e.1.A    --------------------------------------------------------------------------------

Target      DWDLFRQLQLGERSVQEVAEVEGVRRALRGSGVIAGVWLEEARQKLSIYEALKKELLQPEAAVALLEAQAGTGHVIDPAT
4d1e.1.A    --------------------------------------------------------------------------------

Target      SARLTVDEAVRAGLVGPELHEKLLSAEKAVTGYKDPYSGQSVSLFQALKKGLIPREQGLRLLDAQLSTGGTVDPSKSHRL
4d1e.1.A    --------------------------------------------------------------------------------

Target      PLDVACARGYLDEETSTALSAPRDDAKTYYDPRTWELATYSQLQQQCRPDPLTGLSLLPLSEEAARARQQELYSEVQARE
4d1e.1.A    --------------------------------------------------------------------------------

Target      AFQKATVEVPVGSFQGRAVTIWELINSEYFTAEQRQELLRQFRTGKVTVEKIIKIVITIVEEVETTRRERLSFSGLRAPV
4d1e.1.A    --------------------------------------------------------------------------------

Target      PASELLAAGILSSSQFEQLKDGKTSVKDLSELSSLQTLLQGSGCLAGIYLEESKEKVTIYEAMRRGLLRPSTAILLLEAQ
4d1e.1.A    --------------------------------------------------------------------------------

Target      AATGFLVDPVRNQRLYVHEAVKAGVVGPELHEKLLSAEKAVTGYKDPYSGSTISLFQAMKKGLVVREHGIRLLEAQIATG
4d1e.1.A    --------------------------------------------------------------------------------

Target      GIIDPVHSHRVPVDVACQRGYFDEEMNRVLEDPSDDTKGFFDPNTHENLTYRQLLERCVEDPETGLRLLPLKGPEKAEVV
4d1e.1.A    --------------------------------------------------------------------------------

Target      ETTRVYTEEETRRAFEETQIDIPGGGSHGGSTMSLWEVMQSDLIPEEQRAQLMADFQAGRVTKERMIIIIIEIIEKTEIV
4d1e.1.A    --------------------------------------------------------------------------------

Target      RQQNLASYDYVRRRLTAEDLYEARVISRESYSLLREGTRSLREVLEAESAWRYLYGTGCVAGVYLPGSRQTLPIYQALKK
4d1e.1.A    --------------------------------------------------------------------------------

Target      GLLSAEVARLLLEAQAATGFLLDPVKGERLTVDEAVRKGLVGPELHDRLLSAERAVTGYRDPYTEQTISLFQAMKKDLIP
4d1e.1.A    --------------------------------------------------------------------------------

Target      AEEALRLLDAQLATGGIVDPRLGFHLPLEVAYQRGYLNKDTHDQLSEPSEVRSYVDPSTDERLSYTQLLRRCRRDETSGL
4d1e.1.A    --------------------------------------------------------------------------------

Target      FLLPLSEARKLTFRGLRKQITVEELVRSHVMDEATAQRLQEGLTSIEEVSKNLQKFLEGTSSIAGVLVDATKERLSVYQA
4d1e.1.A    --------------------------------------------------------------------------------

Target      MKKGIIRPGTAFELLEAQAATGYVIDPIKGLKLTVEEAVRMGIVGPEFKDKLLSAERAVTGYKDPYSGKLISLFQAMKKG
4d1e.1.A    --------------------------------------------------------------------------------

Target      LILKDHGIRLLEAQIATGGIIDPEESHRLPVDVAYQRGLFDEEMNEILTDPSDDTKGFFDPNTEENLTYLQLMERCVTDP
4d1e.1.A    --------------------------------------------------------------------------------

Target      QTGLRLLPLKEKKRERKTSSKSSVRKRRVVIVDPETGKEMSVYEAYRKGLIDHQTYLELSEQECEWEEITISSSDGVVKS
4d1e.1.A    --------------------------------------------------------------------------------
```

```
Target     MIIDRRSGRQYDIDEAIAKSLIDRSALDQYRAGTLSITEFADMLSGNAGGFRSRSSSVGSSSSYPISPAVSRTQLASWSD
4d1e.1.A   --------------------------------------------------------------------------------

Target     PTEETGPVAGILDTETLEKVSITEAMHRNLVDNITGQRLLEAQACTGGIIDPNTGERFPVTDAVNKGLVDKIMVDRINLA
4d1e.1.A   --------------------------------------------------------------------------------

Target     QKAFCGFEDPRTKTKMSAAQALKKGWLYYEAGQRFLEVQYLTGGLIEPDTPGRVPLDEALQRGTVDARTAQKLRDVSAYS
4d1e.1.A   --------------------------------------------------------------------------------

Target     KYLTCPKTKLKISYKDALDRSMVEEGTGLRLLEAAAQSSKGYYSPYSVSGSGSTTGSRSGSRTGSRAGSRRGSFDATGSG
4d1e.1.A   --------------------------------------------------------------------------------

Target     FSMTFSSSSYSSSGYGRRYASGPTSSLGGPESAAA
4d1e.1.A   -----------------------------------
```

Model #16	File	Built with	Oligo-State	Ligands	GMQE	QMEANDisCo Global
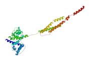	PDB	ProMod3 3.3.0	monomer (matching prediction)	None	0.00	0.64 ± 0.05

Template	Seq Identity	Oligo-state	QSQE	Found by	Method	Resolution	Seq Similarity	Range	Coverage	Description
6sl2.1.A	29.82	homo-dimer	0.00	HHblits	X-ray	3.10Å	0.36	62 - 448	0.08	Calponin homology domain protein putative

Excluded ligands

Ligand Name.Number	Reason for Exclusion	Description
CA.1	Binding site not conserved.	CALCIUM ION
CA.4	Binding site not conserved.	CALCIUM ION
MG.2	Not in contact with model.	MAGNESIUM ION
MG.5	Not in contact with model.	MAGNESIUM ION
TRS.3	Not biologically relevant.	2-AMINO-2-HYDROXYMETHYL-PROPANE-1,3-DIOL
TRS.6	Not biologically relevant.	2-AMINO-2-HYDROXYMETHYL-PROPANE-1,3-DIOL

```
Target     MSGEDQEVRAVVEDGSNGGSGSPSPGDTLPWNLEKTQRSRRGGGGPGGNGSVLDPAERAVIRIADERDRVQKKTFTKWVN
6sl2.1.A   -------------------------------------------------------------ELVAQWEKVQIKTFTKWVN

Target     KHLIKAQRHISDLYEDLRDGHNLISLLEVLSGDSLPR-EKGRMRFHKLQNVQIALDYLRHRQVKLVNIRNDDIADGNPKL
6sl2.1.A   MHLAKKGRKINDVTTDFKNGVELCALLEIIGETTIKCVTNPKMRIQMTENLDKALRFIQSRDVKLTGIGPTDIVDGNVKL

Target     TLGLIWTIILHFQISDIQVSGQSEDMTAKEKLLLWSQRMVEGYQGLRCDNFTSSWRDGRLFNAIIHRHKPMLIDMNKVYR
6sl2.1.A   TLGLVWTLILRFAISELSAE----GLSAKQGLLLWCQKKCEPYP-VKVENFSESFKDGKVFCALIHRHRPDLLDWETVGE

Target     QTNLENLDQAFSVAERDLGVTRLLDPEDVDV-PQPDEKSIITYVSSLYDAMPRVPDVQDGVK--------ANELQLRWQE
6sl2.1.A   D-DRANLEKAFDVAEKELGIPKLLDVDDIVNMPRPDERSVMTYVAALYKVFSSNDQVEKAGKRAGNFLDLLRATEGMVHD

Target     YRELVLLLLQWIRAHTAAFEERRFPSSFEEIEILWCQFLKFKETELPAKEADKNRSKGIYQSLEGAVQAG---QLKVPPG
6sl2.1.A   YEQRAQALKENIEAAINKMNGVEPSDEYHQVKEQINETKNYRKGDKRAFIKEQGDLATLFGQINSKLRGMKRPVYVAPEG

Target     YHPLDVEKEWGKLHVAILEREKQLRSEFERLERLQRIVSKLQMEAGLCEEQLNQADALLQSDVRLLAAGKAPQRAGEVER
6sl2.1.A   LDPKSLEGYIANISEAERALRSKLNTAMR--NCLIALRKAFADPANATDAKINEYRTFVTD-------------------

Target     DLDKADGMIRLLFNDVQALKDGRHPQGEQMYRRVYRLHERLVAIRTEYNLRLRGTPRHPELEDSTLRYLQDLLAWVEENQ
6sl2.1.A   --------------------------------------------------------------------------------

Target     RRVDSAEWGVDLPSVEAQLGSHRGLHQSVEEFRAKIERARTDEGQLSPATRGAYRDCLGRLDLQYAKLLNSSKARLRSLE
6sl2.1.A   --------------------------------------------------------------------------------

Target     SLHGFVAAATKELMWLSEKEEEEVGFDWSERNSNMAAKKEAYSALMRELELKEKKIKEIQSTGDRLLREDHPARPTVESF
6sl2.1.A   --------------------------------------------------------------------------------

Target     QAALQTQWSWMLQLCCCIEAHLKENTAYFQFFSDVREAEEQLRKLQETLHRKYTCDRSITVTRLEDLLQDAQDEKDQLNE
6sl2.1.A   --------------------------------------------------------------------------------
```

```
Target    YRGHLSGLAKRAKAIVQLTPRNPTQPTRGRVPLLAVCDYKQVEATVHKGDECQMLGPAQPFHWKVLSSSGSEAAVPSVCF
6s12.1.A  --------------------------------------------------------------------------------

Target    LVPPPNQEALEAVARLEAQHQALVTLWHQLHTDMKSLLAWQSLSRDVQLIRSWSLVTFRTLKPEEQRQALRSLELHYQAF
6s12.1.A  --------------------------------------------------------------------------------

Target    LRDSQDAGGFGPEDRLQAEREYGSCSRHYQQLLQSLEQGEQEESRCQRCISELKDIRLQLEACETRTVHRLRLPLDKEPA
6s12.1.A  --------------------------------------------------------------------------------

Target    RECAQRIAEQQKAQAEVEGLGKGVARLSAEAEKVLALPEPSPAAPTLRSELELTLGKLEQVRSLSAIYLEKLKTISLVIR
6s12.1.A  --------------------------------------------------------------------------------

Target    STQGAEEALRAHEEQLKEAQAVPAALPELEATKAAMKKLRAQAEAQQPVFDALRDELRGAQEVGERLQQRHGERDVEVER
6s12.1.A  --------------------------------------------------------------------------------

Target    WRERVTQLLERWQAVLAQTDVRQRELEQLGRQLRYYRESADPLGAWLQDARRRQEQIQAVPLADSQAVREQLRQEKALLE
6s12.1.A  --------------------------------------------------------------------------------

Target    EIERHAEKVEECQRFAKQYINAIKDYELQLVTYKAQLEPVASPAKKPKVQSGSESVIQEYVDLRTRYSELSTLTSQYIRF
6s12.1.A  --------------------------------------------------------------------------------

Target    ISETLRRMEEEERLAEQQRAEERERLAEVEAALEKQRQLAEAHAQAKAQAEREAQELQRRMQEEVARREEVAVDAQQQKR
6s12.1.A  --------------------------------------------------------------------------------

Target    SIQEELQQLRQSSEAEIQAKARQVEAAERSRLRIEEEIRVVRLQLETTERQRGGAEGELQALRARAEEAEAQKRQAQEEA
6s12.1.A  --------------------------------------------------------------------------------

Target    ERLRRQVQEETQRKRQAEAELGLRVKAEAEAAREKQRALQALEELRLQAEEAERRLRQAEAERARQVQVALETAQRSAQA
6s12.1.A  --------------------------------------------------------------------------------

Target    ELQSKHASFAEKTAQLERTLEEEHVTVVQLREEATRREQQQAEAERAREEAERELERWQLKANEALRLRLQAEEVAQQKS
6s12.1.A  --------------------------------------------------------------------------------

Target    LAQAEAEKQKEAAEREARRRGKAEEQAVRQRELAEQELERQRQLAEGTAQQRLAAEQELIRLRAETEQGEQQRQLLEEEL
6s12.1.A  --------------------------------------------------------------------------------

Target    ARLQSEAAAATQKRQELEAELAKVRAEMEVLLASKARAEEESRSSSEKSKQRLEAEAGRFRELAEEAARLRALAEEAKRQ
6s12.1.A  --------------------------------------------------------------------------------

Target    RQLAEEDAARQRAEAERVLSEKLAAISEATRLKTEAEIALKEKEAENERLRRLAEDEAFQRRRLEEQAAQHKADIEERLA
6s12.1.A  --------------------------------------------------------------------------------

Target    QLRKASESELERQKGLVEDTLRQRRQVEEEILALKASFEKAAAGKAELELELGRIRGNAEDTLRSKEQAEQEAARQRQLA
6s12.1.A  --------------------------------------------------------------------------------

Target    AEEERRRREAEERVQKSLAAEEEAARQRKAALEEVERLKAKVEEARRLRERAEHESVRQLQLAQEAAQKRLQAEEKAHAF
6s12.1.A  --------------------------------------------------------------------------------

Target    AVQQKEQELQQTLQQEQSVLEKLRSEAEAARRAAEEAEEARERAEREAAQSRQRVEEAERLKQAAEEQAQAQAQAQAAAE
6s12.1.A  --------------------------------------------------------------------------------

Target    KLRKEAEQEAARRAQAEQAALRQKQAADAEMEKHKKFAEQTLRQKAQVEQELTALRLKLEETDHQKSILDQELQRLKAEV
6s12.1.A  --------------------------------------------------------------------------------

Target    TEAARQRSQVEEELFSVRVQMEELGKLKARIEAENRALILRDKDNTQRLLQEEAEKMKQVAEEAARLSVAAQEAARLRQL
6s12.1.A  --------------------------------------------------------------------------------

Target    AEEDLAQQRALAEKMLKEKMQAVQEATRLKAEAELLQQQKELAQEQARRLQEDKEQMAQQLAQETQGFQRTLETERQRQL
6s12.1.A  --------------------------------------------------------------------------------

Target    EMSAEAERLRLRVAEMSRAQARAEEDAQRFRKQAEEIGAKLHRTELATQEKVTLVQTLETQRQQSDRDADRLREAIAELE
6s12.1.A  --------------------------------------------------------------------------------

Target    REKDKLKKEAELLQLKSEEMQTVQQEQLLQETQALQQSFLSEKDSLLQRERFIEEEKAKLERLFQDEVAKAQKLREEQQR
6s12.1.A  --------------------------------------------------------------------------------

Target    QQQQMQQEKQQLLASMEEARRRQHEAEEGVRRKQEELQLLEQQRQQQEQLLAEENRRLRERLEHLEEEHRAALAHSEEIT
6s12.1.A  --------------------------------------------------------------------------------

Target    AAQAAATRALPNGQDAADGPAAEPEHAFEGLRQKVPAQQLQEAGILSTEELQRLAQGHTTVAELTQREDVRRYLQGHSSI
6s12.1.A  --------------------------------------------------------------------------------

Target    AGLLLKPANEKLTIYAALRRQLLSPGTALILLEAQAASGFLLDPVRNRRLTVTEAVKEGVVGPELHHKLLSAERAVTGYK
```

```
6sl2.1.A   --------------------------------------------------------------------------------

Target     DPYTGEKISLFQAMKKDLIVREHGIRLLEAQIATGGVIDPVHSHRVPVDVAYHRGYFDEEMNRVLQDPSDDTKGFFDPNT
6sl2.1.A   --------------------------------------------------------------------------------

Target     HENLTYLQLLERCVEDPETGLRLLPLTDQAAKGGELVYTDSEARDVFEKATVSAPFGKFQGKTVTIWELINSEYFTAEQR
6sl2.1.A   --------------------------------------------------------------------------------

Target     RDLLRQFRTGKVTVEKIIKIVITVIEEHEQKGQLCFQGLRALVPAAELLESGIIDWDLFRQLQLGERSVQEVAEVEGVRR
6sl2.1.A   --------------------------------------------------------------------------------

Target     ALRGSGVIAGVWLEEARQKLSIYEALKKELLQPEAAVALLEAQAGTGHVIDPATSARLTVDEAVRAGLVGPELHEKLLSA
6sl2.1.A   --------------------------------------------------------------------------------

Target     EKAVTGYKDPYSGQSVSLFQALKKGLIPREQGLRLLDAQLSTGGTVDPSKSHRLPLDVACARGYLDEETSTALSAPRDDA
6sl2.1.A   --------------------------------------------------------------------------------

Target     KTYYDPRTWELATYSQLQQQCRPDPLTGLSLLPLSEEAARARQQELYSEVQAREAFQKATVEVPVGSFQGRAVTIWELIN
6sl2.1.A   --------------------------------------------------------------------------------

Target     SEYFTAEQRQELLRQFRTGKVTVEKIIKIVITIVEEVETTRRERLSFSGLRAPVPASELLAAGILSSSQFEQLKDGKTSV
6sl2.1.A   --------------------------------------------------------------------------------

Target     KDLSELSSLQTLLQGSGCLAGIYLEESKEKVTIYEAMRRGLLRPSTAILLLEAQAATGFLVDPVRNQRLYVHEAVKAGVV
6sl2.1.A   --------------------------------------------------------------------------------

Target     GPELHEKLLSAEKAVTGYKDPYSGSTISLFQAMKKGLVVREHGIRLLEAQIATGGIIDPVHSHRVPVDVACQRGYFDEEM
6sl2.1.A   --------------------------------------------------------------------------------

Target     NRVLEDPSDDTKGFFDPNTHENLTYRQLLERCVEDPETGLRLLPLKGPEKAEVVETTRVYTEEETRRAFEETQIDIPGGG
6sl2.1.A   --------------------------------------------------------------------------------

Target     SHGGSTMSLWEVMQSDLIPEEQRAQLMADFQAGRVTKERMIIIIIEIIEKTEIVRQQNLASYDYVRRRLTAEDLYEARVI
6sl2.1.A   --------------------------------------------------------------------------------

Target     SRESYSLLREGTRSLREVLEAESAWRYLYGTGCVAGVYLPGSRQTLPIYQALKKGLLSAEVARLLLEAQAATGFLLDPVK
6sl2.1.A   --------------------------------------------------------------------------------

Target     GERLTVDEAVRKGLVGPELHDRLLSAERAVTGYRDPYTEQTISLFQAMKKDLIPAEEALRLLDAQLATGGIVDPRLGFHL
6sl2.1.A   --------------------------------------------------------------------------------

Target     PLEVAYQRGYLNKDTHDQLSEPSEVRSYVDPSTDERLSYTQLLRRCRRDETSGLFLLPLSEARKLTFRGLRKQITVEELV
6sl2.1.A   --------------------------------------------------------------------------------

Target     RSHVMDEATAQRLQEGLTSIEEVSKNLQKFLEGTSSIAGVLVDATKERLSVYQAMKKGIIRPGTAFELLEAQAATGYVID
6sl2.1.A   --------------------------------------------------------------------------------

Target     PIKGLKLTVEEAVRMGIVGPEFKDKLLSAERAVTGYKDPYSGKLISLFQAMKKGLILKDHGIRLLEAQIATGGIIDPEES
6sl2.1.A   --------------------------------------------------------------------------------

Target     HRLPVDVAYQRGLFDEEMNEILTDPSDDTKGFFDPNTEENLTYLQLMERCVTDPQTGLRLLPLKEKKRERKTSSKSSVRK
6sl2.1.A   --------------------------------------------------------------------------------

Target     RRVVIVDPETGKEMSVYEAYRKGLIDHQTYLELSEQECEWEEITISSSDGVVKSMIIDRRSGRQYDIDEAIAKSLIDRSA
6sl2.1.A   --------------------------------------------------------------------------------

Target     LDQYRAGTLSITEFADMLSGNAGGFRSRSSSVGSSSSYPISPAVSRTQLASWSDPTEETGPVAGILDTETLEKVSITEAM
6sl2.1.A   --------------------------------------------------------------------------------

Target     HRNLVDNITGQRLLEAQACTGGIIDPNTGERFPVTDAVNKGLVDKIMVDRINLAQKAFCGFEDPRTKTKMSAAQALKKGW
6sl2.1.A   --------------------------------------------------------------------------------

Target     LYYEAGQRFLEVQYLTGGLIEPDTPGRVPLDEALQRGTVDARTAQKLRDVSAYSKYLTCPKTKLKISYKDALDRSMVEEG
6sl2.1.A   --------------------------------------------------------------------------------

Target     TGLRLLEAAAQSSKGYYSPYSVSGSGSTTGSRSGSRTGSRAGSRRGSFDATGSGFSMTFSSSSYSSSGYGRRYASGPTSS
6sl2.1.A   --------------------------------------------------------------------------------

Target     LGGPESAAA
6sl2.1.A   ---------
```

Model #11	File	Built with	Oligo-State	Ligands	GMQE	QMEANDisCo Global
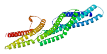	PDB	ProMod3 3.3.0	monomer	None	0.00	0.69 ± 0.05

Template	Seq Identity	Oligo-state	QSQE	Found by	Method	Resolution	Seq Similarity	Range	Coverage	Description
5j1i.1.A	96.47	monomer	0.00	HHblits	X-ray	2.80Å	0.59	991 - 1351	0.08	Plectin

The template contained no ligands.

```
Target     MSGEDQEVRAVVEDGSNGGSGSPSPGDTLPWNLEKTQRSRRGGGGPGGNGSVLDPAERAVIRIADERDRVQKKTFTKWVN
5j1i.1.A   --------------------------------------------------------------------------------

Target     KHLIKAQRHISDLYEDLRDGHNLISLLEVLSGDSLPREKGRMRFHKLQNVQIALDYLRHRQVKLVNIRNDDIADGNPKLT
5j1i.1.A   --------------------------------------------------------------------------------

Target     LGLIWTIILHFQISDIQVSGQSEDMTAKEKLLLWSQRMVEGYQGLRCDNFTSSWRDGRLFNAIIHRHKPMLIDMNKVYRQ
5j1i.1.A   --------------------------------------------------------------------------------

Target     TNLENLDQAFSVAERDLGVTRLLDPEDVDVPQPDEKSIITYVSSLYDAMPRVPDVQDGVKANELQLRWQEYRELVLLLLQ
5j1i.1.A   --------------------------------------------------------------------------------

Target     WIRAHTAAFEERRFPSSFEEIEILWCQFLKFKETELPAKEADKNRSKGIYQSLEGAVQAGQLKVPPGYHPLDVEKEWGKL
5j1i.1.A   --------------------------------------------------------------------------------

Target     HVAILEREKQLRSEFERLERLQRIVSKLQMEAGLCEEQLNQADALLQSDVRLLAAGKAPQRAGEVERDLDKADGMIRLLF
5j1i.1.A   --------------------------------------------------------------------------------

Target     NDVQALKDGRHPQGEQMYRRVYRLHERLVAIRTEYNLRLRGTPRHPELEDSTLRYLQDLLAWVEENQRRVDSAEWGVDLP
5j1i.1.A   --------------------------------------------------------------------------------

Target     SVEAQLGSHRGLHQSVEEFRAKIERARTDEGQLSPATRGAYRDCLGRLDLQYAKLLNSSKARLRSLESLHGFVAAATKEL
5j1i.1.A   --------------------------------------------------------------------------------

Target     MWLSEKEEEEVGFDWSERNSNMAAKKEAYSALMRELELKEKKIKEIQSTGDRLLREDHPARPTVESFQAALQTQWSWMLQ
5j1i.1.A   --------------------------------------------------------------------------------

Target     LCCCIEAHLKENTAYFQFFSDVREAEEQLRKLQETLHRKYTCDRSITVTRLEDLLQDAQDEKDQLNEYRGHLSGLAKRAK
5j1i.1.A   --------------------------------------------------------------------------------

Target     AIVQLTPRNPTQPTRGRVPLLAVCDYKQVEATVHKGDECQMLGPAQPFHWKVLSSSGSEAAVPSVCFLVPPPNQEALEAV
5j1i.1.A   --------------------------------------------------------------------------------

Target     ARLEAQHQALVTLWHQLHTDMKSLLAWQSLSRDVQLIRSWSLVTFRTLKPEEQRQALRSLELHYQAFLRDSQDAGGFGPE
5j1i.1.A   --------------------------------------------------------------------------------

Target     DRLQAEREYGSCSRHYQQLLQSLEQGEQEESRCQRCISELKDIRLQLEACETRTVHRLRLPLDKEPARECAQRIAEQQKA
5j1i.1.A   ---------------------------QEESRCQRCISELKDIRLQLEACETRTVHRLRLPLDKEPARECAQRIAEQQKA

Target     QAEVEGLGKGVARLSAEAEKVLALPEPSPAAPTLRSELELTLGKLEQVRSLSAIYLEKLKTISLVIRSTQGAEEALRAHE
5j1i.1.A   QAEVEGLGKGVARLSAEAEKVLALPEPSPAAPTLRSELELTLGKLEQVRSLSAIYLEKLKTISLVIRGTQGAEEVLRAHE

Target     EQLKEAQAVPAALPELEATKAAMKKLRAQAEAQQPVFDALRDELRGAQEVGERLQQRHGERDVEVERWRERVTQLLERWQ
5j1i.1.A   EQLKEAQAVPATLPELEATKASLKKLRAQAEAQQPTFDALRDELRGAQEVGERLQQRHGERDVEVERWRERVAQLLERWQ

Target     AVLAQTDVRQRELEQLGRQLRYYRESADPLGAWLQDARRRQEQIQAVPLADSQAVREQLRQEKALLEEIERHAEKVEECQ
5j1i.1.A   AVLAQTDVRQRELEQLGRQLRYYRESADPLGAWLQDARRRQEQIQAMPLADSQAVREQLRQEQALLEEIERHGEKVEECQ

Target     RFAKQYINAIKDYELQLVTYKAQLEPVASPAKKPKVQSGSESVIQEYVDLRTRYSELSTLTSQYIRFISETLRRMEEEER
5j1i.1.A   RFAKQYINAIKDYELQLVTYKAQLEPVASPAKKPKVQSGSESVIQEYVDLRTHYSELTTLTSQYIKFISETLRRM-----

Target     LAEQQRAEERERLAEVEAALEKQRQLAEAHAQAKAQAEREAQELQRRMQEEVARREEVAVDAQQQKRSIQEELQQLRQSS
5j1i.1.A   --------------------------------------------------------------------------------

Target     EAEIQAKARQVEAAERSRLRIEEEIRVVRLQLETTERQRGGAEGELQALRARAEEAEAQKRQAQEEAERLRRQVQEETQR
5j1i.1.A   --------------------------------------------------------------------------------
```

```
Target     KRQAEAELGLRVKAEAEAAREKQRALQALEELRLQAEEAERRLRQAEAERARQVQVALETAQRSAQAELQSKHASFAEKT
5jli.1.A   --------------------------------------------------------------------------------

Target     AQLERTLEEEHVTVVQLREEATRREQQQAEAERAREEAERELERWQLKANEALRLRLQAEEVAQQKSLAQAEAEKQKEAA
5jli.1.A   --------------------------------------------------------------------------------

Target     EREARRRGKAEEQAVRQRELAEQELERQRQLAEGTAQQRLAAEQELIRLRAETEQGEQQRQLLEEELARLQSEAAAATQK
5jli.1.A   --------------------------------------------------------------------------------

Target     RQELEAELAKVRAEMEVLLASKARAEEESRSSSEKSKQRLEAEAGRFRELAEEAARLRALAEEAKRQRQLAEEDAARQRA
5jli.1.A   --------------------------------------------------------------------------------

Target     EAERVLSEKLAAISEATRLKTEAEIALKEKEAENERLRRLAEDEAFQRRRLEEQAAQHKADIEERLAQLRKASESELERQ
5jli.1.A   --------------------------------------------------------------------------------

Target     KGLVEDTLRQRRQVEEEILALKASFEKAAAGKAELELELGRIRGNAEDTLRSKEQAEQEAARQRQLAAEEERRRREAEER
5jli.1.A   --------------------------------------------------------------------------------

Target     VQKSLAAEEEAARQRKAALEEVERLKAKVEEARRLRERAEHESVRQLQLAQEAAQKRLQAEEKAHAFAVQQKEQELQQTL
5jli.1.A   --------------------------------------------------------------------------------

Target     QQEQSVLEKLRSEAEAARRAAEEAEEARERAEREAAQSRQRVEEAERLKQAAEEQAQAQAQAQAAAEKLRKEAEQEAARR
5jli.1.A   --------------------------------------------------------------------------------

Target     AQAEQAALRQKQAADAEMEKHKKFAEQTLRQKAQVEQELTALRLKLEETDHQKSILDQELQRLKAEVTEAARQRSQVEEE
5jli.1.A   --------------------------------------------------------------------------------

Target     LFSVRVQMEELGKLKARIEAENRALILRDKDNTQRLLQEEAEKMKQVAEEAARLSVAAQEAARLRQLAEEDLAQQRALAE
5jli.1.A   --------------------------------------------------------------------------------

Target     KMLKEKMQAVQEATRLKAEAELLQQQKELAQEQARRLQEDKEQMAQQLAQETQGFQRTLETERQRQLEMSAEAERLRLRV
5jli.1.A   --------------------------------------------------------------------------------

Target     AEMSRAQARAEEDAQRFRKQAEEIGAKLHRTELATQEKVTLVQTLETQRQQSDRDADRLREAIAELEREKDKLKKEAELL
5jli.1.A   --------------------------------------------------------------------------------

Target     QLKSEEMQTVQQEQLLQETQALQQSFLSEKDSLLQRERFIEEEKAKLERLFQDEVAKAQKLREEQQRQQQMQQEKQQLL
5jli.1.A   --------------------------------------------------------------------------------

Target     ASMEEARRRQHEAEEGVRRKQEELQLLEQQRQQQEQLLAEENRRLRERLEHLEEEHRAALAHSEEITAAQAAATRALPNG
5jli.1.A   --------------------------------------------------------------------------------

Target     QDAADGPAAEPEHAFEGLRQKVPAQQLQEAGILSTEELQRLAQGHTTVAELTQREDVRRYLQGHSSIAGLLLKPANEKLT
5jli.1.A   --------------------------------------------------------------------------------

Target     IYAALRRQLLSPGTALILLEAQAASGFLLDPVRNRRLTVTEAVKEGVVGPELHHKLLSAERAVTGYKDPYTGEKISLFQA
5jli.1.A   --------------------------------------------------------------------------------

Target     MKKDLIVREHGIRLLEAQIATGGVIDPVHSHRVPVDVAYHRGYFDEEMNRVLQDPSDDTKGFFDPNTHENLTYLQLLERC
5jli.1.A   --------------------------------------------------------------------------------

Target     VEDPETGLRLLPLTDQAAKGGELVYTDSEARDVFEKATVSAPFGKFQGKTVTIWELINSEYFTAEQRRDLLRQFRTGKVT
5jli.1.A   --------------------------------------------------------------------------------

Target     VEKIIKIVITVIEEHEQKGQLCFQGLRALVPAAELLESGIIDWDLFRQLQLGERSVQEVAEVEGVRRALRGSGVIAGVWL
5jli.1.A   --------------------------------------------------------------------------------

Target     EEARQKLSIYEALKKELLQPEAAVALLEAQAGTGHVIDPATSARLTVDEAVRAGLVGPELHEKLLSAEKAVTGYKDPYSG
5jli.1.A   --------------------------------------------------------------------------------

Target     QSVSLFQALKKGLIPREQGLRLLDAQLSTGGTVDPSKSHRLPLDVACARGYLDEETSTALSAPRDDAKTYYDPRTWELAT
5jli.1.A   --------------------------------------------------------------------------------

Target     YSQLQQQCRPDPLTGLSLLPLSEEAARARQQELYSEVQAREAFQKATVEVPVGSFQGRAVTIWELINSEYFTAEQRQELL
5jli.1.A   --------------------------------------------------------------------------------

Target     RQFRTGKVTVEKIIKIVITIVEEVETTRRERLSFSGLRAPVPASELLAAGILSSSQFEQLKDGKTSVKDLSELSSLQTLL
5jli.1.A   --------------------------------------------------------------------------------

Target     QGSGCLAGIYLEESKEKVTIYEAMRRGLLRPSTAILLLEAQAATGFLVDPVRNQRLYVHEAVKAGVVGPELHEKLLSAEK
5jli.1.A   --------------------------------------------------------------------------------

Target     AVTGYKDPYSGSTISLFQAMKKGLVVREHGIRLLEAQIATGGIIDPVHSHRVPVDVACQRGYFDEEMNRVLEDPSDDTKG
```

```
5jli.1.A   --------------------------------------------------------------------------------

Target     FFDPNTHENLTYRQLLERCVEDPETGLRLLPLKGPEKAEVVETTRVYTEEETRRAFEETQIDIPGGGSHGGSTMSLWEVM
5jli.1.A   --------------------------------------------------------------------------------

Target     QSDLIPEEQRAQLMADFQAGRVTKERMIIIIIEIIEKTEIVRQQNLASYDYVRRRLTAEDLYEARVISRESYSLLREGTR
5jli.1.A   --------------------------------------------------------------------------------

Target     SLREVLEAESAWRYLYGTGCVAGVYLPGSRQTLPIYQALKKGLLSAEVARLLLEAQAATGFLLDPVKGERLTVDEAVRKG
5jli.1.A   --------------------------------------------------------------------------------

Target     LVGPELHDRLLSAERAVTGYRDPYTEQTISLFQAMKKDLIPAEEALRLLDAQLATGGIVDPRLGFHLPLEVAYQRGYLNK
5jli.1.A   --------------------------------------------------------------------------------

Target     DTHDQLSEPSEVRSYVDPSTDERLSYTQLLRRCRRDETSGLFLLPLSEARKLTFRGLRKQITVEELVRSHVMDEATAQRL
5jli.1.A   --------------------------------------------------------------------------------

Target     QEGLTSIEEVSKNLQKFLEGTSSIAGVLVDATKERLSVYQAMKKGIIRPGTAFELLEAQAATGYVIDPIKGLKLTVEEAV
5jli.1.A   --------------------------------------------------------------------------------

Target     RMGIVGPEFKDKLLSAERAVTGYKDPYSGKLISLFQAMKKGLILKDHGIRLLEAQIATGGIIDPEESHRLPVDVAYQRGL
5jli.1.A   --------------------------------------------------------------------------------

Target     FDEEMNEILTDPSDDTKGFFDPNTEENLTYLQLMERCVTDPQTGLRLLPLKEKKRERKTSSKSSVRKRRVVIVDPETGKE
5jli.1.A   --------------------------------------------------------------------------------

Target     MSVYEAYRKGLIDHQTYLELSEQECEWEEITISSSDGVVKSMIIDRRSGRQYDIDEAIAKSLIDRSALDQYRAGTLSITE
5jli.1.A   --------------------------------------------------------------------------------

Target     FADMLSGNAGGFRSRSSSVGSSSSYPISPAVSRTQLASWSDPTEETGPVAGILDTETLEKVSITEAMHRNLVDNITGQRL
5jli.1.A   --------------------------------------------------------------------------------

Target     LEAQACTGGIIDPNTGERFPVTDAVNKGLVDKIMVDRINLAQKAFCGFEDPRTKTKMSAAQALKKGWLYYEAGQRFLEVQ
5jli.1.A   --------------------------------------------------------------------------------

Target     YLTGGLIEPDTPGRVPLDEALQRGTVDARTAQKLRDVSAYSKYLTCPKTKLKISYKDALDRSMVEEGTGLRLLEAAAQSS
5jli.1.A   --------------------------------------------------------------------------------

Target     KGYYSPYSVSGSGSTTGSRSGSRTGSRAGSRRGSFDATGSGFSMTFSSSSYSSSGYGRRYASGPTSSLGGPESAAA
5jli.1.A   ----------------------------------------------------------------------------
```

Model #04	File	Built with	Oligo-State	Ligands	GMQE	QMEANDisCo Global
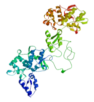	PDB	ProMod3 3.3.0	monomer	None	0.00	0.65 ± 0.05

Template	Seq Identity	Oligo-state	QSQE	Found by	Method	Resolution	Seq Similarity	Range	Coverage	Description
5dzz.1.A	54.12	monomer	0.00	HHblits	X-ray	2.60Å	0.45	2977 - 3553	0.11	Desmoplakin

The template contained no ligands.

```
Target     MSGEDQEVRAVVEDGSNGGSGSPSPGDTLPWNLEKTQRSRRGGGGPGGNGSVLDPAERAVIRIADERDRVQKKTFTKWVN
5dzz.1.A   --------------------------------------------------------------------------------

Target     KHLIKAQRHISDLYEDLRDGHNLISLLEVLSGDSLPREKGRMRFHKLQNVQIALDYLRHRQVKLVNIRNDDIADGNPKLT
5dzz.1.A   --------------------------------------------------------------------------------

Target     LGLIWTIILHFQISDIQVSGQSEDMTAKEKLLLWSQRMVEGYQGLRCDNFTSSWRDGRLFNAIIHRHKPMLIDMNKVYRQ
5dzz.1.A   --------------------------------------------------------------------------------

Target     TNLENLDQAFSVAERDLGVTRLLDPEDVDVPQPDEKSIITYVSSLYDAMPRVPDVQDGVKANELQLRWQEYRELVLLLLQ
5dzz.1.A   --------------------------------------------------------------------------------

Target     WIRAHTAAFEERRFPSSFEEIEILWCQFLKFKETELPAKEADKNRSKGIYQSLEGAVQAGQLKVPPGYHPLDVEKEWGKL
```

```
5dzz.1.A    --------------------------------------------------------------------------------

Target      HVAILEREKQLRSEFERLERLQRIVSKLQMEAGLCEEQLNQADALLQSDVRLLAAGKAPQRAGEVERDLDKADGMIRLLF
5dzz.1.A    --------------------------------------------------------------------------------

Target      NDVQALKDGRHPQGEQMYRRVYRLHERLVAIRTEYNLRLRGTPRHPELEDSTLRYLQDLLAWVEENQRRVDSAEWGVDLP
5dzz.1.A    --------------------------------------------------------------------------------

Target      SVEAQLGSHRGLHQSVEEFRAKIERARTDEGQLSPATRGAYRDCLGRLDLQYAKLLNSSKARLRSLESLHGFVAAATKEL
5dzz.1.A    --------------------------------------------------------------------------------

Target      MWLSEKEEEEVGFDWSERNSNMAAKKEAYSALMRELELKEKKIKEIQSTGDRLLREDHPARPTVESFQAALQTQWSWMLQ
5dzz.1.A    --------------------------------------------------------------------------------

Target      LCCCIEAHLKENTAYFQFFSDVREAEEQLRKLQETLHRKYTCDRSITVTRLEDLLQDAQDEKDQLNEYRGHLSGLAKRAK
5dzz.1.A    --------------------------------------------------------------------------------

Target      AIVQLTPRNPTQPTRGRVPLLAVCDYKQVEATVHKGDECQMLGPAQPFHWKVLSSSGSEAAVPSVCFLVPPPNQEALEAV
5dzz.1.A    --------------------------------------------------------------------------------

Target      ARLEAQHQALVTLWHQLHTDMKSLLAWQSLSRDVQLIRSWSLVTFRTLKPEEQRQALRSLELHYQAFLRDSQDAGGFGPE
5dzz.1.A    --------------------------------------------------------------------------------

Target      DRLQAEREYGSCSRHYQQLLQSLEQGEQEESRCQRCISELKDIRLQLEACETRTVHRLRLPLDKEPARECAQRIAEQQKA
5dzz.1.A    --------------------------------------------------------------------------------

Target      QAEVEGLGKGVARLSAEAEKVLALPEPSPAAPTLRSELELTLGKLEQVRSLSAIYLEKLKTISLVIRSTQGAEEALRAHE
5dzz.1.A    --------------------------------------------------------------------------------

Target      EQLKEAQAVPAALPELEATKAAMKKLRAQAEAQQPVFDALRDELRGAQEVGERLQQRHGERDVEVERWRERVTQLLERWQ
5dzz.1.A    --------------------------------------------------------------------------------

Target      AVLAQTDVRQRELEQLGRQLRYYRESADPLGAWLQDARRRQEQIQAVPLADSQAVREQLRQEKALLEEIERHAEKVEECQ
5dzz.1.A    --------------------------------------------------------------------------------

Target      RFAKQYINAIKDYELQLVTYKAQLEPVASPAKKPKVQSGSESVIQEYVDLRTRYSELSTLTSQYIRFISETLRRMEEEER
5dzz.1.A    --------------------------------------------------------------------------------

Target      LAEQQRAEERERLAEVEAALEKQRQLAEAHAQAKAQAEREAQELQRRMQEEVARREEVAVDAQQQKRSIQEELQQLRQSS
5dzz.1.A    --------------------------------------------------------------------------------

Target      EAEIQAKARQVEAAERSRLRIEEEIRVVRLQLETTERQRGGAEGELQALRARAEEAEAQKRQAQEEAERLRRQVQEETQR
5dzz.1.A    --------------------------------------------------------------------------------

Target      KRQAEAELGLRVKAEAEAAREKQRALQALEELRLQAEEAERRLRQAEAERARQVQVALETAQRSAQAELQSKHASFAEKT
5dzz.1.A    --------------------------------------------------------------------------------

Target      AQLERTLEEEHVTVVQLREEATRREQQQAEAERAREEAERELERWQLKANEALRLRLQAEEVAQQKSLAQAEAEKQKEAA
5dzz.1.A    --------------------------------------------------------------------------------

Target      EREARRRGKAEEQAVRQRELAEQELERQRQLAEGTAQQRLAAEQELIRLRAETEQGEQQRQLLEEELARLQSEAAAATQK
5dzz.1.A    --------------------------------------------------------------------------------

Target      RQELEAELAKVRAEMEVLLASKARAEEESRSSSEKSKQRLEAEAGRFRELAEEAARLRALAEEAKRQRQLAEEDAARQRA
5dzz.1.A    --------------------------------------------------------------------------------

Target      EAERVLSEKLAAISEATRLKTEAEIALKEKEAENERLRRLAEDEAFQRRRLEEQAAQHKADIEERLAQLRKASESELERQ
5dzz.1.A    --------------------------------------------------------------------------------

Target      KGLVEDTLRQRRQVEEEILALKASFEKAAAGKAELELELGRIRGNAEDTLRSKEQAEQEAARQRQLAAEEERRRREAEER
5dzz.1.A    --------------------------------------------------------------------------------

Target      VQKSLAAEEEAARQRKAALEEVERLKAKVEEARRLRERAEHESVRQLQLAQEAAQKRLQAEEKAHAFAVQQKEQELQQTL
5dzz.1.A    --------------------------------------------------------------------------------

Target      QQEQSVLEKLRSEAEAARRAAEEAEEARERAEREAAQSRQRVEEAERLKQAAEEQAQAQAQAQAAAEKLRKEAEQEAARR
5dzz.1.A    --------------------------------------------------------------------------------

Target      AQAEQAALRQKQAADAEMEKHKKFAEQTLRQKAQVEQELTALRLKLEETDHQKSILDQELQRLKAEVTEAARQRSQVEEE
5dzz.1.A    --------------------------------------------------------------------------------

Target      LFSVRVQMEELGKLKARIEAENRALILRDKDNTQRLLQEEAEKMKQVAEEAARLSVAAQEAARLRQLAEEDLAQQRALAE
5dzz.1.A    --------------------------------------------------------------------------------
```

```
Target     KMLKEKMQAVQEATRLKAEAELLQQQKELAQEQARRLQEDKEQMAQQLAQETQGFQRTLETERQRQLEMSAEAERLRLRV
5dzz.1.A   --------------------------------------------------------------------------------

Target     AEMSRAQARAEEDAQRFRKQAEEIGAKLHRTELATQEKVTLVQTLETQRQQSDRDADRLREAIAELEREKDKLKKEAELL
5dzz.1.A   --------------------------------------------------------------------------------

Target     QLKSEEMQTVQQEQLLQETQALQQSFLSEKDSLLQRERFIEEEKAKLERLFQDEVAKAQKLREEQQRQQQQMQQEKQQLL
5dzz.1.A   --------------------------------------------------------------------------------

Target     ASMEEARRRQHEAEEGVRRKQEELQLLEQQRQQQEQLLAEENRRLRERLEHLEEEHRAALAHSEEITAAQAAATRALPNG
5dzz.1.A   --------------------------------------------------------------------------------

Target     QDAADGPAAEPEHAFEGLRQKVPAQQLQEAGILSTEELQRLAQGHTTVAELTQREDVRRYLQGHSSIAGLLLKPANEKLT
5dzz.1.A   --------------------------------------------------------------------------------

Target     IYAALRRQLLSPGTALILLEAQAASGFLLDPVRNRRLTVTEAVKEGVVGPELHHKLLSAERAVTGYKDPYTGEKISLFQA
5dzz.1.A   --------------------------------------------------------------------------------

Target     MKKDLIVREHGIRLLEAQIATGGVIDPVHSHRVPVDVAYHRGYFDEEMNRVLQDPSDDTKGFFDPNTHENLTYLQLLERC
5dzz.1.A   --------------------------------------------------------------------------------

Target     VEDPETGLRLLPLTDQAAKGGELVYTDSEARDVFEKATVSAPFGKFQGKTVTIWELINSEYFTAEQRRDLLRQFRTGKVT
5dzz.1.A   --------------------------------------------------------------------------------

Target     VEKIIKIVITVIEEHEQKGQLCFQGLRALVPAAELLESGIIDWDLFRQLQLGERSVQEVAEVEGVRRALRGSGVIAGVWL
5dzz.1.A   ----------------DTSKLVFDGLRKKVTAMQLYECQLIDKTTLDKLLKGKKSVEEVA--SEIQPFLRGAGSIAGAS-

Target     EEARQKLSIYEALKKELLQPEAAVALLEAQAGTGHVIDPATSARLTVDEAVRAGLVGPELHEKLLSAEKAVTGYKDPYSG
5dzz.1.A   ASPKEKYSLVEAKRKKLISPESTVMLLEAQAATGGIIDPHRNEKLTVDSAIARDLIDFDDRQQIYAAEKAITGFDDPFSG

Target     QSVSLFQALKKGLIPREQGLRLLDAQLSTGGTVDPSKSHRLPLDVACARGYLDEETSTALSAPRDDAKTYYDPRTWELAT
5dzz.1.A   KTVSVSEAIKKNLIDRETGMRLLEAQIASGGVVDPVNSVFLPKDVALARGLIDRDLYRSLNDPRDSQKNFVDPVTKKKVS

Target     YSQLQQQCRPDPLTGLSLLPLSEEAARARQQELYSEVQAREAFQKATVEVPVGSFQGRAVTIWELINSEYFTAEQRQELL
5dzz.1.A   YVQLKERCRIEPHTGLLLLSVQKRS-------------------------------------------------------

Target     RQFRTGKVTVEKIIKIVITIVEEVETTRRERLSFSGLRAPVPASELLAAGILSSSQFEQLKDGKTSVKDLSELSSLQTLL
5dzz.1.A   -------------------------------MSFQGIRQPVTVTELVDSGILRPSTVNELESGQISYDEVG--ERIKDFL

Target     QGSGCLAGIYLEESKEKVTIYEAMRRGLLRPSTAILLLEAQAATGFLVDPVRNQRLYVHEAVKAGVVGPELHEKLLSAEK
5dzz.1.A   QGSSCIAGIYNETTKQKLGIYEAMKIGLVRPGTALELLEAQAATGFIVDPVSNLRLPVEEAYKRGLVGIEFKEKLLSAER

Target     AVTGYKDPYSGSTISLFQAMKKGLVVREHGIRLLEAQIATGGIIDPVHSHRVPVDVACQRGYFDEEMNRVLEDPSDDTKG
5dzz.1.A   AVTGYNDPETGNIISLFQAMNKELIEKGHGIRLLEAQIATGGIIDPKESHRLPVDIAYKRGYFNEELSEILSDPSDDTKG

Target     FFDPNTHENLTYRQLLERCVEDPETGLRLLPLKGPEKAEVVETTRVYTEEETRRAFEETQIDIPGGGSHGGSTMSLWEVM
5dzz.1.A   FFDPNTEENLTYLQLKERCIKDEETGLCLLPLK-----------------------------------------------

Target     QSDLIPEEQRAQLMADFQAGRVTKERMIIIIIEIIEKTEIVRQQNLASYDYVRRRLTAEDLYEARVISRESYSLLREGTR
5dzz.1.A   --------------------------------------------------------------------------------

Target     SLREVLEAESAWRYLYGTGCVAGVYLPGSRQTLPIYQALKKGLLSAEVARLLLEAQAATGFLLDPVKGERLTVDEAVRKG
5dzz.1.A   --------------------------------------------------------------------------------

Target     LVGPELHDRLLSAERAVTGYRDPYTEQTISLFQAMKKDLIPAEEALRLLDAQLATGGIVDPRLGFHLPLEVAYQRGYLNK
5dzz.1.A   --------------------------------------------------------------------------------

Target     DTHDQLSEPSEVRSYVDPSTDERLSYTQLLRRCRRDETSGLFLLPLSEARKLTFRGLRKQITVEELVRSHVMDEATAQRL
5dzz.1.A   --------------------------------------------------------------------------------

Target     QEGLTSIEEVSKNLQKFLEGTSSIAGVLVDATKERLSVYQAMKKGIIRPGTAFELLEAQAATGYVIDPIKGLKLTVEEAV
5dzz.1.A   --------------------------------------------------------------------------------

Target     RMGIVGPEFKDKLLSAERAVTGYKDPYSGKLISLFQAMKKGLILKDHGIRLLEAQIATGGIIDPEESHRLPVDVAYQRGL
5dzz.1.A   --------------------------------------------------------------------------------

Target     FDEEMNEILTDPSDDTKGFFDPNTEENLTYLQLMERCVTDPQTGLRLLPLKEKKRERKTSSKSSVRKRRVVIVDPETGKE
5dzz.1.A   --------------------------------------------------------------------------------

Target     MSVYEAYRKGLIDHQTYLELSEQECEWEEITISSSDGVVKSMIIDRRSGRQYDIDEAIAKSLIDRSALDQYRAGTLSITE
5dzz.1.A   --------------------------------------------------------------------------------

Target     FADMLSGNAGGFRSRSSSVGSSSSYPISPAVSRTQLASWSDPTEETGPVAGILDTETLEKVSITEAMHRNLVDNITGQRL
5dzz.1.A   --------------------------------------------------------------------------------
```

```
Target     LEAQACTGGIIDPNTGERFPVTDAVNKGLVDKIMVDRINLAQKAFCGFEDPRTKTKMSAAQALKKGWLYYEAGQRFLEVQ
5dzz.1.A   --------------------------------------------------------------------------------

Target     YLTGGLIEPDTPGRVPLDEALQRGTVDARTAQKLRDVSAYSKYLTCPKTKLKISYKDALDRSMVEEGTGLRLLEAAAQSS
5dzz.1.A   --------------------------------------------------------------------------------

Target     KGYYSPYSVSGSGSTTGSRSGSRTGSRAGSRRGSFDATGSGFSMTFSSSSYSSSGYGRRYASGPTSSLGGPESAAA
5dzz.1.A   ----------------------------------------------------------------------------
```

Model #12	File	Built with	Oligo-State	Ligands	GMQE	QMEANDisCo Global
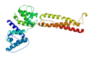	PDB	ProMod3 3.3.0	monomer	None	0.00	0.75 ± 0.05

Template	Seq Identity	Oligo-state	QSQE	Found by	Method	Resolution	Seq Similarity	Range	Coverage	Description
4z6g.1.A	66.86	monomer	0.00	HHblits	X-ray	2.65Å	0.51	65 - 412	0.08	Microtubule-actin cross-linking factor 1, isoforms 1/2/3/5

Excluded ligands

Ligand Name.Number	Reason for Exclusion	Description
PO4.1	Not biologically relevant.	PHOSPHATE ION
PO4.2	Not biologically relevant.	PHOSPHATE ION

```
Target     MSGEDQEVRAVVEDGSNGGSGSPSPGDTLPWNLEKTQRSRRGGGGPGGNGSVLDPAERAVIRIADERDRVQKKTFTKWVN
4z6g.1.A   ----------------------------------------------------------------DERDRVQKKTFTKWVN

Target     KHLIKAQRHISDLYEDLRDGHNLISLLEVLSGDSLPREKGRMRFHKLQNVQIALDYLRHRQVKLVNIRNDDIADGNPKLT
4z6g.1.A   KHLMKVRKHINDLYEDLRDGHNLISLLEVLSGIKLPREKGRMRFHRLQNVQIALDFLKQRQVKLVNIRNDDITDGNPKLT

Target     LGLIWTIILHFQISDIQVSGQSEDMTAKEKLLLWSQRMVEGYQGLRCDNFTSSWRDGRLFNAIIHRHKPMLIDMNKVYRQ
4z6g.1.A   LGLIWTIILHFQISDIYISGESGDMSAKEKLLLWTQKVTAGYTGIKCTNFSSCWSDGKMFNALIHRYRPDLVDMERVQIQ

Target     TNLENLDQAFSVAERDLGVTRLLDPEDVDVPQPDEKSIITYVSSLYDAMPRVPDVQDGVKANELQLRWQEYRELVLLLLQ
4z6g.1.A   SNRENLEQAFEVAER-LGVTRLLDAEDVDVPSPDEKSVITYVSSIYDAFPKVPEGGEGISATEVDSRWQEYQSRVDSLIP

Target     WIRAHTAAFEERRFPSSFEEIEILWCQFLKFKETELPAKEADKNRSKGIYQSLEGAVQAGQLKVPPGYHPLDVEKEWGKL
4z6g.1.A   WIKQHTILMSDKTFPQNPVELKALYNQYIHFKETEILAKEREKGRIEELYKLLEVWIEFGRIKLPQGYHPNDVEEEWGKL

Target     HVAILEREKQLRSEFERLERLQRIVSKLQMEAGLCEEQLNQADALLQSDVRLLAAGKAPQRAGEVERDLDKADGMIRLLF
4z6g.1.A   IIEMLEREKSLR--------------------------------------------------------------------

Target     NDVQALKDGRHPQGEQMYRRVYRLHERLVAIRTEYNLRLRGTPRHPELEDSTLRYLQDLLAWVEENQRRVDSAEWGVDLP
4z6g.1.A   --------------------------------------------------------------------------------

Target     SVEAQLGSHRGLHQSVEEFRAKIERARTDEGQLSPATRGAYRDCLGRLDLQYAKLLNSSKARLRSLESLHGFVAAATKEL
4z6g.1.A   --------------------------------------------------------------------------------

Target     MWLSEKEEEEVGFDWSERNSNMAAKKEAYSALMRELELKEKKIKEIQSTGDRLLREDHPARPTVESFQAALQTQWSWMLQ
4z6g.1.A   --------------------------------------------------------------------------------

Target     LCCCIEAHLKENTAYFQFFSDVREAEEQLRKLQETLHRKYTCDRSITVTRLEDLLQDAQDEKDQLNEYRGHLSGLAKRAK
4z6g.1.A   --------------------------------------------------------------------------------

Target     AIVQLTPRNPTQPTRGRVPLLAVCDYKQVEATVHKGDECQMLGPAQPFHWKVLSSSGSEAAVPSVCFLVPPPNQEALEAV
4z6g.1.A   --------------------------------------------------------------------------------

Target     ARLEAQHQALVTLWHQLHTDMKSLLAWQSLSRDVQLIRSWSLVTFRTLKPEEQRQALRSLELHYQAFLRDSQDAGGFGPE
4z6g.1.A   --------------------------------------------------------------------------------

Target     DRLQAEREYGSCSRHYQQLLQSLEQGEQEESRCQRCISELKDIRLQLEACETRTVHRLRLPLDKEPARECAQRIAEQQKA
4z6g.1.A   --------------------------------------------------------------------------------
```

```
Target     QAEVEGLGKGVARLSAEAEKVLALPEPSPAAPTLRSELELTLGKLEQVRSLSAIYLEKLKTISLVIRSTQGAEEALRAHE
4z6g.1.A   --------------------------------------------------------------------------------

Target     EQLKEAQAVPAALPELEATKAAMKKLRAQAEAQQPVFDALRDELRGAQEVGERLQQRHGERDVEVERWRERVTQLLERWQ
4z6g.1.A   --------------------------------------------------------------------------------

Target     AVLAQTDVRQRELEQLGRQLRYYRESADPLGAWLQDARRRQEQIQAVPLADSQAVREQLRQEKALLEEIERHAEKVEECQ
4z6g.1.A   --------------------------------------------------------------------------------

Target     RFAKQYINAIKDYELQLVTYKAQLEPVASPAKKPKVQSGSESVIQEYVDLRTRYSELSTLTSQYIRFISETLRRMEEEER
4z6g.1.A   --------------------------------------------------------------------------------

Target     LAEQQRAEERERLAEVEAALEKQRQLAEAHAQAKAQAEREAQELQRRMQEEVARREEVAVDAQQQKRSIQEELQQLRQSS
4z6g.1.A   --------------------------------------------------------------------------------

Target     EAEIQAKARQVEAAERSRLRIEEEIRVVRLQLETTERQRGGAEGELQALRARAEEAEAQKRQAQEEAERLRRQVQEETQR
4z6g.1.A   --------------------------------------------------------------------------------

Target     KRQAEAELGLRVKAEAEAAREKQRALQALEELRLQAEEAERRLRQAEAERARQVQVALETAQRSAQAELQSKHASFAEKT
4z6g.1.A   --------------------------------------------------------------------------------

Target     AQLERTLEEEHVTVVQLREEATRREQQQAEAERAREEAERELERWQLKANEALRLRLQAEEVAQQKSLAQAEAEKQKEAA
4z6g.1.A   --------------------------------------------------------------------------------

Target     EREARRRGKAEEQAVRQRELAEQELERQRQLAEGTAQQRLAAEQELIRLRAETEQGEQQRQLLEEELARLQSEAAAATQK
4z6g.1.A   --------------------------------------------------------------------------------

Target     RQELEAELAKVRAEMEVLLASKARAEEESRSSSEKSKQRLEAEAGRFRELAEEAARLRALAEEAKRQRQLAEEDAARQRA
4z6g.1.A   --------------------------------------------------------------------------------

Target     EAERVLSEKLAAISEATRLKTEAEIALKEKEAENERLRRLAEDEAFQRRRLEEQAAQHKADIEERLAQLRKASESELERQ
4z6g.1.A   --------------------------------------------------------------------------------

Target     KGLVEDTLRQRRQVEEEILALKASFEKAAAGKAELELELGRIRGNAEDTLRSKEQAEQEAARQRQLAAEEERRRREAEER
4z6g.1.A   --------------------------------------------------------------------------------

Target     VQKSLAAEEEAARQRKAALEEVERLKAKVEEARRLRERAEHESVRQLQLAQEAAQKRLQAEEKAHAFAVQQKEQELQQTL
4z6g.1.A   --------------------------------------------------------------------------------

Target     QQEQSVLEKLRSEAEAARRAAEEAEEARERAEREAAQSRQRVEEAERLKQAAEEQAQAQAQAQAAAEKLRKEAEQEAARR
4z6g.1.A   --------------------------------------------------------------------------------

Target     AQAEQAALRQKQAADAEMEKHKKFAEQTLRQKAQVEQELTALRLKLEETDHQKSILDQELQRLKAEVTEAARQRSQVEEE
4z6g.1.A   --------------------------------------------------------------------------------

Target     LFSVRVQMEELGKLKARIEAENRALILRDKDNTQRLLQEEAEKMKQVAEEAARLSVAAQEAARLRQLAEEDLAQQRALAE
4z6g.1.A   --------------------------------------------------------------------------------

Target     KMLKEKMQAVQEATRLKAEAELLQQQKELAQEQARRLQEDKEQMAQQLAQETQGFQRTLETERQRQLEMSAEAERLRLRV
4z6g.1.A   --------------------------------------------------------------------------------

Target     AEMSRAQARAEEDAQRFRKQAEEIGAKLHRTELATQEKVTLVQTLETQRQQSDRDADRLREAIAELEREKDKLKKEAELL
4z6g.1.A   --------------------------------------------------------------------------------

Target     QLKSEEMQTVQQEQLLQETQALQQSFLSEKDSLLQRERFIEEEKAKLERLFQDEVAKAQKLREEQQRQQQMQQEKQQLL
4z6g.1.A   --------------------------------------------------------------------------------

Target     ASMEEARRRQHEAEEGVRRKQEELQLLEQQRQQQEQLLAEENRRLRERLEHLEEEHRAALAHSEEITAAQAAATRALPNG
4z6g.1.A   --------------------------------------------------------------------------------

Target     QDAADGPAAEPEHAFEGLRQKVPAQQLQEAGILSTEELQRLAQGHTTVAELTQREDVRRYLQGHSSIAGLLLKPANEKLT
4z6g.1.A   --------------------------------------------------------------------------------

Target     IYAALRRQLLSPGTALILLEAQAASGFLLDPVRNRRLTVTEAVKEGVVGPELHHKLLSAERAVTGYKDPYTGEKISLFQA
4z6g.1.A   --------------------------------------------------------------------------------

Target     MKKDLIVREHGIRLLEAQIATGGVIDPVHSHRVPVDVAYHRGYFDEEMNRVLQDPSDDTKGFFDPNTHENLTYLQLLERC
4z6g.1.A   --------------------------------------------------------------------------------

Target     VEDPETGLRLLPLTDQAAKGGELVYTDSEARDVFEKATVSAPFGKFQGKTVTIWELINSEYFTAEQRRDLLRQFRTGKVT
4z6g.1.A   --------------------------------------------------------------------------------

Target     VEKIIKIVITVIEEHEQKGQLCFQGLRALVPAAELLESGIIDWDLFRQLQLGERSVQEVAEVEGVRRALRGSGVIAGVWL
```

```
4z6g.1.A   --------------------------------------------------------------------------------

Target     EEARQKLSIYEALKKELLQPEAAVALLEAQAGTGHVIDPATSARLTVDEAVRAGLVGPELHEKLLSAEKAVTGYKDPYSG
4z6g.1.A   --------------------------------------------------------------------------------

Target     QSVSLFQALKKGLIPREQGLRLLDAQLSTGGTVDPSKSHRLPLDVACARGYLDEETSTALSAPRDDAKTYYDPRTWELAT
4z6g.1.A   --------------------------------------------------------------------------------

Target     YSQLQQQCRPDPLTGLSLLPLSEEAARARQQELYSEVQAREAFQKATVEVPVGSFQGRAVTIWELINSEYFTAEQRQELL
4z6g.1.A   --------------------------------------------------------------------------------

Target     RQFRTGKVTVEKIIKIVITIVEEVETTRRERLSFSGLRAPVPASELLAAGILSSSQFEQLKDGKTSVKDLSELSSLQTLL
4z6g.1.A   --------------------------------------------------------------------------------

Target     QGSGCLAGIYLEESKEKVTIYEAMRRGLLRPSTAILLLEAQAATGFLVDPVRNQRLYVHEAVKAGVVGPELHEKLLSAEK
4z6g.1.A   --------------------------------------------------------------------------------

Target     AVTGYKDPYSGSTISLFQAMKKGLVVREHGIRLLEAQIATGGIIDPVHSHRVPVDVACQRGYFDEEMNRVLEDPSDDTKG
4z6g.1.A   --------------------------------------------------------------------------------

Target     FFDPNTHENLTYRQLLERCVEDPETGLRLLPLKGPEKAEVVETTRVYTEEETRRAFEETQIDIPGGGSHGGSTMSLWEVM
4z6g.1.A   --------------------------------------------------------------------------------

Target     QSDLIPEEQRAQLMADFQAGRVTKERMIIIIIEIIEKTEIVRQQNLASYDYVRRRLTAEDLYEARVISRESYSLLREGTR
4z6g.1.A   --------------------------------------------------------------------------------

Target     SLREVLEAESAWRYLYGTGCVAGVYLPGSRQTLPIYQALKKGLLSAEVARLLLEAQAATGFLLDPVKGERLTVDEAVRKG
4z6g.1.A   --------------------------------------------------------------------------------

Target     LVGPELHDRLLSAERAVTGYRDPYTEQTISLFQAMKKDLIPAEEALRLLDAQLATGGIVDPRLGFHLPLEVAYQRGYLNK
4z6g.1.A   --------------------------------------------------------------------------------

Target     DTHDQLSEPSEVRSYVDPSTDERLSYTQLLRRCRRDETSGLFLLPLSEARKLTFRGLRKQITVEELVRSHVMDEATAQRL
4z6g.1.A   --------------------------------------------------------------------------------

Target     QEGLTSIEEVSKNLQKFLEGTSSIAGVLVDATKERLSVYQAMKKGIIRPGTAFELLEAQAATGYVIDPIKGLKLTVEEAV
4z6g.1.A   --------------------------------------------------------------------------------

Target     RMGIVGPEFKDKLLSAERAVTGYKDPYSGKLISLFQAMKKGLILKDHGIRLLEAQIATGGIIDPEESHRLPVDVAYQRGL
4z6g.1.A   --------------------------------------------------------------------------------

Target     FDEEMNEILTDPSDDTKGFFDPNTEENLTYLQLMERCVTDPQTGLRLLPLKEKKRERKTSSKSSVRKRRVVIVDPETGKE
4z6g.1.A   --------------------------------------------------------------------------------

Target     MSVYEAYRKGLIDHQTYLELSEQECEWEEITISSSDGVVKSMIIDRRSGRQYDIDEAIAKSLIDRSALDQYRAGTLSITE
4z6g.1.A   --------------------------------------------------------------------------------

Target     FADMLSGNAGGFRSRSSSVGSSSSYPISPAVSRTQLASWSDPTEETGPVAGILDTETLEKVSITEAMHRNLVDNITGQRL
4z6g.1.A   --------------------------------------------------------------------------------

Target     LEAQACTGGIIDPNTGERFPVTDAVNKGLVDKIMVDRINLAQKAFCGFEDPRTKTKMSAAQALKKGWLYYEAGQRFLEVQ
4z6g.1.A   --------------------------------------------------------------------------------

Target     YLTGGLIEPDTPGRVPLDEALQRGTVDARTAQKLRDVSAYSKYLTCPKTKLKISYKDALDRSMVEEGTGLRLLEAAAQSS
4z6g.1.A   --------------------------------------------------------------------------------

Target     KGYYSPYSVSGSGSTTGSRSGSRTGSRAGSRRGSFDATGSGFSMTFSSSSYSSSGYGRRYASGPTSSLGGPESAAA
4z6g.1.A   ----------------------------------------------------------------------------
```

Model #05	File	Built with	Oligo-State	Ligands	GMQE	QMEANDisCo Global
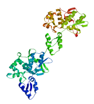	PDB	ProMod3 3.3.0	monomer	None	0.00	0.73 ± 0.05

Template	Seq Identity	Oligo-state	QSQE	Found by	Method	Resolution	Seq Similarity	Range	Coverage	Description
5dzz.1.A	51.14	monomer	0.00	HHblits	X-ray	2.60Å	0.45	3645 - 4131	0.11	Desmoplakin

The template contained no ligands.

```
Target     MSGEDQEVRAVVEDGSNGGSGSPSPGDTLPWNLEKTQRSRRGGGGPGGNGSVLDPAERAVIRIADERDRVQKKTFTKWVN
5dzz.1.A   --------------------------------------------------------------------------------

Target     KHLIKAQRHISDLYEDLRDGHNLISLLEVLSGDSLPREKGRMRFHKLQNVQIALDYLRHRQVKLVNIRNDDIADGNPKLT
5dzz.1.A   --------------------------------------------------------------------------------

Target     LGLIWTIILHFQISDIQVSGQSEDMTAKEKLLLWSQRMVEGYQGLRCDNFTSSWRDGRLFNAIIHRHKPMLIDMNKVYRQ
5dzz.1.A   --------------------------------------------------------------------------------

Target     TNLENLDQAFSVAERDLGVTRLLDPEDVDVPQPDEKSIITYVSSLYDAMPRVPDVQDGVKANELQLRWQEYRELVLLLLQ
5dzz.1.A   --------------------------------------------------------------------------------

Target     WIRAHTAAFEERRFPSSFEEIEILWCQFLKFKETELPAKEADKNRSKGIYQSLEGAVQAGQLKVPPGYHPLDVEKEWGKL
5dzz.1.A   --------------------------------------------------------------------------------

Target     HVAILEREKQLRSEFERLERLQRIVSKLQMEAGLCEEQLNQADALLQSDVRLLAAGKAPQRAGEVERDLDKADGMIRLLF
5dzz.1.A   --------------------------------------------------------------------------------

Target     NDVQALKDGRHPQGEQMYRRVYRLHERLVAIRTEYNLRLRGTPRHPELEDSTLRYLQDLLAWVEENQRRVDSAEWGVDLP
5dzz.1.A   --------------------------------------------------------------------------------

Target     SVEAQLGSHRGLHQSVEEFRAKIERARTDEGQLSPATRGAYRDCLGRLDLQYAKLLNSSKARLRSLESLHGFVAAATKEL
5dzz.1.A   --------------------------------------------------------------------------------

Target     MWLSEKEEEEVGFDWSERNSNMAAKKEAYSALMRELELKEKKIKEIQSTGDRLLREDHPARPTVESFQAALQTQWSWMLQ
5dzz.1.A   --------------------------------------------------------------------------------

Target     LCCCIEAHLKENTAYFQFFSDVREAEEQLRKLQETLHRKYTCDRSITVTRLEDLLQDAQDEKDQLNEYRGHLSGLAKRAK
5dzz.1.A   --------------------------------------------------------------------------------

Target     AIVQLTPRNPTQPTRGRVPLLAVCDYKQVEATVHKGDECQMLGPAQPFHWKVLSSSGSEAAVPSVCFLVPPPNQEALEAV
5dzz.1.A   --------------------------------------------------------------------------------

Target     ARLEAQHQALVTLWHQLHTDMKSLLAWQSLSRDVQLIRSWSLVTFRTLKPEEQRQALRSLELHYQAFLRDSQDAGGFGPE
5dzz.1.A   --------------------------------------------------------------------------------

Target     DRLQAEREYGSCSRHYQQLLQSLEQGEQEESRCQRCISELKDIRLQLEACETRTVHRLRLPLDKEPARECAQRIAEQQKA
5dzz.1.A   --------------------------------------------------------------------------------

Target     QAEVEGLGKGVARLSAEAEKVLALPEPSPAAPTLRSELELTLGKLEQVRSLSAIYLEKLKTISLVIRSTQGAEEALRAHE
5dzz.1.A   --------------------------------------------------------------------------------

Target     EQLKEAQAVPAALPELEATKAAMKKLRAQAEAQQPVFDALRDELRGAQEVGERLQQRHGERDVEVERWRERVTQLLERWQ
5dzz.1.A   --------------------------------------------------------------------------------

Target     AVLAQTDVRQRELEQLGRQLRYYRESADPLGAWLQDARRRQEQIQAVPLADSQAVREQLRQEKALLEEIERHAEKVEECQ
5dzz.1.A   --------------------------------------------------------------------------------

Target     RFAKQYINAIKDYELQLVTYKAQLEPVASPAKKPKVQSGSESVIQEYVDLRTRYSELSTLTSQYIRFISETLRRMEEEER
5dzz.1.A   --------------------------------------------------------------------------------

Target     LAEQQRAEERERLAEVEAALEKQRQLAEAHAQAKAQAEREAQELQRRMQEEVARREEVAVDAQQQKRSIQEELQQLRQSS
5dzz.1.A   --------------------------------------------------------------------------------

Target     EAEIQAKARQVEAAERSRLRIEEEIRVVRLQLETTERQRGGAEGELQALRARAEEAEAQKRQAQEEAERLRRQVQEETQR
5dzz.1.A   --------------------------------------------------------------------------------

Target     KRQAEAELGLRVKAEAEAAREKQRALQALEELRLQAEEAERRLRQAEAERARQVQVALETAQRSAQAELQSKHASFAEKT
5dzz.1.A   --------------------------------------------------------------------------------

Target     AQLERTLEEEHVTVVQLREEATRREQQQAEAERAREEAERELERWQLKANEALRLRLQAEEVAQQKSLAQAEAEKQKEAA
5dzz.1.A   --------------------------------------------------------------------------------

Target     EREARRRGKAEEQAVRQRELAEQELERQRQLAEGTAQQRLAAEQELIRLRAETEQGEQQRQLLEEELARLQSEAAAATQK
5dzz.1.A   --------------------------------------------------------------------------------
```

```
Target    RQELEAELAKVRAEMEVLLASKARAEEESRSSSEKSKQRLEAEAGRFRELAEEAARLRALAEEAKRQRQLAEEDAARQRA
5dzz.1.A  --------------------------------------------------------------------------------

Target    EAERVLSEKLAAISEATRLKTEAEIALKEKEAENERLRRLAEDEAFQRRRLEEQAAQHKADIEERLAQLRKASESELERQ
5dzz.1.A  --------------------------------------------------------------------------------

Target    KGLVEDTLRQRRQVEEEILALKASFEKAAAGKAELELELGRIRGNAEDTLRSKEQAEQEAARQRQLAAEEERRRREAEER
5dzz.1.A  --------------------------------------------------------------------------------

Target    VQKSLAAEEEAARQRKAALEEVERLKAKVEEARRLRERAEHESVRQLQLAQEAAQKRLQAEEKAHAFAVQQKEQELQQTL
5dzz.1.A  --------------------------------------------------------------------------------

Target    QQEQSVLEKLRSEAEAARRAAEEAEEARERAEREAAQSRQRVEEAERLKQAAEEQAQAQAQAQAAAEKLRKEAEQEAARR
5dzz.1.A  --------------------------------------------------------------------------------

Target    AQAEQAALRQKQAADAEMEKHKKFAEQTLRQKAQVEQELTALRLKLEETDHQKSILDQELQRLKAEVTEAARQRSQVEEE
5dzz.1.A  --------------------------------------------------------------------------------

Target    LFSVRVQMEELGKLKARIEAENRALILRDKDNTQRLLQEEAEKMKQVAEEAARLSVAAQEAARLRQLAEEDLAQQRALAE
5dzz.1.A  --------------------------------------------------------------------------------

Target    KMLKEKMQAVQEATRLKAEAELLQQQKELAQEQARRLQEDKEQMAQQLAQETQGFQRTLETERQRQLEMSAEAERLRLRV
5dzz.1.A  --------------------------------------------------------------------------------

Target    AEMSRAQARAEEDAQRFRKQAEEIGAKLHRTELATQEKVTLVQTLETQRQQSDRDADRLREAIAELEREKDKLKKEAELL
5dzz.1.A  --------------------------------------------------------------------------------

Target    QLKSEEMQTVQQEQLLQETQALQQSFLSEKDSLLQRERFIEEEKAKLERLFQDEVAKAQKLREEQQRQQQMQQEKQQLL
5dzz.1.A  --------------------------------------------------------------------------------

Target    ASMEEARRRQHEAEEGVRRKQEELQLLEQQRQQQEQLLAEENRRLRERLEHLEEEHRAALAHSEEITAAQAAATRALPNG
5dzz.1.A  --------------------------------------------------------------------------------

Target    QDAADGPAAEPEHAFEGLRQKVPAQQLQEAGILSTEELQRLAQGHTTVAELTQREDVRRYLQGHSSIAGLLLKPANEKLT
5dzz.1.A  --------------------------------------------------------------------------------

Target    IYAALRRQLLSPGTALILLEAQAASGFLLDPVRNRRLTVTEAVKEGVVGPELHHKLLSAERAVTGYKDPYTGEKISLFQA
5dzz.1.A  --------------------------------------------------------------------------------

Target    MKKDLIVREHGIRLLEAQIATGGVIDPVHSHRVPVDVAYHRGYFDEEMNRVLQDPSDDTKGFFDPNTHENLTYLQLLERC
5dzz.1.A  --------------------------------------------------------------------------------

Target    VEDPETGLRLLPLTDQAAKGGELVYTDSEARDVFEKATVSAPFGKFQGKTVTIWELINSEYFTAEQRRDLLRQFRTGKVT
5dzz.1.A  --------------------------------------------------------------------------------

Target    VEKIIKIVITVIEEHEQKGQLCFQGLRALVPAAELLESGIIDWDLFRQLQLGERSVQEVAEVEGVRRALRGSGVIAGVWL
5dzz.1.A  --------------------------------------------------------------------------------

Target    EEARQKLSIYEALKKELLQPEAAVALLEAQAGTGHVIDPATSARLTVDEAVRAGLVGPELHEKLLSAEKAVTGYKDPYSG
5dzz.1.A  --------------------------------------------------------------------------------

Target    QSVSLFQALKKGLIPREQGLRLLDAQLSTGGTVDPSKSHRLPLDVACARGYLDEETSTALSAPRDDAKTYYDPRTWELAT
5dzz.1.A  --------------------------------------------------------------------------------

Target    YSQLQQQCRPDPLTGLSLLPLSEEAARARQQELYSEVQAREAFQKATVEVPVGSFQGRAVTIWELINSEYFTAEQRQELL
5dzz.1.A  --------------------------------------------------------------------------------

Target    RQFRTGKVTVEKIIKIVITIVEEVETTRRERLSFSGLRAPVPASELLAAGILSSSQFEQLKDGKTSVKDLSELSSLQTLL
5dzz.1.A  --------------------------------------------------------------------------------

Target    QGSGCLAGIYLEESKEKVTIYEAMRRGLLRPSTAILLLEAQAATGFLVDPVRNQRLYVHEAVKAGVVGPELHEKLLSAEK
5dzz.1.A  --------------------------------------------------------------------------------

Target    AVTGYKDPYSGSTISLFQAMKKGLVVREHGIRLLEAQIATGGIIDPVHSHRVPVDVACQRGYFDEEMNRVLEDPSDDTKG
5dzz.1.A  --------------------------------------------------------------------------------

Target    FFDPNTHENLTYRQLLERCVEDPETGLRLLPLKGPEKAEVVETTRVYTEEETRRAFEETQIDIPGGGSHGGSTMSLWEVM
5dzz.1.A  --------------------------------------------------------------------------------

Target    QSDLIPEEQRAQLMADFQAGRVTKERMIIIIIEIIEKTEIVRQQNLASYDYVRRRLTAEDLYEARVISRESYSLLREGTR
5dzz.1.A  --------------------------------------------SKLVFDGLRKKVTAMQLYECQLIDKTTLDKLLKGKK

Target    SLREVLEAESAWRYLYGTGCVAGVYLPGSRQTLPIYQALKKGLLSAEVARLLLEAQAATGFLLDPVKGERLTVDEAVRKG
5dzz.1.A  SVEEVAS--EIQPFLRGAGSIAGAS-ASPKEKYSLVEAKRKKLISPESTVMLLEAQAATGGIIDPHRNEKLTVDSAIARD
```

```
Target     LVGPELHDRLLSAERAVTGYRDPYTEQTISLFQAMKKDLIPAEEALRLLDAQLATGGIVDPRLGFHLPLEVAYQRGYLNK
5dzz.1.A   LIDFDDRQQIYAAEKAITGFDDPFSGKTVSVSEAIKKNLIDRETGMRLLEAQIASGGVVDPVNSVFLPKDVALARGLIDR

Target     DTHDQLSEPSE-VRSYVDPSTDERLSYTQLLRRCRRDETSGLFLLPLSEARKLTFRGLRKQITVEELVRSHVMDEATAQR
5dzz.1.A   DLYRSLNDPRDSQKNFVDPVTKKKVSYVQLKERCRIEPHTGLLLLSVQKR-SMSFQGIRQPVTVTELVDSGILRPSTVNE

Target     LQEGLTSIEEVSKNLQKFLEGTSSIAGVLVDATKERLSVYQAMKKGIIRPGTAFELLEAQAATGYVIDPIKGLKLTVEEA
5dzz.1.A   LESGQISYDEVGERIKDFLQGSSCIAGIYNETTKQKLGIYEAMKIGLVRPGTALELLEAQAATGFIVDPVSNLRLPVEEA

Target     VRMGIVGPEFKDKLLSAERAVTGYKDPYSGKLISLFQAMKKGLILKDHGIRLLEAQIATGGIIDPEESHRLPVDVAYQRG
5dzz.1.A   YKRGLVGIEFKEKLLSAERAVTGYNDPETGNIISLFQAMNKELIEKGHGIRLLEAQIATGGIIDPKESHRLPVDIAYKRG

Target     LFDEEMNEILTDPSDDTKGFFDPNTEENLTYLQLMERCVTDPQTGLRLLPLKEKKRERKTSSKSSVRKRRVVIVDPETGK
5dzz.1.A   YFNEELSEILSDPSDDTKGFFDPNTEENLTYLQLKERCIKDEETGLCLLPLK----------------------------

Target     EMSVYEAYRKGLIDHQTYLELSEQECEWEEITISSSDGVVKSMIIDRRSGRQYDIDEAIAKSLIDRSALDQYRAGTLSIT
5dzz.1.A   --------------------------------------------------------------------------------

Target     EFADMLSGNAGGFRSRSSSVGSSSSYPISPAVSRTQLASWSDPTEETGPVAGILDTETLEKVSITEAMHRNLVDNITGQR
5dzz.1.A   --------------------------------------------------------------------------------

Target     LLEAQACTGGIIDPNTGERFPVTDAVNKGLVDKIMVDRINLAQKAFCGFEDPRTKTKMSAAQALKKGWLYYEAGQRFLEV
5dzz.1.A   --------------------------------------------------------------------------------

Target     QYLTGGLIEPDTPGRVPLDEALQRGTVDARTAQKLRDVSAYSKYLTCPKTKLKISYKDALDRSMVEEGTGLRLLEAAAQS
5dzz.1.A   --------------------------------------------------------------------------------

Target     SKGYYSPYSVSGSGSTTGSRSGSRTGSRAGSRRGSFDATGSGFSMTFSSSSYSSSGYGRRYASGPTSSLGGPESAAA
5dzz.1.A   -----------------------------------------------------------------------------
```

Model #20	File	Built with	Oligo-State	Ligands	GMQE	QMEANDisCo Global
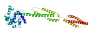	PDB	ProMod3 3.3.0	monomer	None	0.00	0.50 ± 0.05

Template	Seq Identity	Oligo-state	QSQE	Found by	Method	Resolution	Seq Similarity	Range	Coverage	Description
8iah.1.2	15.20	monomer	0.00	HHblits	EM	-	0.28	186 - 630	0.22	Spectrin beta chain

Excluded ligands

Ligand Name.Number	Reason for Exclusion	Description
ADP.1	Binding site not conserved.	ADENOSINE-5'-DIPHOSPHATE
ADP.2	Binding site not conserved.	ADENOSINE-5'-DIPHOSPHATE
ADP.3	Binding site not conserved.	ADENOSINE-5'-DIPHOSPHATE
ADP.4	Binding site not conserved.	ADENOSINE-5'-DIPHOSPHATE
ADP.5	Binding site not conserved.	ADENOSINE-5'-DIPHOSPHATE
ADP.6	Binding site not conserved.	ADENOSINE-5'-DIPHOSPHATE
ADP.7	Binding site not conserved.	ADENOSINE-5'-DIPHOSPHATE
ADP.8	Binding site not conserved.	ADENOSINE-5'-DIPHOSPHATE
ADP.9	Binding site not conserved.	ADENOSINE-5'-DIPHOSPHATE
ADP.10	Binding site not conserved.	ADENOSINE-5'-DIPHOSPHATE
ADP.11	Binding site not conserved.	ADENOSINE-5'-DIPHOSPHATE
ADP.12	Binding site not conserved.	ADENOSINE-5'-DIPHOSPHATE

```
Target     MSGEDQEVRAVVEDGSNGGSGSPSPGDTLPWNLEKTQRSRRGGGGPGGNGSVLDPAERAVIRIADERDRVQKKTFTKWVN
8iah.1.2   --------------------------------------------------------------------------------

Target     KHLIKAQRHISDLYEDLRDGHNLISLLEVLSGDSLPREKGRMRFHKLQNVQIALDYLRHRQVKLVNIRNDDIADGNPKLT
8iah.1.2   --------------------------------------------------------------------------------
```

```
Target     LGLIWTIILHFQISDIQVSGQSEDMTAKEKLLLWSQRMVEGYQGLRCDNFTSSWRDGRLFNAIIHRHKPMLIDMNK--VY
8iah.1.2   -------------------------VQKKTFTKWVNSHLARV-SCRITDLYKDLRDGRMLIKLLEVLSGEMLPKPTKGKM

Target     RQTNLENLDQAFSVAERDLGVTRL-LDPEDVDVPQPDEKSIITYVSSLYDAMPRVPDVQDGVKANELQLRWQEYRELVLL
8iah.1.2   RIHCLENVDKALQFLK-EQRVHLENMGSHDIVDGNH--RLVLGLIWTIILRFQIQDIVVQTQEG-------RETRSAKDA

Target     LLQWIRAHTAAF---EERRFPSSFEEIEILWCQFLKFKETELPAK-EAD---KNRSKGIYQSLEGAVQAGQLKVPPGYHP
8iah.1.2   LLLWCQMKTAGYPNVNVTNFTSSWKDGLAFNALIHKHRPDLIDFDKLKDSNARHNLEHAFDVAERQ-----LGIIQLLDP

Target     LDVE--------------KEWGKLHVAIL--EREKQLRSEFERLERLQRIVSKLQMEAGLCEEQLNQADALLQSDVRL--
8iah.1.2   EDVFTENPDEKSIITYVVAFYHYFSKMKVLAVEGKRVGKVIDHAIETEKMIEKYSGLASDLLTWIEQTITVLNSRKFANS

Target     -LAAGKAPQRAGEVERDLDK--A--DGMIRLLFNDVQA-LKDGR----HPQGEQMYRRVYRLHERLVAIRTEYNLRLRGT
8iah.1.2   LAGVQQQLQAFSTYRTVEKPPKFQEKGNLEVLLFTIQSRMRANNQKVYTPHDGKLVSDINRAWESLEEAEYRRELALRSE

Target     PRH----PELEDSTLRYLQDLLAWVEENQRRVDSAEWGVDLPSVEAQLGSHRGLHQSVEEFRAKIERARTDEGQLS---P
8iah.1.2   LIRQEKLEQLARRFDRKAAMRETWLNENQRLVAQDNFGYDLAAVEAAKKKHEAIETDTAAYEERVRALEDLARELELENY

Target     ATRGAYRDCLGRLDLQYAKLLNSSKARLRSLE---SLHGFVAAATKELMWLSEKEEEEVGFDWSERNSNMAAKKEAYSAL
8iah.1.2   HDQKRITARKDNILRLWNYLQELLQSRRQRLETTLALQQLFQDMLHSIDWMDEIKAHLLSAEFGKHLLEAEDLLQKHKLM

Target     MRELELKEKKIKEIQSTGDRLLREDH---PARPTVESFQAALQTQWSWMLQLCCCIEAHLKENTAYFQFFSDVREAEEQL
8iah.1.2   EADIAIQGDKVKAITAATLQFTEETGYQPCDPQVIRDRVSHLEQCFAELSNTAAGRKAQLEQSKRLWKLFWEMDEAKSWI

Target     RKLQETLHRKYTCDRSITVTRLEDLLQDAQDEKDQLNEYRGHLSGLAKRAKAIVQLTPRNPTQPTRGRVPLLAVCDYKQV
8iah.1.2   KEKEQIYS---SLGYGKDLTSVLILQRKHKAFEDELRRLDPHLDQIFQEAEDMVALKQFG--YP-----K---NEAW---

Target     EATVHKGDECQMLGPAQPFHWKVLSSSGSEAAVPSVCFLVPPPNQEALEAVARLEAQHQALVTLWHQLHTDMKSLLAWQS
8iah.1.2   --------------------------------V--------KEVSAQWDQLKEVPAQWNQLKELAASRKKNLQDTENFFQ

Target     LSRDVQLIRSWSLVTFRTLKPEEQ---RQALRSLELHYQAFLRDSQDAGGF-------------GPEDRLQAEREYGSCS
8iah.1.2   FQGDVDDLKAWLQDAHKLLSGEDVGQDEGATRALGKKHKDFLEELEESRGVMEHLEQQAQDFPERFRDSPDVTNRLQVLR

Target     RHYQQLLQSLEQGEQE---ESRCQRCISELKDIRLQLEACETRTVHRLRLPLDKEPARECAQRIAEQQKAQAEVEGLGKG
8iah.1.2   DLYQQVVAQADLRRQRLQDALDLYTVFGETDACELWMGEKEKWLAQ-MDIPD---TLEDLEVVQHRFDILDQEMKTLIAQ

Target     VARLSAEAEKVLALPEPSPAAPTLRSELELTLGKLEQVRSLSAIYLEKLKTISL---VIRSTQGAEEALRAHEEQLKEAQ
8iah.1.2   IDGVNVAANSLVESN--HPRSTEVKQYQDHLNTRWREFQTMVLARREAVDSALRVHNYCVDCEETSKWIIDKTKVVESTK

Target     AVPAALPELEATKAAMKKLRAQAEAQQPVFDALRDELRGAQEVGERLQQRHGERDVEVERWRERVTQLLERWQAVLAQTD
8iah.1.2   DLGQDLAGVMAIQRKLSGLERDVAAIQVRVGALEQ-------ESHRLMES---HREQEKDIGERQAYVEELWQGLQQALK

Target     VRQRELEQLGRQLRYYRESADPLGAWLQDARRRQEQIQAVPLADSQAVREQLRQEKALLEEIERHAEKVEECQRFAKQYI
8iah.1.2   GQEALLGKS-SQLQAFLQDLDAFEAWLSTAQKEVASK--DMPESLPEAEQLLQQHAALKDDIDRHQENYQHVKASGEKVI

Target     NAIKDYELQLVTYKAQLEPVASPAKKPKVQSGSESVIQEYVDLRTRYSELSTLTSQYIRFISETLRRMEEEERLAEQQRA
8iah.1.2   HG------------------------------------------------------------------------------

Target     EERERLAEVEAALEKQRQLAEAHAQAKAQAEREAQELQRRMQEEVARREEVAVDAQQQKRSIQEELQQLRQSSEAEIQAK
8iah.1.2   --------------------------------------------------------------------------------

Target     ARQVEAAERSRLRIEEEIRVVRLQLETTERQRGGAEGELQALRARAEEAEAQKRQAQEEAERLRRQVQEETQRKRQAEAE
8iah.1.2   --------------------------------------------------------------------------------

Target     LGLRVKAEAEAAREKQRALQALEELRLQAEEAERRLRQAEAERARQVQVALETAQRSAQAELQSKHASFAEKTAQLERTL
8iah.1.2   --------------------------------------------------------------------------------

Target     EEEHVTVVQLREEATRREQQQAEAERAREEAERELERWQLKANEALRLRLQAEEVAQQKSLAQAEAEKQKEAAEREARRR
8iah.1.2   --------------------------------------------------------------------------------

Target     GKAEEQAVRQRELAEQELERQRQLAEGTAQQRLAAEQELIRLRAETEQGEQQRQLLEEELARLQSEAAAATQKRQELEAE
8iah.1.2   --------------------------------------------------------------------------------

Target     LAKVRAEMEVLLASKARAEEESRSSSEKSKQRLEAEAGRFRELAEEAARLRALAEEAKRQRQLAEEDAARQRAEAERVLS
8iah.1.2   --------------------------------------------------------------------------------

Target     EKLAAISEATRLKTEAEIALKEKEAENERLRRLAEDEAFQRRRLEEQAAQHKADIEERLAQLRKASESELERQKGLVEDT
8iah.1.2   --------------------------------------------------------------------------------

Target     LRQRRQVEEEILALKASFEKAAAGKAELELELGRIRGNAEDTLRSKEQAEQEAARQRQLAAEEERRRREAEERVQKSLAA
8iah.1.2   --------------------------------------------------------------------------------

Target     EEEAARQRKAALEEVERLKAKVEEARRLRERAEHESVRQLQLAQEAAQKRLQAEEKAHAFAVQQKEQELQQTLQQEQSVL
```

```
8iah.1.2    --------------------------------------------------------------------------------

Target      EKLRSEAEAARRAAEEAEEARERAEREAAQSRQRVEEAERLKQAAEEQAQAQAQAQAAAEKLRKEAEQEAARRAQAEQAA
8iah.1.2    --------------------------------------------------------------------------------

Target      LRQKQAADAEMEKHKKFAEQTLRQKAQVEQELTALRLKLEETDHQKSILDQELQRLKAEVTEAARQRSQVEEELFSVRVQ
8iah.1.2    --------------------------------------------------------------------------------

Target      MEELGKLKARIEAENRALILRDKDNTQRLLQEEAEKMKQVAEEAARLSVAAQEAARLRQLAEEDLAQQRALAEKMLKEKM
8iah.1.2    --------------------------------------------------------------------------------

Target      QAVQEATRLKAEAELLQQQKELAQEQARRLQEDKEQMAQQLAQETQGFQRTLETERQRQLEMSAEAERLRLRVAEMSRAQ
8iah.1.2    --------------------------------------------------------------------------------

Target      ARAEEDAQRFRKQAEEIGAKLHRTELATQEKVTLVQTLETQRQQSDRDADRLREAIAELEREKDKLKKEAELLQLKSEEM
8iah.1.2    --------------------------------------------------------------------------------

Target      QTVQQEQLLQETQALQQSFLSEKDSLLQRERFIEEEKAKLERLFQDEVAKAQKLREEQQRQQQMQQEKQQLLASMEEAR
8iah.1.2    --------------------------------------------------------------------------------

Target      RRQHEAEEGVRRKQEELQLLEQQRQQQEQLLAEENRRLRERLEHLEEEHRAALAHSEEITAAQAAATRALPNGQDAADGP
8iah.1.2    --------------------------------------------------------------------------------

Target      AAEPEHAFEGLRQKVPAQQLQEAGILSTEELQRLAQGHTTVAELTQREDVRRYLQGHSSIAGLLLKPANEKLTIYAALRR
8iah.1.2    --------------------------------------------------------------------------------

Target      QLLSPGTALILLEAQAASGFLLDPVRNRRLTVTEAVKEGVVGPELHHKLLSAERAVTGYKDPYTGEKISLFQAMKKDLIV
8iah.1.2    --------------------------------------------------------------------------------

Target      REHGIRLLEAQIATGGVIDPVHSHRVPVDVAYHRGYFDEEMNRVLQDPSDDTKGFFDPNTHENLTYLQLLERCVEDPETG
8iah.1.2    --------------------------------------------------------------------------------

Target      LRLLPLTDQAAKGGELVYTDSEARDVFEKATVSAPFGKFQGKTVTIWELINSEYFTAEQRRDLLRQFRTGKVTVEKIIKI
8iah.1.2    --------------------------------------------------------------------------------

Target      VITVIEEHEQKGQLCFQGLRALVPAAELLESGIIDWDLFRQLQLGERSVQEVAEVEGVRRALRGSGVIAGVWLEEARQKL
8iah.1.2    --------------------------------------------------------------------------------

Target      SIYEALKKELLQPEAAVALLEAQAGTGHVIDPATSARLTVDEAVRAGLVGPELHEKLLSAEKAVTGYKDPYSGQSVSLFQ
8iah.1.2    --------------------------------------------------------------------------------

Target      ALKKGLIPREQGLRLLDAQLSTGGTVDPSKSHRLPLDVACARGYLDEETSTALSAPRDDAKTYYDPRTWELATYSQLQQQ
8iah.1.2    --------------------------------------------------------------------------------

Target      CRPDPLTGLSLLPLSEEAARARQQELYSEVQAREAFQKATVEVPVGSFQGRAVTIWELINSEYFTAEQRQELLRQFRTGK
8iah.1.2    --------------------------------------------------------------------------------

Target      VTVEKIIKIVITIVEEVETTRRERLSFSGLRAPVPASELLAAGILSSSQFEQLKDGKTSVKDLSELSSLQTLLQGSGCLA
8iah.1.2    --------------------------------------------------------------------------------

Target      GIYLEESKEKVTIYEAMRRGLLRPSTAILLLEAQAATGFLVDPVRNQRLYVHEAVKAGVVGPELHEKLLSAEKAVTGYKD
8iah.1.2    --------------------------------------------------------------------------------

Target      PYSGSTISLFQAMKKGLVVREHGIRLLEAQIATGGIIDPVHSHRVPVDVACQRGYFDEEMNRVLEDPSDDTKGFFDPNTH
8iah.1.2    --------------------------------------------------------------------------------

Target      ENLTYRQLLERCVEDPETGLRLLPLKGPEKAEVVETTRVYTEEETRRAFEETQIDIPGGGSHGGSTMSLWEVMQSDLIPE
8iah.1.2    --------------------------------------------------------------------------------

Target      EQRAQLMADFQAGRVTKERMIIIIIEIIEKTEIVRQQNLASYDYVRRRLTAEDLYEARVISRESYSLLREGTRSLREVLE
8iah.1.2    --------------------------------------------------------------------------------

Target      AESAWRYLYGTGCVAGVYLPGSRQTLPIYQALKKGLLSAEVARLLLEAQAATGFLLDPVKGERLTVDEAVRKGLVGPELH
8iah.1.2    --------------------------------------------------------------------------------

Target      DRLLSAERAVTGYRDPYTEQTISLFQAMKKDLIPAEEALRLLDAQLATGGIVDPRLGFHLPLEVAYQRGYLNKDTHDQLS
8iah.1.2    --------------------------------------------------------------------------------

Target      EPSEVRSYVDPSTDERLSYTQLLRRCRRDETSGLFLLPLSEARKLTFRGLRKQITVEELVRSHVMDEATAQRLQEGLTSI
8iah.1.2    --------------------------------------------------------------------------------

Target      EEVSKNLQKFLEGTSSIAGVLVDATKERLSVYQAMKKGIIRPGTAFELLEAQAATGYVIDPIKGLKLTVEEAVRMGIVGP
8iah.1.2    --------------------------------------------------------------------------------
```

```
Target     EFKDKLLSAERAVTGYKDPYSGKLISLFQAMKKGLILKDHGIRLLEAQIATGGIIDPEESHRLPVDVAYQRGLFDEEMNE
8iah.1.2   --------------------------------------------------------------------------------

Target     ILTDPSDDTKGFFDPNTEENLTYLQLMERCVTDPQTGLRLLPLKEKKRERKTSSKSSVRKRRVVIVDPETGKEMSVYEAY
8iah.1.2   --------------------------------------------------------------------------------

Target     RKGLIDHQTYLELSEQECEWEEITISSSDGVVKSMIIDRRSGRQYDIDEAIAKSLIDRSALDQYRAGTLSITEFADMLSG
8iah.1.2   --------------------------------------------------------------------------------

Target     NAGGFRSRSSSVGSSSSYPISPAVSRTQLASWSDPTEETGPVAGILDTETLEKVSITEAMHRNLVDNITGQRLLEAQACT
8iah.1.2   --------------------------------------------------------------------------------

Target     GGIIDPNTGERFPVTDAVNKGLVDKIMVDRINLAQKAFCGFEDPRTKTKMSAAQALKKGWLYYEAGQRFLEVQYLTGGLI
8iah.1.2   --------------------------------------------------------------------------------

Target     EPDTPGRVPLDEALQRGTVDARTAQKLRDVSAYSKYLTCPKTKLKISYKDALDRSMVEEGTGLRLLEAAAQSSKGYYSPY
8iah.1.2   --------------------------------------------------------------------------------

Target     SVSGSGSTTGSRSGSRTGSRAGSRRGSFDATGSGFSMTFSSSSYSSSGYGRRYASGPTSSLGGPESAAA
8iah.1.2   ---------------------------------------------------------------------
```

Model #17	File	Built with	Oligo-State	Ligands	GMQE	QMEANDisCo Global
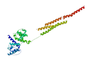	PDB	ProMod3 3.3.0	monomer (matching prediction)	None	0.00	0.65 ± 0.05

Template	Seq Identity	Oligo-state	QSQE	Found by	Method	Resolution	Seq Similarity	Range	Coverage	Description
6sl3.1.A	29.82	homo-dimer	0.00	HHblits	X-ray	3.10Å	0.36	62 - 448	0.08	Calponin homology domain protein putative

Excluded ligands

Ligand Name.Number	Reason for Exclusion	Description
CA.1	Binding site not conserved.	CALCIUM ION
CA.2	Binding site not conserved.	CALCIUM ION

```
Target     MSGEDQEVRAVVEDGSNGGSGSPSPGDTLPWNLEKTQRSRRGGGGPGGNGSVLDPAERAVIRIADERDRVQKKTFTKWVN
6sl3.1.A   -------------------------------------------------------------ELVAQWEKVQIKTFTKWVN

Target     KHLIKAQRHISDLYEDLRDGHNLISLLEVLSGDSLPR-EKGRMRFHKLQNVQIALDYLRHRQVKLVNIRNDDIADGNPKL
6sl3.1.A   MHLAKKGRKINDVTTDFKNGVELCALLEIIGETTIKCVTNPKMRIQMTENLDKALRFIQSRDVKLTGIGPTDIVDGNVKL

Target     TLGLIWTIILHFQISDIQVSGQSEDMTAKEKLLLWSQRMVEGYQGLRCDNFTSSWRDGRLFNAIIHRHKPMLIDMNKVYR
6sl3.1.A   TLGLVWTLILRFAISELSAE----GLSAKQGLLLWCQKKCEPYP-VKVENFSESFKDGKVFCALIHRHRPDLLDWETVGE

Target     QTNLENLDQAFSVAERDLGVTRLLDPEDVDV-PQPDEKSIITYVSSLYDAMPRVPDVQDGVK--------ANELQLRWQE
6sl3.1.A   D-DRANLEKAFDVAEKELGIPKLLDVDDIVNMPRPDERSVMTYVAALYKVFSSNDQVEKAGKRAGNFLDLLRATEGMVHD

Target     YRELVLLLLQWIRAHTAAFEERRFPSSFEEIEILWCQFLKFKETELPAKEADKNRSKGIYQSLEGAVQAG---QLKVPPG
6sl3.1.A   YEQRAQALKENIEAAINKMNGVEPSDEYHQVKEQINETKNYRKGDKRAFIKEQGDLATLFGQINSKLRGMKRPVYVAPEG

Target     YHPLDVEKEWGKLHVAILEREKQLRSEFERLERLQRIVSKLQMEAGLCEEQLNQADALLQSDVRLLAAGKAPQRAGEVER
6sl3.1.A   LDPKSLEGYIANISEAERALRSKLNTAMR--NCLIALRKAFADPANATDAKINEYRTFVTD-------------------

Target     DLDKADGMIRLLFNDVQALKDGRHPQGEQMYRRVYRLHERLVAIRTEYNLRLRGTPRHPELEDSTLRYLQDLLAWVEENQ
6sl3.1.A   --------------------------------------------------------------------------------

Target     RRVDSAEWGVDLPSVEAQLGSHRGLHQSVEEFRAKIERARTDEGQLSPATRGAYRDCLGRLDLQYAKLLNSSKARLRSLE
6sl3.1.A   --------------------------------------------------------------------------------

Target     SLHGFVAAATKELMWLSEKEEEEVGFDWSERNSNMAAKKEAYSALMRELELKEKKIKEIQSTGDRLLREDHPARPTVESF
6sl3.1.A   --------------------------------------------------------------------------------

Target     QAALQTQWSWMLQLCCCIEAHLKENTAYFQFFSDVREAEEQLRKLQETLHRKYTCDRSITVTRLEDLLQDAQDEKDQLNE
```

```
6sl3.1.A    --------------------------------------------------------------------------------

Target      YRGHLSGLAKRAKAIVQLTPRNPTQPTRGRVPLLAVCDYKQVEATVHKGDECQMLGPAQPFHWKVLSSSGSEAAVPSVCF
6sl3.1.A    --------------------------------------------------------------------------------

Target      LVPPPNQEALEAVARLEAQHQALVTLWHQLHTDMKSLLAWQSLSRDVQLIRSWSLVTFRTLKPEEQRQALRSLELHYQAF
6sl3.1.A    --------------------------------------------------------------------------------

Target      LRDSQDAGGFGPEDRLQAEREYGSCSRHYQQLLQSLEQGEQEESRCQRCISELKDIRLQLEACETRTVHRLRLPLDKEPA
6sl3.1.A    --------------------------------------------------------------------------------

Target      RECAQRIAEQQKAQAEVEGLGKGVARLSAEAEKVLALPEPSPAAPTLRSELELTLGKLEQVRSLSAIYLEKLKTISLVIR
6sl3.1.A    --------------------------------------------------------------------------------

Target      STQGAEEALRAHEEQLKEAQAVPAALPELEATKAAMKKLRAQAEAQQPVFDALRDELRGAQEVGERLQQRHGERDVEVER
6sl3.1.A    --------------------------------------------------------------------------------

Target      WRERVTQLLERWQAVLAQTDVRQRELEQLGRQLRYYRESADPLGAWLQDARRRQEQIQAVPLADSQAVREQLRQEKALLE
6sl3.1.A    --------------------------------------------------------------------------------

Target      EIERHAEKVEECQRFAKQYINAIKDYELQLVTYKAQLEPVASPAKKPKVQSGSESVIQEYVDLRTRYSELSTLTSQYIRF
6sl3.1.A    --------------------------------------------------------------------------------

Target      ISETLRRMEEEERLAEQQRAEERERLAEVEAALEKQRQLAEAHAQAKAQAEREAQELQRRMQEEVARREEVAVDAQQQKR
6sl3.1.A    --------------------------------------------------------------------------------

Target      SIQEELQQLRQSSEAEIQAKARQVEAAERSRLRIEEEIRVVRLQLETTERQRGGAEGELQALRARAEEAEAQKRQAQEEA
6sl3.1.A    --------------------------------------------------------------------------------

Target      ERLRRQVQEETQRKRQAEAELGLRVKAEAEAAREKQRALQALEELRLQAEEAERRLRQAEAERARQVQVALETAQRSAQA
6sl3.1.A    --------------------------------------------------------------------------------

Target      ELQSKHASFAEKTAQLERTLEEEHVTVVQLREEATRREQQQAEAERAREEAERELERWQLKANEALRLRLQAEEVAQQKS
6sl3.1.A    --------------------------------------------------------------------------------

Target      LAQAEAEKQKEAAEREARRRGKAEEQAVRQRELAEQELERQRQLAEGTAQQRLAAEQELIRLRAETEQGEQQRQLLEEEL
6sl3.1.A    --------------------------------------------------------------------------------

Target      ARLQSEAAAATQKRQELEAELAKVRAEMEVLLASKARAEEESRSSSEKSKQRLEAEAGRFRELAEEAARLRALAEEAKRQ
6sl3.1.A    --------------------------------------------------------------------------------

Target      RQLAEEDAARQRAEAERVLSEKLAAISEATRLKTEAEIALKEKEAENERLRRLAEDEAFQRRRLEEQAAQHKADIEERLA
6sl3.1.A    --------------------------------------------------------------------------------

Target      QLRKASESELERQKGLVEDTLRQRRQVEEEILALKASFEKAAAGKAELELELGRIRGNAEDTLRSKEQAEQEAARQRQLA
6sl3.1.A    --------------------------------------------------------------------------------

Target      AEEERRRREAEERVQKSLAAEEEAARQRKAALEEVERLKAKVEEARRLRERAEHESVRQLQLAQEAAQKRLQAEEKAHAF
6sl3.1.A    --------------------------------------------------------------------------------

Target      AVQQKEQELQQTLQQEQSVLEKLRSEAEAARRAAEEAEEARERAEREAAQSRQRVEEAERLKQAAEEQAQAQAQAQAAAE
6sl3.1.A    --------------------------------------------------------------------------------

Target      KLRKEAEQEAARRAQAEQAALRQKQAADAEMEKHKKFAEQTLRQKAQVEQELTALRLKLEETDHQKSILDQELQRLKAEV
6sl3.1.A    --------------------------------------------------------------------------------

Target      TEAARQRSQVEEELFSVRVQMEELGKLKARIEAENRALILRDKDNTQRLLQEEAEKMKQVAEEAARLSVAAQEAARLRQL
6sl3.1.A    --------------------------------------------------------------------------------

Target      AEEDLAQQRALAEKMLKEKMQAVQEATRLKAEAELLQQQKELAQEQARRLQEDKEQMAQQLAQETQGFQRTLETERQRQL
6sl3.1.A    --------------------------------------------------------------------------------

Target      EMSAEAERLRLRVAEMSRAQARAEEDAQRFRKQAEEIGAKLHRTELATQEKVTLVQTLETQRQQSDRDADRLREAIAELE
6sl3.1.A    --------------------------------------------------------------------------------

Target      REKDKLKKEAELLQLKSEEMQTVQQEQLLQETQALQQSFLSEKDSLLQRERFIEEEKAKLERLFQDEVAKAQKLREEQQR
6sl3.1.A    --------------------------------------------------------------------------------

Target      QQQQMQQEKQQLLASMEEARRRQHEAEEGVRRKQEELQLLEQQRQQQEQLLAEENRRLRERLEHLEEEHRAALAHSEEIT
6sl3.1.A    --------------------------------------------------------------------------------

Target      AAQAAATRALPNGQDAADGPAAEPEHAFEGLRQKVPAQQLQEAGILSTEELQRLAQGHTTVAELTQREDVRRYLQGHSSI
6sl3.1.A    --------------------------------------------------------------------------------
```

```
Target     AGLLLKPANEKLTIYAALRRQLLSPGTALILLEAQAASGFLLDPVRNRRLTVTEAVKEGVVGPELHHKLLSAERAVTGYK
6sl3.1.A   --------------------------------------------------------------------------------

Target     DPYTGEKISLFQAMKKDLIVREHGIRLLEAQIATGGVIDPVHSHRVPVDVAYHRGYFDEEMNRVLQDPSDDTKGFFDPNT
6sl3.1.A   --------------------------------------------------------------------------------

Target     HENLTYLQLLERCVEDPETGLRLLPLTDQAAKGGELVYTDSEARDVFEKATVSAPFGKFQGKTVTIWELINSEYFTAEQR
6sl3.1.A   --------------------------------------------------------------------------------

Target     RDLLRQFRTGKVTVEKIIKIVITVIEEHEQKGQLCFQGLRALVPAAELLESGIIDWDLFRQLQLGERSVQEVAEVEGVRR
6sl3.1.A   --------------------------------------------------------------------------------

Target     ALRGSGVIAGVWLEEARQKLSIYEALKKELLQPEAAVALLEAQAGTGHVIDPATSARLTVDEAVRAGLVGPELHEKLLSA
6sl3.1.A   --------------------------------------------------------------------------------

Target     EKAVTGYKDPYSGQSVSLFQALKKGLIPREQGLRLLDAQLSTGGTVDPSKSHRLPLDVACARGYLDEETSTALSAPRDDA
6sl3.1.A   --------------------------------------------------------------------------------

Target     KTYYDPRTWELATYSQLQQQCRPDPLTGLSLLPLSEEAARARQQELYSEVQAREAFQKATVEVPVGSFQGRAVTIWELIN
6sl3.1.A   --------------------------------------------------------------------------------

Target     SEYFTAEQRQELLRQFRTGKVTVEKIIKIVITIVEEVETTRRERLSFSGLRAPVPASELLAAGILSSSQFEQLKDGKTSV
6sl3.1.A   --------------------------------------------------------------------------------

Target     KDLSELSSLQTLLQGSGCLAGIYLEESKEKVTIYEAMRRGLLRPSTAILLLEAQAATGFLVDPVRNQRLYVHEAVKAGVV
6sl3.1.A   --------------------------------------------------------------------------------

Target     GPELHEKLLSAEKAVTGYKDPYSGSTISLFQAMKKGLVVREHGIRLLEAQIATGGIIDPVHSHRVPVDVACQRGYFDEEM
6sl3.1.A   --------------------------------------------------------------------------------

Target     NRVLEDPSDDTKGFFDPNTHENLTYRQLLERCVEDPETGLRLLPLKGPEKAEVVETTRVYTEEETRRAFEETQIDIPGGG
6sl3.1.A   --------------------------------------------------------------------------------

Target     SHGGSTMSLWEVMQSDLIPEEQRAQLMADFQAGRVTKERMIIIIIEIIEKTEIVRQQNLASYDYVRRRLTAEDLYEARVI
6sl3.1.A   --------------------------------------------------------------------------------

Target     SRESYSLLREGTRSLREVLEAESAWRYLYGTGCVAGVYLPGSRQTLPIYQALKKGLLSAEVARLLLEAQAATGFLLDPVK
6sl3.1.A   --------------------------------------------------------------------------------

Target     GERLTVDEAVRKGLVGPELHDRLLSAERAVTGYRDPYTEQTISLFQAMKKDLIPAEEALRLLDAQLATGGIVDPRLGFHL
6sl3.1.A   --------------------------------------------------------------------------------

Target     PLEVAYQRGYLNKDTHDQLSEPSEVRSYVDPSTDERLSYTQLLRRCRRDETSGLFLLPLSEARKLTFRGLRKQITVEELV
6sl3.1.A   --------------------------------------------------------------------------------

Target     RSHVMDEATAQRLQEGLTSIEEVSKNLQKFLEGTSSIAGVLVDATKERLSVYQAMKKGIIRPGTAFELLEAQAATGYVID
6sl3.1.A   --------------------------------------------------------------------------------

Target     PIKGLKLTVEEAVRMGIVGPEFKDKLLSAERAVTGYKDPYSGKLISLFQAMKKGLILKDHGIRLLEAQIATGGIIDPEES
6sl3.1.A   --------------------------------------------------------------------------------

Target     HRLPVDVAYQRGLFDEEMNEILTDPSDDTKGFFDPNTEENLTYLQLMERCVTDPQTGLRLLPLKEKKRERKTSSKSSVRK
6sl3.1.A   --------------------------------------------------------------------------------

Target     RRVVIVDPETGKEMSVYEAYRKGLIDHQTYLELSEQECEWEEITISSSDGVVKSMIIDRRSGRQYDIDEAIAKSLIDRSA
6sl3.1.A   --------------------------------------------------------------------------------

Target     LDQYRAGTLSITEFADMLSGNAGGFRSRSSSVGSSSSYPISPAVSRTQLASWSDPTEETGPVAGILDTETLEKVSITEAM
6sl3.1.A   --------------------------------------------------------------------------------

Target     HRNLVDNITGQRLLEAQACTGGIIDPNTGERFPVTDAVNKGLVDKIMVDRINLAQKAFCGFEDPRTKTKMSAAQALKKGW
6sl3.1.A   --------------------------------------------------------------------------------

Target     LYYEAGQRFLEVQYLTGGLIEPDTPGRVPLDEALQRGTVDARTAQKLRDVSAYSKYLTCPKTKLKISYKDALDRSMVEEG
6sl3.1.A   --------------------------------------------------------------------------------

Target     TGLRLLEAAAQSSKGYYSPYSVSGSGSTTGSRSGSRTGSRAGSRRGSFDATGSGFSMTFSSSSYSSSGYGRRYASGPTSS
6sl3.1.A   --------------------------------------------------------------------------------

Target     LGGPESAAA
6sl3.1.A   ---------
```

Model #08	File	Built with	Oligo-State	Ligands	GMQE	QMEANDisCo Global
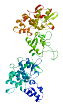	PDB	ProMod3 3.3.0	monomer	None	0.00	0.63 ± 0.05

Template	Seq Identity	Oligo-state	QSQE	Found by	Method	Resolution	Seq Similarity	Range	Coverage	Description
5dzz.1.A	47.12	monomer	0.00	HHblits	X-ray	2.60Å	0.42	2649 - 3222	0.11	Desmoplakin

The template contained no ligands.

```
Target    MSGEDQEVRAVVEDGSNGGSGSPSPGDTLPWNLEKTQRSRRGGGGPGGNGSVLDPAERAVIRIADERDRVQKKTFTKWVN
5dzz.1.A  --------------------------------------------------------------------------------

Target    KHLIKAQRHISDLYEDLRDGHNLISLLEVLSGDSLPREKGRMRFHKLQNVQIALDYLRHRQVKLVNIRNDDIADGNPKLT
5dzz.1.A  --------------------------------------------------------------------------------

Target    LGLIWTIILHFQISDIQVSGQSEDMTAKEKLLLWSQRMVEGYQGLRCDNFTSSWRDGRLFNAIIHRHKPMLIDMNKVYRQ
5dzz.1.A  --------------------------------------------------------------------------------

Target    TNLENLDQAFSVAERDLGVTRLLDPEDVDVPQPDEKSIITYVSSLYDAMPRVPDVQDGVKANELQLRWQEYRELVLLLLQ
5dzz.1.A  --------------------------------------------------------------------------------

Target    WIRAHTAAFEERRFPSSFEEIEILWCQFLKFKETELPAKEADKNRSKGIYQSLEGAVQAGQLKVPPGYHPLDVEKEWGKL
5dzz.1.A  --------------------------------------------------------------------------------

Target    HVAILEREKQLRSEFERLERLQRIVSKLQMEAGLCEEQLNQADALLQSDVRLLAAGKAPQRAGEVERDLDKADGMIRLLF
5dzz.1.A  --------------------------------------------------------------------------------

Target    NDVQALKDGRHPQGEQMYRRVYRLHERLVAIRTEYNLRLRGTPRHPELEDSTLRYLQDLLAWVEENQRRVDSAEWGVDLP
5dzz.1.A  --------------------------------------------------------------------------------

Target    SVEAQLGSHRGLHQSVEEFRAKIERARTDEGQLSPATRGAYRDCLGRLDLQYAKLLNSSKARLRSLESLHGFVAAATKEL
5dzz.1.A  --------------------------------------------------------------------------------

Target    MWLSEKEEEEVGFDWSERNSNMAAKKEAYSALMRELELKEKKIKEIQSTGDRLLREDHPARPTVESFQAALQTQWSWMLQ
5dzz.1.A  --------------------------------------------------------------------------------

Target    LCCCIEAHLKENTAYFQFFSDVREAEEQLRKLQETLHRKYTCDRSITVTRLEDLLQDAQDEKDQLNEYRGHLSGLAKRAK
5dzz.1.A  --------------------------------------------------------------------------------

Target    AIVQLTPRNPTQPTRGRVPLLAVCDYKQVEATVHKGDECQMLGPAQPFHWKVLSSSGSEAAVPSVCFLVPPPNQEALEAV
5dzz.1.A  --------------------------------------------------------------------------------

Target    ARLEAQHQALVTLWHQLHTDMKSLLAWQSLSRDVQLIRSWSLVTFRTLKPEEQRQALRSLELHYQAFLRDSQDAGGFGPE
5dzz.1.A  --------------------------------------------------------------------------------

Target    DRLQAEREYGSCSRHYQQLLQSLEQGEQEESRCQRCISELKDIRLQLEACETRTVHRLRLPLDKEPARECAQRIAEQQKA
5dzz.1.A  --------------------------------------------------------------------------------

Target    QAEVEGLGKGVARLSAEAEKVLALPEPSPAAPTLRSELELTLGKLEQVRSLSAIYLEKLKTISLVIRSTQGAEEALRAHE
5dzz.1.A  --------------------------------------------------------------------------------

Target    EQLKEAQAVPAALPELEATKAAMKKLRAQAEAQQPVFDALRDELRGAQEVGERLQQRHGERDVEVERWRERVTQLLERWQ
5dzz.1.A  --------------------------------------------------------------------------------

Target    AVLAQTDVRQRELEQLGRQLRYYRESADPLGAWLQDARRRQEQIQAVPLADSQAVREQLRQEKALLEEIERHAEKVEECQ
5dzz.1.A  --------------------------------------------------------------------------------

Target    RFAKQYINAIKDYELQLVTYKAQLEPVASPAKKPKVQSGSESVIQEYVDLRTRYSELSTLTSQYIRFISETLRRMEEEER
5dzz.1.A  --------------------------------------------------------------------------------

Target    LAEQQRAEERERLAEVEAALEKQRQLAEAHAQAKAQAEREAQELQRRMQEEVARREEVAVDAQQQKRSIQEELQQLRQSS
5dzz.1.A  --------------------------------------------------------------------------------

Target    EAEIQAKARQVEAAERSRLRIEEEIRVVRLQLETTERQRGGAEGELQALRARAEEAEAQKRQAQEEAERLRRQVQEETQR
5dzz.1.A  --------------------------------------------------------------------------------
```

```
Target     KRQAEAELGLRVKAEAEAAREKQRALQALEELRLQAEEAERRLRQAEAERARQVQVALETAQRSAQAELQSKHASFAEKT
5dzz.1.A   --------------------------------------------------------------------------------

Target     AQLERTLEEEHVTVVQLREEATRREQQQAEAERAREEAERELERWQLKANEALRLRLQAEEVAQQKSLAQAEAEKQKEAA
5dzz.1.A   --------------------------------------------------------------------------------

Target     EREARRRGKAEEQAVRQRELAEQELERQRQLAEGTAQQRLAAEQELIRLRAETEQGEQQRQLLEEELARLQSEAAAATQK
5dzz.1.A   --------------------------------------------------------------------------------

Target     RQELEAELAKVRAEMEVLLASKARAEEESRSSSEKSKQRLEAEAGRFRELAEEAARLRALAEEAKRQRQLAEEDAARQRA
5dzz.1.A   --------------------------------------------------------------------------------

Target     EAERVLSEKLAAISEATRLKTEAEIALKEKEAENERLRRLAEDEAFQRRRLEEQAAQHKADIEERLAQLRKASESELERQ
5dzz.1.A   --------------------------------------------------------------------------------

Target     KGLVEDTLRQRRQVEEEILALKASFEKAAAGKAELELELGRIRGNAEDTLRSKEQAEQEAARQRQLAAEEERRRREAEER
5dzz.1.A   --------------------------------------------------------------------------------

Target     VQKSLAAEEEAARQRKAALEEVERLKAKVEEARRLRERAEHESVRQLQLAQEAAQKRLQAEEKAHAFAVQQKEQELQQTL
5dzz.1.A   --------------------------------------------------------------------------------

Target     QQEQSVLEKLRSEAEAARRAAEEAEEARERAEREAAQSRQRVEEAERLKQAAEEQAQAQAQAQAAAEKLRKEAEQEAARR
5dzz.1.A   --------------------------------------------------------------------------------

Target     AQAEQAALRQKQAADAEMEKHKKFAEQTLRQKAQVEQELTALRLKLEETDHQKSILDQELQRLKAEVTEAARQRSQVEEE
5dzz.1.A   --------------------------------------------------------------------------------

Target     LFSVRVQMEELGKLKARIEAENRALILRDKDNTQRLLQEEAEKMKQVAEEAARLSVAAQEAARLRQLAEEDLAQQRALAE
5dzz.1.A   --------------------------------------------------------------------------------

Target     KMLKEKMQAVQEATRLKAEAELLQQQKELAQEQARRLQEDKEQMAQQLAQETQGFQRTLETERQRQLEMSAEAERLRLRV
5dzz.1.A   --------------------------------------------------------------------------------

Target     AEMSRAQARAEEDAQRFRKQAEEIGAKLHRTELATQEKVTLVQTLETQRQQSDRDADRLREAIAELEREKDKLKKEAELL
5dzz.1.A   --------------------------------------------------------------------------------

Target     QLKSEEMQTVQQEQLLQETQALQQSFLSEKDSLLQRERFIEEEKAKLERLFQDEVAKAQKLREEQQRQQQMQQEKQQLL
5dzz.1.A   --------------------------------------------------------------------------------

Target     ASMEEARRRQHEAEEGVRRKQEELQLLEQQRQQQEQLLAEENRRLRERLEHLEEEHRAALAHSEEITAAQAAATRALPNG
5dzz.1.A   --------------------------------------------------------------------------------

Target     QDAADGPAAEPEHAFEGLRQKVPAQQLQEAGILSTEELQRLAQGHTTVAELTQREDVRRYLQGHSSIAGLLLKPANEKLT
5dzz.1.A   --------DTSKLVFDGLRKKVTAMQLYECQLIDKTTLDKLLKGKKSVEEVA--SEIQPFLRGAGSIAGAS-ASPKEKYS

Target     IYAALRRQLLSPGTALILLEAQAASGFLLDPVRNRRLTVTEAVKEGVVGPELHHKLLSAERAVTGYKDPYTGEKISLFQA
5dzz.1.A   LVEAKRKKLISPESTVMLLEAQAATGGIIDPHRNEKLTVDSAIARDLIDFDDRQQIYAAEKAITGFDDPFSGKTVSVSEA

Target     MKKDLIVREHGIRLLEAQIATGGVIDPVHSHRVPVDVAYHRGYFDEEMNRVLQDPSDDTKGFFDPNTHENLTYLQLLERC
5dzz.1.A   IKKNLIDRETGMRLLEAQIASGGVVDPVNSVFLPKDVALARGLIDRDLYRSLNDPRDSQKNFVDPVTKKKVSYVQLKERC

Target     VEDPETGLRLLPLTDQAAKGGELVYTDSEARDVFEKATVSAPFGKFQGKTVTIWELINSEYFTAEQRRDLLRQFRTGKVT
5dzz.1.A   RIEPHTGLLLLSVQKR----------------------------------------------------------------

Target     VEKIIKIVITVIEEHEQKGQLCFQGLRALVPAAELLESGIIDWDLFRQLQLGERSVQEVAEVEGVRRALRGSGVIAGVWL
5dzz.1.A   -------------------SMSFQGIRQPVTVTELVDSGILRPSTVNELESGQISYDEVGE--RIKDFLQGSSCIAGIYN

Target     EEARQKLSIYEALKKELLQPEAAVALLEAQAGTGHVIDPATSARLTVDEAVRAGLVGPELHEKLLSAEKAVTGYKDPYSG
5dzz.1.A   ETTKQKLGIYEAMKIGLVRPGTALELLEAQAATGFIVDPVSNLRLPVEEAYKRGLVGIEFKEKLLSAERAVTGYNDPETG

Target     QSVSLFQALKKGLIPREQGLRLLDAQLSTGGTVDPSKSHRLPLDVACARGYLDEETSTALSAPRDDAKTYYDPRTWELAT
5dzz.1.A   NIISLFQAMNKELIEKGHGIRLLEAQIATGGIIDPKESHRLPVDIAYKRGYFNEELSEILSDPSDDTKGFFDPNTEENLT

Target     YSQLQQQCRPDPLTGLSLLPLSEEAARARQQELYSEVQAREAFQKATVEVPVGSFQGRAVTIWELINSEYFTAEQRQELL
5dzz.1.A   YLQLKERCIKDEETGLCLLPLK----------------------------------------------------------

Target     RQFRTGKVTVEKIIKIVITIVEEVETTRRERLSFSGLRAPVPASELLAAGILSSSQFEQLKDGKTSVKDLSELSSLQTLL
5dzz.1.A   --------------------------------------------------------------------------------

Target     QGSGCLAGIYLEESKEKVTIYEAMRRGLLRPSTAILLLEAQAATGFLVDPVRNQRLYVHEAVKAGVVGPELHEKLLSAEK
5dzz.1.A   --------------------------------------------------------------------------------

Target     AVTGYKDPYSGSTISLFQAMKKGLVVREHGIRLLEAQIATGGIIDPVHSHRVPVDVACQRGYFDEEMNRVLEDPSDDTKG
```

```
5dzz.1.A   --------------------------------------------------------------------------------

Target     FFDPNTHENLTYRQLLERCVEDPETGLRLLPLKGPEKAEVVETTRVYTEEETRRAFEETQIDIPGGGSHGGSTMSLWEVM
5dzz.1.A   --------------------------------------------------------------------------------

Target     QSDLIPEEQRAQLMADFQAGRVTKERMIIIIIEIIEKTEIVRQQNLASYDYVRRRLTAEDLYEARVISRESYSLLREGTR
5dzz.1.A   --------------------------------------------------------------------------------

Target     SLREVLEAESAWRYLYGTGCVAGVYLPGSRQTLPIYQALKKGLLSAEVARLLLEAQAATGFLLDPVKGERLTVDEAVRKG
5dzz.1.A   --------------------------------------------------------------------------------

Target     LVGPELHDRLLSAERAVTGYRDPYTEQTISLFQAMKKDLIPAEEALRLLDAQLATGGIVDPRLGFHLPLEVAYQRGYLNK
5dzz.1.A   --------------------------------------------------------------------------------

Target     DTHDQLSEPSEVRSYVDPSTDERLSYTQLLRRCRRDETSGLFLLPLSEARKLTFRGLRKQITVEELVRSHVMDEATAQRL
5dzz.1.A   --------------------------------------------------------------------------------

Target     QEGLTSIEEVSKNLQKFLEGTSSIAGVLVDATKERLSVYQAMKKGIIRPGTAFELLEAQAATGYVIDPIKGLKLTVEEAV
5dzz.1.A   --------------------------------------------------------------------------------

Target     RMGIVGPEFKDKLLSAERAVTGYKDPYSGKLISLFQAMKKGLILKDHGIRLLEAQIATGGIIDPEESHRLPVDVAYQRGL
5dzz.1.A   --------------------------------------------------------------------------------

Target     FDEEMNEILTDPSDDTKGFFDPNTEENLTYLQLMERCVTDPQTGLRLLPLKEKKRERKTSSKSSVRKRRVVIVDPETGKE
5dzz.1.A   --------------------------------------------------------------------------------

Target     MSVYEAYRKGLIDHQTYLELSEQECEWEEITISSSDGVVKSMIIDRRSGRQYDIDEAIAKSLIDRSALDQYRAGTLSITE
5dzz.1.A   --------------------------------------------------------------------------------

Target     FADMLSGNAGGFRSRSSSVGSSSSYPISPAVSRTQLASWSDPTEETGPVAGILDTETLEKVSITEAMHRNLVDNITGQRL
5dzz.1.A   --------------------------------------------------------------------------------

Target     LEAQACTGGIIDPNTGERFPVTDAVNKGLVDKIMVDRINLAQKAFCGFEDPRTKTKMSAAQALKKGWLYYEAGQRFLEVQ
5dzz.1.A   --------------------------------------------------------------------------------

Target     YLTGGLIEPDTPGRVPLDEALQRGTVDARTAQKLRDVSAYSKYLTCPKTKLKISYKDALDRSMVEEGTGLRLLEAAAQSS
5dzz.1.A   --------------------------------------------------------------------------------

Target     KGYYSPYSVSGSGSTTGSRSGSRTGSRAGSRRGSFDATGSGFSMTFSSSSYSSSGYGRRYASGPTSSLGGPESAAA
5dzz.1.A   ----------------------------------------------------------------------------
```

Model #10	File	Built with	Oligo-State	Ligands	GMQE	QMEANDisCo Global
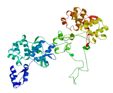	PDB	ProMod3 3.3.0	monomer	None	0.00	0.59 ± 0.05

Template	Seq Identity	Oligo-state	QSQE	Found by	Method	Resolution	Seq Similarity	Range	Coverage	Description
5dzz.1.A	38.07	monomer	0.00	HHblits	X-ray	2.60Å	0.38	3888 - 4476	0.11	Desmoplakin

The template contained no ligands.

```
Target     MSGEDQEVRAVVEDGSNGGSGSPSPGDTLPWNLEKTQRSRRGGGGPGGNGSVLDPAERAVIRIADERDRVQKKTFTKWVN
5dzz.1.A   --------------------------------------------------------------------------------

Target     KHLIKAQRHISDLYEDLRDGHNLISLLEVLSGDSLPREKGRMRFHKLQNVQIALDYLRHRQVKLVNIRNDDIADGNPKLT
5dzz.1.A   --------------------------------------------------------------------------------

Target     LGLIWTIILHFQISDIQVSGQSEDMTAKEKLLLWSQRMVEGYQGLRCDNFTSSWRDGRLFNAIIHRHKPMLIDMNKVYRQ
5dzz.1.A   --------------------------------------------------------------------------------

Target     TNLENLDQAFSVAERDLGVTRLLDPEDVDVPQPDEKSIITYVSSLYDAMPRVPDVQDGVKANELQLRWQEYRELVLLLLQ
5dzz.1.A   --------------------------------------------------------------------------------

Target     WIRAHTAAFEERRFPSSFEEIEILWCQFLKFKETELPAKEADKNRSKGIYQSLEGAVQAGQLKVPPGYHPLDVEKEWGKL
```

```
5dzz.1.A    --------------------------------------------------------------------------------

Target      HVAILEREKQLRSEFERLERLQRIVSKLQMEAGLCEEQLNQADALLQSDVRLLAAGKAPQRAGEVERDLDKADGMIRLLF
5dzz.1.A    --------------------------------------------------------------------------------

Target      NDVQALKDGRHPQGEQMYRRVYRLHERLVAIRTEYNLRLRGTPRHPELEDSTLRYLQDLLAWVEENQRRVDSAEWGVDLP
5dzz.1.A    --------------------------------------------------------------------------------

Target      SVEAQLGSHRGLHQSVEEFRAKIERARTDEGQLSPATRGAYRDCLGRLDLQYAKLLNSSKARLRSLESLHGFVAAATKEL
5dzz.1.A    --------------------------------------------------------------------------------

Target      MWLSEKEEEEVGFDWSERNSNMAAKKEAYSALMRELELKEKKIKEIQSTGDRLLREDHPARPTVESFQAALQTQWSWMLQ
5dzz.1.A    --------------------------------------------------------------------------------

Target      LCCCIEAHLKENTAYFQFFSDVREAEEQLRKLQETLHRKYTCDRSITVTRLEDLLQDAQDEKDQLNEYRGHLSGLAKRAK
5dzz.1.A    --------------------------------------------------------------------------------

Target      AIVQLTPRNPTQPTRGRVPLLAVCDYKQVEATVHKGDECQMLGPAQPFHWKVLSSSGSEAAVPSVCFLVPPPNQEALEAV
5dzz.1.A    --------------------------------------------------------------------------------

Target      ARLEAQHQALVTLWHQLHTDMKSLLAWQSLSRDVQLIRSWSLVTFRTLKPEEQRQALRSLELHYQAFLRDSQDAGGFGPE
5dzz.1.A    --------------------------------------------------------------------------------

Target      DRLQAEREYGSCSRHYQQLLQSLEQGEQEESRCQRCISELKDIRLQLEACETRTVHRLRLPLDKEPARECAQRIAEQQKA
5dzz.1.A    --------------------------------------------------------------------------------

Target      QAEVEGLGKGVARLSAEAEKVLALPEPSPAAPTLRSELELTLGKLEQVRSLSAIYLEKLKTISLVIRSTQGAEEALRAHE
5dzz.1.A    --------------------------------------------------------------------------------

Target      EQLKEAQAVPAALPELEATKAAMKKLRAQAEAQQPVFDALRDELRGAQEVGERLQQRHGERDVEVERWRERVTQLLERWQ
5dzz.1.A    --------------------------------------------------------------------------------

Target      AVLAQTDVRQRELEQLGRQLRYYRESADPLGAWLQDARRRQEQIQAVPLADSQAVREQLRQEKALLEEIERHAEKVEECQ
5dzz.1.A    --------------------------------------------------------------------------------

Target      RFAKQYINAIKDYELQLVTYKAQLEPVASPAKKPKVQSGSESVIQEYVDLRTRYSELSTLTSQYIRFISETLRRMEEEER
5dzz.1.A    --------------------------------------------------------------------------------

Target      LAEQQRAEERERLAEVEAALEKQRQLAEAHAQAKAQAEREAQELQRRMQEEVARREEVAVDAQQQKRSIQEELQQLRQSS
5dzz.1.A    --------------------------------------------------------------------------------

Target      EAEIQAKARQVEAAERSRLRIEEEIRVVRLQLETTERQRGGAEGELQALRARAEEAEAQKRQAQEEAERLRRQVQEETQR
5dzz.1.A    --------------------------------------------------------------------------------

Target      KRQAEAELGLRVKAEAEAAREKQRALQALEELRLQAEEAERRLRQAEAERARQVQVALETAQRSAQAELQSKHASFAEKT
5dzz.1.A    --------------------------------------------------------------------------------

Target      AQLERTLEEEHVTVVQLREEATRREQQQAEAERAREEAERELERWQLKANEALRLRLQAEEVAQQKSLAQAEAEKQKEAA
5dzz.1.A    --------------------------------------------------------------------------------

Target      EREARRRGKAEEQAVRQRELAEQELERQRQLAEGTAQQRLAAEQELIRLRAETEQGEQQRQLLEEELARLQSEAAAATQK
5dzz.1.A    --------------------------------------------------------------------------------

Target      RQELEAELAKVRAEMEVLLASKARAEEESRSSSEKSKQRLEAEAGRFRELAEEAARLRALAEEAKRQRQLAEEDAARQRA
5dzz.1.A    --------------------------------------------------------------------------------

Target      EAERVLSEKLAAISEATRLKTEAEIALKEKEAENERLRRLAEDEAFQRRRLEEQAAQHKADIEERLAQLRKASESELERQ
5dzz.1.A    --------------------------------------------------------------------------------

Target      KGLVEDTLRQRRQVEEEILALKASFEKAAAGKAELELELGRIRGNAEDTLRSKEQAEQEAARQRQLAAEEERRRREAEER
5dzz.1.A    --------------------------------------------------------------------------------

Target      VQKSLAAEEEAARQRKAALEEVERLKAKVEEARRLRERAEHESVRQLQLAQEAAQKRLQAEEKAHAFAVQQKEQELQQTL
5dzz.1.A    --------------------------------------------------------------------------------

Target      QQEQSVLEKLRSEAEAARRAAEEAEEARERAEREAAQSRQRVEEAERLKQAAEEQAQAQAQAQAAAEKLRKEAEQEAARR
5dzz.1.A    --------------------------------------------------------------------------------

Target      AQAEQAALRQKQAADAEMEKHKKFAEQTLRQKAQVEQELTALRLKLEETDHQKSILDQELQRLKAEVTEAARQRSQVEEE
5dzz.1.A    --------------------------------------------------------------------------------

Target      LFSVRVQMEELGKLKARIEAENRALILRDKDNTQRLLQEEAEKMKQVAEEAARLSVAAQEAARLRQLAEEDLAQQRALAE
5dzz.1.A    --------------------------------------------------------------------------------
```

```
Target     KMLKEKMQAVQEATRLKAEAELLQQQKELAQEQARRLQEDKEQMAQQLAQETQGFQRTLETERQRQLEMSAEAERLRLRV
5dzz.1.A   --------------------------------------------------------------------------------

Target     AEMSRAQARAEEDAQRFRKQAEEIGAKLHRTELATQEKVTLVQTLETQRQQSDRDADRLREAIAELEREKDKLKKEAELL
5dzz.1.A   --------------------------------------------------------------------------------

Target     QLKSEEMQTVQQEQLLQETQALQQSFLSEKDSLLQRERFIEEEKAKLERLFQDEVAKAQKLREEQQRQQQQMQQEKQQLL
5dzz.1.A   --------------------------------------------------------------------------------

Target     ASMEEARRRQHEAEEGVRRKQEELQLLEQQRQQQEQLLAEENRRLRERLEHLEEEHRAALAHSEEITAAQAAATRALPNG
5dzz.1.A   --------------------------------------------------------------------------------

Target     QDAADGPAAEPEHAFEGLRQKVPAQQLQEAGILSTEELQRLAQGHTTVAELTQREDVRRYLQGHSSIAGLLLKPANEKLT
5dzz.1.A   --------------------------------------------------------------------------------

Target     IYAALRRQLLSPGTALILLEAQAASGFLLDPVRNRRLTVTEAVKEGVVGPELHHKLLSAERAVTGYKDPYTGEKISLFQA
5dzz.1.A   --------------------------------------------------------------------------------

Target     MKKDLIVREHGIRLLEAQIATGGVIDPVHSHRVPVDVAYHRGYFDEEMNRVLQDPSDDTKGFFDPNTHENLTYLQLLERC
5dzz.1.A   --------------------------------------------------------------------------------

Target     VEDPETGLRLLPLTDQAAKGGELVYTDSEARDVFEKATVSAPFGKFQGKTVTIWELINSEYFTAEQRRDLLRQFRTGKVT
5dzz.1.A   --------------------------------------------------------------------------------

Target     VEKIIKIVITVIEEHEQKGQLCFQGLRALVPAAELLESGIIDWDLFRQLQLGERSVQEVAEVEGVRRALRGSGVIAGVWL
5dzz.1.A   --------------------------------------------------------------------------------

Target     EEARQKLSIYEALKKELLQPEAAVALLEAQAGTGHVIDPATSARLTVDEAVRAGLVGPELHEKLLSAEKAVTGYKDPYSG
5dzz.1.A   --------------------------------------------------------------------------------

Target     QSVSLFQALKKGLIPREQGLRLLDAQLSTGGTVDPSKSHRLPLDVACARGYLDEETSTALSAPRDDAKTYYDPRTWELAT
5dzz.1.A   --------------------------------------------------------------------------------

Target     YSQLQQQCRPDPLTGLSLLPLSEEAARARQQELYSEVQAREAFQKATVEVPVGSFQGRAVTIWELINSEYFTAEQRQELL
5dzz.1.A   --------------------------------------------------------------------------------

Target     RQFRTGKVTVEKIIKIVITIVEEVETTRRERLSFSGLRAPVPASELLAAGILSSSQFEQLKDGKTSVKDLSELSSLQTLL
5dzz.1.A   --------------------------------------------------------------------------------

Target     QGSGCLAGIYLEESKEKVTIYEAMRRGLLRPSTAILLLEAQAATGFLVDPVRNQRLYVHEAVKAGVVGPELHEKLLSAEK
5dzz.1.A   --------------------------------------------------------------------------------

Target     AVTGYKDPYSGSTISLFQAMKKGLVVREHGIRLLEAQIATGGIIDPVHSHRVPVDVACQRGYFDEEMNRVLEDPSDDTKG
5dzz.1.A   --------------------------------------------------------------------------------

Target     FFDPNTHENLTYRQLLERCVEDPETGLRLLPLKGPEKAEVVETTRVYTEEETRRAFEETQIDIPGGGSHGGSTMSLWEVM
5dzz.1.A   --------------------------------------------------------------------------------

Target     QSDLIPEEQRAQLMADFQAGRVTKERMIIIIIEIIEKTEIVRQQNLASYDYVRRRLTAEDLYEARVISRESYSLLREGTR
5dzz.1.A   --------------------------------------------------------------------------------

Target     SLREVLEAESAWRYLYGTGCVAGVYLPGSRQTLPIYQALKKGLLSAEVARLLLEAQAATGFLLDPVKGERLTVDEAVRKG
5dzz.1.A   --------------------------------------------------------------------------------

Target     LVGPELHDRLLSAERAVTGYRDPYTEQTISLFQAMKKDLIPAEEALRLLDAQLATGGIVDPRLGFHLPLEVAYQRGYLNK
5dzz.1.A   --------------------------------------------------------------------------------

Target     DTHDQLSEPSEVRSYVDPSTDERLSYTQLLRRCRRDETSGLFLLPLSEARKLTFRGLRKQITVEELVRSHVMDEATAQRL
5dzz.1.A   -----------------------------------------------DTSKLVFDGLRKKVTAMQLYECQLIDKTTLDKL

Target     QEGLTSIEEVSKNLQKFLEGTSSIAGVLVDATKERLSVYQAMKKGIIRPGTAFELLEAQAATGYVIDPIKGLKLTVEEAV
5dzz.1.A   LKGKKSVEEVASEIQPFLRGAGSIAGAS-ASPKEKYSLVEAKRKKLISPESTVMLLEAQAATGGIIDPHRNEKLTVDSAI

Target     RMGIVGPEFKDKLLSAERAVTGYKDPYSGKLISLFQAMKKGLILKDHGIRLLEAQIATGGIIDPEESHRLPVDVAYQRGL
5dzz.1.A   ARDLIDFDDRQQIYAAEKAITGFDDPFSGKTVSVSEAIKKNLIDRETGMRLLEAQIASGGVVDPVNSVFLPKDVALARGL

Target     FDEEMNEILTDPSDDTKGFFDPNTEENLTYLQLMERCVTDPQTGLRLLPLKEKKRERKTSSKSSVRKRRVVIVDPETGKE
5dzz.1.A   IDRDLYRSLNDPRDSQKNFVDPVTKKKVSYVQLKERCRIEPHTGLLLLSVQKRSM-----------------SFQG----

Target     MSVYEAYRKGLIDHQTYLELSEQECEWEEITISSSDGVVKSMIIDRRSGRQYDIDEAIAKSLIDRSALDQYRAGTLSITE
5dzz.1.A   -----------------------------------------------IRQPVTVTELVDSGILRPSTVNELESGQISYDE

Target     FADMLSGNAGGFRSRSSSVGSSSSYPISPAVSRTQLASWSDPTEETGPVAGILDTETLEKVSITEAMHRNLVDNITGQRL
5dzz.1.A   VGER----------------------------------IKDFLQGSSCIAGIYNETTKQKLGIYEAMKIGLVRPGTALEL
```

```
Target    LEAQACTGGIIDPNTGERFPVTDAVNKGLVDKIMVDRINLAQKAFCGFEDPRTKTKMSAAQALKKGWLYYEAGQRFLEVQ
5dzz.1.A  LEAQAATGFIVDPVSNLRLPVEEAYKRGLVGIEFKEKLLSAERAVTGYNDPETGNIISLFQAMNKELIEKGHGIRLLEAQ

Target    YLTGGLIEPDTPGRVPLDEALQRGTVDARTAQKLRDVSAYSKYLTCPKTKLKISYKDALDRSMVEEGTGLRLLEAAAQSS
5dzz.1.A  IATGGIIDPKESHRLPVDIAYKRGYFNEELSEILSDPSDDTKGFFDPNTEENLTYLQLKERCIKDEETGLCLLPLK----

Target    KGYYSPYSVSGSGSTTGSRSGSRTGSRAGSRRGSFDATGSGFSMTFSSSSYSSSGYGRRYASGPTSSLGGPESAAA
5dzz.1.A  ----------------------------------------------------------------------------
```

Model #13	File	Built with	Oligo-State	Ligands	GMQE	QMEANDisCo Global
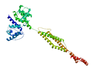	PDB	ProMod3 3.3.0	monomer (matching prediction)	None	0.00	0.62 ± 0.05

Template	Seq Identity	Oligo-state	QSQE	Found by	Method	Resolution	Seq Similarity	Range	Coverage	Description
6sl7.1.A	27.80	homo-dimer	0.00	HHblits	X-ray	3.30Å	0.35	63 - 480	0.09	Calponin homology domain protein putative

The template contained no ligands.

```
Target    MSGEDQEVRAVVEDGSNGGSGSPSPGDTLPWNLEKTQRSRRGGGGPGGNGSVLDPAERAVIRIADERDRVQKKTFTKWVN
6sl7.1.A  --------------------------------------------------------------LVAQWEKVQIKTFTKWVN

Target    KHLIKAQRHISDLYEDLRDGHNLISLLEVLSGDSLPR-EKGRMRFHKLQNVQIALDYLRHRQVKLVNIRNDDIADGNPKL
6sl7.1.A  MHLAKKGRKINDVTTDFKNGVELCALLEIIGETTIKCVTNPKMRIQMTENLDKALRFIQSRDVKLTGIGPTDIVDGNVKL

Target    TLGLIWTIILHFQISDIQVSGQSEDMTAKEKLLLWSQRMVEGYQGLRCDNFTSSWRDGRLFNAIIHRHKPMLIDMNKVYR
6sl7.1.A  TLGLVWTLILRFAISELSAE----GLSAKQGLLLWCQKKCEPYP-VKVENFSESFKDGKVFCALIHRHRPDLLDWETVGE

Target    QTNLENLDQAFSVAERDLGVTRLLDPEDVDV-PQPDEKSIITYVSSLYDAMPRVPDVQDGVK--------ANELQLRWQE
6sl7.1.A  -DDRANLEKAFDVAEKELGIPKLLDVDDIVNMPRPDERSVMTYVAALYKVFSSNDQVEKAGKRAGNFLDLLRATEGMVHD

Target    YRELVLLLLQWIRAHTAAFEERRFPSSFEEIEILWCQFLKFKETELPAKEADKNRSKGIYQSLEGAVQAG---QLKVPPG
6sl7.1.A  YEQRAQALKENIEAAINKMNGVEPSDEYHQVKEQINETKNYRKGDKRAFIKEQGDLATLFGQINSKLRGMKRPVYVAPEG

Target    YHPLDVEKEWGKLHVAILEREKQLRSEFERLERLQRIVSKLQMEAGLCEEQLNQADALLQSDVRLLAAGKAPQRAGEVER
6sl7.1.A  LDPKSLEGYIANISEAERALRSKLNTAMR--NCLIALRKAFADPANATDAKINEYRTFVTDETSEAPLEEQVATLKAKLE

Target    DLDKADGMIRLLFNDVQALKDGRHPQGEQMYRRVYRLHERLVAIRTEYNLRLRGTPRHPELEDSTLRYLQDLLAWVEENQ
6sl7.1.A  ELKQVEAQLPPIE-------------------------------------------------------------------

Target    RRVDSAEWGVDLPSVEAQLGSHRGLHQSVEEFRAKIERARTDEGQLSPATRGAYRDCLGRLDLQYAKLLNSSKARLRSLE
6sl7.1.A  --------------------------------------------------------------------------------

Target    SLHGFVAAATKELMWLSEKEEEEVGFDWSERNSNMAAKKEAYSALMRELELKEKKIKEIQSTGDRLLREDHPARPTVESF
6sl7.1.A  --------------------------------------------------------------------------------

Target    QAALQTQWSWMLQLCCCIEAHLKENTAYFQFFSDVREAEEQLRKLQETLHRKYTCDRSITVTRLEDLLQDAQDEKDQLNE
6sl7.1.A  --------------------------------------------------------------------------------

Target    YRGHLSGLAKRAKAIVQLTPRNPTQPTRGRVPLLAVCDYKQVEATVHKGDECQMLGPAQPFHWKVLSSSGSEAAVPSVCF
6sl7.1.A  --------------------------------------------------------------------------------

Target    LVPPPNQEALEAVARLEAQHQALVTLWHQLHTDMKSLLAWQSLSRDVQLIRSWSLVTFRTLKPEEQRQALRSLELHYQAF
6sl7.1.A  --------------------------------------------------------------------------------

Target    LRDSQDAGGFGPEDRLQAEREYGSCSRHYQQLLQSLEQGEQEESRCQRCISELKDIRLQLEACETRTVHRLRLPLDKEPA
6sl7.1.A  --------------------------------------------------------------------------------

Target    RECAQRIAEQQKAQAEVEGLGKGVARLSAEAEKVLALPEPSPAAPTLRSELELTLGKLEQVRSLSAIYLEKLKTISLVIR
6sl7.1.A  --------------------------------------------------------------------------------

Target    STQGAEEALRAHEEQLKEAQAVPAALPELEATKAAMKKLRAQAEAQQPVFDALRDELRGAQEVGERLQQRHGERDVEVER
6sl7.1.A  --------------------------------------------------------------------------------
```

```
Target     WRERVTQLLERWQAVLAQTDVRQRELEQLGRQLRYYRESADPLGAWLQDARRRQEQIQAVPLADSQAVREQLRQEKALLE
6sl7.1.A   --------------------------------------------------------------------------------

Target     EIERHAEKVEECQRFAKQYINAIKDYELQLVTYKAQLEPVASPAKKPKVQSGSESVIQEYVDLRTRYSELSTLTSQYIRF
6sl7.1.A   --------------------------------------------------------------------------------

Target     ISETLRRMEEEERLAEQQRAEERERLAEVEAALEKQRQLAEAHAQAKAQAEREAQELQRRMQEEVARREEVAVDAQQQKR
6sl7.1.A   --------------------------------------------------------------------------------

Target     SIQEELQQLRQSSEAEIQAKARQVEAAERSRLRIEEEIRVVRLQLETTERQRGGAEGELQALRARAEEAEAQKRQAQEEA
6sl7.1.A   --------------------------------------------------------------------------------

Target     ERLRRQVQEETQRKRQAEAELGLRVKAEAEAAREKQRALQALEELRLQAEEAERRLRQAEAERARQVQVALETAQRSAQA
6sl7.1.A   --------------------------------------------------------------------------------

Target     ELQSKHASFAEKTAQLERTLEEEHVTVVQLREEATRREQQQAEAERAREEAERELERWQLKANEALRLRLQAEEVAQQKS
6sl7.1.A   --------------------------------------------------------------------------------

Target     LAQAEAEKQKEAAEREARRRGKAEEQAVRQRELAEQELERQRQLAEGTAQQRLAAEQELIRLRAETEQGEQQRQLLEEEL
6sl7.1.A   --------------------------------------------------------------------------------

Target     ARLQSEAAAATQKRQELEAELAKVRAEMEVLLASKARAEEESRSSSEKSKQRLEAEAGRFRELAEEAARLRALAEEAKRQ
6sl7.1.A   --------------------------------------------------------------------------------

Target     RQLAEEDAARQRAEAERVLSEKLAAISEATRLKTEAEIALKEKEAENERLRRLAEDEAFQRRRLEEQAAQHKADIEERLA
6sl7.1.A   --------------------------------------------------------------------------------

Target     QLRKASESELERQKGLVEDTLRQRRQVEEEILALKASFEKAAAGKAELELELGRIRGNAEDTLRSKEQAEQEAARQRQLA
6sl7.1.A   --------------------------------------------------------------------------------

Target     AEEERRRREAEERVQKSLAAEEEAARQRKAALEEVERLKAKVEEARRLRERAEHESVRQLQLAQEAAQKRLQAEEKAHAF
6sl7.1.A   --------------------------------------------------------------------------------

Target     AVQQKEQELQQTLQQEQSVLEKLRSEAEAARRAAEEAEEARERAEREAAQSRQRVEEAERLKQAAEEQAQAQAQAQAAAE
6sl7.1.A   --------------------------------------------------------------------------------

Target     KLRKEAEQEAARRAQAEQAALRQKQAADAEMEKHKKFAEQTLRQKAQVEQELTALRLKLEETDHQKSILDQELQRLKAEV
6sl7.1.A   --------------------------------------------------------------------------------

Target     TEAARQRSQVEEELFSVRVQMEELGKLKARIEAENRALILRDKDNTQRLLQEEAEKMKQVAEEAARLSVAAQEAARLRQL
6sl7.1.A   --------------------------------------------------------------------------------

Target     AEEDLAQQRALAEKMLKEKMQAVQEATRLKAEAELLQQQKELAQEQARRLQEDKEQMAQQLAQETQGFQRTLETERQRQL
6sl7.1.A   --------------------------------------------------------------------------------

Target     EMSAEAERLRLRVAEMSRAQARAEEDAQRFRKQAEEIGAKLHRTELATQEKVTLVQTLETQRQQSDRDADRLREAIAELE
6sl7.1.A   --------------------------------------------------------------------------------

Target     REKDKLKKEAELLQLKSEEMQTVQQEQLLQETQALQQSFLSEKDSLLQRERFIEEEKAKLERLFQDEVAKAQKLREEQQR
6sl7.1.A   --------------------------------------------------------------------------------

Target     QQQQMQQEKQQLLASMEEARRRQHEAEEGVRRKQEELQLLEQQRQQQEQLLAEENRRLRERLEHLEEEHRAALAHSEEIT
6sl7.1.A   --------------------------------------------------------------------------------

Target     AAQAAATRALPNGQDAADGPAAEPEHAFEGLRQKVPAQQLQEAGILSTEELQRLAQGHTTVAELTQREDVRRYLQGHSSI
6sl7.1.A   --------------------------------------------------------------------------------

Target     AGLLLKPANEKLTIYAALRRQLLSPGTALILLEAQAASGFLLDPVRNRRLTVTEAVKEGVVGPELHHKLLSAERAVTGYK
6sl7.1.A   --------------------------------------------------------------------------------

Target     DPYTGEKISLFQAMKKDLIVREHGIRLLEAQIATGGVIDPVHSHRVPVDVAYHRGYFDEEMNRVLQDPSDDTKGFFDPNT
6sl7.1.A   --------------------------------------------------------------------------------

Target     HENLTYLQLLERCVEDPETGLRLLPLTDQAAKGGELVYTDSEARDVFEKATVSAPFGKFQGKTVTIWELINSEYFTAEQR
6sl7.1.A   --------------------------------------------------------------------------------

Target     RDLLRQFRTGKVTVEKIIKIVITVIEEHEQKGGQLCFQGLRALVPAAELLESGIIDWDLFRQLQLGERSVQEVAEVEGVRR
6sl7.1.A   --------------------------------------------------------------------------------

Target     ALRGSGVIAGVWLEEARQKLSIYEALKKELLQPEAAVALLEAQAGTGHVIDPATSARLTVDEAVRAGLVGPELHEKLLSA
6sl7.1.A   --------------------------------------------------------------------------------

Target     EKAVTGYKDPYSGQSVSLFQALKKGLIPREQGLRLLDAQLSTGGTVDPSKSHRLPLDVACARGYLDEETSTALSAPRDDA
```

```
6sl7.1.A   --------------------------------------------------------------------------------

Target     KTYYDPRTWELATYSQLQQQCRPDPLTGLSLLPLSEEAARARQQELYSEVQAREAFQKATVEVPVGSFQGRAVTIWELIN
6sl7.1.A   --------------------------------------------------------------------------------

Target     SEYFTAEQRQELLRQFRTGKVTVEKIIKIVITIVEEVETTRRERLSFSGLRAPVPASELLAAGILSSSQFEQLKDGKTSV
6sl7.1.A   --------------------------------------------------------------------------------

Target     KDLSELSSLQTLLQGSGCLAGIYLEESKEKVTIYEAMRRGLLRPSTAILLLEAQAATGFLVDPVRNQRLYVHEAVKAGVV
6sl7.1.A   --------------------------------------------------------------------------------

Target     GPELHEKLLSAEKAVTGYKDPYSGSTISLFQAMKKGLVVREHGIRLLEAQIATGGIIDPVHSHRVPVDVACQRGYFDEEM
6sl7.1.A   --------------------------------------------------------------------------------

Target     NRVLEDPSDDTKGFFDPNTHENLTYRQLLERCVEDPETGLRLLPLKGPEKAEVVETTRVYTEEETRRAFEETQIDIPGGG
6sl7.1.A   --------------------------------------------------------------------------------

Target     SHGGSTMSLWEVMQSDLIPEEQRAQLMADFQAGRVTKERMIIIIIEIIEKTEIVRQQNLASYDYVRRRLTAEDLYEARVI
6sl7.1.A   --------------------------------------------------------------------------------

Target     SRESYSLLREGTRSLREVLEAESAWRYLYGTGCVAGVYLPGSRQTLPIYQALKKGLLSAEVARLLLEAQAATGFLLDPVK
6sl7.1.A   --------------------------------------------------------------------------------

Target     GERLTVDEAVRKGLVGPELHDRLLSAERAVTGYRDPYTEQTISLFQAMKKDLIPAEEALRLLDAQLATGGIVDPRLGFHL
6sl7.1.A   --------------------------------------------------------------------------------

Target     PLEVAYQRGYLNKDTHDQLSEPSEVRSYVDPSTDERLSYTQLLRRCRRDETSGLFLLPLSEARKLTFRGLRKQITVEELV
6sl7.1.A   --------------------------------------------------------------------------------

Target     RSHVMDEATAQRLQEGLTSIEEVSKNLQKFLEGTSSIAGVLVDATKERLSVYQAMKKGIIRPGTAFELLEAQAATGYVID
6sl7.1.A   --------------------------------------------------------------------------------

Target     PIKGLKLTVEEAVRMGIVGPEFKDKLLSAERAVTGYKDPYSGKLISLFQAMKKGLILKDHGIRLLEAQIATGGIIDPEES
6sl7.1.A   --------------------------------------------------------------------------------

Target     HRLPVDVAYQRGLFDEEMNEILTDPSDDTKGFFDPNTEENLTYLQLMERCVTDPQTGLRLLPLKEKKRERKTSSKSSVRK
6sl7.1.A   --------------------------------------------------------------------------------

Target     RRVVIVDPETGKEMSVYEAYRKGLIDHQTYLELSEQECEWEEITISSSDGVVKSMIIDRRSGRQYDIDEAIAKSLIDRSA
6sl7.1.A   --------------------------------------------------------------------------------

Target     LDQYRAGTLSITEFADMLSGNAGGFRSRSSSVGSSSSYPISPAVSRTQLASWSDPTEETGPVAGILDTETLEKVSITEAM
6sl7.1.A   --------------------------------------------------------------------------------

Target     HRNLVDNITGQRLLEAQACTGGIIDPNTGERFPVTDAVNKGLVDKIMVDRINLAQKAFCGFEDPRTKTKMSAAQALKKGW
6sl7.1.A   --------------------------------------------------------------------------------

Target     LYYEAGQRFLEVQYLTGGLIEPDTPGRVPLDEALQRGTVDARTAQKLRDVSAYSKYLTCPKTKLKISYKDALDRSMVEEG
6sl7.1.A   --------------------------------------------------------------------------------

Target     TGLRLLEAAAQSSKGYYSPYSVSGSGSTTGSRSGSRTGSRAGSRRGSFDATGSGFSMTFSSSSYSSSGYGRRYASGPTSS
6sl7.1.A   --------------------------------------------------------------------------------

Target     LGGPESAAA
6sl7.1.A   ---------
```

Model #19	File	Built with	Oligo-State	Ligands	GMQE	QMEANDisCo Global
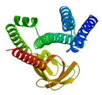	PDB	ProMod3 3.3.0	monomer	None	0.00	0.72 ± 0.05

Template	Seq Identity	Oligo-state	QSQE	Found by	Method	Resolution	Seq Similarity	Range	Coverage	Description
3pe0.1.A	88.53	monomer	0.00	HHblits	X-ray	2.95Å	0.58	626 - 899	0.06	Plectin

Excluded ligands

Ligand Name.Number	Reason for Exclusion	Description
CA.1	Not in contact with model.	CALCIUM ION

```
Target     MSGEDQEVRAVVEDGSNGGSGSPSPGDTLPWNLEKTQRSRRGGGGPGGNGSVLDPAERAVIRIADERDRVQKKTFTKWVN
3pe0.1.A   --------------------------------------------------------------------------------

Target     KHLIKAQRHISDLYEDLRDGHNLISLLEVLSGDSLPREKGRMRFHKLQNVQIALDYLRHRQVKLVNIRNDDIADGNPKLT
3pe0.1.A   --------------------------------------------------------------------------------

Target     LGLIWTIILHFQISDIQVSGQSEDMTAKEKLLLWSQRMVEGYQGLRCDNFTSSWRDGRLFNAIIHRHKPMLIDMNKVYRQ
3pe0.1.A   --------------------------------------------------------------------------------

Target     TNLENLDQAFSVAERDLGVTRLLDPEDVDVPQPDEKSIITYVSSLYDAMPRVPDVQDGVKANELQLRWQEYRELVLLLLQ
3pe0.1.A   --------------------------------------------------------------------------------

Target     WIRAHTAAFEERRFPSSFEEIEILWCQFLKFKETELPAKEADKNRSKGIYQSLEGAVQAGQLKVPPGYHPLDVEKEWGKL
3pe0.1.A   --------------------------------------------------------------------------------

Target     HVAILEREKQLRSEFERLERLQRIVSKLQMEAGLCEEQLNQADALLQSDVRLLAAGKAPQRAGEVERDLDKADGMIRLLF
3pe0.1.A   --------------------------------------------------------------------------------

Target     NDVQALKDGRHPQGEQMYRRVYRLHERLVAIRTEYNLRLRGTPRHPELEDSTLRYLQDLLAWVEENQRRVDSAEWGVDLP
3pe0.1.A   --------------------------------------------------------------------------------

Target     SVEAQLGSHRGLHQSVEEFRAKIERARTDEGQLSPATRGAYRDCLGRLDLQYAKLLNSSKARLRSLESLHGFVAAATKEL
3pe0.1.A   ---------------------------------------------------------------RSLESLHSFVAAATKEL

Target     MWLSEKEEEEVGFDWSERNSNMAAKKEAYSALMRELELKEKKIKEIQSTGDRLLREDHPARPTVESFQAALQTQWSWMLQ
3pe0.1.A   MWLNEKEEEEVGFDWSDRNTNMTAKKESYSALMRELELKEKKIKELQNAGDRLLREDHPARPTVESFQAALQTQWSWMLQ

Target     LCCCIEAHLKENTAYFQFFSDVREAEEQLRKLQETLHRKYTCDRSITVTRLEDLLQDAQDEKDQLNEYRGHLSGLAKRAK
3pe0.1.A   LCCCIEAHLKENAAYFQFFSDVREAEGQLQKLQEALRRKYSCDRSATVTRLEDLLQDAQDEKEQLNEYKGHLSGLAKRAK

Target     AIVQLTPRNPTQPTRGRVPLLAVCDYKQVEATVHKGDECQMLGPAQPFHWKVLSSSGSEAAVPSVCFLVPPPNQEALEAV
3pe0.1.A   AVVQLKPRHPAHPMRGRLPLLAVCDYKQVEVTVHKGDECQLVGPAQPSHWKVLSSSGSEAAVPSVCFLVPPPNQEAQEAV

Target     ARLEAQHQALVTLWHQLHTDMKSLLAWQSLSRDVQLIRSWSLVTFRTLKPEEQRQALRSLELHYQAFLRDSQDAGGFGPE
3pe0.1.A   TRLEAQHQALVTLWHQLHVDMK----------------------------------------------------------

Target     DRLQAEREYGSCSRHYQQLLQSLEQGEQEESRCQRCISELKDIRLQLEACETRTVHRLRLPLDKEPARECAQRIAEQQKA
3pe0.1.A   --------------------------------------------------------------------------------

Target     QAEVEGLGKGVARLSAEAEKVLALPEPSPAAPTLRSELELTLGKLEQVRSLSAIYLEKLKTISLVIRSTQGAEEALRAHE
3pe0.1.A   --------------------------------------------------------------------------------

Target     EQLKEAQAVPAALPELEATKAAMKKLRAQAEAQQPVFDALRDELRGAQEVGERLQQRHGERDVEVERWRERVTQLLERWQ
3pe0.1.A   --------------------------------------------------------------------------------

Target     AVLAQTDVRQRELEQLGRQLRYYRESADPLGAWLQDARRRQEQIQAVPLADSQAVREQLRQEKALLEEIERHAEKVEECQ
3pe0.1.A   --------------------------------------------------------------------------------

Target     RFAKQYINAIKDYELQLVTYKAQLEPVASPAKKPKVQSGSESVIQEYVDLRTRYSELSTLTSQYIRFISETLRRMEEEER
3pe0.1.A   --------------------------------------------------------------------------------

Target     LAEQQRAEERERLAEVEAALEKQRQLAEAHAQAKAQAEREAQELQRRMQEEVARREEVAVDAQQQKRSIQEELQQLRQSS
3pe0.1.A   --------------------------------------------------------------------------------

Target     EAEIQAKARQVEAAERSRLRIEEEIRVVRLQLETTERQRGGAEGELQALRARAEEAEAQKRQAQEEAERLRRQVQEETQR
3pe0.1.A   --------------------------------------------------------------------------------

Target     KRQAEAELGLRVKAEAEAAREKQRALQALEELRLQAEEAERRLRQAEAERARQVQVALETAQRSAQAELQSKHASFAEKT
3pe0.1.A   --------------------------------------------------------------------------------

Target     AQLERTLEEEHVTVVQLREEATRREQQQAEAERAREEAERELERWQLKANEALRLRLQAEEVAQQKSLAQAEAEKQKEAA
3pe0.1.A   --------------------------------------------------------------------------------

Target     EREARRRGKAEEQAVRQRELAEQELERQRQLAEGTAQQRLAAEQELIRLRAETEQGEQQRQLLEEELARLQSEAAAATQK
3pe0.1.A   --------------------------------------------------------------------------------

Target     RQELEAELAKVRAEMEVLLASKARAEEESRSSSEKSKQRLEAEAGRFRELAEEAARLRALAEEAKRQRQLAEEDAARQRA
3pe0.1.A   --------------------------------------------------------------------------------
```

```
Target     EAERVLSEKLAAISEATRLKTEAEIALKEKEAENERLRRLAEDEAFQRRRLEEQAAQHKADIEERLAQLRKASESELERQ
3pe0.1.A   --------------------------------------------------------------------------------

Target     KGLVEDTLRQRRQVEEEILALKASFEKAAAGKAELELELGRIRGNAEDTLRSKEQAEQEAARQRQLAAEEERRRREAEER
3pe0.1.A   --------------------------------------------------------------------------------

Target     VQKSLAAEEEAARQRKAALEEVERLKAKVEEARRLRERAEHESVRQLQLAQEAAQKRLQAEEKAHAFAVQQKEQELQQTL
3pe0.1.A   --------------------------------------------------------------------------------

Target     QQEQSVLEKLRSEAEAARRAAEEAEEARERAEREAAQSRQRVEEAERLKQAAEEQAQAQAQAQAAAEKLRKEAEQEAARR
3pe0.1.A   --------------------------------------------------------------------------------

Target     AQAEQAALRQKQAADAEMEKHKKFAEQTLRQKAQVEQELTALRLKLEETDHQKSILDQELQRLKAEVTEAARQRSQVEEE
3pe0.1.A   --------------------------------------------------------------------------------

Target     LFSVRVQMEELGKLKARIEAENRALILRDKDNTQRLLQEEAEKMKQVAEEAARLSVAAQEAARLRQLAEEDLAQQRALAE
3pe0.1.A   --------------------------------------------------------------------------------

Target     KMLKEKMQAVQEATRLKAEAELLQQQKELAQEQARRLQEDKEQMAQQLAQETQGFQRTLETERQRQLEMSAEAERLRLRV
3pe0.1.A   --------------------------------------------------------------------------------

Target     AEMSRAQARAEEDAQRFRKQAEEIGAKLHRTELATQEKVTLVQTLETQRQQSDRDADRLREAIAELEREKDKLKKEAELL
3pe0.1.A   --------------------------------------------------------------------------------

Target     QLKSEEMQTVQQEQLLQETQALQQSFLSEKDSLLQRERFIEEEKAKLERLFQDEVAKAQKLREEQQRQQQMQQEKQQLL
3pe0.1.A   --------------------------------------------------------------------------------

Target     ASMEEARRRQHEAEEGVRRKQEELQLLEQQRQQQEQLLAEENRRLRERLEHLEEEHRAALAHSEEITAAQAAATRALPNG
3pe0.1.A   --------------------------------------------------------------------------------

Target     QDAADGPAAEPEHAFEGLRQKVPAQQLQEAGILSTEELQRLAQGHTTVAELTQREDVRRYLQGHSSIAGLLLKPANEKLT
3pe0.1.A   --------------------------------------------------------------------------------

Target     IYAALRRQLLSPGTALILLEAQAASGFLLDPVRNRRLTVTEAVKEGVVGPELHHKLLSAERAVTGYKDPYTGEKISLFQA
3pe0.1.A   --------------------------------------------------------------------------------

Target     MKKDLIVREHGIRLLEAQIATGGVIDPVHSHRVPVDVAYHRGYFDEEMNRVLQDPSDDTKGFFDPNTHENLTYLQLLERC
3pe0.1.A   --------------------------------------------------------------------------------

Target     VEDPETGLRLLPLTDQAAKGGELVYTDSEARDVFEKATVSAPFGKFQGKTVTIWELINSEYFTAEQRRDLLRQFRTGKVT
3pe0.1.A   --------------------------------------------------------------------------------

Target     VEKIIKIVITVIEEHEQKGQLCFQGLRALVPAAELLESGIIDWDLFRQLQLGERSVQEVAEVEGVRRALRGSGVIAGVWL
3pe0.1.A   --------------------------------------------------------------------------------

Target     EEARQKLSIYEALKKELLQPEAAVALLEAQAGTGHVIDPATSARLTVDEAVRAGLVGPELHEKLLSAEKAVTGYKDPYSG
3pe0.1.A   --------------------------------------------------------------------------------

Target     QSVSLFQALKKGLIPREQGLRLLDAQLSTGGTVDPSKSHRLPLDVACARGYLDEETSTALSAPRDDAKTYYDPRTWELAT
3pe0.1.A   --------------------------------------------------------------------------------

Target     YSQLQQQCRPDPLTGLSLLPLSEEAARARQQELYSEVQAREAFQKATVEVPVGSFQGRAVTIWELINSEYFTAEQRQELL
3pe0.1.A   --------------------------------------------------------------------------------

Target     RQFRTGKVTVEKIIKIVITIVEEVETTRRERLSFSGLRAPVPASELLAAGILSSSQFEQLKDGKTSVKDLSELSSLQTLL
3pe0.1.A   --------------------------------------------------------------------------------

Target     QGSGCLAGIYLEESKEKVTIYEAMRRGLLRPSTAILLLEAQAATGFLVDPVRNQRLYVHEAVKAGVVGPELHEKLLSAEK
3pe0.1.A   --------------------------------------------------------------------------------

Target     AVTGYKDPYSGSTISLFQAMKKGLVVREHGIRLLEAQIATGGIIDPVHSHRVPVDVACQRGYFDEEMNRVLEDPSDDTKG
3pe0.1.A   --------------------------------------------------------------------------------

Target     FFDPNTHENLTYRQLLERCVEDPETGLRLLPLKGPEKAEVVETTRVYTEEETRRAFEETQIDIPGGGSHGGSTMSLWEVM
3pe0.1.A   --------------------------------------------------------------------------------

Target     QSDLIPEEQRAQLMADFQAGRVTKERMIIIIIEIIEKTEIVRQQNLASYDYVRRRLTAEDLYEARVISRESYSLLREGTR
3pe0.1.A   --------------------------------------------------------------------------------

Target     SLREVLEAESAWRYLYGTGCVAGVYLPGSRQTLPIYQALKKGLLSAEVARLLLEAQAATGFLLDPVKGERLTVDEAVRKG
3pe0.1.A   --------------------------------------------------------------------------------

Target     LVGPELHDRLLSAERAVTGYRDPYTEQTISLFQAMKKDLIPAEEALRLLDAQLATGGIVDPRLGFHLPLEVAYQRGYLNK
3pe0.1.A   --------------------------------------------------------------------------------
```

```
Target     DTHDQLSEPSEVRSYVDPSTDERLSYTQLLRRCRRDETSGLFLLPLSEARKLTFRGLRKQITVEELVRSHVMDEATAQRL
3pe0.1.A   --------------------------------------------------------------------------------

Target     QEGLTSIEEVSKNLQKFLEGTSSIAGVLVDATKERLSVYQAMKKGIIRPGTAFELLEAQAATGYVIDPIKGLKLTVEEAV
3pe0.1.A   --------------------------------------------------------------------------------

Target     RMGIVGPEFKDKLLSAERAVTGYKDPYSGKLISLFQAMKKGLILKDHGIRLLEAQIATGGIIDPEESHRLPVDVAYQRGL
3pe0.1.A   --------------------------------------------------------------------------------

Target     FDEEMNEILTDPSDDTKGFFDPNTEENLTYLQLMERCVTDPQTGLRLLPLKEKKRERKTSSKSSVRKRRVVIVDPETGKE
3pe0.1.A   --------------------------------------------------------------------------------

Target     MSVYEAYRKGLIDHQTYLELSEQECEWEEITISSSDGVVKSMIIDRRSGRQYDIDEAIAKSLIDRSALDQYRAGTLSITE
3pe0.1.A   --------------------------------------------------------------------------------

Target     FADMLSGNAGGFRSRSSSVGSSSSYPISPAVSRTQLASWSDPTEETGPVAGILDTETLEKVSITEAMHRNLVDNITGQRL
3pe0.1.A   --------------------------------------------------------------------------------

Target     LEAQACTGGIIDPNTGERFPVTDAVNKGLVDKIMVDRINLAQKAFCGFEDPRTKTKMSAAQALKKGWLYYEAGQRFLEVQ
3pe0.1.A   --------------------------------------------------------------------------------

Target     YLTGGLIEPDTPGRVPLDEALQRGTVDARTAQKLRDVSAYSKYLTCPKTKLKISYKDALDRSMVEEGTGLRLLEAAAQSS
3pe0.1.A   --------------------------------------------------------------------------------

Target     KGYYSPYSVSGSGSTTGSRSGSRTGSRAGSRRGSFDATGSGFSMTFSSSSYSSSGYGRRYASGPTSSLGGPESAAA
3pe0.1.A   ----------------------------------------------------------------------------
```

Model #09	File	Built with	Oligo-State	Ligands	GMQE	QMEANDisCo Global
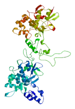	PDB	ProMod3 3.3.0	monomer	None	0.00	0.60 ± 0.05

Template	Seq Identity	Oligo-state	QSQE	Found by	Method	Resolution	Seq Similarity	Range	Coverage	Description
5dzz.1.A	48.75	monomer	0.00	BLAST	X-ray	2.60Å	0.43	2654 - 3223	0.11	Desmoplakin

The template contained no ligands.

```
Target     MSGEDQEVRAVVEDGSNGGSGSPSPGDTLPWNLEKTQRSRRGGGGPGGNGSVLDPAERAVIRIADERDRVQKKTFTKWVN
5dzz.1.A   --------------------------------------------------------------------------------

Target     KHLIKAQRHISDLYEDLRDGHNLISLLEVLSGDSLPREKGRMRFHKLQNVQIALDYLRHRQVKLVNIRNDDIADGNPKLT
5dzz.1.A   --------------------------------------------------------------------------------

Target     LGLIWTIILHFQISDIQVSGQSEDMTAKEKLLLWSQRMVEGYQGLRCDNFTSSWRDGRLFNAIIHRHKPMLIDMNKVYRQ
5dzz.1.A   --------------------------------------------------------------------------------

Target     TNLENLDQAFSVAERDLGVTRLLDPEDVDVPQPDEKSIITYVSSLYDAMPRVPDVQDGVKANELQLRWQEYRELVLLLLQ
5dzz.1.A   --------------------------------------------------------------------------------

Target     WIRAHTAAFEERRFPSSFEEIEILWCQFLKFKETELPAKEADKNRSKGIYQSLEGAVQAGQLKVPPGYHPLDVEKEWGKL
5dzz.1.A   --------------------------------------------------------------------------------

Target     HVAILEREKQLRSEFERLERLQRIVSKLQMEAGLCEEQLNQADALLQSDVRLLAAGKAPQRAGEVERDLDKADGMIRLLF
5dzz.1.A   --------------------------------------------------------------------------------

Target     NDVQALKDGRHPQGEQMYRRVYRLHERLVAIRTEYNLRLRGTPRHPELEDSTLRYLQDLLAWVEENQRRVDSAEWGVDLP
5dzz.1.A   --------------------------------------------------------------------------------

Target     SVEAQLGSHRGLHQSVEEFRAKIERARTDEGQLSPATRGAYRDCLGRLDLQYAKLLNSSKARLRSLESLHGFVAAATKEL
5dzz.1.A   --------------------------------------------------------------------------------

Target     MWLSEKEEEEVGFDWSERNSNMAAKKEAYSALMRELELKEKKIKEIQSTGDRLLREDHPARPTVESFQAALQTQWSWMLQ
5dzz.1.A   --------------------------------------------------------------------------------
```

```
Target      LCCCIEAHLKENTAYFQFFSDVREAEEQLRKLQETLHRKYTCDRSITVTRLEDLLQDAQDEKDQLNEYRGHLSGLAKRAK
5dzz.1.A    --------------------------------------------------------------------------------

Target      AIVQLTPRNPTQPTRGRVPLLAVCDYKQVEATVHKGDECQMLGPAQPFHWKVLSSSGSEAAVPSVCFLVPPPNQEALEAV
5dzz.1.A    --------------------------------------------------------------------------------

Target      ARLEAQHQALVTLWHQLHTDMKSLLAWQSLSRDVQLIRSWSLVTFRTLKPEEQRQALRSLELHYQAFLRDSQDAGGFGPE
5dzz.1.A    --------------------------------------------------------------------------------

Target      DRLQAEREYGSCSRHYQQLLQSLEQGEQEESRCQRCISELKDIRLQLEACETRTVHRLRLPLDKEPARECAQRIAEQQKA
5dzz.1.A    --------------------------------------------------------------------------------

Target      QAEVEGLGKGVARLSAEAEKVLALPEPSPAAPTLRSELELTLGKLEQVRSLSAIYLEKLKTISLVIRSTQGAEEALRAHE
5dzz.1.A    --------------------------------------------------------------------------------

Target      EQLKEAQAVPAALPELEATKAAMKKLRAQAEAQQPVFDALRDELRGAQEVGERLQQRHGERDVEVERWRERVTQLLERWQ
5dzz.1.A    --------------------------------------------------------------------------------

Target      AVLAQTDVRQRELEQLGRQLRYYRESADPLGAWLQDARRRQEQIQAVPLADSQAVREQLRQEKALLEEIERHAEKVEECQ
5dzz.1.A    --------------------------------------------------------------------------------

Target      RFAKQYINAIKDYELQLVTYKAQLEPVASPAKKPKVQSGSESVIQEYVDLRTRYSELSTLTSQYIRFISETLRRMEEEER
5dzz.1.A    --------------------------------------------------------------------------------

Target      LAEQQRAEERERLAEVEAALEKQRQLAEAHAQAKAQAEREAQELQRRMQEEVARREEVAVDAQQQKRSIQEELQQLRQSS
5dzz.1.A    --------------------------------------------------------------------------------

Target      EAEIQAKARQVEAAERSRLRIEEEIRVVRLQLETTERQRGGAEGELQALRARAEEAEAQKRQAQEEAERLRRQVQEETQR
5dzz.1.A    --------------------------------------------------------------------------------

Target      KRQAEAELGLRVKAEAEAAREKQRALQALEELRLQAEEAERRLRQAEAERARQVQVALETAQRSAQAELQSKHASFAEKT
5dzz.1.A    --------------------------------------------------------------------------------

Target      AQLERTLEEEHVTVVQLREEATRREQQQAEAERAREEAERELERWQLKANEALRLRLQAEEVAQQKSLAQAEAEKQKEAA
5dzz.1.A    --------------------------------------------------------------------------------

Target      EREARRRGKAEEQAVRQRELAEQELERQRQLAEGTAQQRLAAEQELIRLRAETEQGEQQRQLLEEELARLQSEAAAATQK
5dzz.1.A    --------------------------------------------------------------------------------

Target      RQELEAELAKVRAEMEVLLASKARAEEESRSSSEKSKQRLEAEAGRFRELAEEAARLRALAEEAKRQRQLAEEDAARQRA
5dzz.1.A    --------------------------------------------------------------------------------

Target      EAERVLSEKLAAISEATRLKTEAEIALKEKEAENERLRRLAEDEAFQRRRLEEQAAQHKADIEERLAQLRKASESELERQ
5dzz.1.A    --------------------------------------------------------------------------------

Target      KGLVEDTLRQRRQVEEEILALKASFEKAAAGKAELELELGRIRGNAEDTLRSKEQAEQEAARQRQLAAEEERRRREAEER
5dzz.1.A    --------------------------------------------------------------------------------

Target      VQKSLAAEEEAARQRKAALEEVERLKAKVEEARRLRERAEHESVRQLQLAQEAAQKRLQAEEKAHAFAVQQKEQELQQTL
5dzz.1.A    --------------------------------------------------------------------------------

Target      QQEQSVLEKLRSEAEAARRAAEEAEEARERAEREAAQSRQRVEEAERLKQAAEEQAQAQAQAQAAAEKLRKEAEQEAARR
5dzz.1.A    --------------------------------------------------------------------------------

Target      AQAEQAALRQKQAADAEMEKHKKFAEQTLRQKAQVEQELTALRLKLEETDHQKSILDQELQRLKAEVTEAARQRSQVEEE
5dzz.1.A    --------------------------------------------------------------------------------

Target      LFSVRVQMEELGKLKARIEAENRALILRDKDNTQRLLQEEAEKMKQVAEEAARLSVAAQEAARLRQLAEEDLAQQRALAE
5dzz.1.A    --------------------------------------------------------------------------------

Target      KMLKEKMQAVQEATRLKAEAELLQQQKELAQEQARRLQEDKEQMAQQLAQETQGFQRTLETERQRQLEMSAEAERLRLRV
5dzz.1.A    --------------------------------------------------------------------------------

Target      AEMSRAQARAEEDAQRFRKQAEEIGAKLHRTELATQEKVTLVQTLETQRQQSDRDADRLREAIAELEREKDKLKKEAELL
5dzz.1.A    --------------------------------------------------------------------------------

Target      QLKSEEMQTVQQEQLLQETQALQQSFLSEKDSLLQRERFIEEEKAKLERLFQDEVAKAQKLREEQQRQQQMQQEKQQLL
5dzz.1.A    --------------------------------------------------------------------------------

Target      ASMEEARRRQHEAEEGVRRKQEELQLLEQQRQQQEQLLAEENRRLRERLEHLEEEHRAALAHSEEITAAQAAATRALPNG
5dzz.1.A    --------------------------------------------------------------------------------

Target      QDAADGPAAEPEHAFEGLRQKVPAQQLQEAGILSTEELQRLAQGHTTVAELTQREDVRRYLQGHSSIAGLLLKPANEKLT
```

```
5dzz.1.A   -------------VFDGLRKKVTAMQLYECQLIDKTTLDKLLKGKKSVEEVAS--EIQPFLRGAGSIAGASASP-KEKYS

Target     IYAALRRQLLSPGTALILLEAQAASGFLLDPVRNRRLTVTEAVKEGVVGPELHHKLLSAERAVTGYKDPYTGEKISLFQA
5dzz.1.A   LVEAKRKKLISPESTVMLLEAQAATGGIIDPHRNEKLTVDSAIARDLIDFDDRQQIYAAEKAITGFDDPFSGKTVSVSEA

Target     MKKDLIVREHGIRLLEAQIATGGVIDPVHSHRVPVDVAYHRGYFDEEMNRVLQDPSDDTKGFFDPNTHENLTYLQLLERC
5dzz.1.A   IKKNLIDRETGMRLLEAQIASGGVVDPVNSVFLPKDVALARGLIDRDLYRSLNDPRDSQKNFVDPVTKKKVSYVQLKERC

Target     VEDPETGLRLLPLTDQAAKGGELVYTDSEARDVFEKATVSAPFGKFQG--KTVTIWELINSEYFTAEQRRDLLRQFRTGK
5dzz.1.A   RIEPHTGLLLLSV---------------------QKRSMS-----FQGIRQPVTVTELVDSGILRPSTVNEL--------

Target     VTVEKIIKIVITVIEEHEQKGQLCFQGLRALVPAAELLESGIIDWDLFRQLQLGERSVQEVAEVEGVRRALRGSGVIAGV
5dzz.1.A   --------------------------------------ESGQISYD-----EVGER----------IKDFLQGSSCIAGI

Target     WLEEARQKLSIYEALKKELLQPEAAVALLEAQAGTGHVIDPATSARLTVDEAVRAGLVGPELHEKLLSAEKAVTGYKDPY
5dzz.1.A   YNETTKQKLGIYEAMKIGLVRPGTALELLEAQAATGFIVDPVSNLRLPVEEAYKRGLVGIEFKEKLLSAERAVTGYNDPE

Target     SGQSVSLFQALKKGLIPREQGLRLLDAQLSTGGTVDPSKSHRLPLDVACARGYLDEETSTALSAPRDDAKTYYDPRTWEL
5dzz.1.A   TGNIISLFQAMNKELIEKGHGIRLLEAQIATGGIIDPKESHRLPVDIAYKRGYFNEELSEILSDPSDDTKGFFDPNTEEN

Target     ATYSQLQQQCRPDPLTGLSLLPLSEEAARARQQELYSEVQAREAFQKATVEVPVGSFQGRAVTIWELINSEYFTAEQRQE
5dzz.1.A   LTYLQLKERCIKDEETGLCLLPLKE-------------------------------------------------------

Target     LLRQFRTGKVTVEKIIKIVITIVEEVETTRRERLSFSGLRAPVPASELLAAGILSSSQFEQLKDGKTSVKDLSELSSLQT
5dzz.1.A   --------------------------------------------------------------------------------

Target     LLQGSGCLAGIYLEESKEKVTIYEAMRRGLLRPSTAILLLEAQAATGFLVDPVRNQRLYVHEAVKAGVVGPELHEKLLSA
5dzz.1.A   --------------------------------------------------------------------------------

Target     EKAVTGYKDPYSGSTISLFQAMKKGLVVREHGIRLLEAQIATGGIIDPVHSHRVPVDVACQRGYFDEEMNRVLEDPSDDT
5dzz.1.A   --------------------------------------------------------------------------------

Target     KGFFDPNTHENLTYRQLLERCVEDPETGLRLLPLKGPEKAEVVETTRVYTEEETRRAFEETQIDIPGGGSHGGSTMSLWE
5dzz.1.A   --------------------------------------------------------------------------------

Target     VMQSDLIPEEQRAQLMADFQAGRVTKERMIIIIIEIIEKTEIVRQQNLASYDYVRRRLTAEDLYEARVISRESYSLLREG
5dzz.1.A   --------------------------------------------------------------------------------

Target     TRSLREVLEAESAWRYLYGTGCVAGVYLPGSRQTLPIYQALKKGLLSAEVARLLLEAQAATGFLLDPVKGERLTVDEAVR
5dzz.1.A   --------------------------------------------------------------------------------

Target     KGLVGPELHDRLLSAERAVTGYRDPYTEQTISLFQAMKKDLIPAEEALRLLDAQLATGGIVDPRLGFHLPLEVAYQRGYL
5dzz.1.A   --------------------------------------------------------------------------------

Target     NKDTHDQLSEPSEVRSYVDPSTDERLSYTQLLRRCRRDETSGLFLLPLSEARKLTFRGLRKQITVEELVRSHVMDEATAQ
5dzz.1.A   --------------------------------------------------------------------------------

Target     RLQEGLTSIEEVSKNLQKFLEGTSSIAGVLVDATKERLSVYQAMKKGIIRPGTAFELLEAQAATGYVIDPIKGLKLTVEE
5dzz.1.A   --------------------------------------------------------------------------------

Target     AVRMGIVGPEFKDKLLSAERAVTGYKDPYSGKLISLFQAMKKGLILKDHGIRLLEAQIATGGIIDPEESHRLPVDVAYQR
5dzz.1.A   --------------------------------------------------------------------------------

Target     GLFDEEMNEILTDPSDDTKGFFDPNTEENLTYLQLMERCVTDPQTGLRLLPLKEKKRERKTSSKSSVRKRRVVIVDPETG
5dzz.1.A   --------------------------------------------------------------------------------

Target     KEMSVYEAYRKGLIDHQTYLELSEQECEWEEITISSSDGVVKSMIIDRRSGRQYDIDEAIAKSLIDRSALDQYRAGTLSI
5dzz.1.A   --------------------------------------------------------------------------------

Target     TEFADMLSGNAGGFRSRSSSVGSSSSYPISPAVSRTQLASWSDPTEETGPVAGILDTETLEKVSITEAMHRNLVDNITGQ
5dzz.1.A   --------------------------------------------------------------------------------

Target     RLLEAQACTGGIIDPNTGERFPVTDAVNKGLVDKIMVDRINLAQKAFCGFEDPRTKTKMSAAQALKKGWLYYEAGQRFLE
5dzz.1.A   --------------------------------------------------------------------------------

Target     VQYLTGGLIEPDTPGRVPLDEALQRGTVDARTAQKLRDVSAYSKYLTCPKTKLKISYKDALDRSMVEEGTGLRLLEAAAQ
5dzz.1.A   --------------------------------------------------------------------------------

Target     SSKGYYSPYSVSGSGSTTGSRSGSRTGSRAGSRRGSFDATGSGFSMTFSSSSYSSSGYGRRYASGPTSSLGGPESAAA
5dzz.1.A   ------------------------------------------------------------------------------
```

Model #21	File	Built with	Oligo-State	Ligands	GMQE	QMEANDisCo Global
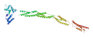	PDB	ProMod3 3.3.0	monomer (matching prediction)	None	0.00	0.59 ± 0.05

Template	Seq Identity	Oligo-state	QSQE	Found by	Method	Resolution	Seq Similarity	Range	Coverage	Description
4d1e.1.A	25.11	homo-dimer	0.00	HHblits	X-ray	3.50Å	0.33	65 - 738	0.15	ALPHA-ACTININ-2

The template contained no ligands.

```
Target     MSGEDQEVRAVVEDGSNGGSGSPSPGDTLPWNLEKTQRSRRGGGGPGGNGSVLDPAERAVIRIADERDRVQKKTFTKWVN
4d1e.1.A   ------------------------------------------------------------LLLDPAWEKQQRKTFTAWCN

Target     KHLIKAQRHISDLYEDLRDGHNLISLLEVLSGDSLPR-EKGRMRFHKLQNVQIALDYLRHRQVKLVNIRNDDIADGNPKL
4d1e.1.A   SHLRKAGTQIENIEEDFRNGLKLMLLLEVISGERLPKPDRGKMRFHKIANVNKALDYIASKGVKLVSIGAEEIVDGNVKM

Target     TLGLIWTIILHFQISDIQVSGQSEDMTAKEKLLLWSQRMVEGYQGLRCDNFTSSWRDGRLFNAIIHRHKPMLIDMNKVYR
4d1e.1.A   TLGMIWTIILRFAIQDISVE----ETSAKEGLLLWCQRKTAPYRNVNIQNFHTSWKDGLGLCALIHRHRPDLIDYSKLNK

Target     QTNLENLDQAFSVAERDLGVTRLLDPEDV-DVPQPDEKSIITYVSSLYDAMPRVPDVQDGVK--------ANELQLRWQE
4d1e.1.A   DDPIGNINLAMEIAEKHLDIPKMLDAEDIVNTPKPDERAIMTYVSCFYHAFAGAEQAETAANRICKVLAVNQENERLMEE

Target     YRELVLLLLQWIRAHTAAFEERRFPSSFEEIEILWCQFLKFKETELPAKEADKNRSKGIYQSLEGAVQAG---QLKVPPG
4d1e.1.A   YERLASELLEWIRRTIPWLENRTPAATMQAMQKKLEDFRDYRRKHKPPKVQEKCQLEINFNTLQTKLRISNRPAFMPSEG

Target     YHPLDVEKEWGKLHVAILEREKQLRSEFERLERLQRIVSKLQMEAGLCEEQLNQADALLQSDV----RLLAAGKAPQRAG
4d1e.1.A   KMVSDIAGAWQRLEQAEKGYEEWLLNEIRRLERLEHLAEKFRQKASTHETWAYGKEQILLQKDYESASLTEVRALLRKHE

Target     EVERDLDKADGMIRLLFNDVQALKDGRHPQGEQMYRRVYRLHERLVAIRTEYNL---RLRGTPRHP----ELEDSTLRYL
4d1e.1.A   AFESDLAAHQDRVEQIAAIAQELNELDYHDAVNVNDRCQKICDQWDRLGTLTQKRREALERMEKLLETIDQLHLEFAKRA

Target     QDLLAWVEENQRRVDSAEWGVDLPSVEAQLGSHRGLHQSVEEFRAKIERARTDEGQL-------S---PATRGAYRDCLG
4d1e.1.A   APFNNWMEGAMEDLQDMFIVHSIEEIQSLITAHEQFKATLPEADGERQSIMAIQNEVEKVIQSYNIRISSSNPYSTVTMD

Target     RLDLQYAKLLNSSKARLRSLES---L--------HGFVAAATKELMWLSEKEEEEVGFDWSERNSNMAAKKEAYSALMRE
4d1e.1.A   ELRTKWDKVKQLVPIRDQSLQEELARQHANERLRRQFAAQANAIGPWIQNKMEEIARSSIQ-ITGALEDQMNQLKQYEHN

Target     LELKEKKIKEIQSTGDRLLREDHPARPTVESFQAALQTQWSWMLQLCCCIEAHLKENTAYFQFFSDVREAEEQLRKLQET
4d1e.1.A   IINYKNNIDKLEGDHQLIQEALVFDNKHTNYTMEHIRVGWELLLTTIARTINEVETQILTRDA-----------------

Target     LHRKYTCDRSITVTRLEDLLQDAQDEKDQLNEYRGHLSGLAKRAKAIVQLTPRNPTQPTRGRVPLLAVCDYKQVEATVHK
4d1e.1.A   --------------------------------------------------------------------------------

Target     GDECQMLGPAQPFHWKVLSSSGSEAAVPSVCFLVPPPNQEALEAVARLEAQHQALVTLWHQLHTDMKSLLAWQSLSRDVQ
4d1e.1.A   --------------------------------------------------------------------------------

Target     LIRSWSLVTFRTLKPEEQRQALRSLELHYQAFLRDSQDAGGFGPEDRLQAEREYGSCSRHYQQLLQSLEQGEQEESRCQR
4d1e.1.A   --------------------------------------------------------------------------------

Target     CISELKDIRLQLEACETRTVHRLRLPLDKEPARECAQRIAEQQKAQAEVEGLGKGVARLSAEAEKVLALPEPSPAAPTLR
4d1e.1.A   --------------------------------------------------------------------------------

Target     SELELTLGKLEQVRSLSAIYLEKLKTISLVIRSTQGAEEALRAHEEQLKEAQAVPAALPELEATKAAMKKLRAQAEAQQP
4d1e.1.A   --------------------------------------------------------------------------------

Target     VFDALRDELRGAQEVGERLQQRHGERDVEVERWRERVTQLLERWQAVLAQTDVRQRELEQLGRQLRYYRESADPLGAWLQ
4d1e.1.A   --------------------------------------------------------------------------------

Target     DARRRQEQIQAVPLADSQAVREQLRQEKALLEEIERHAEKVEECQRFAKQYINAIKDYELQLVTYKAQLEPVASPAKKPK
4d1e.1.A   --------------------------------------------------------------------------------

Target     VQSGSESVIQEYVDLRTRYSELSTLTSQYIRFISETLRRMEEEERLAEQQRAEERERLAEVEAALEKQRQLAEAHAQAKA
4d1e.1.A   --------------------------------------------------------------------------------

Target     QAEREAQELQRRMQEEVARREEVAVDAQQQKRSIQEELQQLRQSSEAEIQAKARQVEAAERSRLRIEEEIRVVRLQLETT
4d1e.1.A   --------------------------------------------------------------------------------
```

```
Target      ERQRGGAEGELQALRARAEEAEAQKRQAQEEAERLRRQVQEETQRKRQAEAELGLRVKAEAEAAREKQRALQALEELRLQ
4d1e.1.A    --------------------------------------------------------------------------------

Target      AEEAERRLRQAEAERARQVQVALETAQRSAQAELQSKHASFAEKTAQLERTLEEEHVTVVQLREEATRREQQQAEAERAR
4d1e.1.A    --------------------------------------------------------------------------------

Target      EEAERELERWQLKANEALRLRLQAEEVAQQKSLAQAEAEKQKEAAEREARRRGKAEEQAVRQRELAEQELERQRQLAEGT
4d1e.1.A    --------------------------------------------------------------------------------

Target      AQQRLAAEQELIRLRAETEQGEQQRQLLEEELARLQSEAAAATQKRQELEAELAKVRAEMEVLLASKARAEEESRSSSEK
4d1e.1.A    --------------------------------------------------------------------------------

Target      SKQRLEAEAGRFRELAEEAARLRALAEEAKRQRQLAEEDAARQRAEAERVLSEKLAAISEATRLKTEAEIALKEKEAENE
4d1e.1.A    --------------------------------------------------------------------------------

Target      RLRRLAEDEAFQRRRLEEQAAQHKADIEERLAQLRKASESELERQKGLVEDTLRQRRQVEEEILALKASFEKAAAGKAEL
4d1e.1.A    --------------------------------------------------------------------------------

Target      ELELGRIRGNAEDTLRSKEQAEQEAARQRQLAAEEERRRREAEERVQKSLAAEEEAARQRKAALEEVERLKAKVEEARRL
4d1e.1.A    --------------------------------------------------------------------------------

Target      RERAEHESVRQLQLAQEAAQKRLQAEEKAHAFAVQQKEQELQQTLQQEQSVLEKLRSEAEAARRAAEEAEEARERAEREA
4d1e.1.A    --------------------------------------------------------------------------------

Target      AQSRQRVEEAERLKQAAEEQAQAQAQAQAAAEKLRKEAEQEAARRAQAEQAALRQKQAADAEMEKHKKFAEQTLRQKAQV
4d1e.1.A    --------------------------------------------------------------------------------

Target      EQELTALRLKLEETDHQKSILDQELQRLKAEVTEAARQRSQVEEELFSVRVQMEELGKLKARIEAENRALILRDKDNTQR
4d1e.1.A    --------------------------------------------------------------------------------

Target      LLQEEAEKMKQVAEEAARLSVAAQEAARLRQLAEEDLAQQRALAEKMLKEKMQAVQEATRLKAEAELLQQQKELAQEQAR
4d1e.1.A    --------------------------------------------------------------------------------

Target      RLQEDKEQMAQQLAQETQGFQRTLETERQRQLEMSAEAERLRLRVAEMSRAQARAEEDAQRFRKQAEEIGAKLHRTELAT
4d1e.1.A    --------------------------------------------------------------------------------

Target      QEKVTLVQTLETQRQQSDRDADRLREAIAELEREKDKLKKEAELLQLKSEEMQTVQQEQLLQETQALQQSFLSEKDSLLQ
4d1e.1.A    --------------------------------------------------------------------------------

Target      RERFIEEEKAKLERLFQDEVAKAQKLREEQQRQQQMQQEKQQLLASMEEARRRQHEAEEGVRRKQEELQLLEQQRQQQE
4d1e.1.A    --------------------------------------------------------------------------------

Target      QLLAEENRRLRERLEHLEEEHRAALAHSEEITAAQAAATRALPNGQDAADGPAAEPEHAFEGLRQKVPAQQLQEAGILST
4d1e.1.A    --------------------------------------------------------------------------------

Target      EELQRLAQGHTTVAELTQREDVRRYLQGHSSIAGLLLKPANEKLTIYAALRRQLLSPGTALILLEAQAASGFLLDPVRNR
4d1e.1.A    --------------------------------------------------------------------------------

Target      RLTVTEAVKEGVVGPELHHKLLSAERAVTGYKDPYTGEKISLFQAMKKDLIVREHGIRLLEAQIATGGVIDPVHSHRVPV
4d1e.1.A    --------------------------------------------------------------------------------

Target      DVAYHRGYFDEEMNRVLQDPSDDTKGFFDPNTHENLTYLQLLERCVEDPETGLRLLPLTDQAAKGGELVYTDSEARDVFE
4d1e.1.A    --------------------------------------------------------------------------------

Target      KATVSAPFGKFQGKTVTIWELINSEYFTAEQRRDLLRQFRTGKVTVEKIIKIVITVIEEHEQKGQLCFQGLRALVPAAEL
4d1e.1.A    --------------------------------------------------------------------------------

Target      LESGIIDWDLFRQLQLGERSVQEVAEVEGVRRALRGSGVIAGVWLEEARQKLSIYEALKKELLQPEAAVALLEAQAGTGH
4d1e.1.A    --------------------------------------------------------------------------------

Target      VIDPATSARLTVDEAVRAGLVGPELHEKLLSAEKAVTGYKDPYSGQSVSLFQALKKGLIPREQGLRLLDAQLSTGGTVDP
4d1e.1.A    --------------------------------------------------------------------------------

Target      SKSHRLPLDVACARGYLDEETSTALSAPRDDAKTYYDPRTWELATYSQLQQQCRPDPLTGLSLLPLSEEAARARQQELYS
4d1e.1.A    --------------------------------------------------------------------------------

Target      EVQAREAFQKATVEVPVGSFQGRAVTIWELINSEYFTAEQRQELLRQFRTGKVTVEKIIKIVITIVEEVETTRRERLSFS
4d1e.1.A    --------------------------------------------------------------------------------

Target      GLRAPVPASELLAAGILSSSQFEQLKDGKTSVKDLSELSSLQTLLQGSGCLAGIYLEESKEKVTIYEAMRRGLLRPSTAI
4d1e.1.A    --------------------------------------------------------------------------------

Target      LLLEAQAATGFLVDPVRNQRLYVHEAVKAGVVGPELHEKLLSAEKAVTGYKDPYSGSTISLFQAMKKGLVVREHGIRLLE
```

```
4dle.1.A   --------------------------------------------------------------------------------

Target     AQIATGGIIDPVHSHRVPVDVACQRGYFDEEMNRVLEDPSDDTKGFFDPNTHENLTYRQLLERCVEDPETGLRLLPLKGP
4dle.1.A   --------------------------------------------------------------------------------

Target     EKAEVVETTRVYTEEETRRAFEETQIDIPGGGSHGGSTMSLWEVMQSDLIPEEQRAQLMADFQAGRVTKERMIIIIIEII
4dle.1.A   --------------------------------------------------------------------------------

Target     EKTEIVRQQNLASYDYVRRRLTAEDLYEARVISRESYSLLREGTRSLREVLEAESAWRYLYGTGCVAGVYLPGSRQTLPI
4dle.1.A   --------------------------------------------------------------------------------

Target     YQALKKGLLSAEVARLLLEAQAATGFLLDPVKGERLTVDEAVRKGLVGPELHDRLLSAERAVTGYRDPYTEQTISLFQAM
4dle.1.A   --------------------------------------------------------------------------------

Target     KKDLIPAEEALRLLDAQLATGGIVDPRLGFHLPLEVAYQRGYLNKDTHDQLSEPSEVRSYVDPSTDERLSYTQLLRRCRR
4dle.1.A   --------------------------------------------------------------------------------

Target     DETSGLFLLPLSEARKLTFRGLRKQITVEELVRSHVMDEATAQRLQEGLTSIEEVSKNLQKFLEGTSSIAGVLVDATKER
4dle.1.A   --------------------------------------------------------------------------------

Target     LSVYQAMKKGIIRPGTAFELLEAQAATGYVIDPIKGLKLTVEEAVRMGIVGPEFKDKLLSAERAVTGYKDPYSGKLISLF
4dle.1.A   --------------------------------------------------------------------------------

Target     QAMKKGLILKDHGIRLLEAQIATGGIIDPEESHRLPVDVAYQRGLFDEEMNEILTDPSDDTKGFFDPNTEENLTYLQLME
4dle.1.A   --------------------------------------------------------------------------------

Target     RCVTDPQTGLRLLPLKEKKRERKTSSKSSVRKRRVVIVDPETGKEMSVYEAYRKGLIDHQTYLELSEQECEWEEITISSS
4dle.1.A   --------------------------------------------------------------------------------

Target     DGVVKSMIIDRRSGRQYDIDEAIAKSLIDRSALDQYRAGTLSITEFADMLSGNAGGFRSRSSSVGSSSSYPISPAVSRTQ
4dle.1.A   --------------------------------------------------------------------------------

Target     LASWSDPTEETGPVAGILDTETLEKVSITEAMHRNLVDNITGQRLLEAQACTGGIIDPNTGERFPVTDAVNKGLVDKIMV
4dle.1.A   --------------------------------------------------------------------------------

Target     DRINLAQKAFCGFEDPRTKTKMSAAQALKKGWLYYEAGQRFLEVQYLTGGLIEPDTPGRVPLDEALQRGTVDARTAQKLR
4dle.1.A   --------------------------------------------------------------------------------

Target     DVSAYSKYLTCPKTKLKISYKDALDRSMVEEGTGLRLLEAAAQSSKGYYSPYSVSGSGSTTGSRSGSRTGSRAGSRRGSF
4dle.1.A   --------------------------------------------------------------------------------

Target     DATGSGFSMTFSSSSYSSSGYGRRYASGPTSSLGGPESAAA
4dle.1.A   -----------------------------------------
```

Model #23	File	Built with	Oligo-State	Ligands	GMQE	QMEANDisCo Global
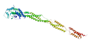	PDB	ProMod3 3.3.0	monomer (matching prediction)	None	0.00	0.57 ± 0.05

Template	Seq Identity	Oligo-state	QSQE	Found by	Method	Resolution	Seq Similarity	Range	Coverage	Description
1sjj.1.B	26.16	homo-dimer	0.00	HHblits	2DX	-	0.33	64 - 737	0.15	actinin

The template contained no ligands.

```
Target     MSGEDQEVRAVVEDGSNGGSGSPSPGDTLPWNLEKTQRSRRGGGGPGGNGSVLDPAERAVIRIADERDRVQKKTFTKWVN
1sjj.1.B   ---------------------------------------------------------------DPAWEKQQRKTFTAWCN

Target     KHLIKAQRHISDLYEDLRDGHNLISLLEVLSGDSLPR-EKGRMRFHKLQNVQIALDYLRHRQVKLVNIRNDDIADGNPKL
1sjj.1.B   SHLRKAGTQIENIEEDFRDGLKLMLLLEVISGERLAKPERGKMRVHKISNVNKALDFIASKGVKLVSIGAEEIVDGNVKM

Target     TLGLIWTIILHFQISDIQVSGGQSEDMTAKEKLLLWSQRMVEGYQGLRCDNFTSSWRDGRLFNAIIHRHKPMLIDMNKVYR
1sjj.1.B   TLGMIWTIILRFAIQDISVE----ETSAKEGLLLWYQRKTAPYKNVNIQNFHISWKDGLGFCALIHRHRPELIDYGKLRK

Target     QTNLENLDQAFSVAERDLGVTRLLDPEDVD-VPQPDEKSIITYVSSLYDAMPRVPDVQDGV--------KANELQLRWQE
```

```
1sjj.1.B  DDPLTNLNTAFDVAEKYLDIPKMLDAEDIVGTARPDEKAIMTYVSSFYHAFSGAQKAETAANRICKVLAVNQENEQLMED

Target    YRELVLLLLQWIRAHTAAFEERRFPSSFEEIEILWCQFLKFKETELPAKEADKNRSKGIYQSLEGAVQAG---QLKVPPG
1sjj.1.B  YEKLASDLLEWIRRTIPWLENRAPENTMQAMQQKLEDFRDYRRLHKPPKVQEKCQLEINFNTLQTKLRLSNRPAFMPSEG

Target    YHPLDVEKEWGKLHVAILEREKQLRSEFERLERLQRIVSKLQMEAGLCEEQLNQADALLQSDV----RLLAAGKAPQRAG
1sjj.1.B  KMVSDINNAWGGLEQAEKGYEEWLLNEIRRLERLDHLAEKFRQKASIHESWTDGKEAMLQQKDYETATLSEIKALLKKHE

Target    EVERDLDKADGMIRLLFNDVQALKDGRHPQGEQMYRRVYRLHERLVAIRTEYNL---RLRGTPRHP----ELEDSTLRYL
1sjj.1.B  AFESDLAAHQDRVEQIAAIAQELNELDYYDSPSVNARCQKICDQWDNLGALTQKRREALERTEKLLETIDQLYLEYAKRA

Target    QDLLAWVEENQRRVDSAEWGVDLPSVEAQLGSHRGLHQSVEEFRAKIERARTDE-------GQLS---PATRGAYRDCLG
1sjj.1.B  APFNNWMEGAMEDLQDTFIVHTIEEIQGLTTAHEQFKATLPDADKERQAILGIHNEVSKIVQTYHVNMAGTNPYTTITPQ

Target    RLDLQYAKLLNSSKARLRSLES----------LH-GFVAAATKELMWLSEKEEEEVGFDWSERNSNMAAKKEAYSALMRE
1sjj.1.B  EINGKWEHVRQLVPRRDQALMEEHARQQQNERLRKQFGAQANVIGPWIQTKMEEIGRISIEMH-GTLEDQLNHLRQYEKS

Target    LELKEKKIKEIQSTGDRLLREDHPARPTVESFQAALQTQWSWMLQLCCCIEAHLKENTAYFQFFSDVREAEEQLRKLQET
1sjj.1.B  IVNYKPKIDQLEGDHQQIQEALIFDNKHTNYTMEHIRVGWEQLLTTIARTINEVENQILTRD------------------

Target    LHRKYTCDRSITVTRLEDLLQDAQDEKDQLNEYRGHLSGLAKRAKAIVQLTPRNPTQPTRGRVPLLAVCDYKQVEATVHK
1sjj.1.B  --------------------------------------------------------------------------------

Target    GDECQMLGPAQPFHWKVLSSSGSEAAVPSVCFLVPPPNQEALEAVARLEAQHQALVTLWHQLHTDMKSLLAWQSLSRDVQ
1sjj.1.B  --------------------------------------------------------------------------------

Target    LIRSWSLVTFRTLKPEEQRQALRSLELHYQAFLRDSQDAGGFGPEDRLQAEREYGSCSRHYQQLLQSLEQGEQEESRCQR
1sjj.1.B  --------------------------------------------------------------------------------

Target    CISELKDIRLQLEACETRTVHRLRLPLDKEPARECAQRIAEQQKAQAEVEGLGKGVARLSAEAEKVLALPEPSPAAPTLR
1sjj.1.B  --------------------------------------------------------------------------------

Target    SELELTLGKLEQVRSLSAIYLEKLKTISLVIRSTQGAEEALRAHEEQLKEAQAVPAALPELEATKAAMKKLRAQAEAQQP
1sjj.1.B  --------------------------------------------------------------------------------

Target    VFDALRDELRGAQEVGERLQQRHGERDVEVERWRERVTQLLERWQAVLAQTDVRQRELEQLGRQLRYYRESADPLGAWLQ
1sjj.1.B  --------------------------------------------------------------------------------

Target    DARRRQEQIQAVPLADSQAVREQLRQEKALLEEIERHAEKVEECQRFAKQYINAIKDYELQLVTYKAQLEPVASPAKKPK
1sjj.1.B  --------------------------------------------------------------------------------

Target    VQSGSESVIQEYVDLRTRYSELSTLTSQYIRFISETLRRMEEEERLAEQQRAEERERLAEVEAALEKQRQLAEAHAQAKA
1sjj.1.B  --------------------------------------------------------------------------------

Target    QAEREAQELQRRMQEEVARREEVAVDAQQQKRSIQEELQQLRQSSEAEIQAKARQVEAAERSRLRIEEEIRVVRLQLETT
1sjj.1.B  --------------------------------------------------------------------------------

Target    ERQRGGAEGELQALRARAEEAEAQKRQAQEEAERLRRQVQEETQRKRQAEAELGLRVKAEAEAAREKQRALQALEELRLQ
1sjj.1.B  --------------------------------------------------------------------------------

Target    AEEAERRLRQAEAERARQVQVALETAQRSAQAELQSKHASFAEKTAQLERTLEEEHVTVVQLREEATRREQQQAEAERAR
1sjj.1.B  --------------------------------------------------------------------------------

Target    EEAERELERWQLKANEALRLRLQAEEVAQQKSLAQAEAEKQKEAAEREARRRGKAEEQAVRQRELAEQELERQRQLAEGT
1sjj.1.B  --------------------------------------------------------------------------------

Target    AQQRLAAEQELIRLRAETEQGEQQRQLLEEELARLQSEAAAATQKRQELEAELAKVRAEMEVLLASKARAEEESRSSSEK
1sjj.1.B  --------------------------------------------------------------------------------

Target    SKQRLEAEAGRFRELAEEAARLRALAEEAKRQRQLAEEDAARQRAEAERVLSEKLAAISEATRLKTEAEIALKEKEAENE
1sjj.1.B  --------------------------------------------------------------------------------

Target    RLRRLAEDEAFQRRRLEEQAAQHKADIEERLAQLRKASESELERQKGLVEDTLRQRRQVEEEILALKASFEKAAAGKAEL
1sjj.1.B  --------------------------------------------------------------------------------

Target    ELELGRIRGNAEDTLRSKEQAEQEAARQRQLAAEEERRRREAEERVQKSLAAEEEAARQRKAALEEVERLKAKVEEARRL
1sjj.1.B  --------------------------------------------------------------------------------

Target    RERAEHESVRQLQLAQEAAQKRLQAEEKAHAFAVQQKEQELQQTLQQEQSVLEKLRSEAEAARRAAEEAEEARERAEREA
1sjj.1.B  --------------------------------------------------------------------------------

Target    AQSRQRVEEAERLKQAAEEQAQAQAQAQAAAEKLRKEAEQEAARRAQAEQAALRQKQAADAEMEKHKKFAEQTLRQKAQV
1sjj.1.B  --------------------------------------------------------------------------------
```

```
Target     EQELTALRLKLEETDHQKSILDQELQRLKAEVTEAARQRSQVEEELFSVRVQMEELGKLKARIEAENRALILRDKDNTQR
1sjj.1.B   --------------------------------------------------------------------------------

Target     LLQEEAEKMKQVAEEAARLSVAAQEAARLRQLAEEDLAQQRALAEKMLKEKMQAVQEATRLKAEAELLQQQKELAQEQAR
1sjj.1.B   --------------------------------------------------------------------------------

Target     RLQEDKEQMAQQLAQETQGFQRTLETERQRQLEMSAEAERLRLRVAEMSRAQARAEEDAQRFRKQAEEIGAKLHRTELAT
1sjj.1.B   --------------------------------------------------------------------------------

Target     QEKVTLVQTLETQRQQSDRDADRLREAIAELEREKDKLKKEAELLQLKSEEMQTVQQEQLLQETQALQQSFLSEKDSLLQ
1sjj.1.B   --------------------------------------------------------------------------------

Target     RERFIEEEKAKLERLFQDEVAKAQKLREEQQRQQQMQQEKQQLLASMEEARRRQHEAEEGVRRKQEELQLLEQQRQQQE
1sjj.1.B   --------------------------------------------------------------------------------

Target     QLLAEENRRLRERLEHLEEEHRAALAHSEEITAAQAAATRALPNGQDAADGPAAEPEHAFEGLRQKVPAQQLQEAGILST
1sjj.1.B   --------------------------------------------------------------------------------

Target     EELQRLAQGHTTVAELTQREDVRRYLQGHSSIAGLLLKPANEKLTIYAALRRQLLSPGTALILLEAQAASGFLLDPVRNR
1sjj.1.B   --------------------------------------------------------------------------------

Target     RLTVTEAVKEGVVGPELHHKLLSAERAVTGYKDPYTGEKISLFQAMKKDLIVREHGIRLLEAQIATGGVIDPVHSHRVPV
1sjj.1.B   --------------------------------------------------------------------------------

Target     DVAYHRGYFDEEMNRVLQDPSDDTKGFFDPNTHENLTYLQLLERCVEDPETGLRLLPLTDQAAKGGELVYTDSEARDVFE
1sjj.1.B   --------------------------------------------------------------------------------

Target     KATVSAPFGKFQGKTVTIWELINSEYFTAEQRRDLLRQFRTGKVTVEKIIKIVITVIEEHEQKGQLCFQGLRALVPAAEL
1sjj.1.B   --------------------------------------------------------------------------------

Target     LESGIIDWDLFRQLQLGERSVQEVAEVEGVRRALRGSGVIAGVWLEEARQKLSIYEALKKELLQPEAAVALLEAQAGTGH
1sjj.1.B   --------------------------------------------------------------------------------

Target     VIDPATSARLTVDEAVRAGLVGPELHEKLLSAEKAVTGYKDPYSGQSVSLFQALKKGLIPREQGLRLLDAQLSTGGTVDP
1sjj.1.B   --------------------------------------------------------------------------------

Target     SKSHRLPLDVACARGYLDEETSTALSAPRDDAKTYYDPRTWELATYSQLQQQCRPDPLTGLSLLPLSEEAARARQQELYS
1sjj.1.B   --------------------------------------------------------------------------------

Target     EVQAREAFQKATVEVPVGSFQGRAVTIWELINSEYFTAEQRQELLRQFRTGKVTVEKIIKIVITIVEEVETTRRERLSFS
1sjj.1.B   --------------------------------------------------------------------------------

Target     GLRAPVPASELLAAGILSSSQFEQLKDGKTSVKDLSELSSLQTLLQGSGCLAGIYLEESKEKVTIYEAMRRGLLRPSTAI
1sjj.1.B   --------------------------------------------------------------------------------

Target     LLLEAQAATGFLVDPVRNQRLYVHEAVKAGVVGPELHEKLLSAEKAVTGYKDPYSGSTISLFQAMKKGLVVREHGIRLLE
1sjj.1.B   --------------------------------------------------------------------------------

Target     AQIATGGIIDPVHSHRVPVDVACQRGYFDEEMNRVLEDPSDDTKGFFDPNTHENLTYRQLLERCVEDPETGLRLLPLKGP
1sjj.1.B   --------------------------------------------------------------------------------

Target     EKAEVVETTRVYTEEETRRAFEETQIDIPGGGSHGGSTMSLWEVMQSDLIPEEQRAQLMADFQAGRVTKERMIIIIIEII
1sjj.1.B   --------------------------------------------------------------------------------

Target     EKTEIVRQQNLASYDYVRRRLTAEDLYEARVISRESYSLLREGTRSLREVLEAESAWRYLYGTGCVAGVYLPGSRQTLPI
1sjj.1.B   --------------------------------------------------------------------------------

Target     YQALKKGLLSAEVARLLLEAQAATGFLLDPVKGERLTVDEAVRKGLVGPELHDRLLSAERAVTGYRDPYTEQTISLFQAM
1sjj.1.B   --------------------------------------------------------------------------------

Target     KKDLIPAEEALRLLDAQLATGGIVDPRLGFHLPLEVAYQRGYLNKDTHDQLSEPSEVRSYVDPSTDERLSYTQLLRRCRR
1sjj.1.B   --------------------------------------------------------------------------------

Target     DETSGLFLLPLSEARKLTFRGLRKQITVEELVRSHVMDEATAQRLQEGLTSIEEVSKNLQKFLEGTSSIAGVLVDATKER
1sjj.1.B   --------------------------------------------------------------------------------

Target     LSVYQAMKKGIIRPGTAFELLEAQAATGYVIDPIKGLKLTVEEAVRMGIVGPEFKDKLLSAERAVTGYKDPYSGKLISLF
1sjj.1.B   --------------------------------------------------------------------------------

Target     QAMKKGLILKDHGIRLLEAQIATGGIIDPEESHRLPVDVAYQRGLFDEEMNEILTDPSDDTKGFFDPNTEENLTYLQLME
1sjj.1.B   --------------------------------------------------------------------------------

Target     RCVTDPQTGLRLLPLKEKKRERKTSSKSSVRKRRVVIVDPETGKEMSVYEAYRKGLIDHQTYLELSEQECEWEEITISSS
1sjj.1.B   --------------------------------------------------------------------------------
```

```
Target     DGVVKSMIIDRRSGRQYDIDEAIAKSLIDRSALDQYRAGTLSITEFADMLSGNAGGFRSRSSSVGSSSSYPISPAVSRTQ
1sjj.1.B   --------------------------------------------------------------------------------

Target     LASWSDPTEETGPVAGILDTETLEKVSITEAMHRNLVDNITGQRLLEAQACTGGIIDPNTGERFPVTDAVNKGLVDKIMV
1sjj.1.B   --------------------------------------------------------------------------------

Target     DRINLAQKAFCGFEDPRTKTKMSAAQALKKGWLYYEAGQRFLEVQYLTGGLIEPDTPGRVPLDEALQRGTVDARTAQKLR
1sjj.1.B   --------------------------------------------------------------------------------

Target     DVSAYSKYLTCPKTKLKISYKDALDRSMVEEGTGLRLLEAAAQSSKGYYSPYSVSGSGSTTGSRSGSRTGSRAGSRRGSF
1sjj.1.B   --------------------------------------------------------------------------------

Target     DATGSGFSMTFSSSSYSSSGYGRRYASGPTSSLGGPESAAA
1sjj.1.B   -----------------------------------------
```

Model #03	File	Built with	Oligo-State	Ligands	GMQE	QMEANDisCo Global
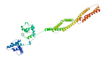	PDB	ProMod3 3.3.0	monomer	None	0.00	0.55 ± 0.05

Template	Seq Identity	Oligo-state	QSQE	Found by	Method	Resolution	Seq Similarity	Range	Coverage	Description
8iah.1.2	38.97	monomer	0.00	BLAST	EM	-	0.38	57 - 526	0.12	Spectrin beta chain

Excluded ligands

Ligand Name.Number	Reason for Exclusion	Description
ADP.1	Binding site not conserved.	ADENOSINE-5'-DIPHOSPHATE
ADP.2	Binding site not conserved.	ADENOSINE-5'-DIPHOSPHATE
ADP.3	Binding site not conserved.	ADENOSINE-5'-DIPHOSPHATE
ADP.4	Binding site not conserved.	ADENOSINE-5'-DIPHOSPHATE
ADP.5	Binding site not conserved.	ADENOSINE-5'-DIPHOSPHATE
ADP.6	Binding site not conserved.	ADENOSINE-5'-DIPHOSPHATE
ADP.7	Binding site not conserved.	ADENOSINE-5'-DIPHOSPHATE
ADP.8	Binding site not conserved.	ADENOSINE-5'-DIPHOSPHATE
ADP.9	Binding site not conserved.	ADENOSINE-5'-DIPHOSPHATE
ADP.10	Binding site not conserved.	ADENOSINE-5'-DIPHOSPHATE
ADP.11	Binding site not conserved.	ADENOSINE-5'-DIPHOSPHATE
ADP.12	Binding site not conserved.	ADENOSINE-5'-DIPHOSPHATE

```
Target     MSGEDQEVRAVVEDGSNGGSGSPSPGDTLPWNLEKTQRSRRGGGGPGGNGSVLDPAERAVIR-IADERDRVQKKTFTKWV
8iah.1.2   --------------------------------------------------------ERSRIKALADEREVVQKKTFTKWV

Target     NKHLIKAQRHISDLYEDLRDGHNLISLLEVLSGDSLPR-EKGRMRFHKLQNVQIALDYLRHRQVKLVNIRNDDIADGNPK
8iah.1.2   NSHLARVSCRITDLYKDLRDGRMLIKLLEVLSGEMLPKPTKGKMRIHCLENVDKALQFLKEQRVHLENMGSHDIVDGNHR

Target     LTLGLIWTIILHFQISDIQVSGQS--EDMTAKEKLLLWSQRMVEGYQGLRCDNFTSSWRDGRLFNAIIHRHKPMLIDMNK
8iah.1.2   LVLGLIWTIILRFQIQDIVVQTQEGRETRSAKDALLLWCQMKTAGYPNVNVTNFTSSWKDGLAFNALIHKHRPDLIDFDK

Target     VYRQTNLENLDQAFSVAERDLGVTRLLDPEDVDVPQPDEKSIITYVSSLYDAMP----------RVPDVQDGVKANELQL
8iah.1.2   LKDSNARHNLEHAFDVAERQLGIIQLLDPEDVFTENPDEKSIITYVVAFYHYFSKMKVLAVEGKRVGKVIDHAIETEKMI

Target     RWQEYRELVLLLLQWIRAHTAAFEERRFPSSFEEIEILWCQFLKFKETELPAKEADKNRSKGIYQSLEGAVQAGQLKV--
8iah.1.2   --EKYSGLASDLLTWIEQTITVLNSRKFANSLAGVQQQLQAFSTYRTVEKPPKFQEKGNLEVLLFTIQSRMRANNQKVYT

Target     -PPGYHPLDVEKEWGKLHVAILEREKQLRSEFERLERLQRIVSKLQMEAGLCEEQLNQADALLQSD---VRLLAAGKAPQ
8iah.1.2   PHDGKLVSDINRAWESLEEAEYRRELALRSELIRQEKLEQLARRFDRKAAMRETWLNENQRLVAQDNFGYDLAAVEAAKK

Target     RAGEVERDLDKADGMIRLLFNDVQALK-DGRHPQGEQMYRR--VYRLHERLVAIRTEYNLRLRGTPRHPELEDSTLRYLQ
8iah.1.2   KHEAIETDTAAYEERVRALEDLARELELENYHDQKRITARKDNILRLWNYLQELLQSRRQRLETTLALQQL--------FQ
```

```
Target      DLLA---WVEENQRRVDSAEWGVDLPSVEAQLGSHRGLHQSVEEFRAKIERAR------TDEGQLSPATRGAYRDCLGRL
8iah.1.2    DMLHSIDWMDEIKAHLLSAEFGKHLLEAEDLLQKHKLMEADIAIQGDKVKAITAATLQFTEETGYQPCDPQVIRDRVSHL

Target      DLQYAKLLNSSKARLRSLESLHGFVAAATKELMWLSEKEEEEVGFDWSERNSNMAAKKEAYSALMRELELKEKKIKEIQS
8iah.1.2    EQCFAELSNTAAGRKAQLE-------------------------------------------------------------

Target      TGDRLLREDHPARPTVESFQAALQTQWSWMLQLCCCIEAHLKENTAYFQFFSDVREAEEQLRKLQETLHRKYTCDRSITV
8iah.1.2    --------------------------------------------------------------------------------

Target      TRLEDLLQDAQDEKDQLNEYRGHLSGLAKRAKAIVQLTPRNPTQPTRGRVPLLAVCDYKQVEATVHKGDECQMLGPAQPF
8iah.1.2    --------------------------------------------------------------------------------

Target      HWKVLSSSGSEAAVPSVCFLVPPPNQEALEAVARLEAQHQALVTLWHQLHTDMKSLLAWQSLSRDVQLIRSWSLVTFRTL
8iah.1.2    --------------------------------------------------------------------------------

Target      KPEEQRQALRSLELHYQAFLRDSQDAGGFGPEDRLQAEREYGSCSRHYQQLLQSLEQGEQEESRCQRCISELKDIRLQLE
8iah.1.2    --------------------------------------------------------------------------------

Target      ACETRTVHRLRLPLDKEPARECAQRIAEQQKAQAEVEGLGKGVARLSAEAEKVLALPEPSPAAPTLRSELELTLGKLEQV
8iah.1.2    --------------------------------------------------------------------------------

Target      RSLSAIYLEKLKTISLVIRSTQGAEEALRAHEEQLKEAQAVPAALPELEATKAAMKKLRAQAEAQQPVFDALRDELRGAQ
8iah.1.2    --------------------------------------------------------------------------------

Target      EVGERLQQRHGERDVEVERWRERVTQLLERWQAVLAQTDVRQRELEQLGRQLRYYRESADPLGAWLQDARRRQEQIQAVP
8iah.1.2    --------------------------------------------------------------------------------

Target      LADSQAVREQLRQEKALLEEIERHAEKVEECQRFAKQYINAIKDYELQLVTYKAQLEPVASPAKKPKVQSGSESVIQEYV
8iah.1.2    --------------------------------------------------------------------------------

Target      DLRTRYSELSTLTSQYIRFISETLRRMEEEERLAEQQRAEERERLAEVEAALEKQRQLAEAHAQAKAQAEREAQELQRRM
8iah.1.2    --------------------------------------------------------------------------------

Target      QEEVARREEVAVDAQQQKRSIQEELQQLRQSSEAEIQAKARQVEAAERSRLRIEEEIRVVRLQLETTERQRGGAEGELQA
8iah.1.2    --------------------------------------------------------------------------------

Target      LRARAEEAEAQKRQAQEEAERLRRQVQEETQRKRQAEAELGLRVKAEAEAAREKQRALQALEELRLQAEEAERRLRQAEA
8iah.1.2    --------------------------------------------------------------------------------

Target      ERARQVQVALETAQRSAQAELQSKHASFAEKTAQLERTLEEEHVTVVQLREEATRREQQQAEAERAREEAERELERWQLK
8iah.1.2    --------------------------------------------------------------------------------

Target      ANEALRLRLQAEEVAQQKSLAQAEAEKQKEAAEREARRRGKAEEQAVRQRELAEQELERQRQLAEGTAQQRLAAEQELIR
8iah.1.2    --------------------------------------------------------------------------------

Target      LRAETEQGEQQRQLLEEELARLQSEAAAATQKRQELEAELAKVRAEMEVLLASKARAEEESRSSSEKSKQRLEAEAGRFR
8iah.1.2    --------------------------------------------------------------------------------

Target      ELAEEAARLRALAEEAKRQRQLAEEDAARQRAEAERVLSEKLAAISEATRLKTEAEIALKEKEAENERLRRLAEDEAFQR
8iah.1.2    --------------------------------------------------------------------------------

Target      RRLEEQAAQHKADIEERLAQLRKASESELERQKGLVEDTLRQRRQVEEEILALKASFEKAAAGKAELELELGRIRGNAED
8iah.1.2    --------------------------------------------------------------------------------

Target      TLRSKEQAEQEAARQRQLAAEEERRRREAEERVQKSLAAEEEAARQRKAALEEVERLKAKVEEARRLRERAEHESVRQLQ
8iah.1.2    --------------------------------------------------------------------------------

Target      LAQEAAQKRLQAEEKAHAFAVQQKEQELQQTLQQEQSVLEKLRSEAEAARRAAEEAEEARERAEREAAQSRQRVEEAERL
8iah.1.2    --------------------------------------------------------------------------------

Target      KQAAEEQAQAQAQAQAAAEKLRKEAEQEAARRAQAEQAALRQKQAADAEMEKHKKFAEQTLRQKAQVEQELTALRLKLEE
8iah.1.2    --------------------------------------------------------------------------------

Target      TDHQKSILDQELQRLKAEVTEAARQRSQVEEELFSVRVQMEELGKLKARIEAENRALILRDKDNTQRLLQEEAEKMKQVA
8iah.1.2    --------------------------------------------------------------------------------

Target      EEAARLSVAAQEAARLRQLAEEDLAQQRALAEKMLKEKMQAVQEATRLKAEAELLQQQKELAQEQARRLQEDKEQMAQQL
8iah.1.2    --------------------------------------------------------------------------------

Target      AQETQGFQRTLETERQRQLEMSAEAERLRLRVAEMSRAQARAEEDAQRFRKQAEEIGAKLHRTELATQEKVTLVQTLETQ
8iah.1.2    --------------------------------------------------------------------------------

Target      RQQSDRDADRLREAIAELEREKDKLKKEAELLQLKSEEMQTVQQEQLLQETQALQQSFLSEKDSLLQRERFIEEEKAKLE
```

```
8iah.1.2    --------------------------------------------------------------------------------

Target      RLFQDEVAKAQKLREEQQRQQQQMQQEKQQLLASMEEARRRQHEAEEGVRRKQEELQLLEQQRQQQEQLLAEENRRLRER
8iah.1.2    --------------------------------------------------------------------------------

Target      LEHLEEEHRAALAHSEEITAAQAAATRALPNGQDAADGPAAEPEHAFEGLRQKVPAQQLQEAGILSTEELQRLAQGHTTV
8iah.1.2    --------------------------------------------------------------------------------

Target      AELTQREDVRRYLQGHSSIAGLLLKPANEKLTIYAALRRQLLSPGTALILLEAQAASGFLLDPVRNRRLTVTEAVKEGVV
8iah.1.2    --------------------------------------------------------------------------------

Target      GPELHHKLLSAERAVTGYKDPYTGEKISLFQAMKKDLIVREHGIRLLEAQIATGGVIDPVHSHRVPVDVAYHRGYFDEEM
8iah.1.2    --------------------------------------------------------------------------------

Target      NRVLQDPSDDTKGFFDPNTHENLTYLQLLERCVEDPETGLRLLPLTDQAAKGGELVYTDSEARDVFEKATVSAPFGKFQG
8iah.1.2    --------------------------------------------------------------------------------

Target      KTVTIWELINSEYFTAEQRRDLLRQFRTGKVTVEKIIKIVITVIEEHEQKGQLCFQGLRALVPAAELLESGIIDWDLFRQ
8iah.1.2    --------------------------------------------------------------------------------

Target      LQLGERSVQEVAEVEGVRRALRGSGVIAGVWLEEARQKLSIYEALKKELLQPEAAVALLEAQAGTGHVIDPATSARLTVD
8iah.1.2    --------------------------------------------------------------------------------

Target      EAVRAGLVGPELHEKLLSAEKAVTGYKDPYSGQSVSLFQALKKGLIPREQGLRLLDAQLSTGGTVDPSKSHRLPLDVACA
8iah.1.2    --------------------------------------------------------------------------------

Target      RGYLDEETSTALSAPRDDAKTYYDPRTWELATYSQLQQQCRPDPLTGLSLLPLSEEAARARQQELYSEVQAREAFQKATV
8iah.1.2    --------------------------------------------------------------------------------

Target      EVPVGSFQGRAVTIWELINSEYFTAEQRQELLRQFRTGKVTVEKIIKIVITIVEEVETTRRERLSFSGLRAPVPASELLA
8iah.1.2    --------------------------------------------------------------------------------

Target      AGILSSSQFEQLKDGKTSVKDLSELSSLQTLLQGSGCLAGIYLEESKEKVTIYEAMRRGLLRPSTAILLLEAQAATGFLV
8iah.1.2    --------------------------------------------------------------------------------

Target      DPVRNQRLYVHEAVKAGVVGPELHEKLLSAEKAVTGYKDPYSGSTISLFQAMKKGLVVREHGIRLLEAQIATGGIIDPVH
8iah.1.2    --------------------------------------------------------------------------------

Target      SHRVPVDVACQRGYFDEEMNRVLEDPSDDTKGFFDPNTHENLTYRQLLERCVEDPETGLRLLPLKGPEKAEVVETTRVYT
8iah.1.2    --------------------------------------------------------------------------------

Target      EEETRRAFEETQIDIPGGGSHGGSTMSLWEVMQSDLIPEEQRAQLMADFQAGRVTKERMIIIIIEIIEKTEIVRQQNLAS
8iah.1.2    --------------------------------------------------------------------------------

Target      YDYVRRRLTAEDLYEARVISRESYSLLREGTRSLREVLEAESAWRYLYGTGCVAGVYLPGSRQTLPIYQALKKGLLSAEV
8iah.1.2    --------------------------------------------------------------------------------

Target      ARLLLEAQAATGFLLDPVKGERLTVDEAVRKGLVGPELHDRLLSAERAVTGYRDPYTEQTISLFQAMKKDLIPAEEALRL
8iah.1.2    --------------------------------------------------------------------------------

Target      LDAQLATGGIVDPRLGFHLPLEVAYQRGYLNKDTHDQLSEPSEVRSYVDPSTDERLSYTQLLRRCRRDETSGLFLLPLSE
8iah.1.2    --------------------------------------------------------------------------------

Target      ARKLTFRGLRKQITVEELVRSHVMDEATAQRLQEGLTSIEEVSKNLQKFLEGTSSIAGVLVDATKERLSVYQAMKKGIIR
8iah.1.2    --------------------------------------------------------------------------------

Target      PGTAFELLEAQAATGYVIDPIKGLKLTVEEAVRMGIVGPEFKDKLLSAERAVTGYKDPYSGKLISLFQAMKKGLILKDHG
8iah.1.2    --------------------------------------------------------------------------------

Target      IRLLEAQIATGGIIDPEESHRLPVDVAYQRGLFDEEMNEILTDPSDDTKGFFDPNTEENLTYLQLMERCVTDPQTGLRLL
8iah.1.2    --------------------------------------------------------------------------------

Target      PLKEKKRERKTSSKSSVRKRRVVIVDPETGKEMSVYEAYRKGLIDHQTYLELSEQECEWEEITISSSDGVVKSMIIDRRS
8iah.1.2    --------------------------------------------------------------------------------

Target      GRQYDIDEAIAKSLIDRSALDQYRAGTLSITEFADMLSGNAGGFRSRSSSVGSSSSYPISPAVSRTQLASWSDPTEETGP
8iah.1.2    --------------------------------------------------------------------------------

Target      VAGILDTETLEKVSITEAMHRNLVDNITGQRLLEAQACTGGIIDPNTGERFPVTDAVNKGLVDKIMVDRINLAQKAFCGF
8iah.1.2    --------------------------------------------------------------------------------

Target      EDPRTKTKMSAAQALKKGWLYYEAGQRFLEVQYLTGGLIEPDTPGRVPLDEALQRGTVDARTAQKLRDVSAYSKYLTCPK
8iah.1.2    --------------------------------------------------------------------------------
```

```
Target     TKLKISYKDALDRSMVEEGTGLRLLEAAAQSSKGYYSPYSVSGSGSTTGSRSGSRTGSRAGSRRGSFDATGSGFSMTFSS
8iah.1.2   --------------------------------------------------------------------------------

Target     SSYSSSGYGRRYASGPTSSLGGPESAAA
8iah.1.2   ----------------------------
```

Model #22	File	Built with	Oligo-State	Ligands	GMQE	QMEANDisCo Global
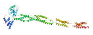	PDB	ProMod3 3.3.0	monomer (matching prediction)	None	0.00	0.57 ± 0.05

Template	Seq Identity	Oligo-state	QSQE	Found by	Method	Resolution	Seq Similarity	Range	Coverage	Description
1sjj.1.A	26.16	homo-dimer	0.00	HHblits	2DX	-	0.33	64 - 737	0.15	actinin

The template contained no ligands.

```
Target     MSGEDQEVRAVVEDGSNGGSGSPSPGDTLPWNLEKTQRSRRGGGGPGGNGSVLDPAERAVIRIADERDRVQKKTFTKWVN
1sjj.1.A   ---------------------------------------------------------------DPAWEKQQRKTFTAWCN

Target     KHLIKAQRHISDLYEDLRDGHNLISLLEVLSGDSLPR-EKGRMRFHKLQNVQIALDYLRHRQVKLVNIRNDDIADGNPKL
1sjj.1.A   SHLRKAGTQIENIEEDFRDGLKLMLLLEVISGERLAKPERGKMRVHKISNVNKALDFIASKGVKLVSIGAEEIVDGNVKM

Target     TLGLIWTIILHFQISDIQVSGQSEDMTAKEKLLLWSQRMVEGYQGLRCDNFTSSWRDGRLFNAIIHRHKPMLIDMNKVYR
1sjj.1.A   TLGMIWTIILRFAIQDISVE----ETSAKEGLLLWYQRKTAPYKNVNIQNFHISWKDGLGFCALIHRHRPELIDYGKLRK

Target     QTNLENLDQAFSVAERDLGVTRLLDPEDVD-VPQPDEKSIITYVSSLYDAMPRVPDVQDGV--------KANELQLRWQE
1sjj.1.A   DDPLTNLNTAFDVAEKYLDIPKMLDAEDIVGTARPDEKAIMTYVSSFYHAFSGAQKAETAANRICKVLAVNQENEQLMED

Target     YRELVLLLLQWIRAHTAAFEERRFPSSFEEIEILWCQFLKFKETELPAKEADKNRSKGIYQSLEGAVQAG---QLKVPPG
1sjj.1.A   YEKLASDLLEWIRRTIPWLENRAPENTMQAMQQKLEDFRDYRRLHKPPKVQEKCQLEINFNTLQTKLRLSNRPAFMPSEG

Target     YHPLDVEKEWGKLHVAILEREKQLRSEFERLERLQRIVSKLQMEAGLCEEQLNQADALLQSDV----RLLAAGKAPQRAG
1sjj.1.A   KMVSDINNAWGGLEQAEKGYEEWLLNEIRRLERLDHLAEKFRQKASIHESWTDGKEAMLQQKDYETATLSEIKALLKKHE

Target     EVERDLDKADGMIRLLFNDVQALKDGRHPQGEQMYRRVYRLHERLVAIRTEYNL---RLRGTPRHP----ELEDSTLRYL
1sjj.1.A   AFESDLAAHQDRVEQIAAIAQELNELDYYDSPSVNARCQKICDQWDNLGALTQKRREALERTEKLLETIDQLYLEYAKRA

Target     QDLLAWVEENQRRVDSAEWGVDLPSVEAQLGSHRGLHQSVEEFRAKIERARTDE-------GQLS---PATRGAYRDCLG
1sjj.1.A   APFNNWMEGAMEDLQDTFIVHTIEEIQGLTTAHEQFKATLPDADKERQAILGIHNEVSKIVQTYHVNMAGTNPYTTITPQ

Target     RLDLQYAKLLNSSKARLRSLES----------LH-GFVAAATKELMWLSEKEEEEVGFDWSERNSNMAAKKEAYSALMRE
1sjj.1.A   EINGKWEHVRQLVPRRDQALMEEHARQQQNERLRKQFGAQANVIGPWIQTKMEEIGRISIEMH-GTLEDQLNHLRQYEKS

Target     LELKEKKIKEIQSTGDRLLREDHPARPTVESFQAALQTQWSWMLQLCCCIEAHLKENTAYFQFFSDVREAEEQLRKLQET
1sjj.1.A   IVNYKPKIDQLEGDHQQIQEALIFDNKHTNYTMEHIRVGWEQLLTTIARTINEVENQILTRD------------------

Target     LHRKYTCDRSITVTRLEDLLQDAQDEKDQLNEYRGHLSGLAKRAKAIVQLTPRNPTQPTRGRVPLLAVCDYKQVEATVHK
1sjj.1.A   --------------------------------------------------------------------------------

Target     GDECQMLGPAQPFHWKVLSSSGSEAAVPSVCFLVPPPNQEALEAVARLEAQHQALVTLWHQLHTDMKSLLAWQSLSRDVQ
1sjj.1.A   --------------------------------------------------------------------------------

Target     LIRSWSLVTFRTLKPEEQRQALRSLELHYQAFLRDSQDAGGFGPEDRLQAEREYGSCSRHYQQLLQSLEQGEQEESRCQR
1sjj.1.A   --------------------------------------------------------------------------------

Target     CISELKDIRLQLEACETRTVHRLRLPLDKEPARECAQRIAEQQKAQAEVEGLGKGVARLSAEAEKVLALPEPSPAAPTLR
1sjj.1.A   --------------------------------------------------------------------------------

Target     SELELTLGKLEQVRSLSAIYLEKLKTISLVIRSTQGAEEALRAHEEQLKEAQAVPAALPELEATKAAMKKLRAQAEAQQP
1sjj.1.A   --------------------------------------------------------------------------------

Target     VFDALRDELRGAQEVGERLQQRHGERDVEVERWRERVTQLLERWQAVLAQTDVRQRELEQLGRQLRYYRESADPLGAWLQ
1sjj.1.A   --------------------------------------------------------------------------------
```

```
Target     DARRRQEQIQAVPLADSQAVREQLRQEKALLEEIERHAEKVEECQRFAKQYINAIKDYELQLVTYKAQLEPVASPAKKPK
1sjj.1.A   --------------------------------------------------------------------------------

Target     VQSGSESVIQEYVDLRTRYSELSTLTSQYIRFISETLRRMEEEERLAEQQRAEERERLAEVEAALEKQRQLAEAHAQAKA
1sjj.1.A   --------------------------------------------------------------------------------

Target     QAEREAQELQRRMQEEVARREEVAVDAQQQKRSIQEELQQLRQSSEAEIQAKARQVEAAERSRLRIEEEIRVVRLQLETT
1sjj.1.A   --------------------------------------------------------------------------------

Target     ERQRGGAEGELQALRARAEEAEAQKRQAQEEAERLRRQVQEETQRKRQAEAELGLRVKAEAEAAREKQRALQALEELRLQ
1sjj.1.A   --------------------------------------------------------------------------------

Target     AEEAERRLRQAEAERARQVQVALETAQRSAQAELQSKHASFAEKTAQLERTLEEEHVTVVQLREEATRREQQQAEAERAR
1sjj.1.A   --------------------------------------------------------------------------------

Target     EEAERELERWQLKANEALRLRLQAEEVAQQKSLAQAEAEKQKEAAEREARRRGKAEEQAVRQRELAEQELERQRQLAEGT
1sjj.1.A   --------------------------------------------------------------------------------

Target     AQQRLAAEQELIRLRAETEQGEQQRQLLEEELARLQSEAAAATQKRQELEAELAKVRAEMEVLLASKARAEEESRSSSEK
1sjj.1.A   --------------------------------------------------------------------------------

Target     SKQRLEAEAGRFRELAEEAARLRALAEEAKRQRQLAEEDAARQRAEAERVLSEKLAAISEATRLKTEAEIALKEKEAENE
1sjj.1.A   --------------------------------------------------------------------------------

Target     RLRRLAEDEAFQRRRLEEQAAQHKADIEERLAQLRKASESELERQKGLVEDTLRQRRQVEEEILALKASFEKAAAGKAEL
1sjj.1.A   --------------------------------------------------------------------------------

Target     ELELGRIRGNAEDTLRSKEQAEQEAARQRQLAAEEERRRREAEERVQKSLAAEEEAARQRKAALEEVERLKAKVEEARRL
1sjj.1.A   --------------------------------------------------------------------------------

Target     RERAEHESVRQLQLAQEAAQKRLQAEEKAHAFAVQQKEQELQQTLQQEQSVLEKLRSEAEAARRAAEEAEEARERAEREA
1sjj.1.A   --------------------------------------------------------------------------------

Target     AQSRQRVEEAERLKQAAEEQAQAQAQAQAAAEKLRKEAEQEAARRAQAEQAALRQKQAADAEMEKHKKFAEQTLRQKAQV
1sjj.1.A   --------------------------------------------------------------------------------

Target     EQELTALRLKLEETDHQKSILDQELQRLKAEVTEAARQRSQVEEELFSVRVQMEELGKLKARIEAENRALILRDKDNTQR
1sjj.1.A   --------------------------------------------------------------------------------

Target     LLQEEAEKMKQVAEEAARLSVAAQEAARLRQLAEEDLAQQRALAEKMLKEKMQAVQEATRLKAEAELLQQQKELAQEQAR
1sjj.1.A   --------------------------------------------------------------------------------

Target     RLQEDKEQMAQQLAQETQGFQRTLETERQRQLEMSAEAERLRLRVAEMSRAQARAEEDAQRFRKQAEEIGAKLHRTELAT
1sjj.1.A   --------------------------------------------------------------------------------

Target     QEKVTLVQTLETQRQQSDRDADRLREAIAELEREKDKLKKEAELLQLKSEEMQTVQQEQLLQETQALQQSFLSEKDSLLQ
1sjj.1.A   --------------------------------------------------------------------------------

Target     RERFIEEEKAKLERLFQDEVAKAQKLREEQQRQQQMQQEKQQLLASMEEARRRQHEAEEGVRRKQEELQLLEQQRQQQE
1sjj.1.A   --------------------------------------------------------------------------------

Target     QLLAEENRRLRERLEHLEEEHRAALAHSEEITAAQAAATRALPNGQDAADGPAAEPEHAFEGLRQKVPAQQLQEAGILST
1sjj.1.A   --------------------------------------------------------------------------------

Target     EELQRLAQGHTTVAELTQREDVRRYLQGHSSIAGLLLKPANEKLTIYAALRRQLLSPGTALILLEAQAASGFLLDPVRNR
1sjj.1.A   --------------------------------------------------------------------------------

Target     RLTVTEAVKEGVVGPELHHKLLSAERAVTGYKDPYTGEKISLFQAMKKDLIVREHGIRLLEAQIATGGVIDPVHSHRVPV
1sjj.1.A   --------------------------------------------------------------------------------

Target     DVAYHRGYFDEEMNRVLQDPSDDTKGFFDPNTHENLTYLQLLERCVEDPETGLRLLPLTDQAAKGGELVYTDSEARDVFE
1sjj.1.A   --------------------------------------------------------------------------------

Target     KATVSAPFGKFQGKTVTIWELINSEYFTAEQRRDLLRQFRTGKVTVEKIIKIVITVIEEHEQKGQLCFQGLRALVPAAEL
1sjj.1.A   --------------------------------------------------------------------------------

Target     LESGIIDWDLFRQLQLGERSVQEVAEVEGVRRALRGSGVIAGVWLEEARQKLSIYEALKKELLQPEAAVALLEAQAGTGH
1sjj.1.A   --------------------------------------------------------------------------------

Target     VIDPATSARLTVDEAVRAGLVGPELHEKLLSAEKAVTGYKDPYSGQSVSLFQALKKGLIPREQGLRLLDAQLSTGGTVDP
1sjj.1.A   --------------------------------------------------------------------------------

Target     SKSHRLPLDVACARGYLDEETSTALSAPRDDAKTYYDPRTWELATYSQLQQQCRPDPLTGLSLLPLSEEAARARQQELYS
1sjj.1.A   --------------------------------------------------------------------------------
```

```
Target    EVQAREAFQKATVEVPVGSFQGRAVTIWELINSEYFTAEQRQELLRQFRTGKVTVEKIIKIVITIVEEVETTRRERLSFS
1sjj.1.A  --------------------------------------------------------------------------------

Target    GLRAPVPASELLAAGILSSSQFEQLKDGKTSVKDLSELSSLQTLLQGSGCLAGIYLEESKEKVTIYEAMRRGLLRPSTAI
1sjj.1.A  --------------------------------------------------------------------------------

Target    LLLEAQAATGFLVDPVRNQRLYVHEAVKAGVVGPELHEKLLSAEKAVTGYKDPYSGSTISLFQAMKKGLVVREHGIRLLE
1sjj.1.A  --------------------------------------------------------------------------------

Target    AQIATGGIIDPVHSHRVPVDVACQRGYFDEEMNRVLEDPSDDTKGFFDPNTHENLTYRQLLERCVEDPETGLRLLPLKGP
1sjj.1.A  --------------------------------------------------------------------------------

Target    EKAEVVETTRVYTEEETRRAFEETQIDIPGGGSHGGSTMSLWEVMQSDLIPEEQRAQLMADFQAGRVTKERMIIIIIEII
1sjj.1.A  --------------------------------------------------------------------------------

Target    EKTEIVRQQNLASYDYVRRRLTAEDLYEARVISRESYSLLREGTRSLREVLEAESAWRYLYGTGCVAGVYLPGSRQTLPI
1sjj.1.A  --------------------------------------------------------------------------------

Target    YQALKKGLLSAEVARLLLEAQAATGFLLDPVKGERLTVDEAVRKGLVGPELHDRLLSAERAVTGYRDPYTEQTISLFQAM
1sjj.1.A  --------------------------------------------------------------------------------

Target    KKDLIPAEEALRLLDAQLATGGIVDPRLGFHLPLEVAYQRGYLNKDTHDQLSEPSEVRSYVDPSTDERLSYTQLLRRCRR
1sjj.1.A  --------------------------------------------------------------------------------

Target    DETSGLFLLPLSEARKLTFRGLRKQITVEELVRSHVMDEATAQRLQEGLTSIEEVSKNLQKFLEGTSSIAGVLVDATKER
1sjj.1.A  --------------------------------------------------------------------------------

Target    LSVYQAMKKGIIRPGTAFELLEAQAATGYVIDPIKGLKLTVEEAVRMGIVGPEFKDKLLSAERAVTGYKDPYSGKLISLF
1sjj.1.A  --------------------------------------------------------------------------------

Target    QAMKKGLILKDHGIRLLEAQIATGGIIDPEESHRLPVDVAYQRGLFDEEMNEILTDPSDDTKGFFDPNTEENLTYLQLME
1sjj.1.A  --------------------------------------------------------------------------------

Target    RCVTDPQTGLRLLPLKEKKRERKTSSKSSVRKRRVVIVDPETGKEMSVYEAYRKGLIDHQTYLELSEQECEWEEITISSS
1sjj.1.A  --------------------------------------------------------------------------------

Target    DGVVKSMIIDRRSGRQYDIDEAIAKSLIDRSALDQYRAGTLSITEFADMLSGNAGGFRSRSSSVGSSSSYPISPAVSRTQ
1sjj.1.A  --------------------------------------------------------------------------------

Target    LASWSDPTEETGPVAGILDTETLEKVSITEAMHRNLVDNITGQRLLEAQACTGGIIDPNTGERFPVTDAVNKGLVDKIMV
1sjj.1.A  --------------------------------------------------------------------------------

Target    DRINLAQKAFCGFEDPRTKTKMSAAQALKKGWLYYEAGQRFLEVQYLTGGLIEPDTPGRVPLDEALQRGTVDARTAQKLR
1sjj.1.A  --------------------------------------------------------------------------------

Target    DVSAYSKYLTCPKTKLKISYKDALDRSMVEEGTGLRLLEAAAQSSKGYYSPYSVSGSGSTTGSRSGSRTGSRAGSRRGSF
1sjj.1.A  --------------------------------------------------------------------------------

Target    DATGSGFSMTFSSSSYSSSGYGRRYASGPTSSLGGPESAAA
1sjj.1.A  -----------------------------------------
```

Model #07	File	Built with	Oligo-State	Ligands	GMQE	QMEANDisCo Global
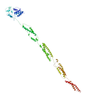	PDB	ProMod3 3.3.0	monomer (matching prediction)	None	0.00	0.47 ± 0.05

Template	Seq Identity	Oligo-state	QSQE	Found by	Method	Resolution	Seq Similarity	Range	Coverage	Description
1sjj.1.B	12.54	homo-dimer	0.00	HHblits	2DX	-	0.27	187 - 806	0.13	actinin

The template contained no ligands.

```
Target    MSGEDQEVRAVVEDGSNGGSGSPSPGDTLPWNLEKTQRSRRGGGGPGGNGSVLDPAERAVIRIADERDRVQKKTFTKWVN
1sjj.1.B  --------------------------------------------------------------------------------
```

```
Target     KHLIKAQRHISDLYEDLRDGHNLISLLEVLSGDSLPREKGRMRFHKLQNVQIALDYLRHRQVKLVNIRNDDIADGNPKLT
1sjj.1.B   --------------------------------------------------------------------------------

Target     LGLIWTIILHFQISDIQVSGQSEDMTAKEKLLLWSQRMVEGYQGLRCDNFTSSWRDGRLFNAIIHRHKPMLIDMN--KVY
1sjj.1.B   --------------------------QRKTFTAWCNSHLRKA-GTQIENIEEDFRDGLKLMLLLEVISGERLAKPERGKM

Target     RQTNLENLDQAFSVAERDLGVT-RLLDPEDVDVPQPDEKSIITYVSSLYDAMPRVPDVQDGVKANELQLRWQEYRELVLL
1sjj.1.B   RVHKISNVNKALDFIA-SKGVKLVSIGAEEIVDGNV--KMTLGMIWTIILRFAIQDISVE-------------ETSAKEG

Target     LLQWIRAHTAAFE---ERRFPSSFEEIEILWCQFLKFKETELP----AKEADKNRSKGIYQSLEGAVQAGQLKVPPGY--
1sjj.1.B   LLLWYQRKTAPYKNVNIQNFHISWKDGLGFCALIHRHRPELIDYGKLRKDDPLTNLNTAFDVAEKYLDIPKMLDAEDIVG

Target     -----------HPLDVEKEWGKLHVAILEREKQLRSEFERLERLQRIVSKLQMEAGLCEEQLNQADALLQSDVRL---LA
1sjj.1.B   TARPDEKAIMTYVSSFYHAFSGAQKAETAA-NRICKVLAVNQENEQLMEDYEKLASDLLEWIRRTIPWLENRAPENTMQA

Target     AGKAPQRAGEVERDLDKADG----MIRLLFNDV-QALKD-GRHPQGEQMYRRVYRLHERLVAIRT---EYNLRLRGTPRH
1sjj.1.B   MQQKLEDFRDYRRLHKPPKVQEKCQLEINFNTLQTKLRLSNRPAFMPSEGKMVSDINNAWGGLEQAEKGYEEWLLNEIRR

Target     P----ELEDSTLRYLQDLLAWVEENQRRVDSAEWGV-DLPSVEAQLGSHRGLHQSVEEFRAKIERARTDEGQLS---PAT
1sjj.1.B   LERLDHLAEKFRQKASIHESWTDGKEAMLQQKDYETATLSEIKALLKKHEAFESDLAAHQDRVEQIAAIAQELNELDYYD

Target     RGAYRDCLGRLDLQYAKLLNSSKARLRSLES-----------LHGFVAAATKELMWLSEKEEEEVGFDWSERNSNMAAKK
1sjj.1.B   SPSVNARCQKICDQWDNLGALTQKRREALERTEKLLETIDQLYLEYAKRAAPFNNWMEGAMEDLQDTFIVHTIEEIQGLT

Target     EAYSALMRELELKEKKIKEI---QSTGDRLLREDH----PARPTVESFQAALQTQWSWMLQLCCCIEAHLKENTAY----
1sjj.1.B   TAHEQFKATLPDADKERQAILGIHNEVSKIVQTYHVNMAGTNPYTTITPQEINGKWEHVRQLVPRRDQALMEEHARQQQN

Target     ----FQFFSDVREAEEQLRKLQETLHRKYTCDRSITVTRLEDLLQDAQDEKDQLNEYRGHLSGLAKRAKAIVQLTPRNPT
1sjj.1.B   ERLRKQFGAQANVIGPWIQTKMEEIGRISI----EMHGTLEDQLNHLRQYEKSIVNYKPKIDQLEGDHQQIQEAL-----

Target     QPTRGRVPLLAVCDYKQVEATVHKGDECQMLGPAQPFHWKVLSSSGSEAAVPSVCFLVPPPNQEALEAVARLEAQHQALV
1sjj.1.B   --------------------------------------------------------------------------------

Target     TLWHQLHTDMKSLLAWQSLSRDVQLIRSWSLVTFRTLKPEEQRQALRSLELHYQAFLRDSQDAGGFGPEDRLQAEREYGS
1sjj.1.B   --------------------------------------------------------------------------------

Target     CSRHYQQLLQSLEQGEQEESRCQRCISELKDIRLQLEACETRTVHRLRLPLDKEPARECAQRIAEQQKAQAEVEGLGKGV
1sjj.1.B   --------------------------------------------------------------------------------

Target     ARLSAEAEKVLALPEPSPAAPTLRSELELTLGKLEQVRSLSAIYLEKLKTISLVIRSTQGAEEALRAHEEQLKEAQAVPA
1sjj.1.B   --------------------------------------------------------------------------------

Target     ALPELEATKAAMKKLRAQAEAQQPVFDALRDELRGAQEVGERLQQRHGERDVEVERWRERVTQLLERWQAVLAQTDVRQR
1sjj.1.B   --------------------------------------------------------------------------------

Target     ELEQLGRQLRYYRESADPLGAWLQDARRRQEQIQAVPLADSQAVREQLRQEKALLEEIERHAEKVEECQRFAKQYINAIK
1sjj.1.B   --------------------------------------------------------------------------------

Target     DYELQLVTYKAQLEPVASPAKKPKVQSGSESVIQEYVDLRTRYSELSTLTSQYIRFISETLRRMEEEERLAEQQRAEERE
1sjj.1.B   --------------------------------------------------------------------------------

Target     RLAEVEAALEKQRQLAEAHAQAKAQAEREAQELQRRMQEEVARREEVAVDAQQQKRSIQEELQQLRQSSEAEIQAKARQV
1sjj.1.B   --------------------------------------------------------------------------------

Target     EAAERSRLRIEEEIRVVRLQLETTERQRGGAEGELQALRARAEEAEAQKRQAQEEAERLRRQVQEETQRKRQAEAELGLR
1sjj.1.B   --------------------------------------------------------------------------------

Target     VKAEAEAAREKQRALQALEELRLQAEEAERRLRQAEAERARQVQVALETAQRSAQAELQSKHASFAEKTAQLERTLEEEH
1sjj.1.B   --------------------------------------------------------------------------------

Target     VTVVQLREEATRREQQQAEAERAREEAERELERWQLKANEALRLRLQAEEVAQQKSLAQAEAEKQKEAAEREARRRGKAE
1sjj.1.B   --------------------------------------------------------------------------------

Target     EQAVRQRELAEQELERQRQLAEGTAQQRLAAEQELIRLRAETEQGEQQRQLLEEELARLQSEAAAATQKRQELEAELAKV
1sjj.1.B   --------------------------------------------------------------------------------

Target     RAEMEVLLASKARAEEESRSSSEKSKQRLEAEAGRFRELAEEAARLRALAEEAKRQRQLAEEDAARQRAEAERVLSEKLA
1sjj.1.B   --------------------------------------------------------------------------------

Target     AISEATRLKTEAEIALKEKEAENERLRRLAEDEAFQRRRLEEQAAQHKADIEERLAQLRKASESELERQKGLVEDTLRQR
1sjj.1.B   --------------------------------------------------------------------------------

Target     RQVEEEILALKASFEKAAAGKAELELELGRIRGNAEDTLRSKEQAEQEAARQRQLAAEEERRRREAEERVQKSLAAEEEA
```

```
1sjj.1.B    --------------------------------------------------------------------------------

Target      ARQRKAALEEVERLKAKVEEARRLRERAEHESVRQLQLAQEAAQKRLQAEEKAHAFAVQQKEQELQQTLQQEQSVLEKLR
1sjj.1.B    --------------------------------------------------------------------------------

Target      SEAEAARRAAEEAEEARERAEREAAQSRQRVEEAERLKQAAEEQAQAQAQAQAAAEKLRKEAEQEAARRAQAEQAALRQK
1sjj.1.B    --------------------------------------------------------------------------------

Target      QAADAEMEKHKKFAEQTLRQKAQVEQELTALRLKLEETDHQKSILDQELQRLKAEVTEAARQRSQVEEELFSVRVQMEEL
1sjj.1.B    --------------------------------------------------------------------------------

Target      GKLKARIEAENRALILRDKDNTQRLLQEEAEKMKQVAEEAARLSVAAQEAARLRQLAEEDLAQQRALAEKMLKEKMQAVQ
1sjj.1.B    --------------------------------------------------------------------------------

Target      EATRLKAEAELLQQQKELAQEQARRLQEDKEQMAQQLAQETQGFQRTLETERQRQLEMSAEAERLRLRVAEMSRAQARAE
1sjj.1.B    --------------------------------------------------------------------------------

Target      EDAQRFRKQAEEIGAKLHRTELATQEKVTLVQTLETQRQQSDRDADRLREAIAELEREKDKLKKEAELLQLKSEEMQTVQ
1sjj.1.B    --------------------------------------------------------------------------------

Target      QEQLLQETQALQQSFLSEKDSLLQRERFIEEEKAKLERLFQDEVAKAQKLREEQQRQQQMQQEKQQLLASMEEARRRQH
1sjj.1.B    --------------------------------------------------------------------------------

Target      EAEEGVRRKQEELQLLEQQRQQQEQLLAEENRRLRERLEHLEEEHRAALAHSEEITAAQAAATRALPNGQDAADGPAAEP
1sjj.1.B    --------------------------------------------------------------------------------

Target      EHAFEGLRQKVPAQQLQEAGILSTEELQRLAQGHTTVAELTQREDVRRYLQGHSSIAGLLLKPANEKLTIYAALRRQLLS
1sjj.1.B    --------------------------------------------------------------------------------

Target      PGTALILLEAQAASGFLLDPVRNRRLTVTEAVKEGVVGPELHHKLLSAERAVTGYKDPYTGEKISLFQAMKKDLIVREHG
1sjj.1.B    --------------------------------------------------------------------------------

Target      IRLLEAQIATGGVIDPVHSHRVPVDVAYHRGYFDEEMNRVLQDPSDDTKGFFDPNTHENLTYLQLLERCVEDPETGLRLL
1sjj.1.B    --------------------------------------------------------------------------------

Target      PLTDQAAKGGELVYTDSEARDVFEKATVSAPFGKFQGKTVTIWELINSEYFTAEQRRDLLRQFRTGKVTVEKIIKIVITV
1sjj.1.B    --------------------------------------------------------------------------------

Target      IEEHEQKGQLCFQGLRALVPAAELLESGIIDWDLFRQLQLGERSVQEVAEVEGVRRALRGSGVIAGVWLEEARQKLSIYE
1sjj.1.B    --------------------------------------------------------------------------------

Target      ALKKELLQPEAAVALLEAQAGTGHVIDPATSARLTVDEAVRAGLVGPELHEKLLSAEKAVTGYKDPYSGQSVSLFQALKK
1sjj.1.B    --------------------------------------------------------------------------------

Target      GLIPREQGLRLLDAQLSTGGTVDPSKSHRLPLDVACARGYLDEETSTALSAPRDDAKTYYDPRTWELATYSQLQQQCRPD
1sjj.1.B    --------------------------------------------------------------------------------

Target      PLTGLSLLPLSEEAARARQQELYSEVQAREAFQKATVEVPVGSFQGRAVTIWELINSEYFTAEQRQELLRQFRTGKVTVE
1sjj.1.B    --------------------------------------------------------------------------------

Target      KIIKIVITIVEEVETTRRERLSFSGLRAPVPASELLAAGILSSSQFEQLKDGKTSVKDLSELSSLQTLLQGSGCLAGIYL
1sjj.1.B    --------------------------------------------------------------------------------

Target      EESKEKVTIYEAMRRGLLRPSTAILLLEAQAATGFLVDPVRNQRLYVHEAVKAGVVGPELHEKLLSAEKAVTGYKDPYSG
1sjj.1.B    --------------------------------------------------------------------------------

Target      STISLFQAMKKGLVVREHGIRLLEAQIATGGIIDPVHSHRVPVDVACQRGYFDEEMNRVLEDPSDDTKGFFDPNTHENLT
1sjj.1.B    --------------------------------------------------------------------------------

Target      YRQLLERCVEDPETGLRLLPLKGPEKAEVVETTRVYTEEETRRAFEETQIDIPGGGSHGGSTMSLWEVMQSDLIPEEQRA
1sjj.1.B    --------------------------------------------------------------------------------

Target      QLMADFQAGRVTKERMIIIIIEIIEKTEIVRQQNLASYDYVRRRLTAEDLYEARVISRESYSLLREGTRSLREVLEAESA
1sjj.1.B    --------------------------------------------------------------------------------

Target      WRYLYGTGCVAGVYLPGSRQTLPIYQALKKGLLSAEVARLLLEAQAATGFLLDPVKGERLTVDEAVRKGLVGPELHDRLL
1sjj.1.B    --------------------------------------------------------------------------------

Target      SAERAVTGYRDPYTEQTISLFQAMKKDLIPAEEALRLLDAQLATGGIVDPRLGFHLPLEVAYQRGYLNKDTHDQLSEPSE
1sjj.1.B    --------------------------------------------------------------------------------

Target      VRSYVDPSTDERLSYTQLLRRCRRDETSGLFLLPLSEARKLTFRGLRKQITVEELVRSHVMDEATAQRLQEGLTSIEEVS
1sjj.1.B    --------------------------------------------------------------------------------
```

```
Target    KNLQKFLEGTSSIAGVLVDATKERLSVYQAMKKGIIRPGTAFELLEAQAATGYVIDPIKGLKLTVEEAVRMGIVGPEFKD
1sjj.1.B  --------------------------------------------------------------------------------

Target    KLLSAERAVTGYKDPYSGKLISLFQAMKKGLILKDHGIRLLEAQIATGGIIDPEESHRLPVDVAYQRGLFDEEMNEILTD
1sjj.1.B  --------------------------------------------------------------------------------

Target    PSDDTKGFFDPNTEENLTYLQLMERCVTDPQTGLRLLPLKEKKRERKTSSKSSVRKRRVVIVDPETGKEMSVYEAYRKGL
1sjj.1.B  --------------------------------------------------------------------------------

Target    IDHQTYLELSEQECEWEEITISSSDGVVKSMIIDRRSGRQYDIDEAIAKSLIDRSALDQYRAGTLSITEFADMLSGNAGG
1sjj.1.B  --------------------------------------------------------------------------------

Target    FRSRSSSVGSSSSYPISPAVSRTQLASWSDPTEETGPVAGILDTETLEKVSITEAMHRNLVDNITGQRLLEAQACTGGII
1sjj.1.B  --------------------------------------------------------------------------------

Target    DPNTGERFPVTDAVNKGLVDKIMVDRINLAQKAFCGFEDPRTKTKMSAAQALKKGWLYYEAGQRFLEVQYLTGGLIEPDT
1sjj.1.B  --------------------------------------------------------------------------------

Target    PGRVPLDEALQRGTVDARTAQKLRDVSAYSKYLTCPKTKLKISYKDALDRSMVEEGTGLRLLEAAAQSSKGYYSPYSVSG
1sjj.1.B  --------------------------------------------------------------------------------

Target    SGSTTGSRSGSRTGSRAGSRRGSFDATGSGFSMTFSSSSYSSSGYGRRYASGPTSSLGGPESAAA
1sjj.1.B  -----------------------------------------------------------------
```

Materials and Methods

Template Search

Template search with has been performed against the SWISS-MODEL template library (SMTL, last update: 2023-11-08, last included PDB release: 2023-11-03).

Template Selection

For each identified template, the template's quality has been predicted from features of the target-template alignment. The templates with the highest quality have then been selected for model building.

Model Building

Models are built based on the target-template alignment using ProMod3 (Studer et al.). Coordinates which are conserved between the target and the template are copied from the template to the model. Insertions and deletions are remodelled using a fragment library. Side chains are then rebuilt. Finally, the geometry of the resulting model is regularized by using a force field.

Model Quality Estimation

The global and per-residue model quality has been assessed using the QMEAN scoring function (Studer et al.).

Ligand Modelling

Ligands present in the template structure are transferred by homology to the model when the following criteria are met: (a) The ligands are annotated as biologically relevant in the template library, (b) the ligand is in contact with the model, (c) the ligand is not clashing with the protein, (d) the residues in contact with the ligand are conserved between the target and the template. If any of these four criteria is not satisfied, a certain ligand will not be included in the model. The model summary includes information on why and which ligand has not been included.

Oligomeric State Conservation

The quaternary structure annotation of the template is used to model the target sequence in its oligomeric form. The method (Bertoni et al.) is based on a supervised machine learning algorithm, Support Vector Machines (SVM), which combines interface conservation, structural clustering, and other template features to provide a quaternary structure quality estimate (QSQE). The QSQE score is a number between 0 and 1, reflecting the expected accuracy of the interchain contacts for a model built based a given alignment and template. Higher numbers indicate higher reliability. This complements the GMQE score which estimates the accuracy of the tertiary structure of the resulting model.

References

- **BLAST**
  Camacho, C., Coulouris, G., Avagyan, V., Ma, N., Papadopoulos, J., Bealer, K., Madden, T.L. BLAST+: architecture and applications. BMC Bioinformatics 10, 421-430 (2009).

- **HHblits**
  Steinegger, M., Meier, M., Mirdita, M., Vöhringer, H., Haunsberger, S. J., Söding, J. HH-suite3 for fast remote homology detection and deep protein annotation. BMC Bioinformatics 20, 473 (2019).

Table T1:

Primary amino acid sequence for which templates were searched and models were built.

```
MSGEDQEVRAVVEDGSNGGSGSPSPGDTLPWNLEKTQRSRRGGGGPGGNGSVLDPAERAVIRIADERDRVQKKTFTKWVNKHLIKAQRHISDLYEDLRDG
HNLISLLEVLSGDSLPREKGRMRFHKLQNVQIALDYLRHRQVKLVNIRNDDIADGNPKLTLGLIWTIILHFQISDIQVSGQSEDMTAKEKLLLWSQRMVE
GYQGLRCDNFTSSWRDGRLFNAIIHRHKPMLIDMNKVYRQTNLENLDQAFSVAERDLGVTRLLDPEDVDVPQPDEKSIITYVSSLYDAMPRVPDVQDGVK
ANELQLRWQEYRELVLLLLQWIRAHTAAFEERRFPSSFEEIEILWCQFLKFKETELPAKEADKNRSKGIYQSLEGAVQAGQLKVPPGYHPLDVEKEWGKL
HVAILEREKQLRSEFERLERLQRIVSKLQMEAGLCEEQLNQADALLQSDVRLLAAGKAPQRAGEVERDLDKADGMIRLLFNDVQALKDGRHPQGEQMYRR
VYRLHERLVAIRTEYNLRLRGTPRHPELEDSTLRYLQDLLAWVEENQRRVDSAEWGVDLPSVEAQLGSHRGLHQSVEEFRAKIERARTDEGQLSPATRGA
YRDCLGRLDLQYAKLLNSSKARLRSLESLHGFVAAATKELMWLSEKEEEEVGFDWSERNSNMAAKKEAYSALMRELELKEKKIKEIQSTGDRLLREDHPA
RPTVESFQAALQTQWSWMLQLCCCIEAHLKENTAYFQFFSDVREAEEQLRKLQETLHRKYTCDRSITVTRLEDLLQDAQDEKDQLNEYRGHLSGLAKRAK
AIVQLTPRNPTQPTRGRVPLLAVCDYKQVEATVHKGDECQMLGPAQPFHWKVLSSSGSEAAVPSVCFLVPPPNQEALEAVARLEAQHQALVTLWHQLHTD
MKSLLAWQSLSRDVQLIRSWSLVTFRTLKPEEQRQALRSLELHYQAFLRDSQDAGGFGPEDRLQAEREYGSCSRHYQQLLQSLEQGEQEESRCQRCISEL
KDIRLQLEACETRTVHRLRLPLDKEPARECAQRIAEQQKAQAEVEGLGKGVARLSAEAEKVLALPEPSPAAPTLRSELELTLGKLEQVRSLSAIYLEKLK
TISLVIRSTQGAEEALRAHEEQLKEAQAVPAALPELEATKAAMKKLRAQAEAQQPVFDALRDELRGAQEVGERLQQRHGERDVEVERWRERVTQLLERWQ
AVLAQTDVRQRELEQLGRQLRYYRESADPLGAWLQDARRRQEQIQAVPLADSQAVREQLRQEKALLEEIERHAEKVEECQRFAKQYINAIKDYELQLVTY
KAQLEPVASPAKKPKVQSGSESVIQEYVDLRTRYSELSTLTSQYIRFISETLRRMEEEERLAEQQRAEERERLAEVEAALEKQRQLAEAHAQAKAQAERE
AQELQRRMQEEVARREEVAVDAQQQKRSIQEELQQLRQSSEAEIQAKARQVEAAERSRLRIEEEIRVVRLQLETTERQRGGAEGELQALRARAEEAEAQK
RQAQEEAERLRRQVQEETQRKRQAEAELGLRVKAEAEAAREKQRALQALEELRLQAEEAERRLRQAEAERARQVQVALETAQRSAQAELQSKHASFAEKT
AQLERTLEEEHVTVVQLREEATRREQQQAEAERAREEAERELERWQLKANEALRLRLQAEEVAQQKSLAQAEAEKQKEAAEREARRRGKAEEQAVRQREL
AEQELERQRQLAEGTAQQRLAAEQELIRLRAETEQGEQQRQLLEEELARLQSEAAAATQKRQELEAELAKVRAEMEVLLASKARAEEESRSSSEKSKQRL
EAEAGRFRELAEEAARLRALAEEAKRQRQLAEEDAARQRAEAERVLSEKLAAISEATRLKTEAEIALKEKEAENERLRRLAEDEAFQRRRLEEQAAQHKA
DIEERLAQLRKASESELERQKGLVEDTLRQRRQVEEEILALKASFEKAAAGKAELELELGRIRGNAEDTLRSKEQAEQEAARQRQLAAEEERRRREAEER
VQKSLAAEEEAARQRKAALEEVERLKAKVEEARRLRERAEHESVRQLQLAQEAAQKRLQAEEKAHAFAVQQKEQELQQTLQQEQSVLEKLRSEAEAARRA
AEEAEEARERAEREAAQSRQRVEEAERLKQAAEEQAQAQAQAQAAAEKLRKEAEQEAARRAQAEQAALRQKQAADAEMEKHKKFAEQTLRQKAQVEQELT
ALRLKLEETDHQKSILDQELQRLKAEVTEAARQRSQVEEELFSVRVQMEELGKLKARIEAENRALILRDKDNTQRLLQEEAEKMKQVAEEAARLSVAAQE
AARLRQLAEEDLAQQRALAEKMLKEKMQAVQEATRLKAEAELLQQQKELAQEQARRLQEDKEQMAQQLAQETQGFQRTLETERQRQLEMSAEAERLRLRV
AEMSRAQARAEEDAQRFRKQAEEIGAKLHRTELATQEKVTLVQTLETQRQQSDRDADRLREAIAELEREKDKLKKEAELLQLKSEEMQTVQQEQLLQETQ
ALQQSFLSEKDSLLQRERFIEEEKAKLERLFQDEVAKAQKLREEQQRQQQQMQQEKQQLLASMEEARRRQHEAEEGVRRKQEELQLLEQQRQQQEQLLAE
ENRRLRERLEHLEEEHRAALAHSEEITAAQAAATRALPNGQDAADGPAAEPEHAFEGLRQKVPAQQLQEAGILSTEELQRLAQGHTTVAELTQREDVRRY
LQGHSSIAGLLLKPANEKLTIYAALRRQLLSPGTALILLEAQAASGFLLDPVRNRRLTVTEAVKEGVVGPELHHKLLSAERAVTGYKDPYTGEKISLFQA
MKKDLIVREHGIRLLEAQIATGGVIDPVHSHRVPVDVAYHRGYFDEEMNRVLQDPSDDTKGFFDPNTHENLTYLQLLERCVEDPETGLRLLPLTDQAAKG
GELVYTDSEARDVFEKATVSAPFGKFQGKTVTIWELINSEYFTAEQRRDLLRQFRTGKVTVEKIIKIVITVIEEHEQKGQLCFQGLRALVPAAELLESGI
IDWDLFRQLQLGERSVQEVAEVEGVRRALRGSGVIAGVWLEEARQKLSIYEALKKELLQPEAAVALLEAQAGTGHVIDPATSARLTVDEAVRAGLVGPEL
HEKLLSAEKAVTGYKDPYSGQSVSLFQALKKGLIPREQGLRLLDAQLSTGGTVDPSKSHRLPLDVACARGYLDEETSTALSAPRDDAKTYYDPRTWELAT
YSQLQQQCRPDPLTGLSLLPLSEEAARARQQELYSEVQAREAFQKATVEVPVGSFQGRAVTIWELINSEYFTAEQRQELLRQFRTGKVTVEKIIKIVITI
VEEVETTRRERLSFSGLRAPVPASELLAAGILSSSQFEQLKDGKTSVKDLSELSSLQTLLQGSGCLAGIYLEESKEKVTIYEAMRRGLLRPSTAILLLEA
QAATGFLVDPVRNQRLYVHEAVKAGVVGPELHEKLLSAEKAVTGYKDPYSGSTISLFQAMKKGLVVREHGIRLLEAQIATGGIIDPVHSHRVPVDVACQR
GYFDEEMNRVLEDPSDDTKGFFDPNTHENLTYRQLLERCVEDPETGLRLLPLKGPEKAEVVETTRVYTEEETRRAFEETQIDIPGGGSHGGSTMSLWEVM
QSDLIPEEQRAQLMADFQAGRVTKERMIIIIIEIIEKTEIVRQQNLASYDYVRRRLTAEDLYEARVISRESYSLLREGTRSLREVLEAESAWRYLYGTGC
VAGVYLPGSRQTLPIYQALKKGLLSAEVARLLLEAQAATGFLLDPVKGERLTVDEAVRKGLVGPELHDRLLSAERAVTGYRDPYTEQTISLFQAMKKDLI
PAEEALRLLDAQLATGGIVDPRLGFHLPLEVAYQRGYLNKDTHDQLSEPSEVRSYVDPSTDERLSYTQLLRRCRRDETSGLFLLPLSEARKLTFRGLRKQ
ITVEELVRSHVMDEATAQRLQEGLTSIEEVSKNLQKFLEGTSSIAGVLVDATKERLSVYQAMKKGIIRPGTAFELLEAQAATGYVIDPIKGLKLTVEEAV
RMGIVGPEFKDKLLSAERAVTGYKDPYSGKLISLFQAMKKGLILKDHGIRLLEAQIATGGIIDPEESHRLPVDVAYQRGLFDEEMNEILTDPSDDTKGFF
DPNTEENLTYLQLMERCVTDPQTGLRLLPLKEKKRERKTSSKSSVRKRRVVIVDPETGKEMSVYEAYRKGLIDHQTYLELSEQECEWEEITISSSDGVVK
SMIIDRRSGRQYDIDEAIAKSLIDRSALDQYRAGTLSITEFADMLSGNAGGFRSRSSSVGSSSSYPISPAVSRTQLASWSDPTEETGPVAGILDTETLEK
VSITEAMHRNLVDNITGQRLLEAQACTGGIIDPNTGERFPVTDAVNKGLVDKIMVDRINLAQKAFCGFEDPRTKTKMSAAQALKKGWLYYEAGQRFLEVQ
YLTGGLIEPDTPGRVPLDEALQRGTVDARTAQKLRDVSAYSKYLTCPKTKLKISYKDALDRSMVEEGTGLRLLEAAAQSSKGYYSPYSVSGSGSTTGSRS
GSRTGSRAGSRRGSFDATGSGFSMTFSSSSYSSSGYGRRYASGPTSSLGGPESAAA
```

Table T2:

Template	Seq Identity	Oligo-state	QSQE	Found by	Method	Resolution	Seq Similarity	Coverage	Description
H9F0F3.1.A	94.23	monomer	-	AFDB search	AlphaFold v2	NA	0.59	0.26	Plectin isoform 1c
5dzz.1.A	45.12	monomer	-	BLAST	X-ray	2.60Å	0.40	0.10	Desmoplakin
8iah.1.2	38.97	monomer	-	BLAST	EM	NA	0.38	0.12	Spectrin beta chain
8iah.1.3	38.97	monomer	-	BLAST	EM	NA	0.38	0.12	Spectrin beta chain
5dzz.1.A	54.12	monomer	-	HHblits	X-ray	2.60Å	0.45	0.11	Desmoplakin
5dzz.1.A	51.14	monomer	-	HHblits	X-ray	2.60Å	0.45	0.11	Desmoplakin
1sjj.1.A	12.54	homo-dimer	-	HHblits	2DX	NA	0.27	0.13	actinin
1sjj.1.B	12.54	homo-dimer	-	HHblits	2DX	NA	0.27	0.13	actinin
5dzz.1.A	51.48	monomer	-	BLAST	X-ray	2.60Å	0.45	0.10	Desmoplakin
5dzz.1.A	47.12	monomer	-	HHblits	X-ray	2.60Å	0.42	0.11	Desmoplakin
5dzz.1.A	48.75	monomer	-	BLAST	X-ray	2.60Å	0.43	0.11	Desmoplakin
5dzz.1.A	38.07	monomer	-	HHblits	X-ray	2.60Å	0.38	0.11	Desmoplakin
5j1i.1.A	96.47	monomer	-	HHblits	X-ray	2.80Å	0.59	0.08	Plectin

Template	Seq Identity	Oligo-state	QSQE	Found by	Method	Resolution	Seq Similarity	Coverage	Description
8iah.1.1	38.97	monomer	-	BLAST	EM	NA	0.38	0.12	Spectrin beta chain
5j1i.1.A	96.37	monomer	-	BLAST	X-ray	2.80Å	0.59	0.08	Plectin
4z6g.1.A	66.86	monomer	-	HHblits	X-ray	2.65Å	0.51	0.08	Microtubule-actin cross-linking factor 1, isoforms 1/2/3/5
6sl7.1.A	27.80	homo-dimer	-	HHblits	X-ray	3.30Å	0.35	0.09	Calponin homology domain protein putative
3f7p.1.A	96.93	monomer	-	HHblits	X-ray	2.75Å	0.60	0.06	Plectin-1
7ank.1.A	12.69	monomer	-	HHblits	X-ray	3.20Å	0.27	0.10	Alpha-actinin-2
1hci.1.A	14.35	homo-dimer	-	HHblits	X-ray	2.80Å	0.28	0.09	ALPHA-ACTININ 2
7a8u.1.A	14.35	homo-dimer	-	HHblits	X-ray	3.80Å	0.28	0.09	Alpha-actinin-2
7a8t.1.A	14.35	homo-dimer	-	HHblits	X-ray	2.69Å	0.28	0.09	Alpha-actinin-2
6sl2.1.A	29.82	homo-dimer	-	HHblits	X-ray	3.10Å	0.36	0.08	Calponin homology domain protein putative
6sl3.1.A	29.82	homo-dimer	-	HHblits	X-ray	3.10Å	0.36	0.08	Calponin homology domain protein putative
4d1e.1.A	12.65	homo-dimer	-	HHblits	X-ray	3.50Å	0.26	0.09	ALPHA-ACTININ-2
3pe0.1.A	88.53	monomer	-	HHblits	X-ray	2.95Å	0.58	0.06	Plectin
8iah.1.Z	15.20	monomer	-	HHblits	EM	NA	0.28	0.22	Spectrin beta chain
8iah.1.Y	23.78	monomer	-	HHblits	EM	NA	0.32	0.25	Spectrin beta chain
8iah.1.X	23.78	monomer	-	HHblits	EM	NA	0.32	0.25	Spectrin beta chain
8iah.1.W	23.78	monomer	-	HHblits	EM	NA	0.32	0.25	Spectrin beta chain
8iah.1.Z	23.78	monomer	-	HHblits	EM	NA	0.32	0.25	Spectrin beta chain
8iah.1.0	23.78	monomer	-	HHblits	EM	NA	0.32	0.25	Spectrin beta chain
8iah.1.1	23.78	monomer	-	HHblits	EM	NA	0.32	0.25	Spectrin beta chain
8iah.1.2	23.78	monomer	-	HHblits	EM	NA	0.32	0.25	Spectrin beta chain
8iah.1.3	23.78	monomer	-	HHblits	EM	NA	0.32	0.25	Spectrin beta chain
8iah.1.Y	15.20	monomer	-	HHblits	EM	NA	0.28	0.22	Spectrin beta chain
8iah.1.X	15.20	monomer	-	HHblits	EM	NA	0.28	0.22	Spectrin beta chain
8iah.1.W	15.20	monomer	-	HHblits	EM	NA	0.28	0.22	Spectrin beta chain
8iah.1.0	38.97	monomer	-	BLAST	EM	NA	0.38	0.12	Spectrin beta chain
8iah.1.0	15.20	monomer	-	HHblits	EM	NA	0.28	0.22	Spectrin beta chain
8iah.1.1	15.20	monomer	-	HHblits	EM	NA	0.28	0.22	Spectrin beta chain
8iah.1.2	15.20	monomer	-	HHblits	EM	NA	0.28	0.22	Spectrin beta chain
8iah.1.3	15.20	monomer	-	HHblits	EM	NA	0.28	0.22	Spectrin beta chain
4d1e.1.A	25.11	homo-dimer	-	HHblits	X-ray	3.50Å	0.33	0.15	ALPHA-ACTININ-2
1sjj.1.A	26.16	homo-dimer	-	HHblits	2DX	NA	0.33	0.15	actinin
1sjj.1.B	26.16	homo-dimer	-	HHblits	2DX	NA	0.33	0.15	actinin
8iah.1.Y	38.97	monomer	-	BLAST	EM	NA	0.38	0.12	Spectrin beta chain
8iah.1.X	38.97	monomer	-	BLAST	EM	NA	0.38	0.12	Spectrin beta chain
8iah.1.W	38.97	monomer	-	BLAST	EM	NA	0.38	0.12	Spectrin beta chain
8iah.1.Z	38.97	monomer	-	BLAST	EM	NA	0.38	0.12	Spectrin beta chain

The table above shows the top 50 filtered templates. A further 1,277 templates were found which were considered to be less suitable for modelling than the filtered list.
1aa2.1.A, 1aj3.1.A, 1aoa.1.A, 1aww.1.A, 1awx.1.A, 1b07.1.A, 1bhd.1.A, 1bhd.2.A, 1bkr.1.A, 1c1g.1.A, 1c1g.1.B, 1c1g.2.A, 1c1g.2.B, 1csk.1.A, 1csk.2.A, 1cun.1.A, 1cun.1.B, 1d7m.1.A, 1d7m.1.B, 1dxx.1.A, 1fmk.1.A, 1g83.1.A, 1g83.2.A, 1g8x.1.A, 1g8x.1.B, 1gl5.1.A, 1gri.1.A, 1gri.1.B, 1h67.1.A, 1hci.1.A, 1k9a.1.A, 1k9a.2.A, 1k9a.3.A, 1k9a.5.A, 1k9a.6.A, 1ksw.1.A, 1lck.1.A, 1lm5.1.A, 1lm5.1.B, 1lm7.1.A, 1m3a.1.A, 1m3b.1.A, 1mb8.1.A, 1mv3.1.A, 1ng2.1.A, 1opk.1.A, 1opl.1.A, 1owa.1.A, 1p2x.1.A, 1p5s.1.A, 1pa7.1.A, 1pxy.1.A, 1pxy.2.A, 1qag.1.A, 1qag.2.A, 1qcf.1.A, 1qly.1.A, 1quu.1.A, 1rt8.1.A, 1s1n.1.A, 1s35.1.A, 1sh5.1.A, 1sh5.1.B, 1sh6.1.A, 1sjj.1.A, 1sjj.1.B, 1tjt.1.A, 1u4q.1.A, 1u4q.2.A, 1u5p.1.A, 1uec.1.A, 1ueg.1.A, 1ujo.1.A, 1v5k.1.A, 1vka.1.A, 1wfw.1.A, 1wjo.1.A, 1wku.1.A, 1wlx.1.A, 1wxt.1.A, 1wyl.1.A, 1wym.1.A, 1wyn.1.A, 1wyo.1.A, 1wyp.1.A, 1wyq.1.A, 1wyr.1.A, 1x6g.1.A, 1x79.1.B, 1x79.1.C, 1y57.1.A, 1ywo.1.A, 1ywp.1.A, 2abl.1.A, 2b9c.1.A, 2b9c.1.B,

2c0i.1.A, 2c0o.1.A, 2c0o.2.A, 2c0t.2.A, 2d3e.1.A, 2d3e.1.B, 2d85.1.A, 2d86.1.A, 2d87.1.A, 2d88.1.A, 2d89.1.A, 2dfs.1.A, 2dfs.1.H, 2dk9.1.A, 2dx1.1.A, 2dyb.1.A, 2dyb.2.A, 2e7s.1.A, 2e7s.1.B, 2e7s.10.A, 2e7s.10.B, 2e7s.2.A, 2e7s.2.B, 2e7s.3.A, 2e7s.3.B, 2e7s.4.A, 2e7s.4.B, 2e7s.5.A, 2e7s.5.B, 2e7s.6.A, 2e7s.6.B, 2e7s.7.A, 2e7s.7.B, 2e7s.8.A, 2e7s.8.B, 2e7s.9.A, 2e7s.9.B, 2e9k.1.A, 2ee7.1.A, 2efr.1.A, 2efr.1.B, 2efs.1.A, 2efs.1.B, 2egc.1.A, 2ege.1.A, 2elb.1.A, 2epd.1.A, 2eqb.1.B, 2eqb.1.C, 2eyi.1.A, 2eyn.1.A, 2eyw.1.A, 2eyz.1.A, 2fo0.1.A, 2fxm.1.A, 2fxm.1.B, 2fxo.1.A, 2fxo.1.B, 2fxo.2.A, 2fxo.2.B, 2h8h.1.A, 2i1j.1.A, 2i1k.1.A, 2iak.1.A, 2j6k.1.A, 2j7i.1.A, 2jv9.1.A, 2k2r.1.A, 2k3s.1.A, 2k79.1.A, 2k7a.1.A, 2kr3.1.A, 2kym.1.A, 2l3g.1.A, 2l3s.1.A, 2lmj.1.A, 2lqn.1.A, 2lqw.1.A, 2lx7.1.A, 2n03.1.A, 2ocy.1.A, 2ocy.1.B, 2odu.1.A, 2odv.1.A, 2oto.1.A, 2oto.1.B, 2oto.2.A, 2oto.2.B, 2ptk.1.A, 2pz1.1.A, 2q13.1.A, 2qjz.1.A, 2qjz.2.A, 2r0o.1.A, 2r0o.2.A, 2r8u.1.A, 2rn8.1.A, 2rna.1.A, 2rqr.1.A, 2rqv.1.A, 2rqw.1.A, 2rr8.1.A, 2spc.1.A, 2spc.1.B, 2src.1.A, 2v66.1.A, 2v66.1.B, 2v66.1.C, 2v66.1.D, 2v71.1.A, 2v71.1.B, 2vzc.1.A, 2vzd.1.A, 2vzd.2.A, 2vzg.1.B, 2w4u.1.K, 2w4u.1.L, 2w4u.1.M, 2wa5.1.A, 2wa6.1.A, 2wa7.1.A, 2wfn.1.A, 2y3a.1.B, 2yrn.1.A, 2z0o.1.A, 3a98.1.A, 3a98.2.A, 3byh.1.B, 3co1.1.A, 3dtp.1.A, 3dtp.1.B, 3edu.1.A, 3edv.1.A, 3edv.1.B, 3f31.1.A, 3f31.1.B, 3f57.1.A, 3f7p.1.A, 3fb2.1.A, 3fb2.2.A, 3fer.1.A, 3fer.2.A, 3fer.4.A, 3hoc.1.A, 3hop.1.A, 3i6x.1.A, 3jak.1.C, 3jax.1.A, 3jax.1.B, 3jbh.1.A, 3jbh.1.B, 3jv3.1.A, 3kbt.1.A, 3kbt.2.A, 3kbu.1.A, 3kbu.2.A, 3ky9.1.A, 3lbx.1.A, 3lbx.1.B, 3lue.1.K, 3nhn.1.A, 3o0z.1.A, 3o0z.1.B, 3o0z.2.A, 3o0z.2.B, 3oja.1.B, 3pdy.1.A, 3pdy.2.A, 3pe0.1.A, 3plt.1.A, 3plt.1.B, 3plt.2.A, 3r6n.1.A, 3r6n.2.A, 3s84.1.A, 3s84.1.B, 3tnu.1.B, 3u1c.1.A, 3u1c.2.A, 3u59.1.A, 3u59.1.B, 3u59.2.A, 3u59.3.A, 3uf4.1.A, 3uul.1.A, 3uum.1.A, 3uum.2.A, 3uun.1.A, 3uun.2.A, 3vkg.1.A, 3vkg.1.B, 3vkh.1.A, 3vkh.2.A, 3vry.2.A, 3vs3.2.A, 3vs7.1.A, 3vs7.2.A, 3wuq.1.A, 4a7f.1.B, 4a7f.1.H, 4b7l.1.A, 4b7l.2.A, 4d1e.1.A, 4d8k.1.A, 4edl.1.A, 4edl.2.A, 4edl.3.A, 4edl.4.A, 4edl.5.A, 4edm.1.A, 4edm.2.A, 4edn.1.A, 4edn.2.A, 4edn.5.A, 4gkw.1.A, 4gkw.1.B, 4h5y.1.A, 4h8s.1.A, 4h8s.1.B, 4h8s.2.A, 4h8s.2.B, 4hpq.1.C, 4jio.1.A, 4jio.2.A, 4l3i.1.A, 4l3i.1.B, 4l6y.1.A, 4l6y.1.B, 4lin.1.A, 4lin.1.B, 4lin.1.C, 4lin.2.A, 4lin.2.B, 4lin.2.C, 4lin.3.A, 4lin.3.B, 4lin.3.C, 4lin.4.A, 4lin.4.B, 4lin.4.C, 4ll8.1.B, 4ll8.1.C, 4mh6.1.A, 4mt6.1.A, 4p1w.1.C, 4p1w.1.F, 4pd3.1.A, 4pd3.2.A, 4q28.1.A, 4q57.1.B, 4q58.1.A, 4q58.2.A, 4q59.1.A, 4q59.2.A, 4qmd.1.A, 4qmd.2.A, 4rfx.1.A, 4rfx.1.B, 4rfx.2.A, 4rfx.2.B, 4rfx.3.A, 4rh7.1.A, 4rm8.1.A, 4rm8.2.A, 4tql.1.A, 4txi.1.A, 4txk.1.A, 4u5w.1.B, 4u5w.1.D, 4uer.1.a, 4uxv.1.A, 4xa1.1.A, 4xa1.1.B, 4xa1.2.A, 4xa1.2.B, 4xa3.1.A, 4xa3.1.B, 4xa6.1.A, 4xa6.1.B, 4xa6.2.A, 4xa6.2.B, 4xi2.1.A, 4z6g.1.A, 4zdw.1.B, 4zdw.1.C, 4zry.1.A, 5a36.1.A, 5a37.1.A, 5a38.1.A, 5a4b.1.A, 5a5t.1.A, 5ayh.1.A, 5bvr.1.A, 5c5b.1.B, 5cj0.1.A, 5cj0.1.B, 5cj1.1.A, 5cj1.1.B, 5cj1.2.A, 5cj1.2.B, 5cj1.3.A, 5cj1.3.B, 5cj1.4.A, 5cj1.4.B, 5dzz.1.A, 5gox.1.A, 5gox.1.B, 5h0g.1.A, 5i4e.1.A, 5ih2.1.A, 5ih2.2.A, 5ijn.1.F, 5ijn.1.G, 5ijn.1.H, 5ijn.1.L, 5ijn.1.R, 5j1f.1.A, 5j1f.2.A, 5j1g.1.A, 5j1h.1.A, 5j1h.2.A, 5j1i.1.A, 5j4o.1.A, 5jhf.1.C, 5jhf.3.C, 5l0o.1.A, 5l23.1.A, 5m6s.1.A, 5mo4.1.A, 5nl6.1.A, 5nl6.1.B, 5nl7.1.A, 5nl7.2.A, 5nug.1.A, 5tby.1.A, 5tby.1.B, 5to5.1.A, 5to5.1.B, 5to6.1.A, 5to6.1.B, 5to6.1.C, 5to6.1.D, 5to7.1.A, 5to7.1.B, 5to7.1.C, 5to7.1.D, 5tvb.1.A, 5tvb.1.B, 5ul6.1.A, 5wjb.1.A, 5wjb.1.B, 5wlq.1.A, 5wlz.1.A, 5wlz.1.B, 5wlz.1.C, 5wlz.1.D, 5xg2.1.A, 6amv.1.A, 6amw.1.A, 6anu.1.G, 6atv.1.A, 6b25.1.A, 6b26.1.A, 6b27.1.A, 6b27.2.A, 6b27.5.A, 6b28.1.A, 6d8c.1.A, 6djl.1.B, 6djl.2.A, 6djl.3.A, 6djl.4.A, 6ek8.1.A, 6el1.1.T, 6f1t.1.U, 6f1t.1.h, 6f3f.1.A, 6fec.1.A, 6fsa.1.A, 6fyy.1.g, 6gao.1.A, 6gao.1.B, 6gao.1.C, 6gap.1.A, 6gap.1.B, 6gap.1.C, 6gy6.1.B, 6gy7.1.A, 6gy7.2.A, 6gy7.3.A, 6gy7.4.A, 6h2x.1.A, 6ian.1.A, 6ian.1.B, 6ian.2.A, 6ian.2.B, 6ici.1.A, 6ign.1.A, 6ixv.1.A, 6ixv.2.A, 6ixv.3.A, 6ixv.4.A, 6jlb.1.A, 6jlb.1.B, 6jlb.1.C, 6jlb.1.D, 6kn7.1.P, 6kn7.1.Q, 6kn8.1.P, 6kn8.1.Q, 6l5h.1.A, 6l5h.1.B, 6l5j.1.A, 6l5j.1.B, 6l5j.2.A, 6l5j.2.B, 6m3p.1.B, 6m3q.1.B, 6m3r.1.B, 6m5g.1.F, 6mi3.1.A, 6mi3.1.B, 6mi4.1.A, 6mi4.1.B, 6mib.1.B, 6nf1.1.A, 6o31.1.A, 6oa6.1.A, 6pf2.1.A, 6pf2.1.B, 6pfp.1.A, 6pfp.1.B, 6pfp.2.A, 6pfp.2.B, 6rlb.1.A, 6rlb.1.B, 6sc2.1.A, 6sc2.1.B, 6sdf.1.A, 6sdf.2.A, 6sl2.1.A, 6sl3.1.A, 6sl7.1.A, 6snz.1.A, 6snz.1.B, 6snz.2.A, 6snz.2.B, 6swt.1.A, 6u42.439.A, 6u42.440.A, 6u42.441.A, 6u42.442.A, 6up6.1.A, 6upn.1.A, 6uui.1.B, 6uy7.1.A, 6uy8.1.A, 6uy8.2.A, 6uy9.1.A, 6vec.1.L, 6vzf.1.A, 6w2s.1.9, 6w2t.1.d, 6wg3.1.A, 6x5z.1.B, 6x5z.1.D, 6xe9.1.A, 6xe9.1.D, 6xf1.1.A, 6xf1.2.A, 6xf2.1.A, 6xf2.2.A, 6yam.1.g, 6ybd.1.C, 6yjd.1.A, 6yrl.1.A, 6yrl.1.B, 6yrl.1.C, 6yrl.1.D, 6yrn.1.A, 6yrn.1.B, 6yrn.1.C, 6yrn.1.D, 6yrn.2.A, 6yrn.2.B, 6yrn.2.C, 6yrn.2.D, 6ys4.1.A, 6ys4.1.B, 6ys4.2.A, 6ys4.2.B, 6ys4.3.A, 6ys4.3.B, 6ysy.1.A, 6yuf.1.C, 6yvd.1.C, 6yvu.1.A, 6yvv.1.A, 6z26.1.A, 6z26.1.B, 6z26.2.A, 6z26.2.B, 6z47.1.G, 6z47.1.H, 6z6f.1.C, 6z6f.1.D, 6z6h.1.C, 6z6h.1.D, 6z6o.1.C, 6z6o.1.D, 6z6p.1.C, 6z6p.1.D, 6zce.1.a, 6zmw.1.E, 6znm.1.H, 6znm.1.J, 6zp4.1.c, 6zsh.1.B, 6zsh.2.B, 6zu9.1.c, 6zvr.1.A, 6zvr.1.B, 6zvr.1.C, 6zvr.1.D, 6zvr.1.E, 6zvs.1.A, 6zvs.2.A, 6zvs.3.A, 6zvs.4.A, 6zvs.5.A, 6zvt.3.A, 6zvt.4.A, 6zvt.6.A, 6zw4.2.A, 6zw4.3.A, 6zw4.4.A, 6zw4.5.A, 6zw6.1.A, 6zw6.2.A, 6zw6.3.A, 6zw6.4.A, 6zw6.5.A, 6zw6.6.A, 6zw7.2.A, 6zw7.5.A, 6zw7.6.A, 6zw7.7.A, 6zyw.1.A, 6zyw.1.B, 6zyw.1.C, 7a09.1.c, 7a8t.1.A, 7a8u.1.A, 7abk.1.A, 7ank.1.A, 7ank.1.B, 7aw8.1.A, 7d9n.1.A, 7d9n.1.B, 7dl2.1.A, 7dl2.1.D, 7jh7.1.B, 7ju4.1.J, 7ju4.1.K, 7k58.1.A, 7k58.1.B, 7k58.1.D, 7k5b.1.A, 7k5b.1.B, 7k5b.1.C, 7kek.1.A, 7kek.1.B, 7kek.1.D, 7ko4.1.P, 7ko4.1.Q, 7ko5.1.P, 7ko5.1.Q, 7kog.1.A, 7kog.1.B, 7kzm.1.6, 7kzm.1.9, 7kzm.1.P, 7kzm.1.Q, 7kzo.1.P, 7mf3.1.A, 7mf3.1.B, 7mgm.1.A, 7moq.1.B, 7moq.1.C, 7moq.1.X, 7moq.1.Z, 7n61.7.A, 7n61.8.A, 7n85.1.E, 7n85.1.F, 7n85.1.G, 7n85.1.H, 7n85.1.I, 7n85.1.J, 7n85.1.K, 7n85.1.L, 7n85.1.M, 7n85.1.N, 7n85.1.O, 7n85.1.P, 7n9f.1.8, 7n9f.1.9, 7n9f.1.E, 7n9f.1.F, 7n9f.1.G, 7n9f.1.H, 7n9f.1.I, 7n9f.1.J, 7n9f.1.K, 7n9f.1.L, 7n9f.1.M, 7n9f.1.N, 7n9f.1.O, 7n9f.1.P, 7nep.1.L, 7nep.1.M, 7nsu.1.D, 7nyw.1.A, 7nyw.1.B, 7nyx.1.A, 7nyx.1.B, 7nyy.1.A, 7nyy.1.B, 7nyz.1.B, 7nz0.1.A, 7nz0.1.B, 7nz3.1.A, 7o3w.1.A, 7o3w.1.B, 7o3w.1.C, 7o3w.1.D, 7o3w.1.E, 7o3w.1.F, 7o3x.1.A, 7o3x.1.B, 7o3x.1.C, 7o3x.1.D, 7o3x.1.E, 7o3x.1.F, 7o3y.1.A, 7o3y.1.B, 7o3y.1.C, 7o3y.1.E, 7o3y.1.F, 7o3z.1.A, 7o3z.1.B, 7o3z.1.C, 7o3z.1.D, 7o3z.1.E, 7o3z.1.F, 7o40.1.A, 7o40.1.B, 7o40.1.C, 7o40.1.D, 7o40.1.E, 7o40.1.F, 7ogt.1.B, 7per.1.G, 7per.1.J, 7q2x.1.A, 7q2y.1.A, 7qp6.1.m, 7r3m.1.A, 7r5k.43.A, 7r5k.49.A, 7r5k.53.A, 7r5k.54.A, 7r5k.94.A, 7r5k.95.A, 7r94.1.F, 7r94.1.G, 7rro.12.A, 7rro.120.A, 7rro.121.A, 7rro.122.A, 7rro.123.A, 7rro.13.A, 7rro.14.A, 7rro.15.A, 7rro.153.A, 7rro.154.A, 7rro.156.A, 7rro.157.A, 7rro.158.A, 7rro.32.A, 7rro.33.A, 7rro.34.A, 7rro.36.A, 7rro.37.A, 7rro.38.A, 7rro.39.A, 7rro.56.A, 7rro.57.A, 7rro.58.A, 7rro.61.A, 7rro.62.A, 7rro.63.A, 7rro.79.A, 7rro.80.A, 7rro.81.A, 7rro.82.A, 7rro.83.A, 7rro.84.A, 7rro.85.A, 7rro.86.A, 7sj9.1.M, 7som.195.A, 7sqk.1.A, 7sx8.1.D, 7sx9.1.G, 7sxa.1.A, 7tdz.1.2, 7tdz.1.3, 7tdz.1.5, 7ung.12.A, 7ung.13.A, 7ung.14.A, 7ung.15.A, 7ung.31.A, 7ung.32.A, 7ung.34.A, 7ung.56.A, 7ung.57.A, 7ung.58.A, 7ung.59.A, 7ung.74.A, 7ung.75.A, 7ung.76.A, 7ung.77.A, 7ung.78.A, 7uti.1.Q, 7uti.1.R, 7utl.1.S, 7utl.1.T, 7vf2.1.C, 7vf2.1.D, 7vf5.1.B, 7vf5.1.C, 7vop.1.0, 7vop.1.1, 7vop.1.2, 7vop.1.3, 7vop.1.4, 7vop.1.5, 7w1m.1.A, 7w1m.1.B, 7wkk.1.G, 7wkk.1.I, 7wkk.1.J, 7wkk.1.L, 7wkk.1.U, 7wkk.1.X, 7woo.1.H, 7wot.1.F, 7wot.1.I, 7wot.1.J, 7wot.1.S, 7wot.1.V, 7wot.1.W, 7yg4.1.B, 7yg4.1.C, 7yqe.1.A, 7yqe.2.A, 7yxw.1.B, 7z8f.1.0, 7z8f.1.7, 7z8f.1.8, 7z8f.1.Z, 7z8f.1.f, 7z8f.1.g, 7z8k.1.H, 7z8k.1.I, 7zgx.1.A, 7zr1.1.C, 7zr1.1.D, 8ae1.1.A, 8at2.1.A, 8at3.1.A, 8auc.1.A, 8auc.2.A, 8bd7.1.G, 8bd7.1.I, 8bpo.1.m, 8bwy.1.A, 8bwy.1.B, 8bwy.1.C, 8bx8.1.A, 8bx8.1.B, 8bx8.1.C, 8dd0.1.G, 8dd0.1.H, 8dgo.1.A, 8dyu.1.A, 8dyv.1.A, 8dzz.1.A, 8efh.1.G, 8efh.1.H, 8efi.1.A, 8efi.1.G, 8enc.1.A, 8enc.1.H, 8fck.1.A, 8fcy.1.A, 8fd6.1.A, 8fdt.1.A, 8fed.1.C, 8g4l.10.A, 8g4l.100.A, 8g4l.101.A, 8g4l.102.A, 8g4l.104.A, 8g4l.105.A, 8g4l.106.A, 8g4l.111.A, 8g4l.112.A, 8g4l.118.A, 8g4l.17.A, 8g4l.18.A, 8g4l.19.A, 8g4l.2.A, 8g4l.20.A, 8g4l.21.A, 8g4l.22.A, 8g4l.23.A, 8g4l.24.A, 8g4l.30.A, 8g4l.35.A, 8g4l.36.A, 8g4l.46.A, 8g4l.47.A, 8g4l.48.A, 8g4l.49.A, 8g4l.5.A, 8g4l.50.A, 8g4l.51.A, 8g4l.58.A, 8g4l.59.A, 8g4l.6.A, 8g4l.60.A, 8g4l.61.A, 8g4l.62.A, 8g4l.63.A, 8g4l.64.A, 8g4l.65.A, 8g4l.7.A, 8g4l.70.A, 8g4l.71.A, 8g4l.76.A, 8g4l.77.A, 8g4l.8.A, 8g4l.83.A, 8g4l.87.A, 8g4l.88.A, 8g4l.89.A, 8g4l.9.A, 8g4l.90.A, 8g4l.91.A, 8g4l.92.A, 8g4l.99.A, 8glv.195.A, 8glv.215.A, 8glv.216.A, 8glv.217.A, 8glv.218.A, 8glv.346.A, 8glv.347.A, 8glv.357.A, 8glv.358.A, 8glv.359.A, 8glv.360.A, 8glv.420.A, 8glv.421.A, 8glv.442.A, 8glv.443.A, 8glv.455.A, 8glv.456.A, 8glv.481.A, 8glv.492.A, 8glv.498.A, 8glv.530.A, 8glv.531.A, 8glv.542.A, 8glv.554.A, 8glv.602.A,

8glv.603.A, 8glv.756.A, 8glv.844.A, 8glv.854.A, 8glv.855.A, 8glv.856.A, 8glv.891.A, 8glv.892.A, 8glv.902.A, 8glv.913.A, 8glv.951.A, 8glv.952.A, 8glv.953.A, 8glv.959.A, 8glv.960.A, 8glv.962.A, 8glv.963.A, 8glv.966.A, 8glv.974.A, 8gmb.1.A, 8i7o.1.A, 8i7o.11.A, 8i7o.18.A, 8i7o.19.A, 8i7o.20.A, 8i7o.27.A, 8i7o.28.A, 8i7o.31.A, 8i7o.47.A, 8i7o.48.A, 8i7o.49.A, 8i7o.56.A, 8i7o.57.A, 8i7o.59.A, 8i7o.91.A, 8i7o.92.A, 8i7r.107.A, 8i7r.108.A, 8i7r.109.A, 8i7r.110.A, 8i7r.111.A, 8i7r.112.A, 8i7r.113.A, 8i7r.114.A, 8i7r.115.A, 8i7r.128.A, 8i7r.130.A, 8i7r.132.A, 8i7r.133.A, 8i7r.134.A, 8i7r.135.A, 8i7r.136.A, 8i7r.137.A, 8i7r.138.A, 8i7r.139.A, 8i7r.140.A, 8i7r.19.A, 8i7r.2.A, 8i7r.20.A, 8i7r.21.A, 8i7r.213.A, 8i7r.214.A, 8i7r.215.A, 8i7r.216.A, 8i7r.22.A, 8i7r.24.A, 8i7r.25.A, 8i7r.26.A, 8i7r.27.A, 8i7r.398.A, 8i7r.4.A, 8i7r.411.A, 8i7r.42.A, 8i7r.43.A, 8i7r.44.A, 8i7r.45.A, 8i7r.46.A, 8i7r.47.A, 8i7r.48.A, 8i7r.49.A, 8i7r.5.A, 8i7r.62.A, 8i7r.63.A, 8i7r.64.A, 8i7r.65.A, 8i7r.69.A, 8i7r.70.A, 8i7r.71.A, 8i7r.73.A, 8i7r.74.A, 8i7r.75.A, 8iah.1.2, 8iah.1.4, 8iah.1.5, 8iah.1.6, 8iah.1.7, 8iai.1.1, 8iai.1.2, 8iai.1.T, 8iai.1.U, 8iai.1.W, 8iai.1.X, 8iyj.11.A, 8iyj.12.A, 8iyj.13.A, 8iyj.14.A, 8iyj.15.A, 8iyj.16.A, 8iyj.35.A, 8iyj.357.A, 8iyj.358.A, 8iyj.359.A, 8iyj.36.A, 8iyj.360.A, 8iyj.361.A, 8iyj.37.A, 8iyj.378.A, 8iyj.379.A, 8iyj.38.A, 8iyj.380.A, 8iyj.381.A, 8iyj.382.A, 8iyj.383.A, 8iyj.384.A, 8iyj.385.A, 8iyj.386.A, 8iyj.387.A, 8iyj.39.A, 8iyj.404.A, 8iyj.405.A, 8iyj.406.A, 8iyj.407.A, 8iyj.408.A, 8iyj.424.A, 8iyj.425.A, 8iyj.426.A, 8iyj.427.A, 8iyj.428.A, 8iyj.429.A, 8iyj.430.A, 8iyj.431.A, 8iyj.432.A, 8iyj.433.A, 8iyj.450.A, 8iyj.451.A, 8iyj.452.A, 8iyj.453.A, 8iyj.454.A, 8iyj.455.A, 8iyj.456.A, 8iyj.457.A, 8iyj.458.A, 8iyj.459.A, 8iyj.476.A, 8iyj.477.A, 8iyj.478.A, 8iyj.479.A, 8iyj.480.A, 8iyj.481.A, 8iyj.543.A, 8iyj.544.A, 8iyj.545.A, 8iyj.546.A, 8iyj.547.A, 8iyj.548.A, 8iyj.549.A, 8iyj.56.A, 8iyj.57.A, 8iyj.58.A, 8iyj.59.A, 8iyj.60.A, 8iyj.61.A, 8iyj.81.A, 8iyj.82.A, 8iyj.83.A, 8iyj.84.A, 8j07.1.A, 8j07.149.A, 8j07.152.A, 8j07.153.A, 8j07.156.A, 8j07.163.A, 8j07.2.A, 8j07.3.A, 8j07.31.A, 8j07.78.A, 8j07.844.A, 8j07.848.A, 8j07.849.A, 8j07.851.A, 8j07.852.A, 8j07.853.A, 8j07.854.A, 8j07.856.A, 8j07.857.A, 8j07.859.A, 8j07.860.A, 8j07.861.A, 8j07.862.A, 8j07.863.A, 8j07.864.A, 8j07.866.A, 8j07.867.A, 8j07.868.A, 8j07.869.A, 8j07.876.A, 8j07.877.A, 8j07.894.A, 8j07.896.A, 8j07.899.A, 8j07.908.A, 8j07.915.A, 8j07.916.A, 8j07.935.A, 8j07.937.A, 8j07.954.A, 8j07.956.A, 8j07.963.A, 8j07.965.A, 8p0s.1.A, 8p0s.1.B, 8p4y.1.A, 8ppl.1.L, 8q4g.1.G, 8q4g.1.H, 8q6t.1.A, 8q6t.1.B, 8q6t.1.H, 8q6t.1.N, 8q6t.1.O, 8q6t.1.Q, 8snb.124.A, 8snb.125.A, 8snb.126.A, 8snb.127.A, 8snb.128.A, 8snb.129.A, 8snb.130.A, 8snb.133.A, 8snb.135.A, 8snb.63.A, 8snb.64.A, 8srq.1.H, 8ssn.1.A, 8ssn.2.A, 8tek.1.A, 8tek.1.B, 8tek.1.D, 8tek.1.F, 8tek.1.I, 8th8.1.A, 8th8.1.D, 8th8.1.E, 8th8.1.J, 8th8.1.K, 8th8.1.P, 8th8.1.R, 8tid.1.1, 8tid.1.2, 8tid.1.3, 8tid.1.A, 8tid.1.B, 8tid.1.D, 8tid.1.E, 8tid.1.J, 8tid.1.K, 8tid.1.N, 8tid.1.P, 8tid.1.S, 8tid.1.T, 8tj5.1.U, 8tj5.1.V, 8tj5.1.W, 8tj5.1.Y, 8tj5.1.b, 8tj5.1.c, 8tj5.1.d, 8to0.18.A, 8to0.23.A, 8to0.397.A, 8to0.398.A, 8to0.400.A, 8to0.409.A, 8to0.410.A, 8to0.415.A, 8to0.439.A, 8to0.460.A, 8to0.462.A, 8to0.72.A, 8tvl.1.A, 8u95.1.A, 8u95.1.B
